# Lysing with Light: Trackable On Demand Molecular Delivery

**DOI:** 10.1021/jacs.6c07800

**Published:** 2026-06-15

**Authors:** Kavyasree Manal, Vasudev Deepa Sreekumar, Lakshmy Kannadi Valloli, Brieanna Lewis, Steffen Jockusch, Jayaraman Sivaguru

**Affiliations:** Center for Photochemical Sciences and Department of Chemistry, 1888 Bowling Green State University, Bowling Green, Ohio 43403, United States

## Abstract

A light-initiated lysing system is disclosed featuring a previously unknown and novel reactivity of enaminone that can be tuned at different wavelengths spanning both UV and visible regions. This novel reaction leads to on demand release of a desired cargo coupled with in situ generation of a fluorescent reporter.

## Graphical abstract

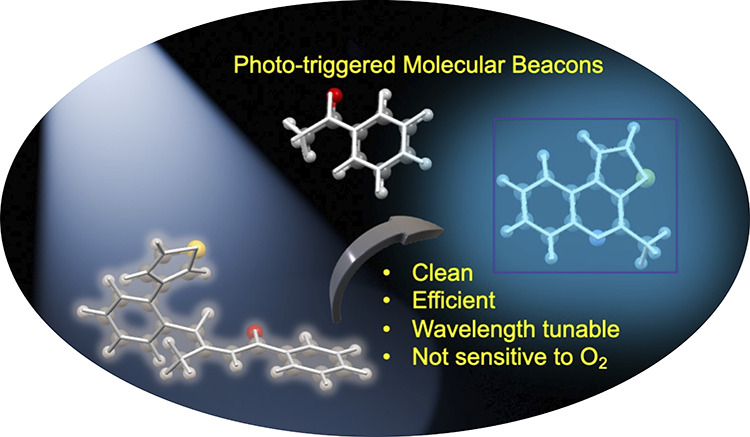

Nature utilizes light as a noninvasive energy pocket to influence biological events that are crucial to life sustenance.^1^ Tailoring light driven processes to stimulate biorelevant events require precise manipulation of excited state behavior of molecules.^2^ In particular, precise delivery of therapeutic chemicals to biosystem(s) using light and tracking the location of the delivery is challenging.^3,4^ In this report we disclose a new photoreaction of enaminones that can be tailored for delivery of molecules with concomitant tracking of the location of the delivered “cargo” based on the emissive signal of one of the photoproducts. This provides the ability to not only use light as an external stimulus to trigger the release of chemicals (phototrigger) but also offer avenues to track the location of the delivery using emissive signal from a fluorescent reporter that serves as a molecular beacon. The developed photoreaction features a light initiated lysing process that is clean and efficient which can be fine-tuned to different wavelengths (from UV to visible).

Currently available strategies for phototriggering chemicals^3^ can be broadly classified into three categories (Figure 1, top), viz., (a) cleavage-based phototrigger,^5–9^ (b) atom abstraction induced cleavage,^10,11^ and (c) photoisomerization induced cleavage.^12–19^ Over the years, various groups have tailored different systems^20–23^ for on demand light driven cleavage for drug delivery with varying efficiency. Developing phototriggering systems that is modular with customizable wavelength for delivery coupled with high efficiency along with the ability to track the location of delivery is still challenging. To address these issues, we have developed a novel excited state photochemical transformation featuring enaminone **3** (Figure 1, bottom). Enaminone **3** can be synthesized from simple starting materials (**1** and **2**; Scheme 1) and can be tuned to deliver a “cargo” of interest at a specific wavelength range (UV to visible). This light initiated lysing process featuring enaminone **3** has an added advantage of generating a quinoline fluorescent reporter **4** in situ with concomitant delivery of the “cargo/chemical” **5** of interest.

**Figure 1:**
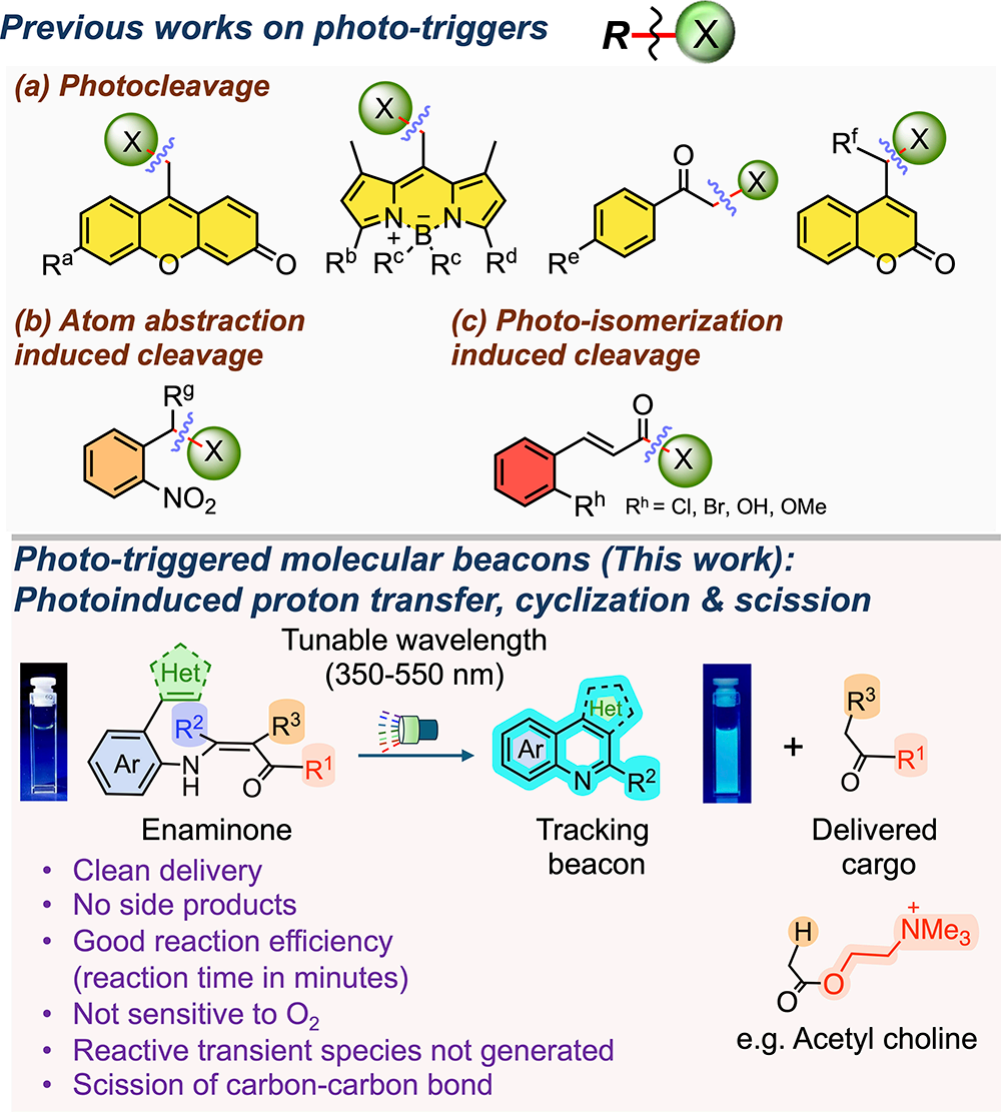
Phototriggering strategies (top) and the disclosed light-initiated lysing process with ability to track the delivery using an in situ generated molecular beacon (bottom).

**Scheme 1:**
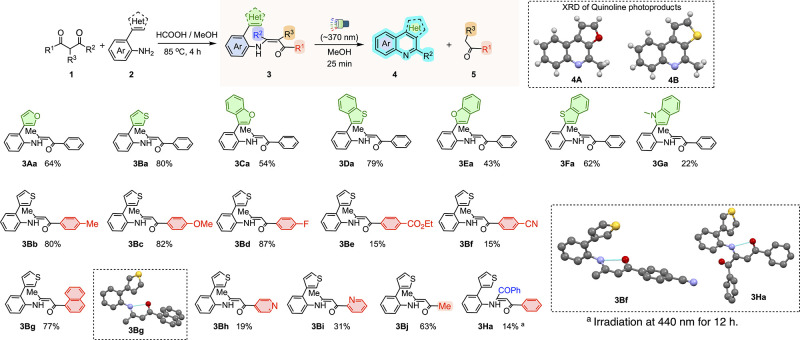
Evaluating the Scope of Light-Initiated Lysing Reaction of Enaminones^a^

a Yields are determined by ^1^H NMR spectroscopy with triphenyl methane as internal standard with an average of three trials (error ±5%).

We recently disclosed a novel photochemical reactivity of 1,3-dicarbonyl compounds with amino-styrene that underwent a photocyclization reaction bypassing the known De Mayo reaction.^24^ This novel reaction occurred through an in situ generated enaminone featuring an ultrafast excited state proton transfer process enabled by conical intersection on the excited state surface.^25^ As we understood the excited state trajectories of this transformation, we wanted to exploit this new reaction towards drug delivery. To accomplish this, changing the reaction trajectory in the excited state became crucial. We conjectured that substituting enaminones with a heteroaromatic functionality on the aryl ring will open up the possibility of cleaving the photogenerated intermediates which will unlock opportunities to develop a new light driven delivery system. To evaluate this possibility, we synthesized a series of enaminones **3Aa**–**3Ga** featuring heteroaromatic functionality on the aryl ring and evaluated its photoreactivity by exposing to UV and/or visible light.^26^ We began our investigation by utilizing **3Ba** that featured a 3-thienyl substitution on the aryl ring. Irradiation of **3Ba** in methanol with ∼370 nm LED resulted in complete consumption of the starting material after 25 min of irradiation. In addition, during the course of the photoreaction, there was a concomitant development of fluorescence. The formation of the photoproduct was investigated by various analytical techniques,^26^ viz., NMR spectroscopy, HRMS, and single crystal XRD, that revealed a light initiated lysing reaction resulting in quinoline **4B** and acetophenone **5a**. The structure of **4B** was unequivocally established by single crystal XRD (Scheme 1) and the identity of **5a** was established by comparison with an authentic sample. The overall yield of the reaction was 80% (based on **4B**). Control studies in the absence of light at both room (dark reaction for 24 h) and elevated temperatures (62 °C; 12 h; Figure S6)^26^ did not yield any photoproduct with recovery of the enaminone reactant.

Replacing the 3-thiophene unit by a 3-furanyl unit, i.e., enaminone **3Aa**, resulted in similar reactivity, leading to the formation of quinoline **4A** (yield, 64%) and acetophenone **5a**. Employing 3-benzofuranyl derivate **3Ca** and 3-benzothienyl derivative **3Da** resulted in the corresponding quinoline **4C** (yield, 54%) and **4D** (yield, 79%), respectively, along with acetophenone **5a**. Changing the substitution pattern on the benzofuranyl and benzothienyl motifs from the 3-position to the 2-position, i.e., corresponding 2-benzofuranyl derivative **3Ea** and 2-benzothienyl derivative **3Fa**, resulted in analogous reactivity. Photoreaction of 2-benzofuranyl derivative **3Ea** led to quinoline **4E** (yield, 43%) and acetophenone **5a**. For 2-benzothienyl derivative **3Fa**, quinoline **4F** (yield, 62%) and the acetophenone **5a** were observed. The reaction was also compatible with changing the heteroaromatic substituent to a nitrogen (**3Ga**). Irradiation of **3Ga** featuring indole substitution resulted in quinoline **4G** (yield, 22%) and acetophenone **5a**.

As 3-thienyl substituted enaminone **3Ba** gave high yields, we kept that functionality on the enaminone and systematically varied the aryl substituent on the enone carbonyl, i.e., enaminones **3Bb**–**3Bj** (Scheme 1). Photoreaction of 3-thienyl enaminones with electron-donating aryl substituents on the enone-carbonyl motif gave high conversions and yields compared to electron-withdrawing aryl substituents on the enone-carbonyl unit. Irradiation of enaminones featuring *p*-methyl (**3Bb**) and *p*-methoxy (**3Bc**) substituents resulted in higher yields of the quinoline photoproduct **4B** (yield: **3Bb** = 80%, **3Bc** = 82%) and the corresponding acetophenone derivatives **5b** and **5c**, respectively. Irradiation of enaminones featuring electron-withdrawing substituents *p*-COOEt (**3Be**) and *p*-cyano (**3Bf**) resulted in diminished yields (**3Be** yield, 15%; **3Bf** yield, 15%) of quinoline **4B** and the corresponding acetophenone derivatives **5e** and **5f**, respectively. Incorporating a pyridyl unit on an enone-carbonyl motif also led to lower yields of quinoline **4B** (yield: **3Bh** = 19% and **3Bi** = 31%) and the corresponding acetylpyridine derivatives **5h** and **5i**, respectively. Incorporating *p*-fluorophenyl substituent **3Bd** on the enone-carbonyl unit led to higher conversions and yields of quinoline **4B** and *p*-fluoroacetophenone **5d** (conv. 100%, yield = 87%). This enaminone was utilized for evaluating the influence of solvent (Figure 2A) due to practical considerations (*p*-fluoroacetophenone **5d** was not volatile). The reaction was also flexible for incorporation of a naphthyl unit (**3Bg**) on the enone carbonyl functionality. Irradiation of **3Bg** resulted in quinoline **4B** (yield, 77%; conv. 100%) and acetylnaphthone **5g**. The light-initiated lysing reaction of **3Bg** in the presence of oxygen and air did not significantly alter the yield of quinoline **4B** (yield: 75% under aerated conditions and 61% under oxygen atmosphere; Table S4).^26^ Naphthyl-substituted enaminone **3Bg** was utilized for photophysical studies (Figure 3A–D) due to ease of following excited-state processes with the naphthyl unit as a handle (Figure 3).

**Figure 2:**
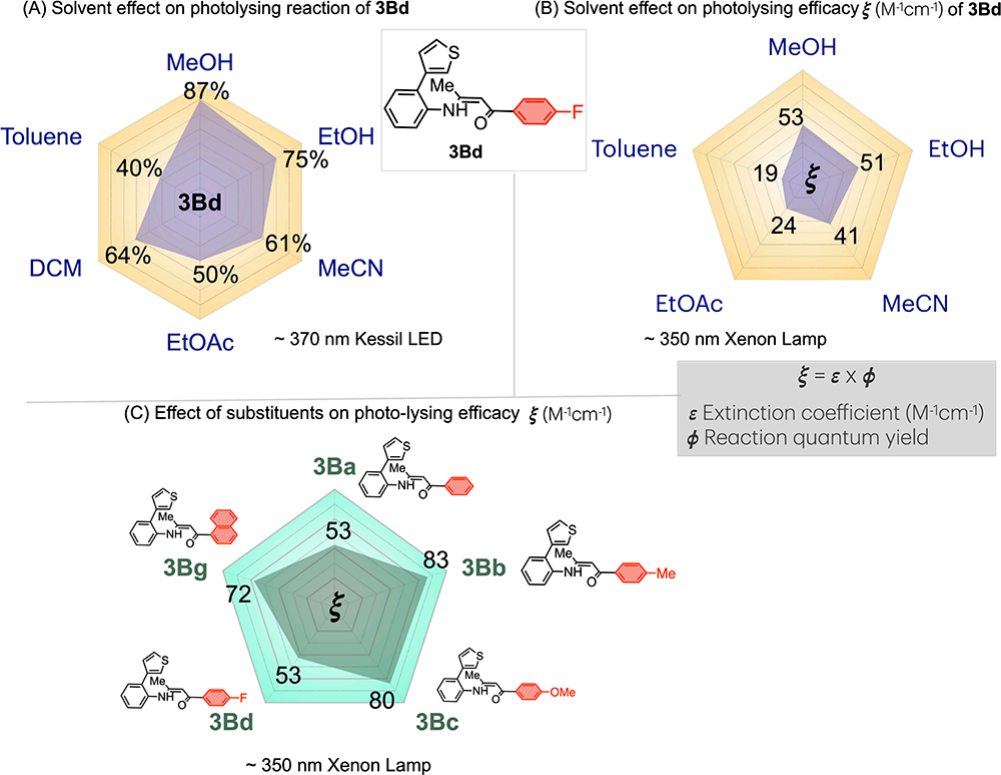
Effect of solvents (A) and efficacy (B, C) of light initiated lysing reaction of enaminones.

**Figure 3:**
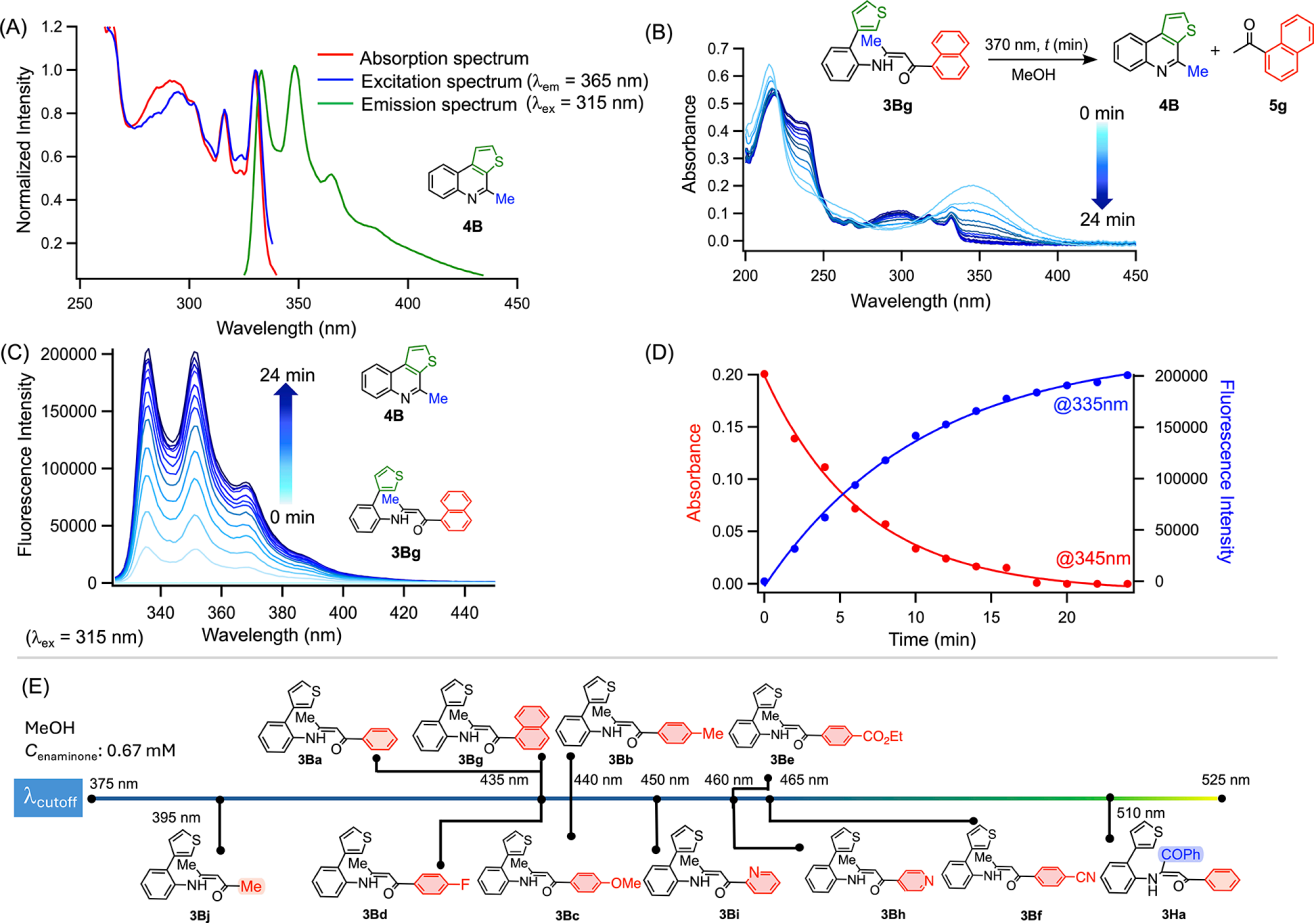
Photophysical features of **4B** in acetonitrile (A). Monitoring photolyzing process of **3Bg** by absorption (B) and emission (C) spectroscopy. (D) Reaction progress by monitoring quinoline emission at 335 nm (blue trace) and enaminone absorption at 345 nm (red trace). (E) Absorption wavelength cutoff (where absorptivity is close to zero) of different enaminones.

To gauge the influence of solvents, we utilized enaminone **3Bd** and evaluated the efficiency by monitoring the yield of the quinoline photoproduct **4B**. Irradiation of **3Bd** in polar protic solvents methanol and ethanol gave **4B** in 87% and 75% yields, respectively (Figure 2A). Changing from protic to aprotic polar acetonitrile resulted in 61% yield of **4B**. Lowering the polarity of the solvent resulted in moderate yields of **4B** with ethyl acetate, dichloromethane, and toluene giving 50%, 64%, and 40% yields, respectively (Figure 2A). Lowering the temperature resulted in lowering the efficiency of the light initiated lysing process. Irradiation of **3Bd** in methanol at −40 °C resulted in 57% yield of **4B** compared to 87% yield at ambient temperature (Table S4). We observed a bright fluorescence during the light initiated lysing process, and we attributed this emission to originate from the quinoline photoproduct **4**. To establish this, we analyzed the photophysical features (Figure 3A), viz., absorption (red trace), fluorescence excitation (blue trace), and emission spectra (green trace) of quinoline **4B** in acetonitrile. Quinoline **4B** exhibited a structured emission with a fluorescence quantum yield of 0.037.^26^

The excitation spectra of this emission matched the absorption profile (Figure 3A), indicating that the emission observed during the light-initiated lysing process has its origin in the quinoline photoproduct **4**. In order to quantify the light-initiated lysing process, we irradiated enaminone **3Bg** with ∼370 nm Kessil LEDs (Figure 3B) for different time intervals (2–24 min).

A gradual disappearance of enaminone absorption peak at ∼350 nm was observed with concurrent formation of new peak at ∼330 nm (Figure 3B). Upon photolysis, a prominent emission was observed (Figure 3C) that resembled the emission of quinoline **4B**. This emission gradually increased at higher reaction conversions (Figure 3C). A plot of formation of quinoline **4B** monitored at 335 nm by emission spectroscopy (Figure 3D, blue trace) and disappearance of enaminone **3Bg** monitored at 345 nm by UV–vis spectroscopy (Figure 3D, red trace) over a period of 24 min (Figure 3D) revealed that the observed emission correlates with consumption of the reactant leading to the formation of the quinoline photoproduct. The quantum yield and the light initiated lysing reaction efficiency were determined for the system (Figure 2B) using potassium ferrioxalate as actinometer with 350 nm irradiation (Figures S8–S13, Table S5),^26–28^ and the efficacy values (considering the extinction coefficient) of light initiated lysing process was calculated, which was found to vary from 53 (**3Ba** and **3Bd**) to 83 M^–1^ cm^–1^ (**3Bb**).^3^ Such efficient release enables us to test the system for delivery of biologically relevant molecules.

Having developed a highly wavelength tunable and efficient light initiated lysing process that can be initiated by UV and/or visible light, we propose a mechanism for the process as detailed in Scheme 2. We have previously shown that β-enaminone undergoes excited state intramolecular proton transfer (ESIPT)^24^ in the ultrafast time scales (subpicosecond).^25^ We conjecture that ESIPT in β-enaminones **3** with heteroatomic substitutions leads to imine-enol-**3** (Scheme 2). This imine-enol-**3** undergoes double dearomative cyclization to form the int-cyclic-imine-enol-**3**. This int-cyclic-imine-enol-**3** tautomerizes to form int-cyclic-keto-imine-**3** that subsequently rearomatizes to form quinoline **4** and enol-**5**. This enol-**5** tautomerizes to the ketone product **5** (Scheme 2).

**Scheme 2:**
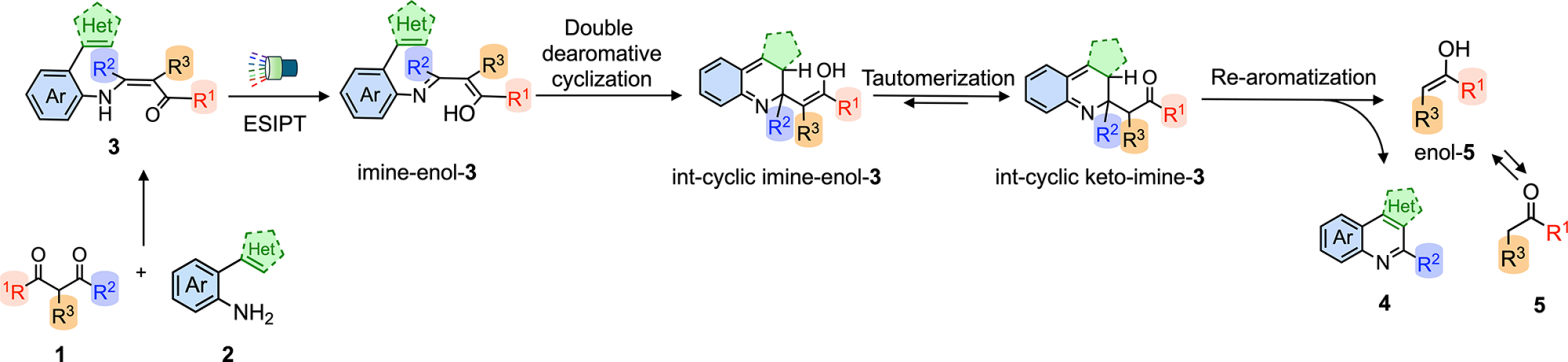
Mechanism of Light Initiated Lysing Reaction of Enaminones

To showcase the broad utility of the newly discovered light initiated lysing system, we evaluated the release of biomolecules with light. As reaction was also amenable to alkyl substitution on the enaminone-carbonyl unit (Scheme 1; enaminone **3Bj**: 100% conversions, yield: 63%), we extended our system for release of biologically relevant scaffolds **3Bk**-**3Bm** (Scheme 3). The light initiated lysing reaction of enaminones appended to biomolecules were monitored by HRMS (Figures S20−S28, Scheme 3). Irradiation of enaminone **3Bk** resulted in the formation of acetyl choline **5k** and the corresponding quinoline **4B** that served as a fluorescent reporter. We were successful in utilizing our light initiated lysing process for on demand release of anticancer drugs acetyl silybin **5l** (acetylated silybin derivatives have a reported IC_50_ varying from 22 to 473 μM for HepG2 human hepatocellular [liver] carcinoma cell line)^29^ and acetyl taxol **5m** (e.g., 7-acetyl taxol has a reported IC_50_ of 94 nM for A549/T cell line)^30^ by irradiating the corresponding enaminone **3Bl** and **3Bm** (Scheme 3) with concomitant formation of quinoline **4B** as the fluorescent reporter. Thus, our newly developed light initiated lysing process is effective for on demand release of biologically relevant molecules (Scheme 3). For broad application especially in the field of drug delivery, having absorption beyond the UV region is ideal. The systematic variation of absorptivity was evaluated for various substituted enaminones (Figure 3E). By incorporating a substituent on the β-position of the enaminone functionality i.e., enaminone **3Ha**, a bathochromic shift in absorption was observed (absorptivity cutoff ∼510 nm, Figures S2 and S3; Table S1).^26^ Irradiation of enaminone **3Ha** in acetonitrile at 440 nm led to quinoline fluorescent reporter **4H** (characterized by ^1^H NMR spectroscopy and HRMS; Figures S54 and S55) with 14% yield along the release of acetophenone **5a** and other unidentified side products, showcasing that the system can be fine-tuned to work at different wavelengths.

**Scheme 3:**
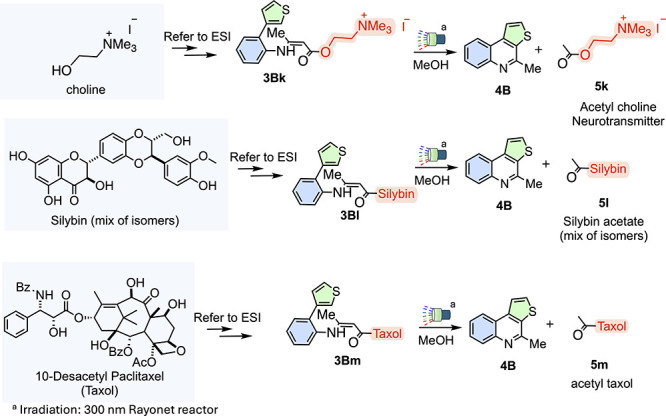
Light-Initiated Lysing Reaction Featuring Biologically Relevant Motifs

In summary, our work has led to the development of a novel light initiated lysing platform that has broad applicability. The newly developed excited state process featuring enaminone is broad in scope and applicability. The reaction is amenable to various structural features that can be fine-tuned for light initiated lysing process at various wavelengths. The quantum yield of the lysing process was ∼0.003 (efficacy ∼83 M^–1^ cm^–1^) at 350 nm with a fluorescence quantum yield of the quinoline beacon being ∼0.04. In addition, the reaction can also be carried out in water for release of biological relevant scaffolds. With the ability to track the release of such molecular/biomolecular scaffolds using fluorescent reporter coupled with the flexibility to tune the wavelength of the lysing process, the newly disclosed photoreleasing platform will have broad applicability in various disciplines which are being currently pursued in our laboratory.

## Supplementary Material



## Notes

Synthetic protocol, reaction procedures, and characterization of compounds (PDF)
