# Supplementary material for: Lysing with Light: Trackable On Demand Molecular Delivery
Source: J Am Chem Soc. 2026 Jun 15;148(27):28043–8. doi: 10.1021/jacs.6c07800 (PMC13383719; doi:10.1021/jacs.6c07800)
Supplement: Supplementary file 1 [file ja6c07800_si_001.pdf]

Lysing with Light: Trackable On Demand Molecular Delivery

Kavyasree Manal[a], Vasudev Deepa Sreekumar[a], Lakshmy Kannadi Valloli[b], Brieanna Lewis[c], Steffen Jockusch[a], Jayaraman Sivaguru*[a]

Corresponding author: sivagj@bgsu.edu

Table of Contents

1. General methods 5

2. Chemical structures of photoproducts and its precursors used in the photoreactions. 6

3. Setup for photoreaction of enaminones. 7

4. General procedure for the synthesis of substrates. 8

4.1. Synthesis of 1,3-diketones ***1b-1i****.* 8

4.2. Synthesis of diketone ***1k****.* 10

4.3. Synthesis of diketone ***1n****.* 11

4.4. Synthesis of o-(aryl)aniline ***2A-2F****.* 12

4.5. Synthesis of o-(N-methyl indole)aniline ***2G****.* 15

4.6. General procedure for the synthesis of enaminones ***3****.* 16

4.7. Synthesis of enaminone ***3Ha****.* 22

5. UV-vis absorption spectrum of enaminones. 23

5.1. Plot of extinction coefficient vs wavelength of enaminones ***3B-3Bk, 3Ha****.* 23

5.2. UV-vis absorption spectra of enaminones ***3*** *at reaction concentration.* 24

5.3. UV-Vis absorption spectra of enaminone ***3Bd*** *in different solvents.* 25

6. Irradiation procedure. 26

7. Solvent optimization for the photoreaction of enaminone 28

8. Control studies of enaminone 3Bg under dark and thermal conditions 29

9. Photoreaction of enaminones at different conditions. 30

10. Determination of fluorescence quantum yield 31

11. Monitoring the photolysis reaction of enaminone by ^{1}H NMR spectroscopy 32

12. Quantum yield studies of enaminones. 33

12.1. Photolysis of actinometer. 33

*12.2. Photolysis of enaminones **3B**. 34*

12.2.1 Photolysis of enaminone **3Ba**. 34
12.2.2 Photolysis of enaminone **3Bb**. 34
12.2.3 Photolysis of enaminone **3Bc**. 35
12.2.4 Photolysis of enaminone **3Bd**. 35
12.2.5 Photolysis of enaminone **3Bg**. 36

12.3. Determination of quantum yield and efficacy of photoreaction. 37

*12.4. Solvent study on photolysis of enaminone **3Bd**. 38*

12.4.1 Photolysis of enaminone **3Bd** in methanol 38
12.4.2 Photolysis of enaminone **3Bd** in methanol 38
12.4.3 Photolysis of enaminone **3Bd** in ethanol 39
12.4.4 Photolysis of enaminone **3Bd** in acetonitrile 39
12.4.5 Photolysis of enaminone **3Bd** in ethyl acetate 40
12.4.6 Photolysis of enaminone **3Bd** in toluene 40

13. Photolysis of enaminones 3Bl, 3Bm, 3Bn 41

*13.1. Photolysis of enaminone **3Bk**. 41*

*13.2. Photolysis of enaminone **3Bk** in water. 43*

*13.3. Photolysis of enaminone **3Bl**. 43*

*13.4. Photolysis of enaminone **3Bm**. 45*

14. Photolysis of enaminone 3Ha 48

15. Emission study of photoproduct. 49

16. Emission study on photolysis of enaminone. 50

17. Characterization of photoproducts. 51

*17.1. Characterization of photoproduct **4A**. 51*

*17.2. Characterization of photoproduct **4B**. 54*

*17.3. Characterization of photoproduct **4C**. 58*

*17.4. Characterization of photoproduct **4D**. 61*

*17.5. Characterization of photoproduct **4E**. 64*

*17.6. Characterization of photoproduct **4F**. 67*

*17.7. Characterization of photoproduct **4G**. 70*

*17.1. Characterization of photoproduct **4H**. 73*

18. Spectroscopic characterization of precursors used for synthesis. 75

*18.1. Characterization of 1,3-diketone **1b**. ... 75*
*18.2. Characterization of 1,3-diketone **1c**. ... 77*
*18.3. Characterization of 1,3-diketone **1d**. ... 79*
*18.4. Characterization of 1,3-diketone **1e**. ... 81*
*18.5. Characterization of 1,3-diketone **1f**. ... 83*
*18.6. Characterization of 1,3-diketone **1g**. ... 85*
*18.7. Characterization of 1,3-diketone **1h**. ... 87*
*18.8. Characterization of 1,3-diketone **1i**. ... 89*
*18.9. Characterization of 1,3-diketone **8**. ... 91*
*18.10. Characterization of 1,3-diketone **1k**. ... 93*
*18.11. Characterization of diketone **1n**. ... 95*
*18.1. Characterization of 2(3-furan)benzenamine **2A**. ... 97*
*18.2. Characterization of 2(3-thienyl)benzenamine **2B**. ... 99*
*18.1. Characterization of 2(3-benzofuranyl)benzaniline **2C**. ... 101*
*18.1. Characterization of 2(3-benzothienyl)benzaniline **2D**. ... 103*
*18.1. Characterization of 2-(2-benzofuranyl)benzaniline **2E**. ... 105*
*18.1. Characterization of 2-(2-benzothienyl)benzaniline **2F**. ... 107*
*18.1. Characterization of 1-(2-Aminophenyl)ethanone 2-methyl-2-phenylhydrazone **15**. ... 109*
*18.1. Characterization of 2-(1-Methyl-1H-indol-2-yl)benzenaniline **2G**. ... 111*
*18.2. Characterization of enaminone **3Aa**. ... 113*
*18.3. Characterization of enaminone **3Ba**. ... 116*
*18.1. Characterization of enaminone **3Ca**. ... 119*
*18.1. Characterization of enaminone **3Da**. ... 122*
*18.1. Characterization of enaminone **3Ea**. ... 125*
*18.2. Characterization of enaminone **3Fa**. ... 128*
*18.3. Characterization of enaminone **3Ga**. ... 131*
*18.4. Characterization of enaminone **3Bb**. ... 134*
*18.1. Characterization of enaminone **3Bc**. ... 137*
*18.2. Characterization of enaminone **3Bd**. ... 140*
*18.3. Characterization of enaminone **3Be**. ... 143*
*18.1. Characterization of enaminone **3Bf**. ... 146*
*18.1. Characterization of enaminone **3Bg**. ... 149*
*18.2. Characterization of enaminone **3Bh**. ... 152*
*18.1. Characterization of enaminone **3Bi**. ... 155*
*18.2. Characterization of enaminone **3Bj**. ... 158*

*18.1. Characterization of enaminone **3Bk**.* *161*
*18.1. Characterization of enaminone **3Ha**.* *165*

19. XRD analysis........ 168
*19.1. Structure of enaminone **3Bf**.* *169*
*19.2. Structure of enaminone **3Bg**.* *169*
*19.3. Structure of enaminone **3Ha**.* *169*
*19.4. Structure of quinoline photoproduct **4A**.* *170*
*19.5. Structure of quinoline photoproduct **4B**.* *170*

20. References. 171

1. General methods

All commercially obtained reagents/solvents were used as received; chemicals were purchased from Alfa Aesar®, Sigma-Aldrich®, Acros organics® ,Ambeed® and TCI America® and were used as received without further purification. Spectrophotometric grade solvents (e.g. ethanol) were purchased from Sigma-Aldrich® and used without further purification for emission measurements. Unless stated otherwise, reactions were conducted in oven-dried glassware under nitrogen atmosphere. ^{1}H-NMR and ^{13}C-NMR spectra were recorded on Bruker 500 MHz and/or 600 MHz (126 MHz and/or 151 MHz for ^{13}C-NMR) spectrometers. Data from the NMR spectroscopic measurements are reported as chemical shift (δ ppm) with the corresponding integration values. Coupling constants (*J*) are reported in hertz (Hz). Standard abbreviations indicating multiplicity were used as follows: s (singlet), d (doublet), t (triplet), q (quartet) and m (multiplet). Data for ^{13}C-NMR spectra are reported in terms of chemical shift (δ ppm). High-resolution mass spectrometry (HRMS) was performed using a Waters XeVo G3 QToF instrument, using Electron Spray Ionization (ESI) on positive mode. When necessary, the compounds were purified by chromatography using a Combiflash SI-3 system equipped with dual wavelength UV-Vis absorbance detector (Teledyne ISCO) using hexanes/ethyl acetate solvent mixture as the mobile phase and Redisep® cartridge filled with silica (Teledyne ISCO) as stationary phase. Unless indicated, the retardation factor (R*f*) values were recorded using a 5-50% ethyl acetate: hexanes as mobile phase and on Sorbent Technologies®, silica gel TLC plates (200 mm thickness w/UV_{254}).

Photophysical Methods: Spectrophotometric solvents (Sigma-Aldrich®) were used whenever necessary unless or otherwise mentioned. UV quality fluorimeter cells (with range until 190 nm) were purchased from Starna cells®. Absorbance measurements were performed using a Cary 300 UV-Vis spectrophotometer. Steady state and time resolved emission measurements were performed in Edinburg instrument FLS 1000.

X-ray crystal structure determination: Single crystal X-ray diffraction data of the compounds **3Bf**, **3Bg**, **3Ha**, **4A** and **4B** were collected on a Bruker Apex Duo diffractometer with a Photon III detector at T =300K. Cu radiation was used. All structures were processed with Apex 3 v2019.1-0 software package (SAINT v. 8.38A) and Olex 2 v 1.3.0 [1]. XT [2] structure solution program based on Intrinsic Phasing was used to solve the structures after multi-scan absorption corrections and refined with the XL [3] refinement package using Least Squares minimization.

2. Chemical structures of photoproducts and its precursors used in the photoreactions.

Chart 1

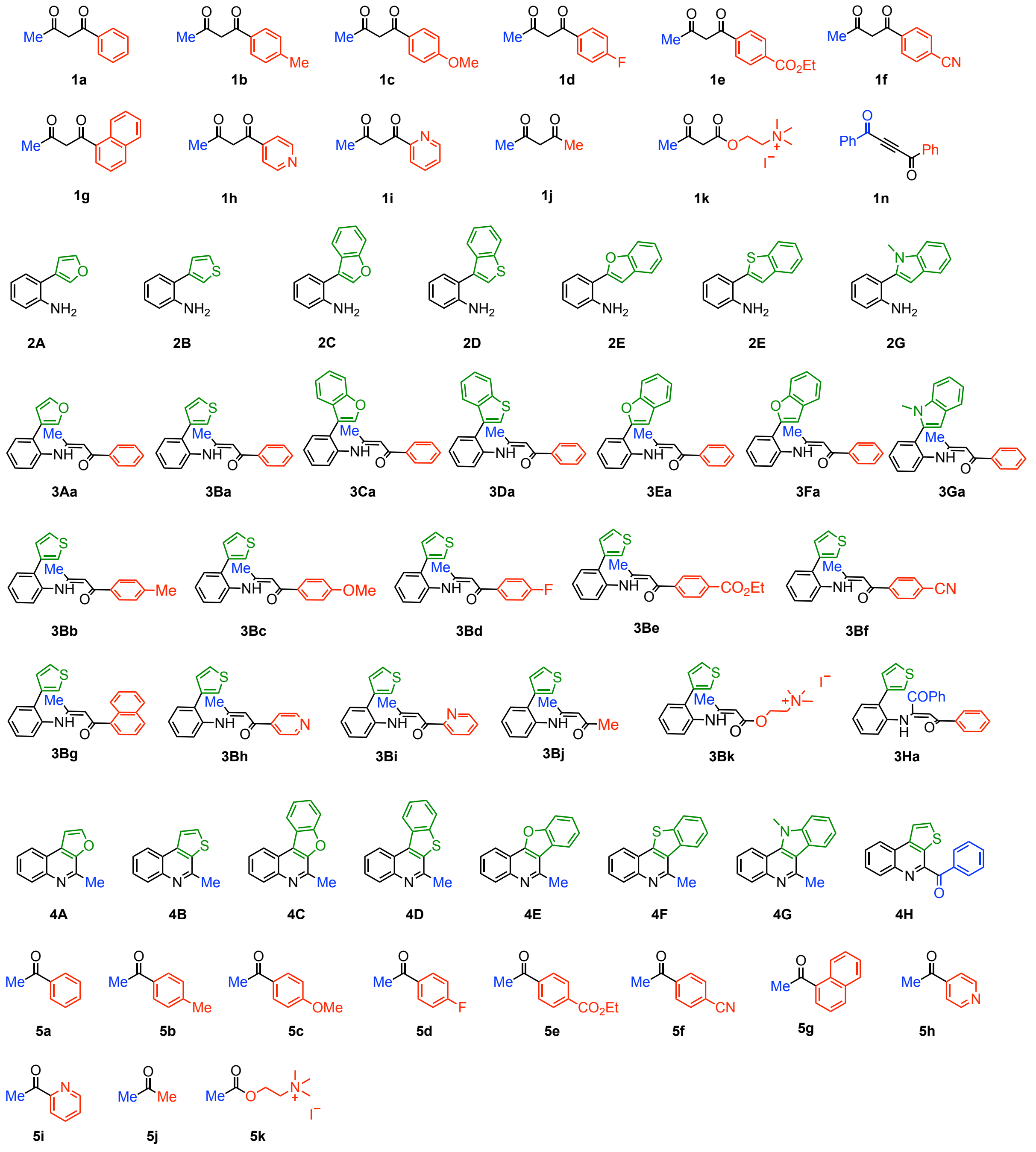

3. Setup for photoreaction of enaminones.

Method 1: Photoreactions were carried out in Pyrex tube using two Kessil LEDs placed at a distance of 6 cm from the sample. 370 nm Gen 2, 390 nm and 427 nm Kessil LEDs were employed for the reaction with 100% power. The reaction mixture is allowed to stir throughout the course of the reaction and along with a fan kept in front of the reaction to cool the reaction mixture.

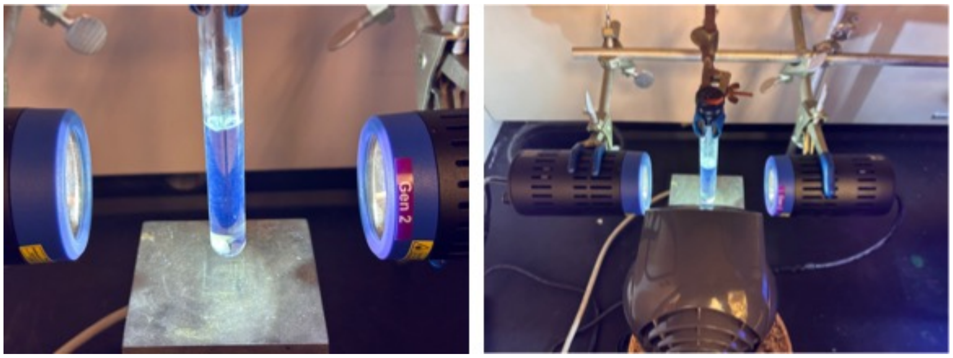

Figure S1: General photoreaction setup with 370 nm Gen 2 Kessil LED.

Method 2: Rayonet reactor (Southern New England Ultraviolet Co. - RPR-100 / RPR-200) was employed for carrying out reactions at ~300 nm and ~350 nm. Reactions were carried out in Pyrex tube and kept inside Rayonet reactor equipped with merry-go-round.

4. General procedure for the synthesis of substrates.

4.1. Synthesis of 1,3-diketones **1b-1i**.

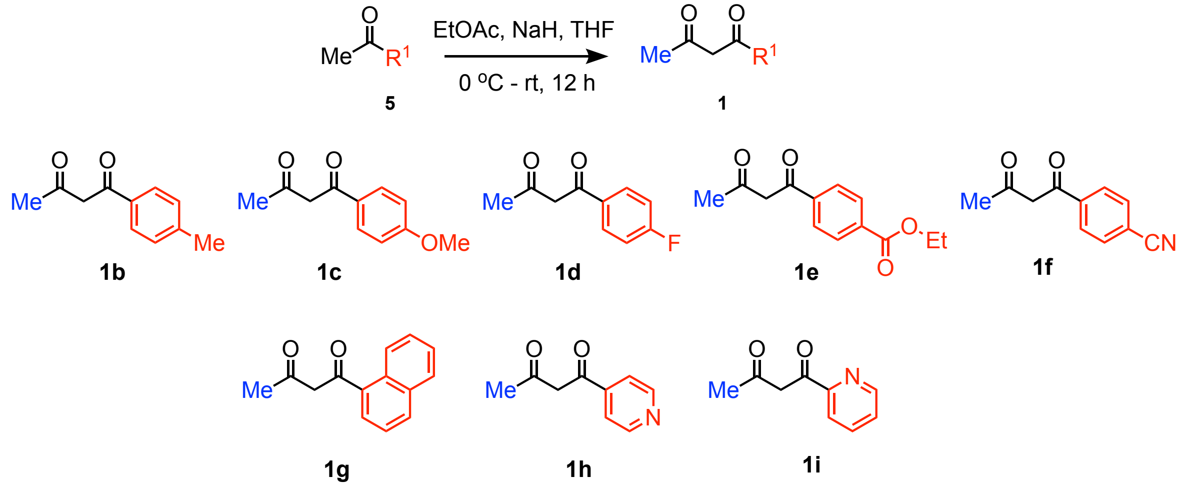

Scheme S1: Synthesis of 1,3-diketones **1**.

1,3-diketone **1** was synthesized by following the literature reported procedure.[1] A solution of compound **5** (6.6 mmol, 1 equiv.) in ethyl acetate (5 mL) was added to the suspension of sodium hydride (26.4 mmol, 4 equiv.) in ethyl acetate (5 mL) at 0 °C and stirred at room temperature for 12 h. Completion of reaction was monitored by TLC. A saturated solution of ammonium chloride (20 mL) was then added to the crude reaction mixture followed by acidification with dil. HCl (20 mL of 10% aqueous HCl solution). Organic layer was separated and the aqueous layer was extracted with ethyl acetate (3 x 10 mL). Combined organic layers were washed with 10 mL of brine solution and dried over anhydrous sodium sulfate and concentrated under reduced pressure. The crude product was purified by chromatography (Combiflash) using ethyl acetate/hexanes mixture as mobile phase.

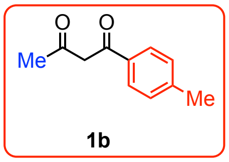

R_f = 0.5 (90% hexanes:10% ethyl acetate). Yield = 89%.

^{1}H NMR (500 MHz, $CDCl_3$) δ 7.81 (d, J = 8.5 Hz, 2H), 7.27 (d, J = 8.5 Hz, 2H), 6.18 (s, 1H), 2.43 (s, 3H), 2.21 (s, 3H). Enolizable proton resonances not identified.

^{13}C NMR (126 MHz, $CDCl_3$) δ 193.08, 183.76, 143.08, 132.19, 129.36, 127.09, 96.34, 25.71, 21.64.

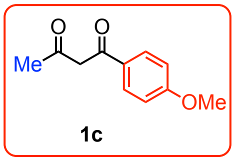

R_f = 0.5 (90% hexanes:10% ethyl acetate). Yield = 97%.

^{1}H NMR (500 MHz, $CDCl_3$) δ 7.86 (d, J = 8.9 Hz, 2H), 6.94 (d, J = 8.9 Hz, 2H), 6.11 (s, 1H), 3.86 (s, 3H), 2.17 (s, 3H). Enolizable proton resonances not identified.

^{13}C NMR (126 MHz, $CDCl_3$) δ 191.62, 184.15, 163.09, 129.13, 127.55, 113.93, 95.80, 55.47, 25.33.

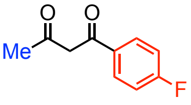

1d

R_f = 0.5 (90% hexanes:10% ethyl acetate). Yield = 94%.

^{1}H NMR (500 MHz, $CDCl_3$) δ 7.90 (dd, J = 9.0, 5.4 Hz, 2H), 7.13 (t, J = 8.7 Hz, 2H), 6.13 (s, 1H), 2.20 (s, 3H). Enolizable proton resonances not identified.

^{13}C NMR (126 MHz, $CDCl_3$) δ 192.92, 182.97, 166.33 and 164.32 ($J_{C\text{-}F}$ = 253.3 Hz), 131.28 and 131.25 ($J_{C\text{-}F}$ = 3.8 Hz), 129.50 and 129.43 ($J_{C\text{-}F}$ = 8.8 Hz), 115.88 and 115.70 ($J_{C\text{-}F}$ = 22.7 Hz), 96.40, 25.59.

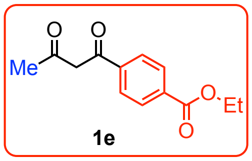

1e

R_f = 0.5 (90% hexanes:10% ethyl acetate). Yield = 81%.

^{1}H NMR (500 MHz, $CDCl_3$) δ 8.10 (d, J = 8.5 Hz, 2H), 7.92 (d, J = 8.5 Hz, 2H), 6.21 (s, 1H), 4.40 (q, J = 7.1 Hz, 2H), 2.23 (s, 3H), 1.41 (t, J = 7.1 Hz, 3H). Enolizable proton resonances not identified.

^{13}C NMR (126 MHz, $CDCl_3$) δ 195.44, 181.05, 165.86, 138.53, 133.49, 129.77, 126.85, 97.55, 61.42, 26.35, 14.32.

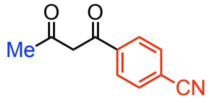

1f

R_f = 0.3 (90% hexanes:10% ethyl acetate). Yield = 86%.

^{1}H NMR (500 MHz, $CDCl_3$) δ 7.95 (d, J = 8.7 Hz, 2H), 7.79 – 7.65 (m, 2H), 6.19 (s, 1H), 2.24 (s, 3H). Enolizable proton resonances not identified.

^{13}C NMR (126 MHz, $CDCl_3$) δ 195.86, 179.76, 138.74, 132.43, 127.42, 118.13, 115.38, 97.70, 26.40.

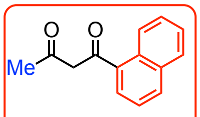

1g

R_f = 0.5 (90% hexanes:10% ethyl acetate). Yield = 88%.

^{1}H NMR (500 MHz, $CDCl_3$) δ 8.46 (dd, J = 9 Hz, 1.0 Hz, 1H), 7.96 (d, J = 8.2 Hz, 1H), 7.89 (dd, J = 9 Hz, 1.1 Hz, 1H), 7.72 (dd, J = 7.1, 1.2 Hz, 1H), 7.60 – 7.52 (m, 2H), 7.50 (dd, J = 8.2, 7.2 Hz, 1H), 6.04 (s, 1H), 2.22 (s, 3H). Enolizable proton resonances not identified.

^{13}C NMR (126 MHz, $CDCl_3$) δ 192.48, 188.32, 134.29, 133.82, 131.67, 130.13, 128.51, 127.23, 126.98, 126.37, 125.57, 124.78, 101.75, 25.45.

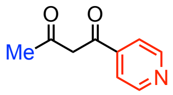

1h

R_f = 0.3 (90% hexanes:10% ethyl acetate). Yield = 56%.

^{1}H NMR (500 MHz, $CDCl_3$) δ 8.79 – 8.78 (m, 2H), 7.76-7.75 (m, 2H), 6.25 (s, 1H), 2.28 (s, 3H). Enolizable proton resonances not identified.

^{13}C NMR (126 MHz, $CDCl_3$) δ 196.84, 178.77, 150.65, 141.72, 120.28, 97.84, 26.69.

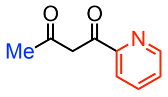

1i

R_f = 0.3 (90% hexanes:10% ethyl acetate). Yield = 80%.

^{1}H NMR (500 MHz, $CDCl_3$) δ 8.67 – 8.65 (m, 1H), 8.08 – 8.07 (m, 1H), 7.84 – 7.83 (m, 1H), 7.41 (m, 1H), 6.82 (d, J = 3.7 Hz, 1H), 2.24 (s, 3H). Enolizable proton resonances not identified.

^{13}C NMR (126 MHz, $CDCl_3$) δ 195.14, 180.83, 152.14, 149.28, 137.06, 126.26, 122.12, 97.28, 26.13.

4.2. Synthesis of diketone **1k**.

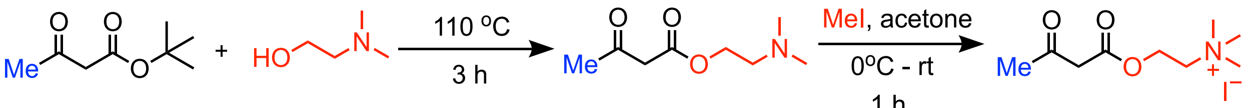

6 **7** **8** **1k**

Scheme S2: Synthesis of 1,3-diketones **1k**.

1,3-diketone **8** was synthesized following literature reported procedure.[2-3] A mixture of tert-butyl acetoacetate (4.5 mmol, 1.5 equiv.) and 2-(Dimethylamino)ethanol (3 mmol, 1 equiv.) is heated at 100 °C for 3 h. After completion of the reaction, the crude product was purified by chromatography (Combiflash) using ethyl acetate/hexanes mixture as mobile phase.

1,3-diketone **1k** was synthesized following similar literature reported procedure.[3] Methyl iodide (0.520 mmol, 1.5 equiv.) was added to a solution of 1,3-diketone **8** (0.346 mmol, 1 equiv.) in dry acetone kept at 0 °C. The resulting mixture was stirred at room temperature for 1 h. After the completion of reaction, crude reaction mixture was filtered and washed using acetone to obtain the product **1k**.

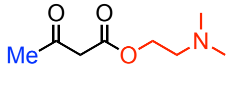

8

R_f = 0.3 (100% ethyl acetate). Yield = 73%.

^{1}H NMR (500 MHz, $CDCl_3$) δ 4.24 (t, J = 5.7 Hz, 2H), 3.49 (s, 2H), 2.57 (t, J = 5.7 Hz, 2H), 2.27 (d, J = 2.5 Hz, 9H).

^{13}C NMR (126 MHz, $CDCl_3$) δ 200.62, 167.25, 62.93, 57.66, 50.06, 45.65, 30.17.

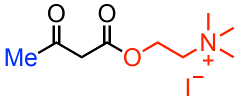

1k

R_f = 0.3 (95% ethyl acetate, 5% methanol). Yield = 80%.

^{1}H NMR (500 MHz, CD_3CN) δ 4.48 (dd, J = 4.5, 2.1 Hz, 2H), 3.58 (s, 4H), 3.11 (d, J = 8.8 Hz, 9H), 2.20 (s, 3H).

^{13}C NMR (126 MHz, CD_3CN) δ 201.26, 166.69, 64.53, 58.22, 53.84, 49.33, 29.64.

4.3. Synthesis of diketone **1n**.

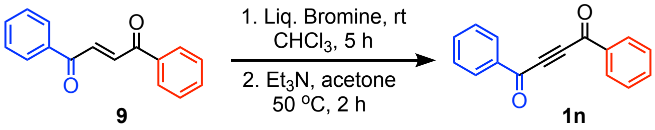

Scheme S3: Synthesis of diketone **1n**.

Diketone **1n** was synthesized following literature reported procedure.[4] To a solution of *trans*-1,2-dibenzoylethylene in chloroform, liquid bromine was added dropwise at 0 °C. The reaction mixture was stirred at room temperature for 5 h. After completion of the reaction, reaction mixture was poured to aqueous solution of sodium thiosulfate and the compounds from the aqueous layer was extracted with dichloromethane (3 x 10 mL). Combined organic layers were washed with brine solution and dried over anhydrous sodium sulfate, filtered and concentrated under reduced pressure. Triethylamine was added to the resulting reaction mixture in acetone and heated at 50 °C for 2h. After 2 h, solvent was removed under reduced pressure. To the crude reaction mixture, DI water (20 mL) was added and the compounds from the aqueous layer was extracted with dichloromethane (3 x 10 mL). Combined organic layers were washed with brine solution and dried over anhydrous sodium sulfate, filtered and concentrated under reduced pressure. The crude product was purified by chromatography (Combiflash) using ethyl acetate/hexanes mixture as mobile phase.

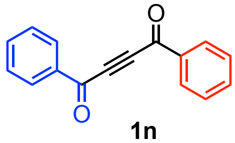

1n

R_f = 0.3 (90% hexanes:10% ethyl acetate). Yield = 69%.

^{1}H NMR (500 MHz, $CDCl_3$) δ 8.22 – 8.20 (m, 2H), 7.73 – 7.70 (m, 1H), 7.57 (t, J = 7.6 Hz, 2H).

^{13}C NMR (126 MHz, $CDCl_3$) δ 176.60, 135.80, 135.23, 129.84, 129.01, 85.88.

4.4. Synthesis of *o*-(aryl)aniline **2A**-**2F**.

Procedure A:

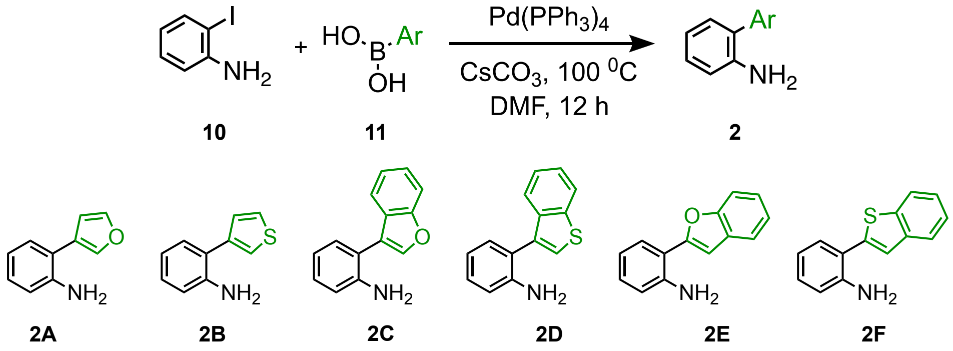

Scheme S4: Synthesis of *o*-(aryl)aniline **2A**-**F**.

o-(aryl)aniline **2** was synthesized by following a literature reported.[5] A mixture of 2-iodoaniline (1 equiv.), arylboronic acid (1.2 equiv.) and cesium carbonate (2 equiv.) in ~5 mL dimethylformamide (DMF) was allowed to stir at room temperature under nitrogen atmosphere for 5 minutes. Tetrakis(triphenylphosphine)palladium (0.2 equiv.) was then added to the mixture, and the resulting reaction mixture was refluxed at 100 ^{0}C for 12 h. Completion of the reaction was monitored by TLC. After the completion of reaction, reaction mixture was cooled to room temperature. Solvent was removed under reduced pressure. To the crude reaction mixture, DI water (20 mL) was added and the compounds from the aqueous layer was extracted with ethyl acetate (3 x 10 mL). Combined organic layers were washed with brine solution and dried over anhydrous sodium sulfate, filtered and concentrated under reduced pressure. The crude product was purified by chromatography (Combiflash) using ethyl acetate/hexanes mixture as mobile phase.

Procedure B:

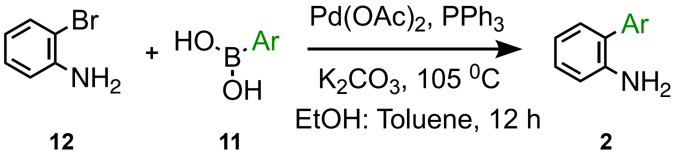

Scheme S5: Synthesis of *o*-(aryl)aniline **2A**-**F**.

Alternatively, aniline **2** was also synthesized following a different literature reported procedure.[6] A mixture of 2-bromoaniline (1 equiv.), arylboronic acid (1.5 equiv.), $Pd(OAc)_2$ (0.03 equiv.), triphenyl phosphine (0.12 equiv.) and potassium carbonate (2.5 equiv.) in ethanol: toluene (5:1) is refluxed under nitrogen atmosphere for 12 h. Completion of the reaction was monitored by TLC. After the completion of reaction, reaction mixture was cooled to room temperature. Solvent was removed under

reduced pressure. To the crude reaction mixture, DI water (20 mL) was added and the compounds from the aqueous layer was extracted with ethyl acetate (3 x 10 mL). Combined organic layers were washed with brine solution and dried over anhydrous sodium sulfate, filtered and concentrated under reduced pressure. The crude product was purified by chromatography (Combiflash) using ethyl acetate/hexanes mixture as mobile phase.

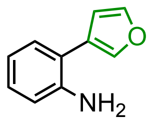

2A

R_f = 0.3 (90% hexanes:10% ethyl acetate). Yield = 51%.

^{1}H NMR (500 MHz, $CDCl_3$) δ 7.66 (dd, *J* = 1.4, 0.9 Hz, 1H), 7.52 (t, *J* = 1.7 Hz, 1H), 7.21 (dd, *J* = 7.6, 1.5 Hz, 1H), 7.16 – 7.10 (m, 1H), 6.85 – 6.76 (m, 2H), 6.64 (dd, *J* = 1.8, 0.9 Hz, 1H), 4.09 (s, 2H).

^{13}C NMR (126 MHz, $CDCl_3$) δ 143.42, 139.95, 129.91, 128.50, 123.48, 119.10, 116.07, 110.98.

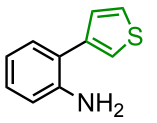

2B

R_f = 0.3 (90% hexanes:10% ethyl acetate). Yield = 96% (Procedure B).

^{1}H NMR (500 MHz, $CDCl_3$) δ 7.43 (dd, *J* = 4.8, 3.0 Hz, 1H), 7.38 (d, *J* = 2.7 Hz, 1H), 7.27 (d, *J* = 4.9 Hz, 1H), 7.21 (d, *J* = 7.5 Hz, 1H), 7.15 (t, *J* = 8 Hz, 1H), 6.81 (t, *J* = 7.5 Hz, 1H), 6.78 (d, *J* = 8.0 Hz, 1H), 3.89 (s, 2H).

^{13}C NMR (126 MHz, $CDCl_3$) δ 143.83, 139.80, 130.22, 128.51, 128.40, 126.03, 122.54, 118.63, 115.74.

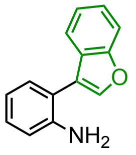

2C

R_f = 0.3 (90% hexanes:10% ethyl acetate). Yield = 60%.

^{1}H NMR (500 MHz, $CDCl_3$) δ 7.80 (s, 1H), 7.65 – 7.57 (m, 2H), 7.40 – 7.35 (m, 1H), 7.34 (dd, *J* = 7.5, 1.3 Hz, 1H), 7.32 – 7.29 (m, 1H), 7.28 – 7.24 (m, 1H), 6.91 (ddd, *J* = 8.3, 7.7, 1.4 Hz, 2H), 4.22 (s, 2H).

^{13}C NMR (126 MHz, $CDCl_3$) δ 155.40, 144.03, 142.61, 130.88, 129.05, 127.00, 124.74, 122.89, 121.00, 118.98, 118.93, 117.09, 115.99, 111.75.

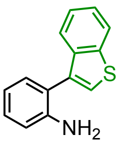

2D

R_f = 0.3 (90% hexanes:10% ethyl acetate). Yield = 53%.

^{1}H NMR (500 MHz, $CDCl_3$) δ 7.98 – 7.92 (m, 1H), 7.68 – 7.63 (m, 1H), 7.47 (s, 1H), 7.44 – 7.35 (m, 2H), 7.26 (ddd, *J* = 7.4, 3.5, 0.8 Hz, 2H), 6.97 – 6.82 (m, 2H), 3.93 (s, 2H).

^{13}C NMR (126 MHz, $CDCl_3$) δ 144.29, 140.35, 138.21, 134.79, 131.17, 129.10, 124.80, 124.58, 124.30, 123.41, 122.83, 121.22, 118.59, 115.76.

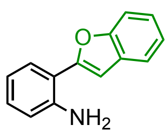

2E

R_f = 0.3 (90% hexanes:10% ethyl acetate). Yield = 27%.

^{1}H NMR (500 MHz, $CDCl_3$) δ 7.67 (dd, *J* = 7.8, 1.3 Hz, 1H), 7.63 – 7.59 (m, 1H), 7.55 (dd, *J* = 7.9, 1.0 Hz, 1H), 7.30 (dd, *J* = 7.3, 1.5 Hz, 1H), 7.28 – 7.25 (m, 1H), 7.22 (ddd, *J* = 8.1, 7.3, 1.6 Hz, 1H), 6.99 (d, *J* = 0.9 Hz, 1H), 6.94 – 6.85 (m, 2H), 4.96 (s, 2H).

^{13}C NMR (126 MHz, $CDCl_3$) δ 155.43, 154.34, 143.31, 129.89, 129.05, 128.86, 128.61, 124.07, 123.03, 120.74, 119.09, 117.34, 116.00, 111.10, 103.17.

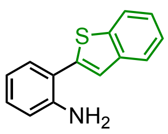

2F

R_f = 0.3 (90% hexanes:10% ethyl acetate). Yield = 30%.

^{1}H NMR (500 MHz, $CDCl_3$) δ 7.89 – 7.85 (m, 1H), 7.81 – 7.76 (m, 1H), 7.47 (d, *J* = 0.5 Hz, 1H), 7.41 (dd, *J* = 7.7, 1.4 Hz, 1H), 7.38 – 7.35 (m, 2H), 7.22 (ddd, *J* = 8.0, 7.4, 1.6 Hz, 1H), 6.96 – 6.84 (m, 2H), 4.91 (s, 2H).

^{13}C NMR (126 MHz, $CDCl_3$) δ 142.90, 141.12, 140.36, 139.87, 131.16, 129.56, 124.47, 124.27, 123.50, 122.75, 122.14, 120.65, 119.53, 116.78.

4.5. Synthesis of *o*-(N-methyl indole)aniline **2G**.

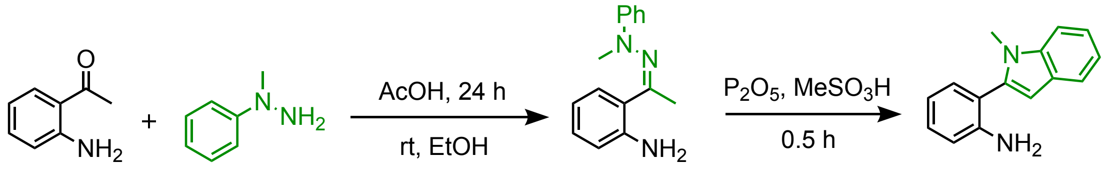

Scheme S6: Synthesis of *o*-(N-methyl indole)aniline **2G**.

o-(N-methyl indole)aniline was synthesized following literature reported procedure.[4] A mixture of 2'aminoacetophenone, N-methyl hydrazine and catalytic amount of acetic acid in ethanol is allowed to stir at room temperature for 24 h (acetic acid is added and reaction is allowed to stir for longer time for complete consumption of starting material). Completion of the reaction was monitored by TLC. After the completion of reaction, solvent was removed under reduced pressure. To the crude reaction mixture, DI water (20 mL) was added and the compounds from the aqueous layer was extracted with ethyl acetate (3 x 10 mL). Combined organic layers were washed with brine solution and dried over anhydrous sodium sulfate, filtered and concentrated under reduced pressure. The crude product **15** was purified by chromatography (Combiflash) using ethyl acetate/hexanes mixture as mobile phase.

A mixture of phosphorous pentoxide and methane sulphonic acid is allowed to stir at 100 °C for 10 minutes. Compound **15** is added slowly to this reaction mixture and allowed to reflux at 100 °C for 30 mins. After completion of the reaction, the reaction mixture is poured to crushed ice containing sodium hydroxide. The product can be either purified by filtration with water or chromatography (Combiflash) using ethyl acetate/hexanes mixture as mobile phase.

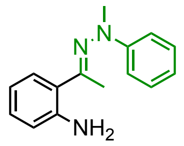

15

R_f = 0.4 (90% hexanes:10% ethyl acetate). Yield = 69%.

^{1}H NMR (500 MHz, $CDCl_3$) δ 7.55 (dd, *J* = 8, 1.5 Hz, 1H), 7.31 (dd, *J* = 8, 1 Hz, 2H), 7.21 – 7.17 (m, 1H), 7.01 (d, *J* = 7.5 Hz, 2H), 6.94 (t, *J* = 7.5 Hz, 1H), 6.79 – 6.71 (m, 2H), 6.38 (s, 2H), 3.18 (s, 3H), 2.46 (s, 3H).

^{13}C NMR (126 MHz, $CDCl_3$) δ 170.62, 151.16, 148.01, 130.44, 129.97, 129.25, 128.95, 119.94, 118.54, 116.81, 116.17, 115.10, 112.60, 42.69, 16.74.

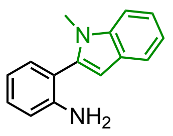

2G

R_f = 0.3 (90% hexanes:10% ethyl acetate). Yield = 48%.

^{1}H NMR (500 MHz, $CDCl_3$) δ 7.68 (d, *J* = 8 Hz, 1H), 7.40 (d, *J* = 8 Hz, 1H), 7.32 – 7.24 (m, 2H), 7.23 – 7.17 (m, 2H), 6.87 (td, *J* = 7.0, 1.0 Hz, 1H), 6.82 (dd, *J* = 8.0, 1.0 Hz, 1H), 6.58 (s, 1H), 3.63 (s, 3H).

^{13}C NMR (126 MHz, $CDCl_3$) δ 145.65, 138.08, 137.78, 131.66, 129.86, 128.13, 121.59, 120.49, 119.78, 118.06, 117.89, 115.29, 109.64, 101.83, 60.46, 30.51.

4.6. General procedure for the synthesis of enaminones **3**.

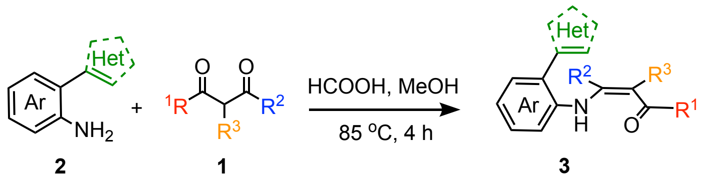

Scheme S7: Synthesis of enaminones.

Enaminones (except **3Ha**) were synthesized following the literature reported procedure.[7] A mixture of *o*-aryl aniline (1 equiv.), 1,3-diketones (1 equiv.) and formic acid (10 µL) was refluxed at 85 °C for 4 hours in methanol (15 mL). Completion of the reaction was monitored by TLC. After the completion of reaction, reaction mixture was cooled to room temperature. Solvent was removed under reduced pressure. To the crude reaction mixture, DI water (20 mL) was added and the compounds from the aqueous layer was extracted with ethyl acetate (3 x 10 mL). Combined organic layers were washed with brine solution and dried over anhydrous sodium sulfate, filtered and concentrated under reduced pressure. The crude product was purified by chromatography (Combiflash) using ethyl acetate/hexanes mixture as mobile phase.

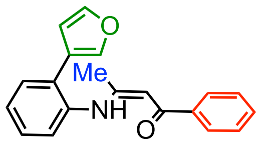

3Aa

R_f = 0.5 (90% hexanes: 10% ethyl acetate). Yield = 81%.

^{1}H NMR (500 MHz, CDCl$_3$) δ 12.90 (s, 1H), 7.96 – 7.92 (m, 2H), 7.70 (dd, *J* = 1.4, 1.0 Hz, 1H), 7.57 – 7.53 (m, 1H), 7.50 – 7.41 (m, 4H), 7.35 (td, *J* = 7.5, 1.6 Hz, 1H), 7.31 (td, *J* = 7.5, 1.7 Hz, 1H), 7.27 – 7.23 (m, 2H), 6.67 (dd, *J* = 2, 1 Hz, 1H), 1.85 (s, 3H).

^{13}C NMR (126 MHz, CDCl$_3$) δ 188.98, 163.66, 143.31, 140.67, 140.04, 135.61, 131.09, 130.36, 128.98, 128.65, 128.43, 127.79, 127.68, 127.28, 122.85, 110.02, 93.61, 20.13.

HRMS-ESI (m/z) ([M + H]$^+$): Calculated: 340.1338; Observed: 340.1340; |Δm| = 0.66 ppm

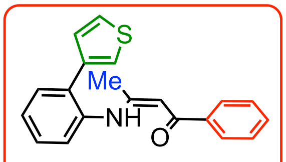

3Ba

R_f = 0.5 (90% hexanes: 10% ethyl acetate). Yield = 84%.

^{1}H NMR (500 MHz, CDCl$_3$) δ 12.96 (s, 1H), 7.95 – 7.90 (m, 2H), 7.59 – 7.53 (m, 1H), 7.49 – 7.41 (m, 4H), 7.38 – 7.30 (m, 3H), 7.29 – 7.25 (m, 2H), 5.85 (s, 1H), 1.80 (s, 3H).

^{13}C NMR (126 MHz, CDCl$_3$) δ 188.64, 163.39, 140.02, 138.69, 135.75, 133.61, 130.86, 130.19, 128.26, 128.08, 128.03, 127.89, 127.4, 127.10, 125.75, 123.50, 93.59, 19.98.

HRMS-ESI (m/z) ([M + H]$^+$): Calculated: 320.1109; Observed: 320.1124; |Δm| = 4.7 ppm

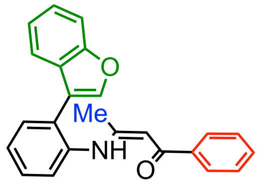

3Ca

R_f = 0.5 (90% hexanes: 10% ethyl acetate). Yield = 86%.

^{1}H NMR (500 MHz, CDCl3) δ 13.01 (s, 1H), 7.85 (dd, J = 8.2, 1.4 Hz, 2H), 7.81 (s, 1H), 7.74 – 7.71 (m, 1H), 7.68 (d, J = 7.9 Hz, 1H), 7.52 (d, J = 8.1 Hz, 1H), 7.46 – 7.39 (m, 5H), 7.37 – 7.30 (m, 2H), 7.28 (dd, J = 7.7, 1.0 Hz, 1H), 7.25 (m, 1H), 1.86 (s, 3H).

^{13}C NMR (126 MHz, $CDCl_3$) δ 188.84, 155.22, 143.40, 139.83, 136.35, 130.96, 130.35, 128.96, 128.64, 128.26, 128.11, 127.94, 127.30, 127.12, 127.02, 126.67, 124.62, 123.06, 120.18, 117.52, 111.81, 20.11.

HRMS-ESI (m/z) ([M + H]$^+$): Calculated: 354.1494; Observed: 354.1491; |Δm| = 0.85 ppm

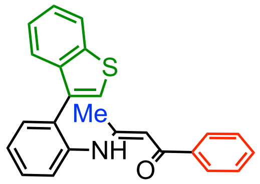

3Da

R_f = 0.5 (90% hexanes: 10% ethyl acetate). Yield = 80%.

^{1}H NMR (500 MHz, $CDCl_3$) δ 12.88 (s, 1H), 7.90 (ddd, *J* = 4.1, 2.7, 0.6 Hz, 1H), 7.81 – 7.77 (m, 2H), 7.67 (ddd, *J* = 5.0, 2.7, 0.6 Hz, 1H), 7.57 (dd, *J* = 7.5, 1.7 Hz, 1H), 7.50 (s, 1H), 7.48 – 7.44 (m, 1H), 7.44 – 7.36 (m, *J* = 9.3, 5.3, 4.8, 2.3 Hz, 7H), 5.69 (s, 1H), 1.61 (s, 3H).

^{13}C NMR (126 MHz, $CDCl_3$) δ 188.56, 162.58, 140.20, 140.04, 138.33, 137.19, 133.02, 132.23, 131.27, 130.74, 128.39, 128.18, 127.43, 127.00, 126.67, 125.92, 124.39, 124.36, 122.84, 122.52, 94.06, 20.02.

HRMS-ESI (m/z) ([M + H]$^+$): Calculated: 370.1266; Observed: 370.1257; |Δm| = 2.43 ppm

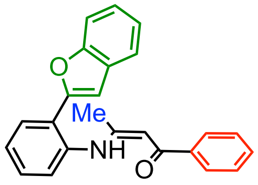

3Ea

R_f = 0.5 (90% hexanes: 10% ethyl acetate). Yield = 71%.

^{1}H NMR (500 MHz, $CDCl_3$) δ 13.10 (s, 1H), 8.15 (d, *J* = 7.6 Hz, 1H), 8.01 (d, *J* = 6.6 Hz, 2H), 7.57 – 7.45 (m, 6H), 7.45 – 7.38 (m, 2H), 7.22 (dd, *J* = 14.1, 6.7 Hz, 2H), 7.12 (s, 1H), 1.93 (s, 3H).

^{13}C NMR (126 MHz, $CDCl_3$) δ 189.09, 163.28, 154.27, 151.94, 139.92, 135.22, 132.32, 131.07, 129.88, 129.27, 128.91, 128.79, 128.64, 128.37, 127.99, 127.77, 127.70, 127.21, 127.02, 124.83, 124.42, 123.08, 122.92, 121.66, 120.90, 111.46, 111.02, 105.74, 96.73, 94.04, 25.91, 20.02.

HRMS-ESI (m/z) ([M + H]$^+$): Calculated: 354.1494; Observed: 354.1491; |Δm| = 0.85 ppm

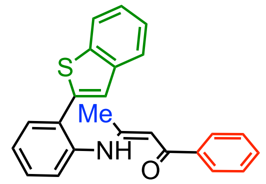

3Fa

R_f = 0.5 (90% hexanes: 10% ethyl acetate). Yield = 84%.

^{1}H NMR (500 MHz, $CDCl_3$) δ 13.00 (s, 1H), 7.93 (d, J = 7.4 Hz, 2H), 7.82 – 7.70 (m, 3H), 7.54 (s, 1H), 7.42 (dd, J = 21.3, 5.5 Hz, 6H), 7.30 (d, J = 1.5 Hz, 3H), 1.85 (s, 3H).

^{13}C NMR (126 MHz, $CDCl_3$) δ 193.82, 163.41, 136.79, 135.82, 132.31, 131.41, 130.96, 130.35, 129.61, 128.85, 128.77, 128.64, 128.29, 127.72, 127.18, 127.02, 124.38, 123.90, 123.67, 123.20, 122.12, 96.73, 25.91, 20.10.

HRMS-ESI (m/z) ([M + H]$^+$): Calculated: 370.1266; Observed: 370.1264; |Δm| = 0.54 ppm

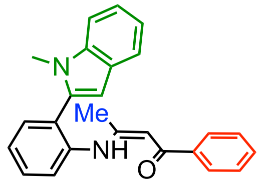

3Ga

R_f = 0.5 (90% hexanes: 10% ethyl acetate). Yield = 23%.

^{1}H NMR (500 MHz, $CDCl_3$) δ 12.98 (s, 1H), 7.83 – 7.76 (m, 2H), 7.65 (d, *J* = 7.8 Hz, 1H), 7.55 – 7.46 (m, 2H), 7.45 – 7.31 (m, 6H), 7.27 – 7.19 (m, 2H), 7.14 (t, *J* = 7.4 Hz, 1H), 6.58 (s, 1H), 3.65 (s, 3H), 1.86 (s, 3H).

^{13}C NMR (126 MHz, $CDCl_3$) δ 162.52, 139.92, 138.02, 137.76, 132.56, 130.82, 129.91, 129.25, 128.19, 127.96, 127.28, 127.04, 126.78, 121.75, 120.65, 119.71, 109.76, 102.96, 94.17, 30.99, 20.04.

HRMS-ESI (m/z) ([M + H]$^+$): Calculated: 367.1810; Observed: 367.1821; |Δm| = 2.9 ppm

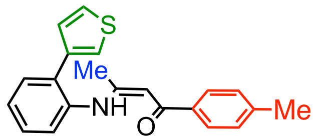

3Bb

R_f = 0.5 (90% hexanes: 10% ethyl acetate). Yield = 94%.

^{1}H NMR (500 MHz, $CDCl_3$) δ 12.90 (s, 1H), 7.82 (d, *J* = 8.1 Hz, 2H), 7.55 (dd, *J* = 4.8, 2.6 Hz, 1H), 7.42 (d, *J* = 1.5 Hz, 1H), 7.33 (ddd, *J* = 8.1, 4.7, 2.8 Hz, 3H), 7.29 – 7.20 (m, 4H), 5.82 (s, 1H), 2.40 (s, 3H), 1.78 (s, 3H).

^{13}C NMR (126 MHz, $CDCl_3$) δ 188.52, 163.00, 141.24, 138.74, 137.31, 135.86, 133.60, 130.17, 128.99, 128.11, 128.06, 127.88, 127.34, 127.17, 125.73, 123.4, 93.48, 21.52, 19.9.

HRMS-ESI (m/z) ([M + H]$^+$): Calculated: 334.1266; Observed: 334.1278; |Δm| = 3.6 ppm

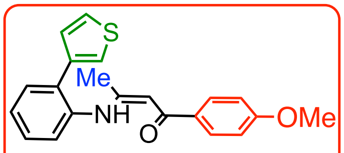

3Bc

R_f = 0.5 (90% hexanes: 10% ethyl acetate). Yield = 55%.

^{1}H NMR (500 MHz, $CDCl_3$) δ 12.85 (s, 1H), 7.92 – 7.87 (m, 2H), 7.57 – 7.51 (m, 1H), 7.41 (dd, *J* = 2.9, 1.3 Hz, 1H), 7.33 (ddd, *J* = 8.1, 4.9, 2.7 Hz, 3H), 7.28 – 7.23 (m, 2H), 6.96 – 6.89 (m, 2H), 5.78 (s, 1H), 3.86 (s, 3H), 1.77 (s, 3H).

^{13}C NMR (126 MHz, $CDCl_3$) δ 187.83, 161.92, 138.75, 135.91, 133.59, 132.66, 130.15, 129.01, 128.12, 128.06, 127.86, 127.29, 125.70, 123.47, 113.46, 55.39, 20.02.

HRMS-ESI (m/z) ([M + H]$^+$): Calculated: 350.1215; Observed: 350.1211; |Δm| = 1.14 ppm

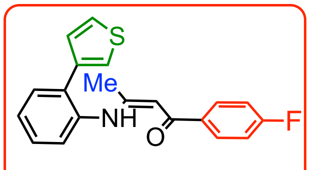

3Bd

R_f = 0.5 (90% hexanes: 10% ethyl acetate). Yield = 74%.

^{1}H NMR (500 MHz, $CDCl_3$) δ 12.87 (s, 1H), 7.94 – 7.86 (m, 2H), 7.59 – 7.50 (m, 1H), 7.40 (dd, J = 3.0, 1.3 Hz, 1H), 7.36 – 7.31 (m, 3H), 7.26 – 7.23 (m, 2H), 7.14 – 7.05 (m, 2H), 5.76 (s, 1H), 1.78 (s, 3H).

^{13}C NMR (126 MHz, $CDCl_3$) δ 187.19, 165.51 and 163.54 ($J_{C\text{-}F}$ = 248.22 Hz), 138.66, 136.23 and 136.21($J_{C\text{-}F}$ = 2.52 Hz), 135.65, 133.60, 130.22, 129.37 and 129.30 ($J_{C\text{-}F}$ = 8.82 Hz), 128.03, 128.02, 127.92, 127.49, 125.78, 123.50, 115.24 and 115.07 ($J_{C\text{-}F}$ = 21.42 Hz), 93.21, 19.99.

HRMS-ESI (m/z) ([M + H]$^+$): Calculated: 338.1015; Observed: 338.1025; |Δm| = 2.95 ppm

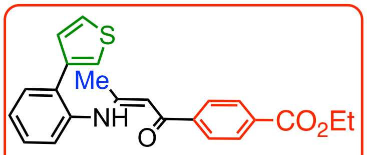

3Be

R_f = 0.5 (90% hexanes: 10% ethyl acetate). Yield = 93%.

^{1}H NMR (500 MHz, $CDCl_3$) δ 13.01 (s, 1H), 8.09 (d, J = 8.3 Hz, 2H), 7.93 (d, J = 8.3 Hz, 2H), 7.58 – 7.53 (m, 1H), 7.40 (dd, J = 2.9, 1.2 Hz, 1H), 7.35 (ddt, J = 8.2, 4.9, 4.2 Hz, 3H), 7.27 – 7.23 (m, 2H), 5.82 (s, 1H), 4.40 (q, J = 7.1 Hz, 2H), 1.80 (s, 3H), 1.41 (t, J = 7.1 Hz, 3H).

^{13}C NMR (126 MHz, $CDCl_3$) δ 187.39, 166.29, 164.25, 143.82, 138.59, 135.48, 133.60, 132.19, 130.27, 129.54, 128.00, 127.97, 127.95, 127.63, 127.00, 125.87, 123.55, 93.93, 61.18, 19.98, 14.35.

HRMS-ESI (m/z) ([M + H]$^+$): Calculated: 392.1320; Observed: 392.1310; |Δm| = 2.5 ppm

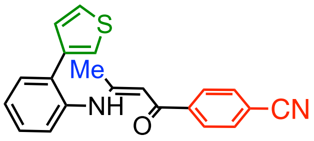

3Bf

R_f = 0.5 (90% hexanes: 10% ethyl acetate). Yield = 66%.

^{1}H NMR (500 MHz, $CDCl_3$) δ 13.04 (s, 1H), 7.99 (d, J = 8.3 Hz, 2H), 7.74 (d, J = 8.2 Hz, 2H), 7.62 – 7.55 (m, 1H), 7.44 – 7.34 (m, 4H), 7.28 – 7.22 (m, 2H), 5.80 (s, 1H), 1.83 (s, 3H).

^{13}C NMR (126 MHz, $CDCl_3$) δ 186.00, 164.96, 143.83, 138.50, 135.23, 133.58, 132.17, 130.34, 128.03, 127.95, 127.85, 127.82, 127.61, 125.93, 123.56, 118.67, 113.95, 93.68, 19.99.

HRMS-ESI (m/z) ([M + H]$^+$): Calculated: 345.1062; Observed: 345.1063; |Δm| = 0.3 ppm

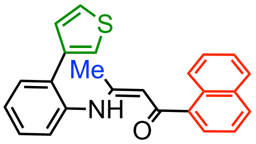

3Bg

R_f = 0.5 (90% hexanes: 10% ethyl acetate). Yield = 93%.

^{1}H NMR (500 MHz, $CDCl_3$) δ 12.94 (s, 1H), 8.43 (d, J = 8.4 Hz, 1H), 7.87 (t, J = 7.7 Hz, 2H), 7.68 – 7.63 (m, 1H), 7.60 – 7.55 (m, 1H), 7.55 – 7.50 (m, 2H), 7.49 – 7.44 (m, 2H), 7.38 (m, 3H), 7.35 – 7.29 (m, 2H), 5.61 (s, 1H), 1.81 (s, 3H).

^{13}C NMR (126 MHz, $CDCl_3$) δ 193.00, 163.25, 140.05, 138.68, 135.74, 133.82, 133.66, 130.26, 129.81, 128.20, 128.02, 127.95, 127.46, 126.52, 126.12, 125.93, 125.75, 125.24, 124.85, 123.64, 98.45, 19.86.

HRMS-ESI (m/z) ([M + H]$^+$): Calculated: 370.1266; Observed: 370.1257; |Δm| = 2.43 ppm

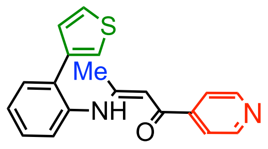

3Bh

R_f = 0.5 (50% hexanes: 50% ethyl acetate). Yield = 62%.

^{1}H NMR (500 MHz, $CDCl_3$) δ 13.03 (s, 1H), 8.71 (d, J = 5.6 Hz, 2H), 7.71 (dd, J = 4.6, 1.4 Hz, 2H), 7.58 – 7.53 (m, 1H), 7.41 – 7.36 (m, 3H), 7.34 (dd, J = 5.0, 2.9 Hz, 1H), 7.27 – 7.24 (m, 1H), 7.22 (dd, J = 5.0, 1.3 Hz, 1H), 5.79 (s, 1H), 1.81 (s, 3H).

^{13}C NMR (126 MHz, $CDCl_3$) δ 185.83, 165.26, 150.09, 146.85, 138.46, 135.16, 133.60, 130.35, 128.04, 127.93, 127.88, 127.83, 125.96, 123.57, 120.94, 93.56, 19.95.

HRMS-ESI (m/z) ([M + H]$^+$): Calculated: 321.1062; Observed: 321.1053; |Δm| = 2.8 ppm

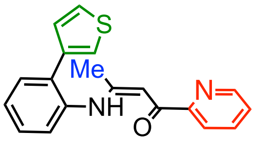

3Bi

R_f = 0.5 (50% hexanes: 50% ethyl acetate). Yield = 88%.

^{1}H NMR (500 MHz, $CDCl_3$) δ 12.94 (s, 1H), 8.64 (dd, J = 4.7, 0.7 Hz, 1H), 8.12 (d, J = 7.9 Hz, 1H), 7.83 (td, J = 7.7, 1.7 Hz, 1H), 7.58 – 7.52 (m, 1H), 7.40 (dd, J = 3.0, 1.3 Hz, 1H), 7.39 – 7.33 (m, 3H), 7.33 – 7.30 (m, 1H), 7.27 – 7.23 (m, 2H), 6.54 (s, 1H), 1.86 (s, 3H).

^{13}C NMR (126 MHz, $CDCl_3$) δ 164.95, 155.99, 148.45, 138.58, 136.98, 135.58, 133.54, 130.24, 127.96, 127.91, 127.55, 125.88, 125.31, 123.53, 121.77, 93.03, 19.99.

HRMS-ESI (m/z) ([M + H]$^+$): Calculated: 321.1062; Observed: 321.1053; |Δm| = 2.8 ppm

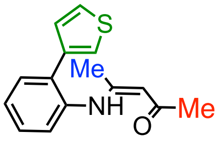

3Bj

R_f = 0.5 (90% hexanes: 10% ethyl acetate). Yield = 48%.

^{1}H NMR (500 MHz, CDCl3) δ 12.31 (s, 1H), 7.53 – 7.48 (m, 1H), 7.38 – 7.36 (m, 1H), 7.36 – 7.33 (m, 1H), 7.32 – 7.29 (m, 2H), 7.23 – 7.17 (m, 2H), 5.12 (s, 1H), 2.06 (s,3H), 1.66 (s, 3H).

^{13}C NMR (126 MHz, $CDCl_3$) δ 196.09, 161.27, 138.70, 135.83, 133.52, 130.16, 128.06, 127.96, 127.82, 127.14, 125.64, 123.44, 97.06, 29.13, 19.42.

HRMS-ESI (m/z) ([M + H]$^+$): Calculated: 258.0952; Observed: 258.09543; |Δm| = 3.48 ppm

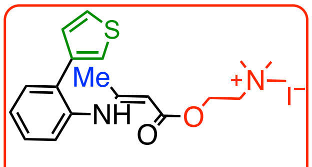

3Bk

R_f = 0.5 (90% ethylacetate: 10% methanol). Yield = 59%.

^{1}H NMR (500 MHz, CD_3OD) δ 10.16 (s, 1H), 7.59 – 7.53 (m, 1H), 7.50 – 7.45 (m, 1H), 7.38 (ddd, *J* = 6.3, 5.5, 3.6 Hz, 1H), 7.30 – 7.26 (m, 1H), 7.24 (dd, *J* = 4.1, 2.2 Hz, 1H), 4.72 (s, 1H), 4.54 (ddd, *J* = 7.4, 5.1, 2.6 Hz, 2H), 3.78 – 3.64 (m, 2H), 3.24 (d, *J* = 4.8 Hz, 9H), 1.78 (d, *J* = 1.6 Hz, 3H).

^{1}H NMR (500 MHz, $CDCl_3$) δ 10.07 (s, 1H), 7.54 – 7.46 (m, 1H), 7.39 (dd, *J* = 5.0, 3.0 Hz, 1H), 7.32 (dd, *J* = 4.8, 3.1 Hz, 3H), 7.22 – 7.14 (m, 2H), 4.59 (s, 1H), 4.53 (s, 2H), 4.08 – 3.94 (m, 2H), 3.62 – 3.43 (m, 9H), 1.75 (s, 3H).

^{13}C NMR (126 MHz, $CDCl_3$) δ 168.50, 162.22, 138.63, 135.63, 133.38, 130.26, 128.04, 127.97, 127.85, 127.17, 126.03, 123.46, 83.53, 65.68, 56.12, 54.83, 20.05.

HRMS-ESI (m/z) ($[M]^+$): Calculated: 345.1637; Observed: 345.1635; |Δm| = 0.5 ppm

4.7. Synthesis of enaminone **3Ha**.

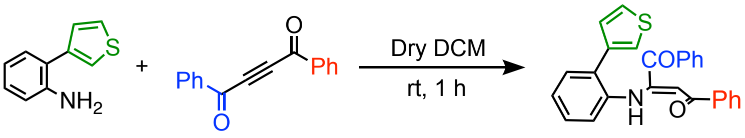

Scheme S8: Synthesis of enaminone **3Ha**.

Enaminone **3Ha** was synthesized following similar literature reported procedure.[8] A solution of 1,3-diketone **1l** (1 equiv.) in dry dichloromethane is added dropwise to a solution of *o*-(2-thiophene) aniline (1 equiv.) in dry dichloromethane. The resulting solution is allowed to stir at room temperature for 1 h. After the completion of reaction, the product can either be purified by recrystallization with ethanol or chromatography (Combiflash) using ethyl acetate/hexanes mixture as mobile phase.

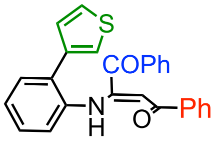

3Ha

R_f = 0.5 (90% hexanes: 10% ethyl acetate). Yield = 57%.

^{1}H NMR (500 MHz, $CDCl_3$) δ 12.36 (s, 1H), 7.91 – 7.86 (m, 2H), 7.83 – 7.77 (m, 2H), 7.53 (t, *J* = 7.4 Hz, 1H), 7.49 (t, *J* = 7.4 Hz, 1H), 7.46 – 7.40 (m, 4H), 7.36 (t, *J* = 7.8 Hz, 2H), 7.31 – 7.27 (m, 2H), 7.12 – 7.06 (m, 2H), 7.04 – 7.00 (m, 1H), 6.10 (s, 1H).

^{13}C NMR (126 MHz, $CDCl_3$) δ 192.15, 190.89, 156.63, 139.07, 138.48, 136.37, 134.89, 134.04, 131.92, 130.63, 130.51, 129.60, 128.58, 128.47, 128.45, 128.03, 127.47, 126.10, 125.97, 124.22, 123.90, 95.95.

HRMS-ESI (m/z) ([M + H]$^+$): Calculated: 410.1215; Observed: 410.1205; |Δm| = 2.4 ppm

5. UV-vis absorption spectrum of enaminones.

5.1. Plot of extinction coefficient *vs* wavelength of enaminones **3B-3Bk**, **3Ha**.

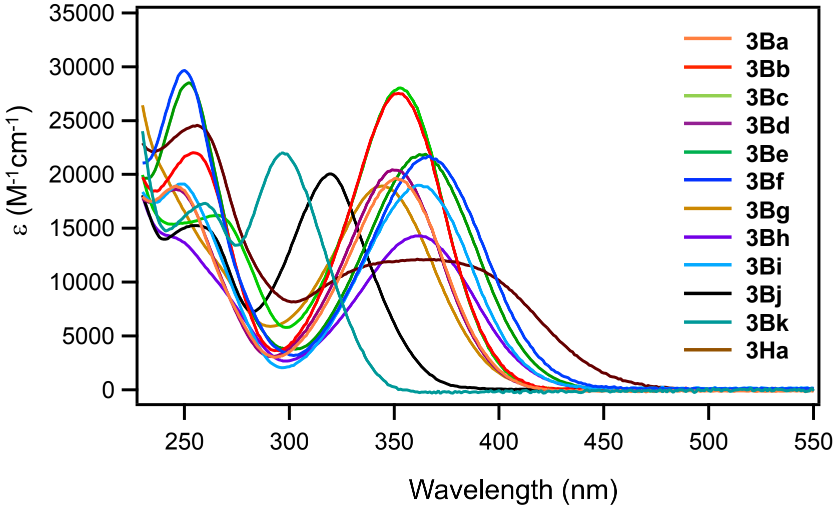

Figure S2: Plot of extinction coefficient *vs* wavelength of enaminones **3Ba-3Bk**, **3Ha**.

Table S1: Extinction coefficient of enaminones at 350 nm, 370 nm and at λ_{max} at long wavelength peak.

Enaminone	ε @350 nm	ε @370 nm	ε @λ_{max}
**3Ba**	19631 $M^{-1}cm^{-1}$	13842 $M^{-1}cm^{-1}$	19656 $M^{-1}cm^{-1}$ @351nm
**3Bb**	27501 $M^{-1}cm^{-1}$	19690 $M^{-1}cm^{-1}$	27545 $M^{-1}cm^{-1}$ @352nm
**3Bc**	27787 $M^{-1}cm^{-1}$	19979 $M^{-1}cm^{-1}$	28034 $M^{-1}cm^{-1}$ @353nm
**3Bd**	20401 $M^{-1}cm^{-1}$	13799 $M^{-1}cm^{-1}$	20443 $M^{-1}cm^{-1}$ @349nm
**3Be**	19323 $M^{-1}cm^{-1}$	20943 $M^{-1}cm^{-1}$	21902 $M^{-1}cm^{-1}$ @365nm
**3Bf**	18236 $M^{-1}cm^{-1}$	21448 $M^{-1}cm^{-1}$	21707 $M^{-1}cm^{-1}$ @367nm
**3Bg**	18436 $M^{-1}cm^{-1}$	10848 $M^{-1}cm^{-1}$	18926 $M^{-1}cm^{-1}$ @345nm
**3Bh**	13147 $M^{-1}cm^{-1}$	13568 $M^{-1}cm^{-1}$	14303 $M^{-1}cm^{-1}$ @362nm
**3Bi**	17021 $M^{-1}cm^{-1}$	18040 $M^{-1}cm^{-1}$	19008 $M^{-1}cm^{-1}$ @362nm
**3Bj**	6184 $M^{-1}cm^{-1}$	1005 $M^{-1}cm^{-1}$	20067 $M^{-1}cm^{-1}$ @319nm
**3Bk**	265 $M^{-1}cm^{-1}$	~0 $M^{-1}cm^{-1}$	22002 $M^{-1}cm^{-1}$ @297nm
**3Ha**	11901 $M^{-1}cm^{-1}$	12038 $M^{-1}cm^{-1}$	12124 $M^{-1}cm^{-1}$ @369nm

5.2. UV-vis absorption spectra of enaminones **3** at reaction concentration.

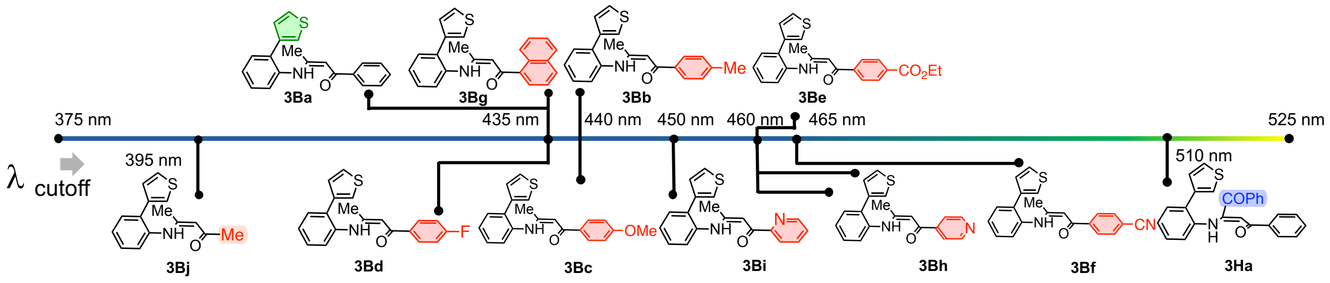

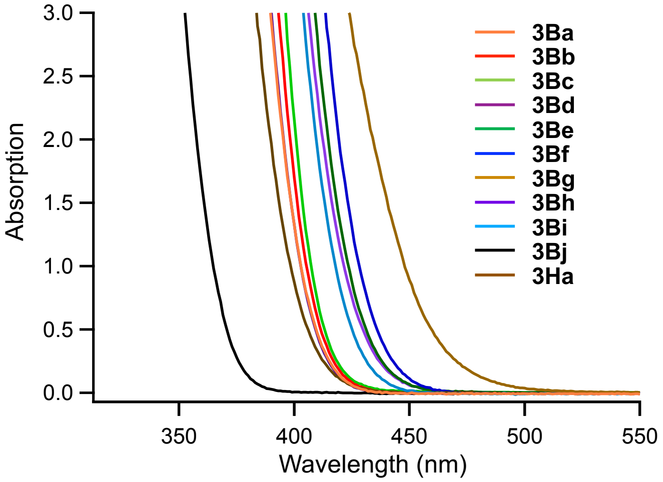

Figure S3: Plot of absorption *vs* wavelength of enaminones **3** at reaction concentration (0.67mM).

5.3. UV-Vis absorption spectra of enaminone **3Bd** in different solvents.

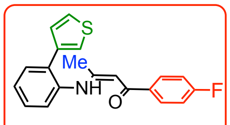

3Bd

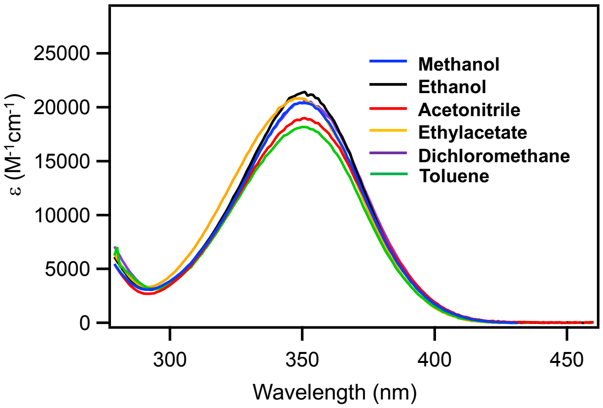

Figure S4: Plot of extinction coefficient *vs* wavelength of enaminone **3Bd** in different solvents.

Table S2: Extinction coefficient of enaminone **3Bd** at 350 nm, 370 nm and at λ_{max} in different solvents.

Solvent	ε @350 nm	ε @370 nm	ε @λ_{max}
Methanol	20401 $M^{-1}cm^{-1}$	13799 $M^{-1}cm^{-1}$	20433 $M^{-1}cm^{-1}$ @349nm
Ethanol	21367 $M^{-1}cm^{-1}$	14325 $M^{-1}cm^{-1}$	21442 $M^{-1}cm^{-1}$ @351nm
Acetonitrile	18970 $M^{-1}cm^{-1}$	13316 $M^{-1}cm^{-1}$	19003 $M^{-1}cm^{-1}$ @351nm
Ethyl acetate	20811 $M^{-1}cm^{-1}$	13260 $M^{-1}cm^{-1}$	20836 $M^{-1}cm^{-1}$ @347nm
Dichloromethane	20361 $M^{-1}cm^{-1}$	14454 $M^{-1}cm^{-1}$	20703 $M^{-1}cm^{-1}$ @351nm
Toluene	18172 $M^{-1}cm^{-1}$	12392 $M^{-1}cm^{-1}$	18176 $M^{-1}cm^{-1}$ @351nm

6. Irradiation procedure.

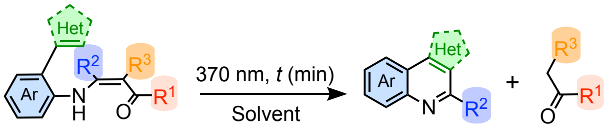

3 4 5

Scheme S9: Photoreaction of enaminones.

A solution of enaminone **3** (0.67 mM) in ~9-15 mL of methanol is taken a Pyrex tube and sealed. The solution is degassed with N_2 for 20 mins. The resulting solution is irradiated at 370 nm Kessil LED (Pyrex tube placed in-between 2 Kessil LEDs placed at a distance of ~12 cm, 100% power) for 25 min with stirring.

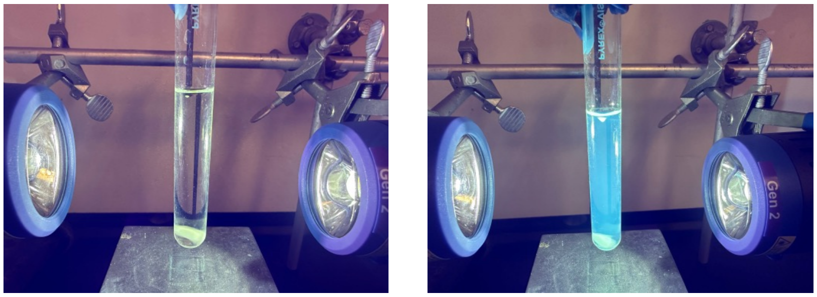

0 min under 370 nm Kessil LED   25 min under 370 nm Kessil LED

Figure S5: Reaction mixture before and after irradiation under 370 nm Kessil LED. Development of fluorescence is observed during the course of the reaction which shows to the formation of quinoline photoproduct.

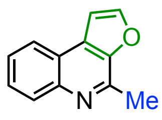

4A

^{1}H NMR (500 MHz, $CDCl_3$) δ 8.15 (d, *J* = 8.4 Hz, 1H), 8.08 (dd, *J* = 8.1, 0.9 Hz, 1H), 7.87 (d, *J* = 2.0 Hz, 1H), 7.67 (ddd, *J* = 8.4, 7.0, 1.5 Hz, 1H), 7.62 – 7.54 (m, 1H), 7.26 (s, *J* = 2.0 Hz, 1H), 2.93 (s, 3H).

^{13}C NMR (126 MHz, $CDCl_3$) δ 148.43, 146.90, 146.00, 143.99, 129.07, 129.02, 127.51, 125.95, 123.40, 122.81, 105.66, 19.56.

HRMS-ESI (m/z) ([M + H]$^+$): Calculated: 184.0762; Observed: 184.0762; |Δm| = 0 ppm

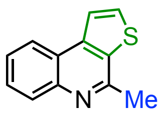

4B

^{1}H NMR (500 MHz, $CDCl_3$) δ 8.23 (d, *J* = 8.0 Hz, 1H), 8.16 (d, *J* = 8.3 Hz, 1H), 7.97 (d, *J* = 5.3 Hz, 1H), 7.80 (d, *J* = 5.3 Hz, 1H), 7.73 – 7.66 (m, 1H), 7.59 (t, *J* = 7.2 Hz, 1H), 2.94 (s, 3H).

^{13}C NMR (126 MHz, $CDCl_3$) δ 153.19, 144.87, 141.63, 133.40, 130.70, 129.10, 128.05, 126.03, 123.42, 123.26, 122.16, 24.20.

HRMS-ESI (m/z) ([M + H]$^+$): Calculated: 200.0534; Observed: 200.0530; |Δm| = 1.9 ppm

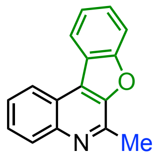

4C

^{1}H NMR (500 MHz, $CDCl_3$) δ 8.54 (t, *J* = 6.6 Hz, 1H), 8.43 (t, *J* = 6.9 Hz, 1H), 8.26 (d, *J* = 7.8 Hz, 1H), 7.80 (dd, *J* = 8.3, 3.4 Hz, 1H), 7.78 – 7.70 (m, 2H), 7.69 – 7.64 (m, 1H), 7.55 (td, *J* = 7.7, 3.4 Hz, 1H), 3.05 (s, 3H).

^{13}C NMR (126 MHz, $CDCl_3$) δ 156.18, 148.97, 146.81, 129.54, 128.55, 127.47, 126.69, 123.86, 123.61, 123.56, 123.36, 123.20, 112.77, 29.74.

HRMS-ESI (m/z) ($[M + H]^+$): Calculated: 234.0919; Observed: 234.0930; |Δm| = 4.7 ppm

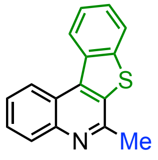

4D

^{1}H NMR (500 MHz, $CDCl_3$) δ 8.88 – 8.77 (m, 2H), 8.26 (d, *J* = 8.2 Hz, 1H), 8.03 (dd, *J* = 7.4, 1.5 Hz, 1H), 7.78 – 7.73 (m, 1H), 7.72 – 7.67 (m, 1H), 7.66 – 7.59 (m, 2H), 2.98 (s, 3H).

^{13}C NMR (126 MHz, $CDCl_3$) δ 153.44, 145.64, 140.69, 135.75, 135.39, 133.58, 129.90, 127.72, 127.33, 126.59, 125.87, 125.35, 124.31, 123.57, 122.66, 24.26.

HRMS-ESI (m/z) ($[M + H]^+$): Calculated: 250.0690; Observed: 250.0697; |Δm| = 2.7 ppm

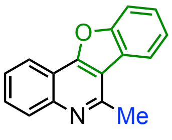

4E

^{1}H NMR (500 MHz, $CDCl_3$) δ 8.39 (d, *J* = 7.9 Hz, 1H), 8.21 (d, *J* = 8.4 Hz, 1H), 8.10 (d, *J* = 7.1 Hz, 1H), 7.78 (dd, *J* = 7.6, 4.2 Hz, 2H), 7.66 (t, *J* = 7.5 Hz, 1H), 7.57 (t, *J* = 7.6 Hz, 1H), 7.51 (t, *J* = 7.4 Hz, 1H), 3.17 (s, 3H).

^{13}C NMR (126 MHz, $CDCl_3$) δ 157.45, 155.86, 154.81, 146.77, 129.45, 128.81, 126.82, 126.17, 124.04, 123.41, 121.82, 120.72, 116.17, 115.45, 112.11, 24.23.

HRMS-ESI (m/z) ($[M + H]^+$): Calculated: 234.0919; Observed: 234.0916; |Δm| = 1.28 ppm

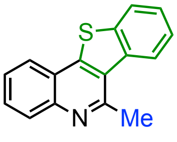

4F

^{1}H NMR (500 MHz, $CDCl_3$) δ 8.46 (d, *J* = 8.0 Hz, 1H), 8.19 (d, *J* = 8.3 Hz, 1H), 8.10 (dd, *J* = 8.1, 0.9 Hz, 1H), 8.05 – 7.99 (m, 1H), 7.76 (ddd, *J* = 8.3, 7.0, 1.3 Hz, 1H), 7.61 (td, *J* = 8.0, 1.0 Hz, 2H), 7.58 – 7.52 (m, 1H), 3.27 (s, 3H).

^{13}C NMR (126 MHz, $CDCl_3$) δ 154.74, 146.80, 144.15, 138.97, 135.88, 129.45, 129.15, 127.95, 126.38, 126.21, 125.35, 124.64, 123.79, 123.17, 123.08, 26.55.

HRMS-ESI (m/z) ($[M + H]^+$): Calculated: 250.0690; Observed: 250.0697; |Δm| = 2.7 ppm

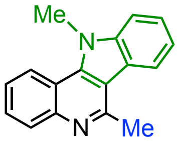

4G

^{1}H NMR (600 MHz, $CDCl_3$) δ 8.60 (d, J = 8.2 Hz, 1H), 8.29 (d, J = 7.4 Hz, 1H), 8.25 (d, J = 7.9 Hz, 1H), 7.75 – 7.69 (m, 1H), 7.64 – 7.56 (m, 3H), 7.44 (m, 1H), 4.38 (s, 3H), 3.21 (s, 3H).

^{13}C NMR (151 MHz, $CDCl_3$) δ 154.56, 140.56, 140.17, 129.01, 128.05, 125.33, 125.01, 122.23, 121.98, 121.76, 121.31, 120.78, 117.23, 113.89, 109.41, 33.57, 29.72.

HRMS-ESI (m/z) ($[M + H]^+$): Calculated: 247.1235; Observed: 247.1246; |Δm| = 4.45 ppm

7. Solvent optimization for the photoreaction of enaminone

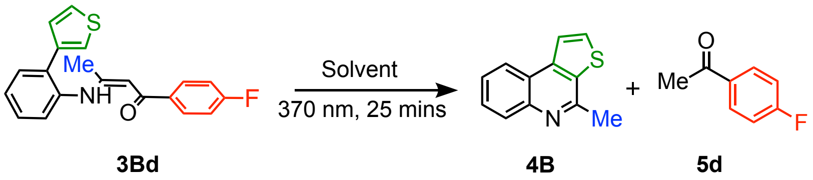

Scheme S10: Optimization reaction of enaminone **3Bd** in different solvents.

A solution of enaminone **3Bd** (0.67 mM) in ~11 mL of solvent is taken a Pyrex tube and sealed. The solution is degassed with N_2 for 20 mins. The resulting solution irradiated at 370 nm. After the completion of reaction, a known amount of triphenyl methane (internal standard) is added to the reaction mixture and solvent was evaporated under reduced pressure. ^1H NMR is recorded in $CDCl_3$ for determining the NMR yield. Proton resonance at ~5.56 ppm (internal standard) is used as a reference to monitor the formation of photoproduct.

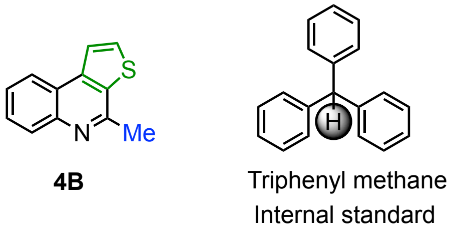

Table S3: Solvent optimization for photoreaction of enaminone **3Bd**.

Solvent	Yield
Methanol	85%
Ethanol	75%
Acetonitrile	61%
Ethyl acetate	50%
Dichloromethane	64%
Toluene	40%

8. Control studies of enaminone 3Bg under dark and thermal conditions

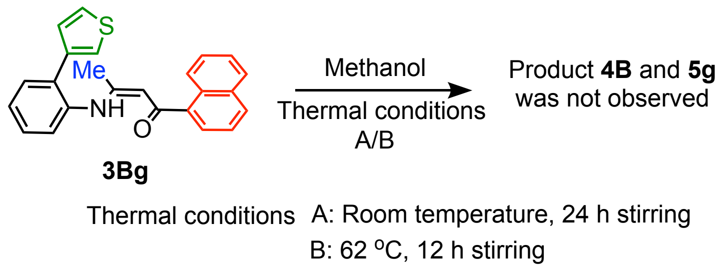

Scheme S11: Control reaction of enaminone **3Bg** under different conditions.

A solution of enaminone **3** in ~10 mL of methanol is taken a Pyrex tube and sealed. The solution is degassed with N_2 for 20 mins. The resulting solution is stirred at room temperature for 24 h (condition A) or heated at 62 °C for 12 h (condition B). After 24 h/ 12 h, the solvent was removed under reduced pressure and analyzed by ^{1}H NMR spectroscopy.

^{1}H NMR spectroscopy did not show the formation of photoproduct.

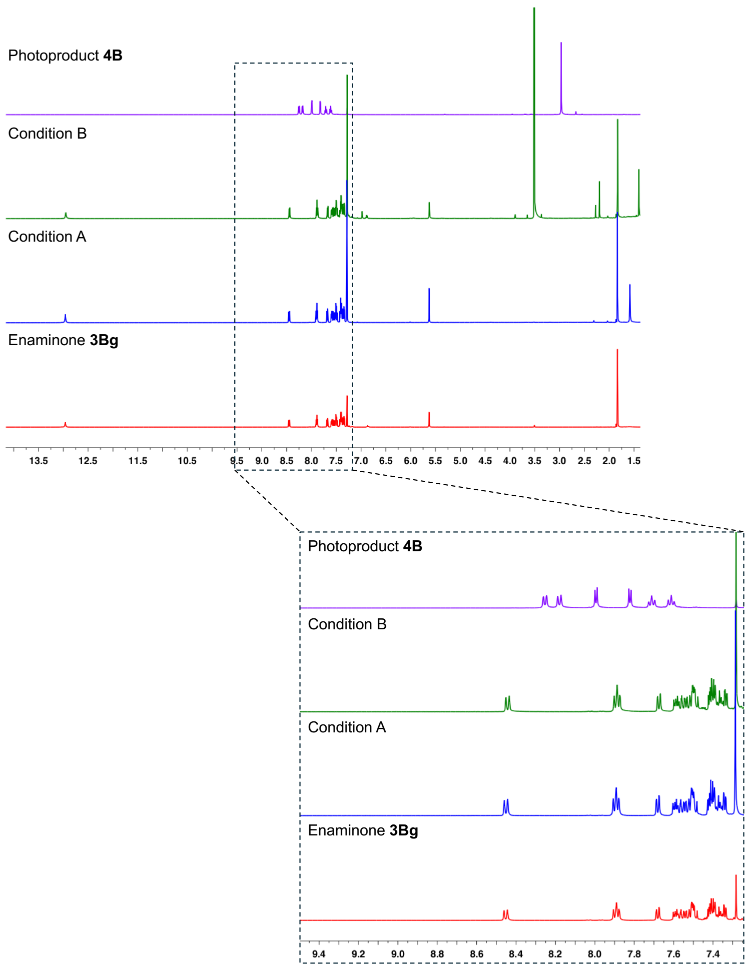

Figure S6: ^{1}H NMR overlayed spectrum of enaminone **3Bg**, control reaction condition A, condition B and photoproduct **4B**.

9. Photoreaction of enaminones at different conditions.

Table S4: Irradiation of enaminones **3** at various conditions.

SL.No	Enaminone	Irradiation source	Concentration	Solvent	Irradiation time	Yield of **4**
1.	**3Ba**	370 nm	0.78 mM	Methanol	25 min	80%
2.	**3Bd**	370 nm	0.74 mM	Methanol	25 min	95%
3.	**3Bd**	370 nm	0.67 mM	Methanol	25 min	85%
4.	**3Bd** (-40 °C)	370 nm	0.67 mM	Methanol	25 min	57%
5.	**3Bd**	390 nm	0.67 mM	Methanol	1 h	63%
6.	**3Bc**	370 nm	0.72 mM	Methanol	25 min	91%
7.	**3Be**	370 nm	0.64 mM	Methanol	25 min	20%
8.	**3Be**	370 nm	0.64 mM	Methanol	3.5 h	53%
9.	**3Bg**	370 nm	0.67 mM	Methanol	25 min	77%
10.	**3Bg** (Air)	370 nm	0.67 mM	Methanol	25 min	75%
11.	**3Bg** (O_2)	370 nm	0.67 mM	Methanol	25 min	61%
12.	**3Bh**	370 nm	0.78 mM	Methanol	25 min	23%
13.	**3Bj**	370 nm	0.97 mM	Methanol	25 min	83%
14.	**3Bj**	370 nm	0.67 mM	Methanol	25 min	63%
15.	**3Bk**	350 nm	0.75mM	Methanol	24 h	19%*

*Isolated yield

10. Determination of fluorescence quantum yield

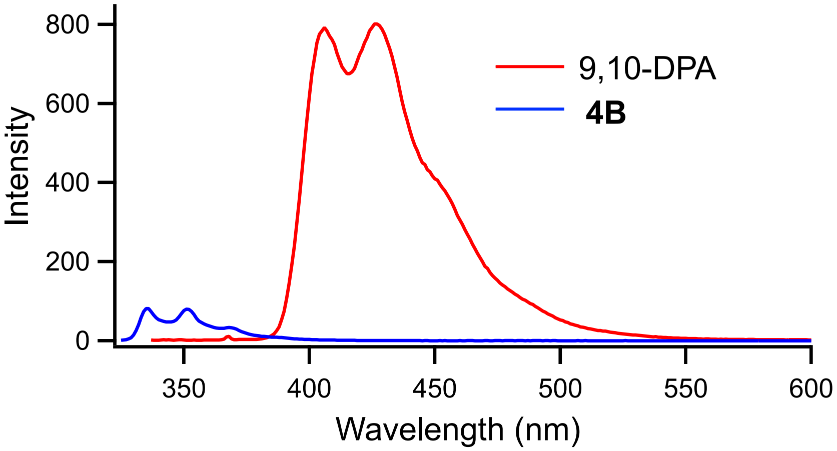

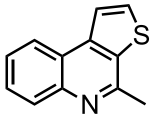

4B

Solvent : MeOH

$\Phi_f = 0.037$

Figure S7: Fluorescence quantum yield determination: Fluorescence spectra of quinoline photoproduct **4B** (blue trace), in methanol compared with the fluorescence quantum yield of standard, 9,10-diphenylanthracene (9,10-DPA) (red trace) recorded in ethanol at λ_{exc} = 332 nm. Fluorescence quantum yields were calculated using Φ_f of standard, 0.95 and correcting for the refractive index of the solvents.[9-10]

11. Monitoring the photolysis reaction of enaminone by ^{1}H NMR spectroscopy.

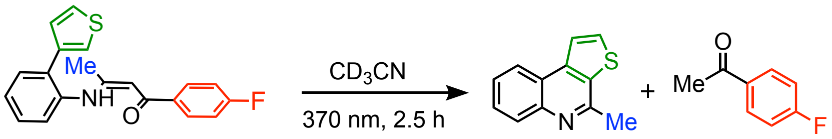

3Bd **4B** **5d**

A solution of enaminone **3Bd** (12 mM) in ~0.6 mL of acetonitrile-d_3 is taken an NMR tube and sealed. The resulting solution is irradiated at 370 nm Kessil LED (Pyrex tube placed in-between 2 Kessil LEDs placed at a distance of ~12 cm, 100% power) for 2.5 h.

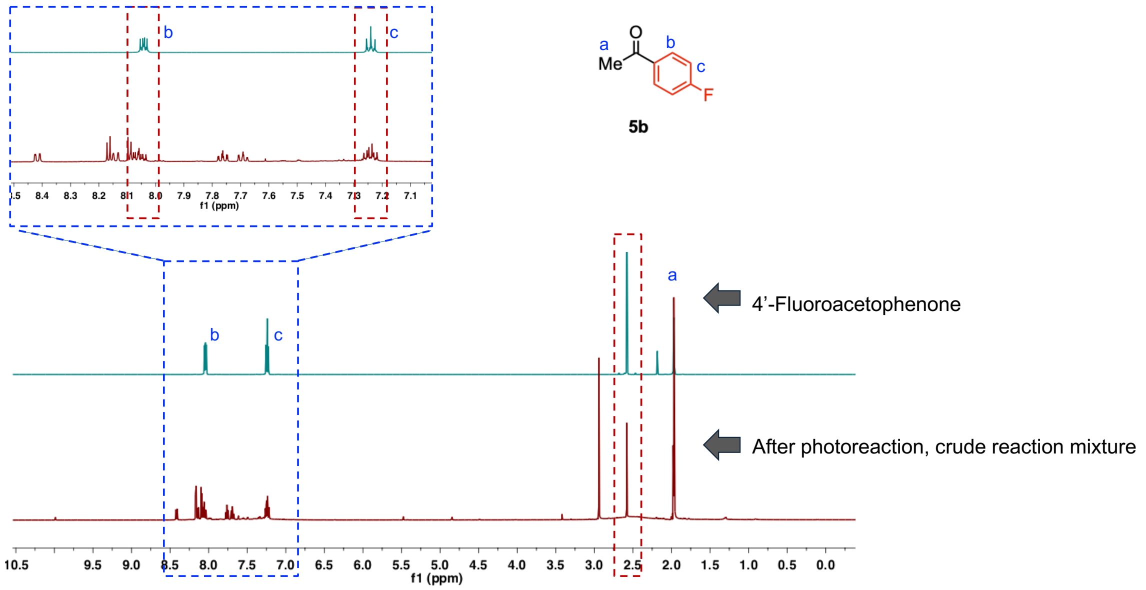

12. Quantum yield studies of enaminones.

12.1. Photolysis of actinometer.

Quantum yield of photoreaction of enaminones at 350 nm was determined using potassium ferrioxalate actinometry.[11-14]

The photochemical reaction studied in potassium ferrioxalate actinometry is shown below. The number of moles of photons absorbed per unit time is determined using this net photochemical reaction, where the number of moles of ferrous ion produced is found through absorption spectroscopy, by monitoring the complex formation of ferrous ion and 1,10 phenanthroline at 510nm. The literature reported quantum yield of potassium ferrioxalate actinometer is 1.25 at 350 nm.

$$2Fe^{3+} + C_2O_4^{2-} \longrightarrow 2Fe^{2+} + CO_2$$

$$\Phi = \frac{\text{Number of moles of product formed}}{\text{Number of moles of photons absorbed}}$$

Actinometer solution was prepared following literature reported procedure. Each actinometer solution (3 mL) was irradiated at 350 nm light (with constant stirring) employing a 450 W Xenon lamp and monochromator (SPEX 1881 0.22m spectrometer) with 3mm slit width for 2, 4, 6, 8, and 10 minutes. After irradiation, 1 mL from each sample added to a 10 mL standard flask, along with 1,10-phenanthroline and buffer solution which is made up to 10 mL (followed concentrations of solution from literature). Similarly, a non-irradiated solution was also prepared. These solutions were left to stand for one hour in dark. After one hour, absorption spectra were recorded in 1 x 1 cm cuvette (Scheme S8; left).

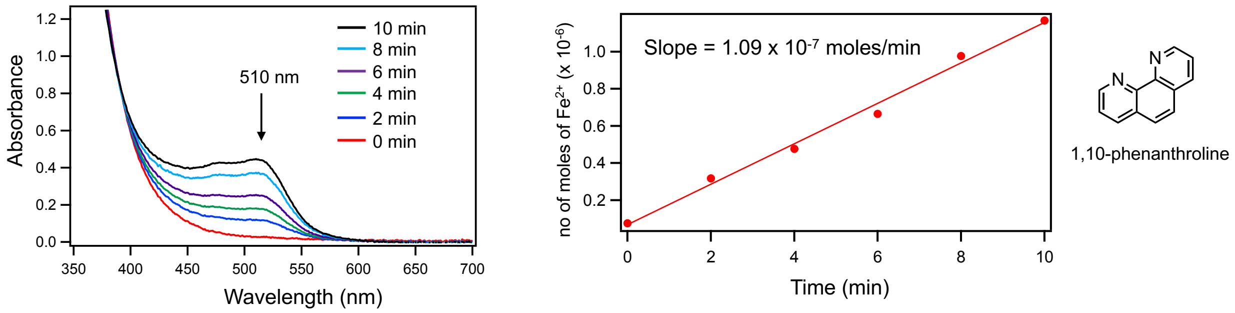

Figure S8: (a) UV-vis absorption spectra after irradiation of actinometer solution at 350 nm.(b) A plot of number of moles of ferrous ion produced as a function of time.

Considering the absorption of the complex at 510 nm (ε = 1.15 x 10^4 $M^{-1}cm^{-1}$) and dilution of the solutions, number of moles of ferrous ion produced is determined. A plot of number of moles of ferrous ion produced over time is shown in figure S8 (right), the slope of which is 1.09 x 10^{-7} $mole.min^{-1}$. Number of moles of photons absorbed per min is determined (from this slope and quantum yield of ferrioxalate actinometry at 350 nm) to be 0.872 x 10^{-7} einstein/min.

12.2. Photolysis of enaminones **3B**.

Enaminones **3B** were irradiated at 350 nm employing a xenon lamp with 3 mm slit width. Irradiation was carried out with proper stirring in methanol as solvent with a time interval of 30 mins.

12.2.1 Photolysis of enaminone **3Ba**.

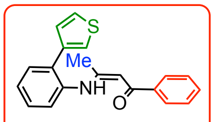

3Ba

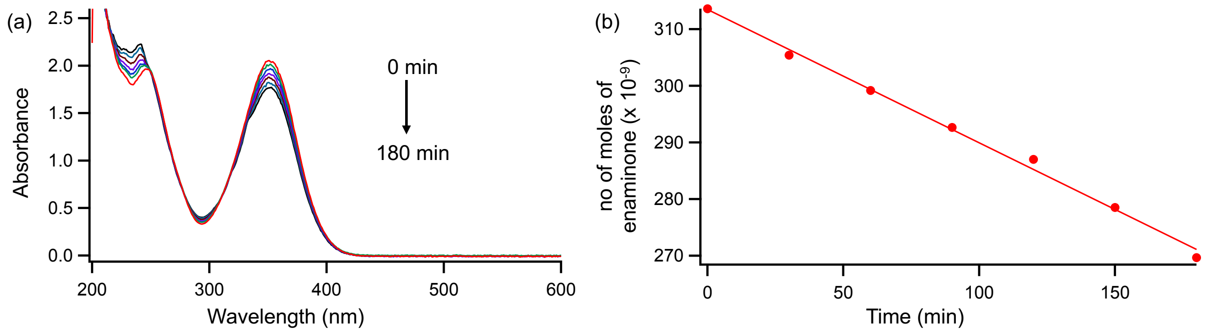

Figure S9: (a) Absorption spectra on photolysis of enaminone **3Ba** at 350 nm over a time of 180 min. (b) plot of no. of moles of enaminones *vs* time, with a slope of -2.35 x 10^{-10} mol/min.

12.2.2 Photolysis of enaminone **3Bb**.

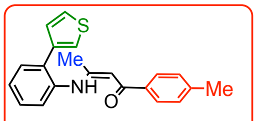

3Bb

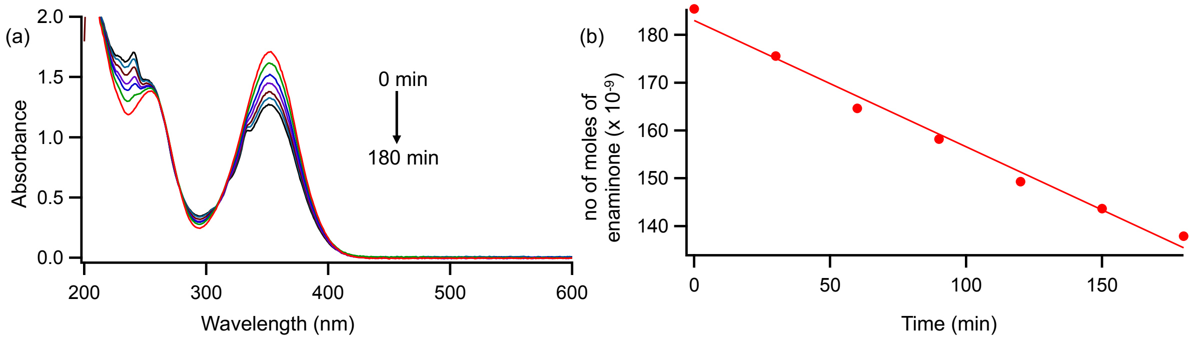

Figure S10: (a) Absorption spectra on photolysis of enaminone **3Bc** at 350 nm over a time of 180 min. (b) plot of no. of moles of enaminones *vs* time, with a slope of -2.64 x 10^{-10} mol/min.

12.2.3 Photolysis of enaminone **3Bc**.

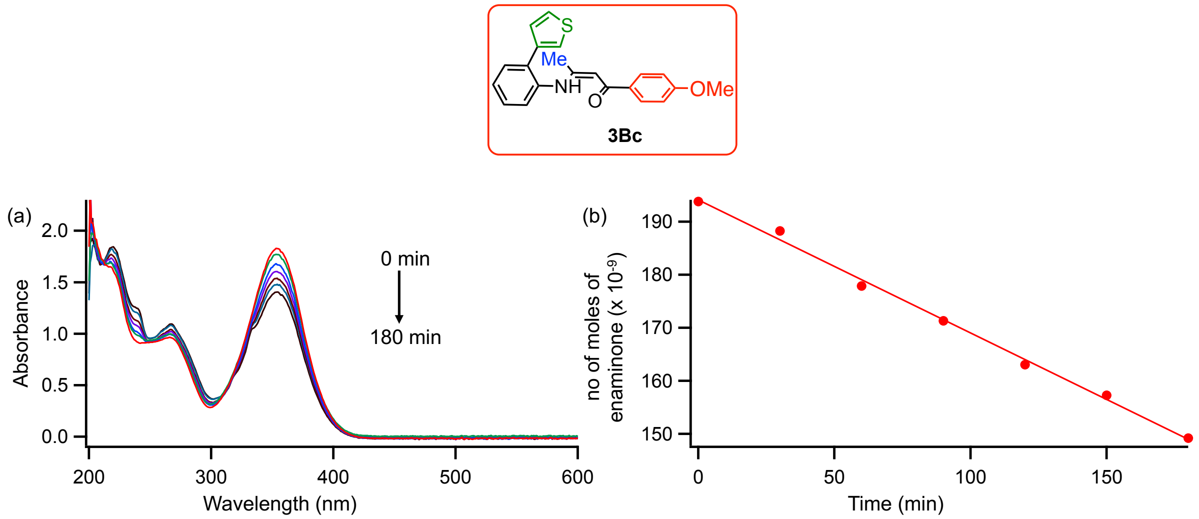

Figure S11: (a) Absorption spectra on photolysis of enaminone **3Bc** at 350 nm over a time of 180 min. (b) plot of no. of moles of enaminones *vs* time, with a slope of -2.51 x 10^{-10} mol/min.

12.2.4 Photolysis of enaminone **3Bd**.

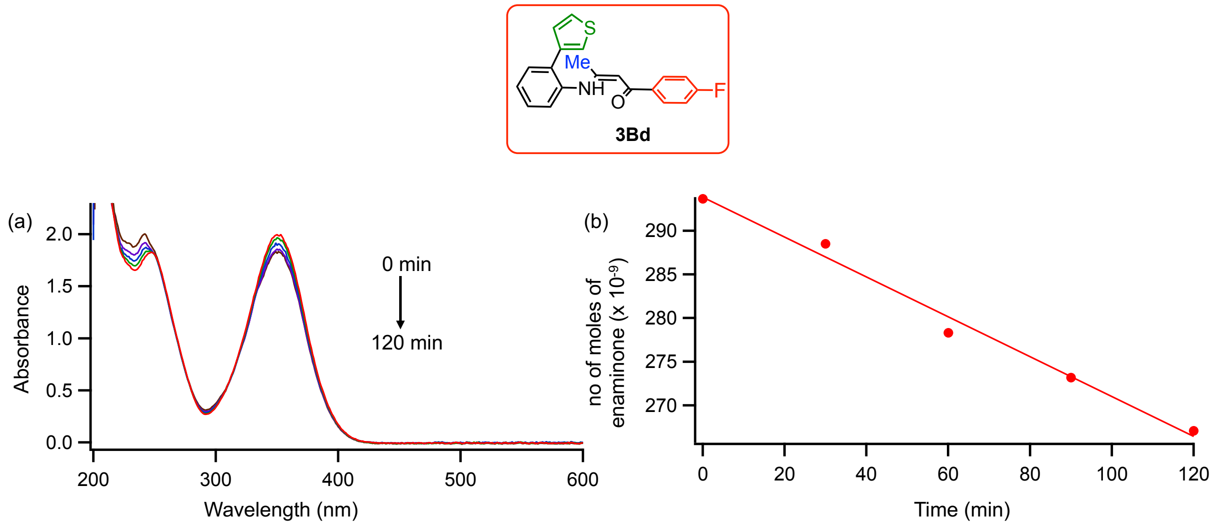

Figure S12: (a) Absorption spectra on photolysis of enaminone **3Bd** at 350 nm over a time of 120 min. (b) plot of no. of moles of enaminones *vs* time, with a slope of -2.28 x 10^{-10} mol/min.

12.2.5 Photolysis of enaminone **3Bg**.

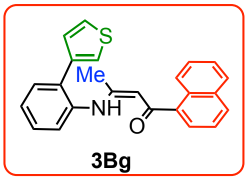

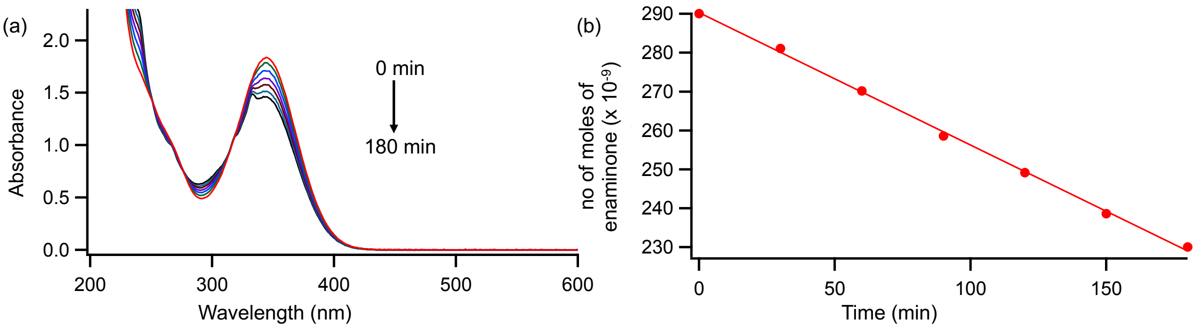

Figure S13: (a) Absorption spectra on photolysis of enaminone **3Bg** at 350 nm over a time of 180 min. (b) plot of no. of moles of enaminones *vs* time, with a slope of -3.40 x 10^{-10} mol/min.

12.3. Determination of quantum yield and efficacy of photoreaction.

$$\Phi = \frac{\text{Number of moles of product formed}}{\text{Number of moles of photons absorbed}}$$

Quantum yield of photoreaction (Φ) for enaminones **3Ba**, **3Bb**, **3Bc**, **3Bd** and **3Bg** at 350 nm in methanol is determined using potassium ferrioxalate actinometry employing the above-mentioned equation, where, no of moles of photons absorbed per minute is 0.872×10^{-7} einstein/min.
Efficacy of photoreaction is found from the product of quantum yield and extinction coefficient at 350 nm.

Table S5: Quantum yield and efficacy of photolysis of enaminones **3B**.

Enaminone	No of moles of product formed per min	Extinction coefficient @350 nm, ε ($M^{-1}cm^{-1}$)	Quantum yield Φ	Efficacy ξ ($M^{-1}cm^{-1}$)
**3Ba**	2.35×10^{-10}	19631	0.0027	53
**3Bb**	2.64×10^{-10}	27501	0.0030	83
**3Bc**	2.51×10^{-10}	27787	0.0029	80
**3Bd**	2.28×10^{-10}	20401	0.0026	53
**3Bg**	3.40×10^{-10}	18436	0.0039	72

12.4. Solvent study on photolysis of enaminone **3Bd**.

Enaminone **3Bd** is subjected to photolysis in solvents such as methanol, ethanol, acetonitrile, ethyl acetate, dichloromethane and toluene at 350 nm employing xenon lamp .

12.4.1 Photolysis of enaminone **3Bd** in methanol

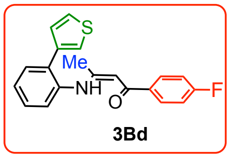

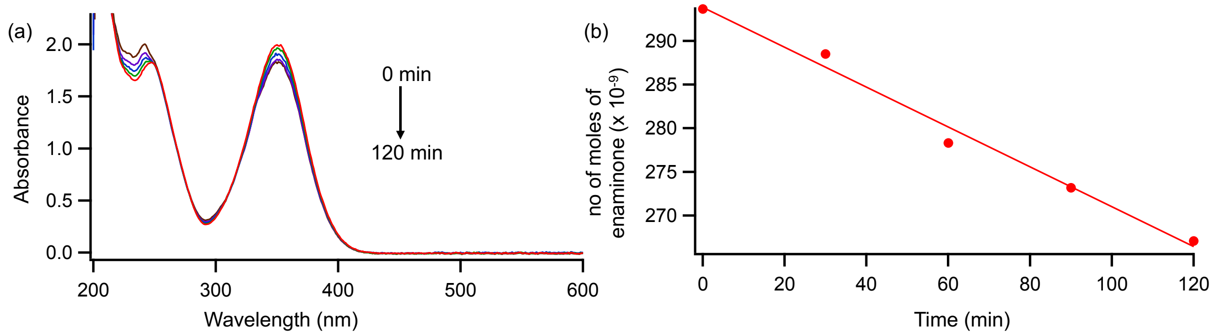

Figure S14: (a) Absorption spectra on photolysis of enaminone **3Bd** in methanol at 350 nm with a slit width of 3mm, over a time of 120 min. (b) plot of no. of moles of enaminones *vs* time, with a slope of -2.28×10^{-10} mol/min.

12.4.2 Photolysis of enaminone **3Bd** in methanol

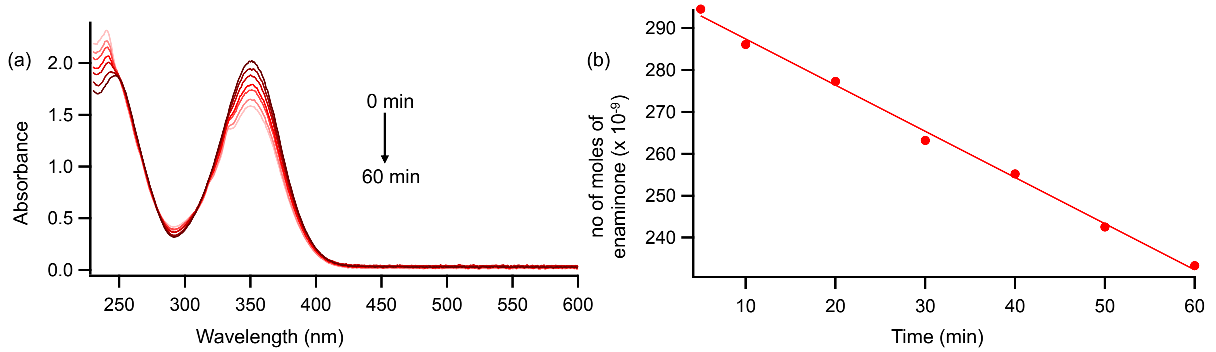

Figure S15: (a) Absorption spectra on photolysis of enaminone **3Bd** in ethanol at 350 nm with a slit width of 6mm, over a time of 60 min. (b) plot of no. of moles of enaminones *vs* time, with a slope of -1.103×10^{-9} mol/min.

12.4.3 Photolysis of enaminone **3Bd** in ethanol

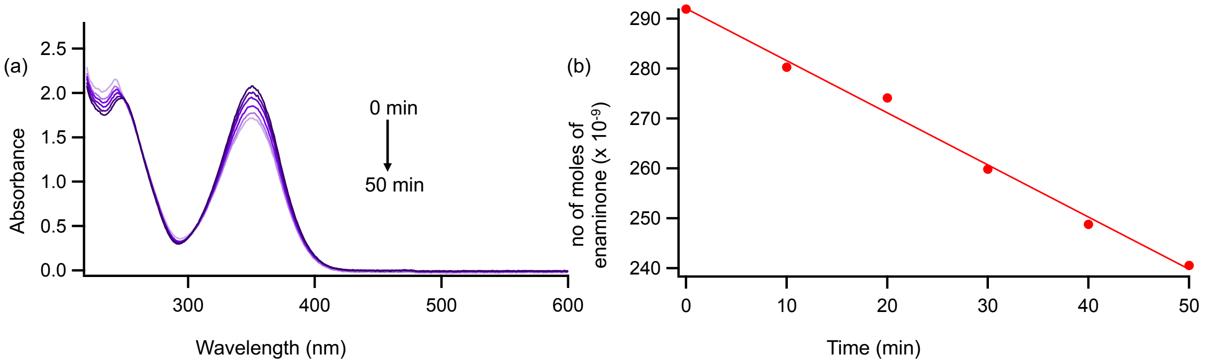

Figure S16: (a) Absorption spectra on photolysis of enaminone **3Bd** in ethanol at 350 nm with a slit width of 6mm, over a time of 50 min. (b) plot of no. of moles of enaminones *vs* time, with a slope of -1.0441×10^{-9} mol/min.

12.4.4 Photolysis of enaminone **3Bd** in acetonitrile

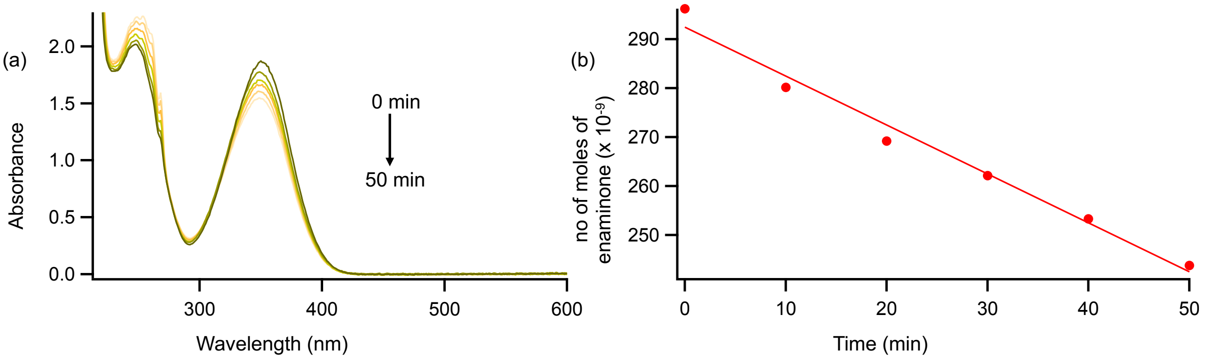

Figure S17: (a) Absorption spectra on photolysis of enaminone **3Bd** in acetonitrile at 350 nm with a slit width of 6mm, over a time of 50 min. (b) plot of no. of moles of enaminones *vs* time, with a slope of -9.9885×10^{-10} mol/min.

12.4.5 Photolysis of enaminone **3Bd** in ethyl acetate

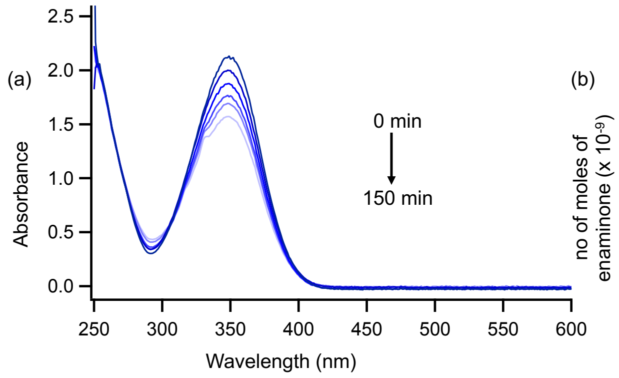

Figure S18: (a) Absorption spectra on photolysis of enaminone **3Bd** in ethyl acetate at 350 nm with a slit width of 6mm, over a time of 150 min. (b) plot of no. of moles of enaminones *vs* time, with a slope of -5.1264 x 10^{-10} mol/min.

12.4.6 Photolysis of enaminone **3Bd** in toluene

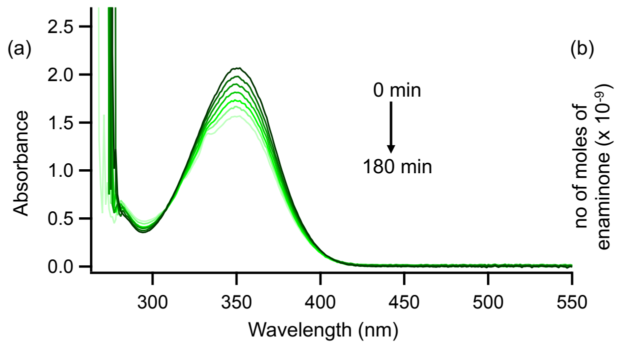

Figure S19: (a) Absorption spectra on photolysis of enaminone **3Bd** in toluene at 350 nm with a slit width of 6mm, over a time of 180 min. (b) plot of no. of moles of enaminones *vs* time, with a slope of -4.5542 x 10^{-10} mol/min.

Table S 6: Quantum yield and efficacy of photolysis of enaminones **3Bd** in different solvents.

Solvent	No of moles of product formed per min	Extinction coefficient @350 nm, ε ($M^{-1}cm^{-1}$)	Quantum yield Φ	Efficacy ξ ($M^{-1}cm^{-1}$)
Methanol	2.3 x 10^{-10}	20401 $M^{-1}cm^{-1}$	0.0026	53
Ethanol	2.1 x 10^{-10}	21367 $M^{-1}cm^{-1}$	0.0024	51
Acetonitrile	1.9 x 10^{-10}	18970 $M^{-1}cm^{-1}$	0.0022	41
Ethyl acetate	1.0 x 10^{-10}	20811 $M^{-1}cm^{-1}$	0.0011	24
Toluene	0.9 x 10^{-10}	18172 $M^{-1}cm^{-1}$	0.0010	19

13. Photolysis of enaminones 3Bl, 3Bm, 3Bn

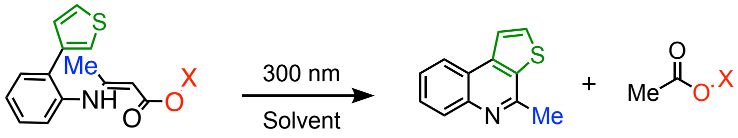

3B **4B** **5**

A solution of enaminone **3B** (0.67 mM) in 5-10 mL of solvent is taken a quartz tube and sealed. The solution is degassed with N_2 for 20 mins. The resulting solution irradiated at 300 nm. After the completion of reaction, solvent is removed under reduced pressure.

Formation of product is confirmed by High Resolution Mass Spectrometry (HRMS).

13.1. Photolysis of enaminone **3Bk**.

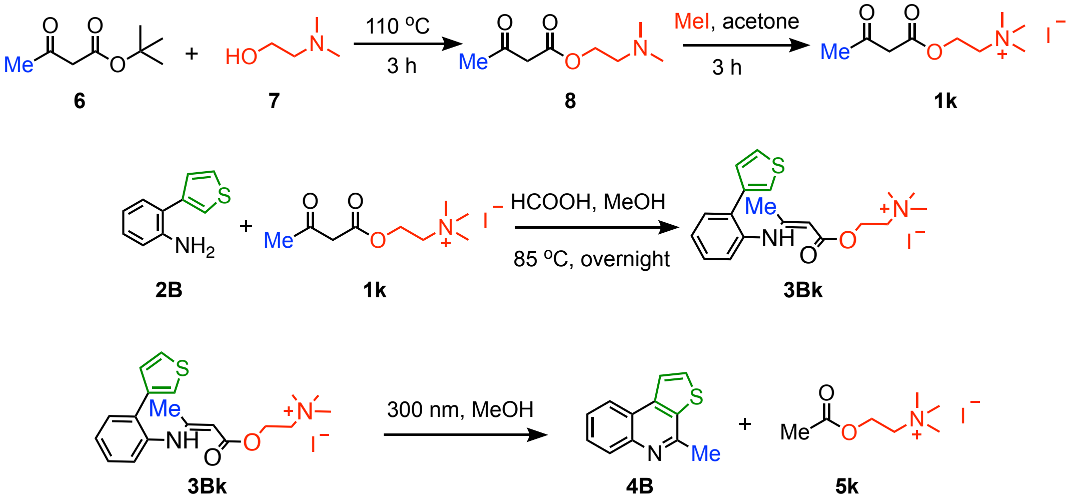

Scheme S12: Synthesis scheme for photolysis of enaminone **3Bk**.

HRMS-ESI (m/z) ([M + H]$^+$): Calculated: 200.0534, Observed: 200.0530, |Δm| = 1.9 ppm

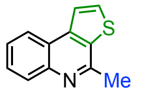

4B

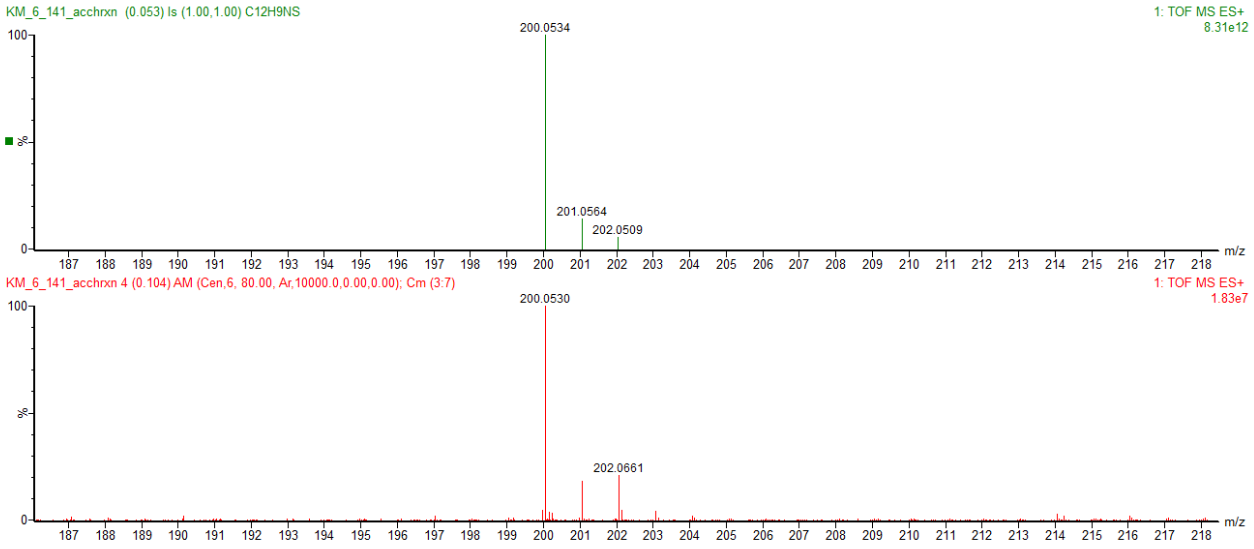

Figure S20: HRMS data of quinoline photoproduct **4B** on photolysis of enaminone **3Bk**.

HRMS-ESI (m/z) ($[M]^+$): Calculated: 146.1181, Observed: 146.1181, |Δm| = 0 ppm

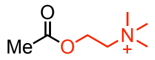

5k

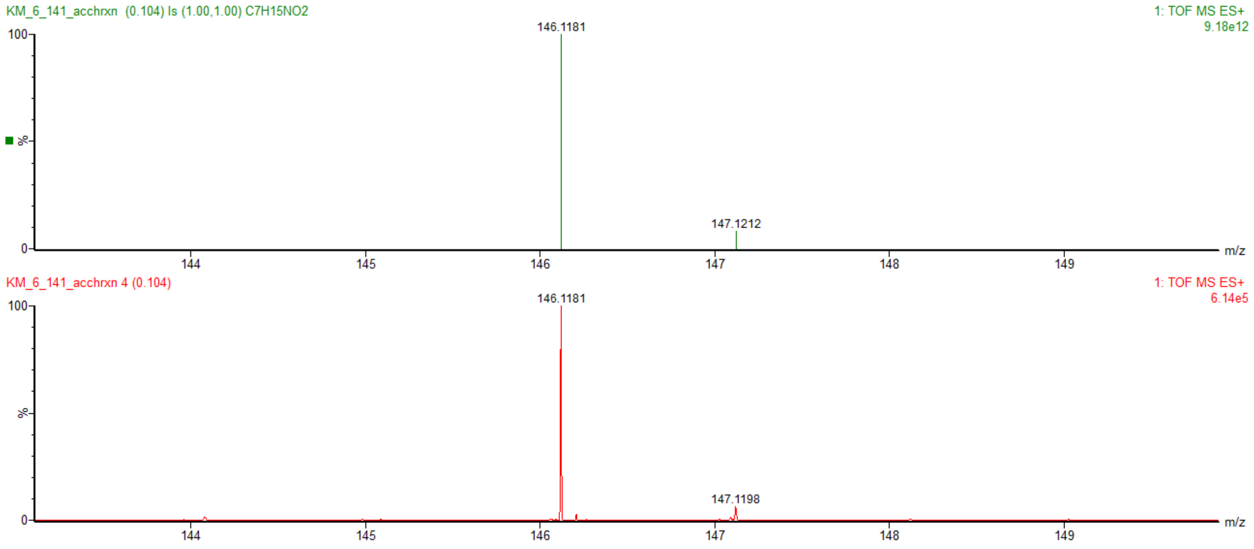

Figure S21: HRMS data of quinoline photoproduct **5k** on photolysis of enaminone **3Bk**.

13.2. Photolysis of enaminone **3Bk** in water.

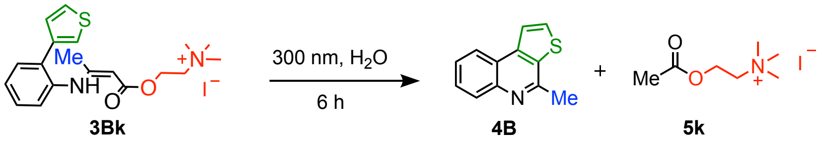

Scheme S13: Synthesis scheme for photolysis of enaminone **3Bk**.

A solution of enaminone **3Bk** (0.35 mM) in 15 mL of water is taken a quartz tube and sealed. The resulting solution irradiated at 300 nm for 6 h. After the completion of reaction, a known amount of triphenyl methane is added. To the reaction mixture, dichloromethane was added and the compounds from the aqueous layer was extracted. Combined organic layers were dried over anhydrous sodium sulfate, filtered and concentrated under reduced pressure. Due to insolubility of **4B** in water the experimental protocol carries significant error (±10).

NMR yield of **4B** obtained: 23%.

13.3. Photolysis of enaminone **3Bl**.

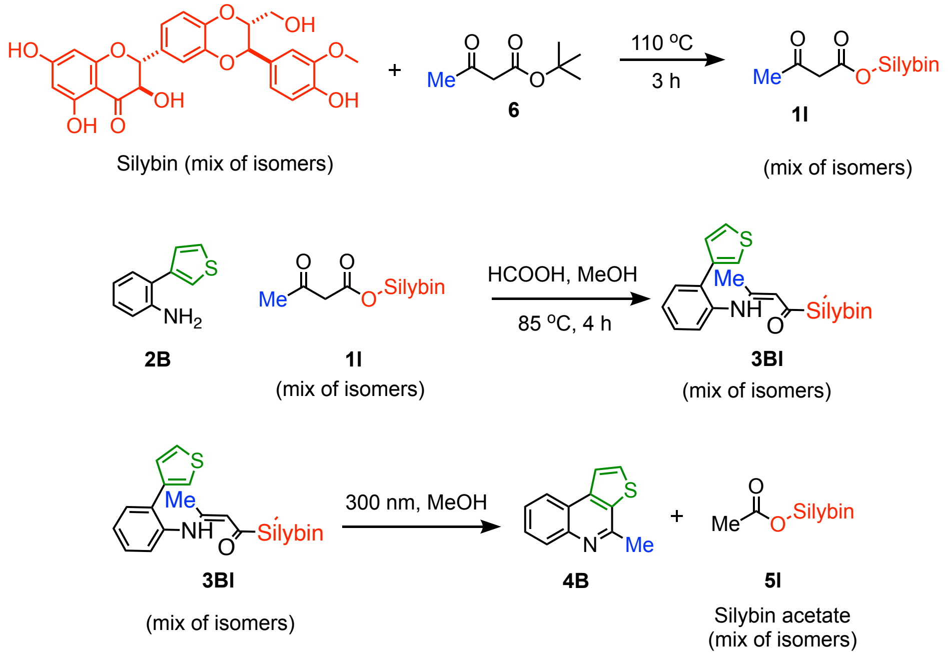

Scheme S14: Synthesis and photolyzing reaction of enaminone **3Bl**.

Note: The position of functionalization in Silybin in our system is not known and is characterized only by HRMS.

HRMS-ESI (m/z) ([M + H]$^+$): Calculated: 724.1852, Observed: 724.1848, |Δm| = 0.55 ppm

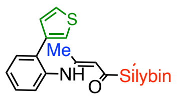

3BI
(mix of isomers)

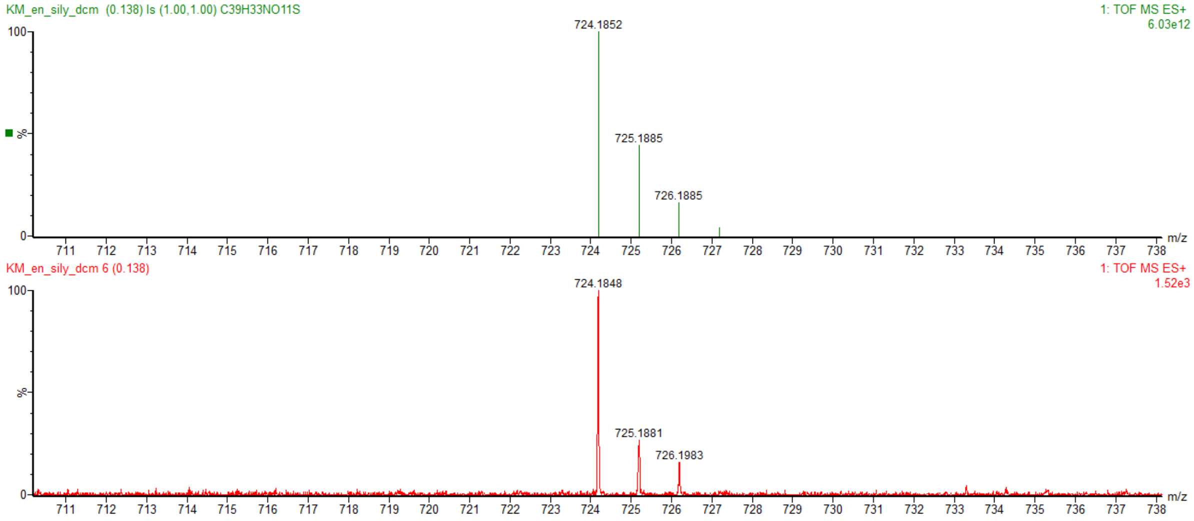

Figure S22: HRMS data of enaminone **3BI**.

HRMS-ESI (m/z) ([M + H]$^+$): Calculated: 200.0534, Observed: 200.0535, |Δm| = 0.5 ppm

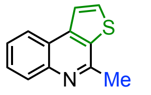

4B

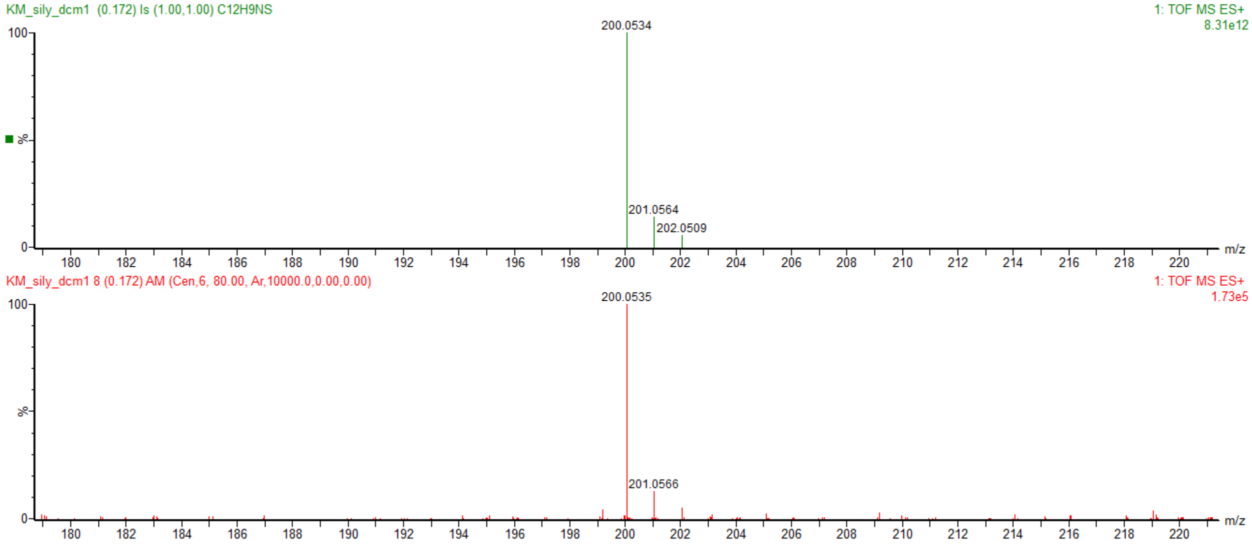

Figure S23: HRMS data of quinoline photoproduct **4B** on photolysis of enaminone **3BI**.

HRMS-ESI (m/z) ([M + H]$^+$): Calculated: 525.1397, Observed: 525.1404, |Δm| = 1.3 ppm

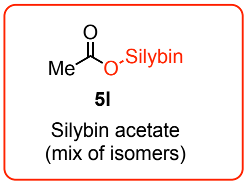

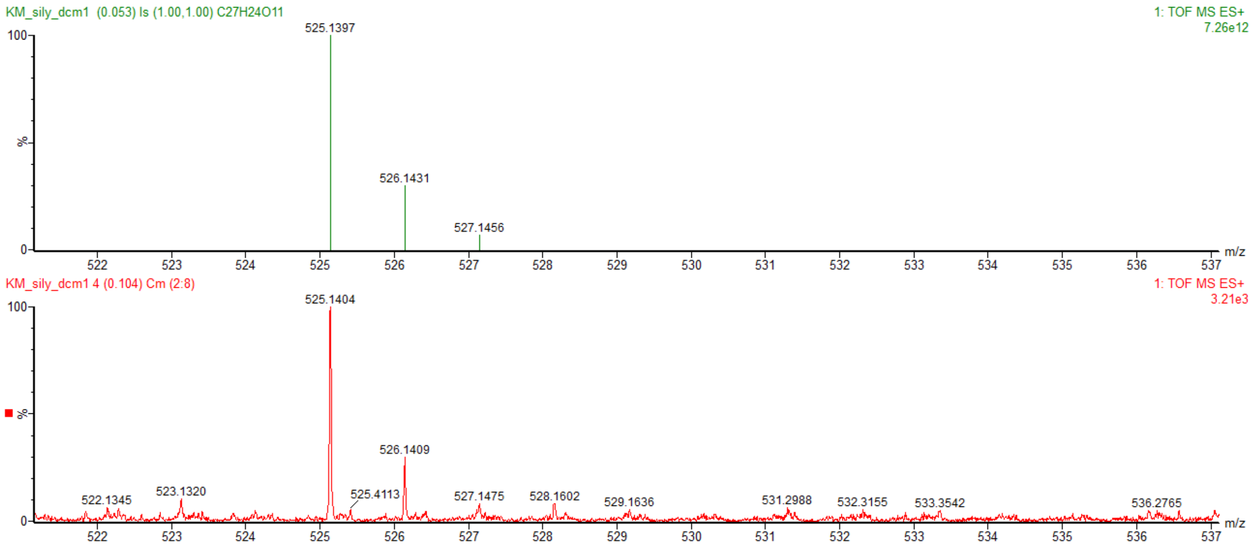

Figure S24: HRMS data of quinoline photoproduct **5l** on photolysis of enaminone **3Bl**.

13.4. Photolysis of enaminone **3Bm**.

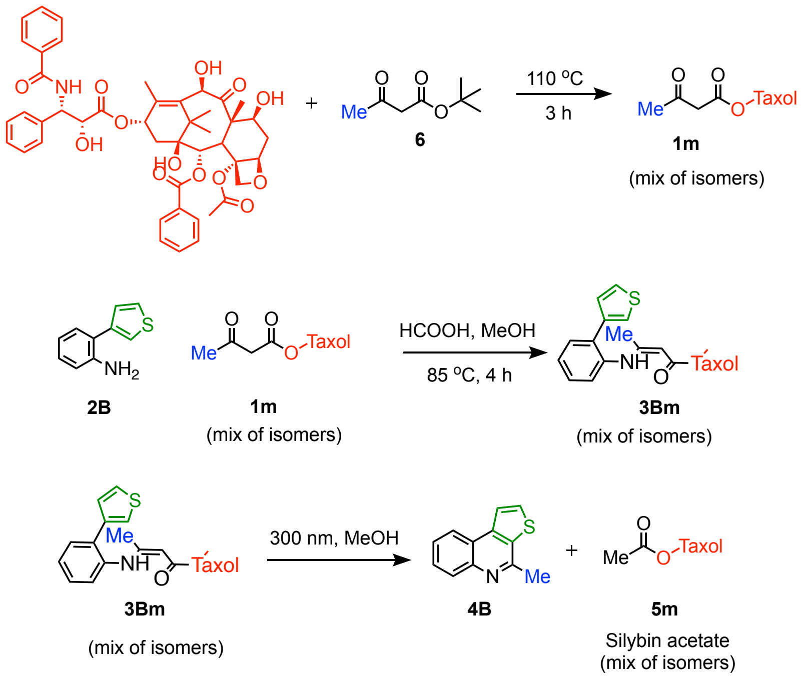

Scheme S15: Synthesis and photolyzing reaction of enaminone **3Bm**.

Note: The position of functionalization in Taxol in our system is not known and is characterized only by HRMS.

HRMS-ESI (m/z) ([M + H]$^+$): Calculated: 896.3494, Observed: 896.3476, |Δm| = 2 ppm

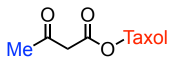

1m

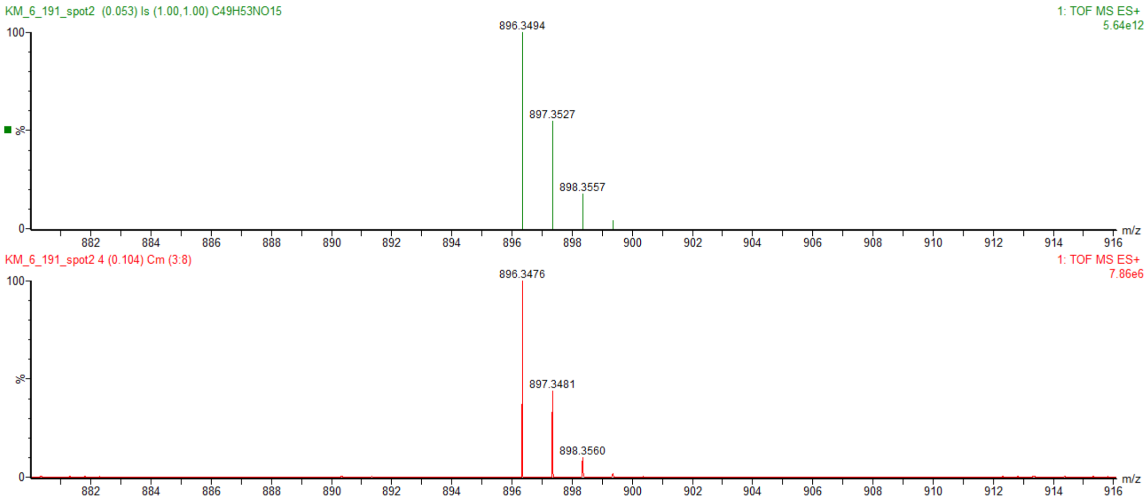

Figure S25: HRMS data of diketone**1m**.

HRMS-ESI (m/z) ([M + H]$^+$): Calculated: 1053.3844, Observed: 1053.3756, |Δm| = 8.16 ppm

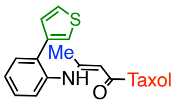

3Bm

(mix of isomers)

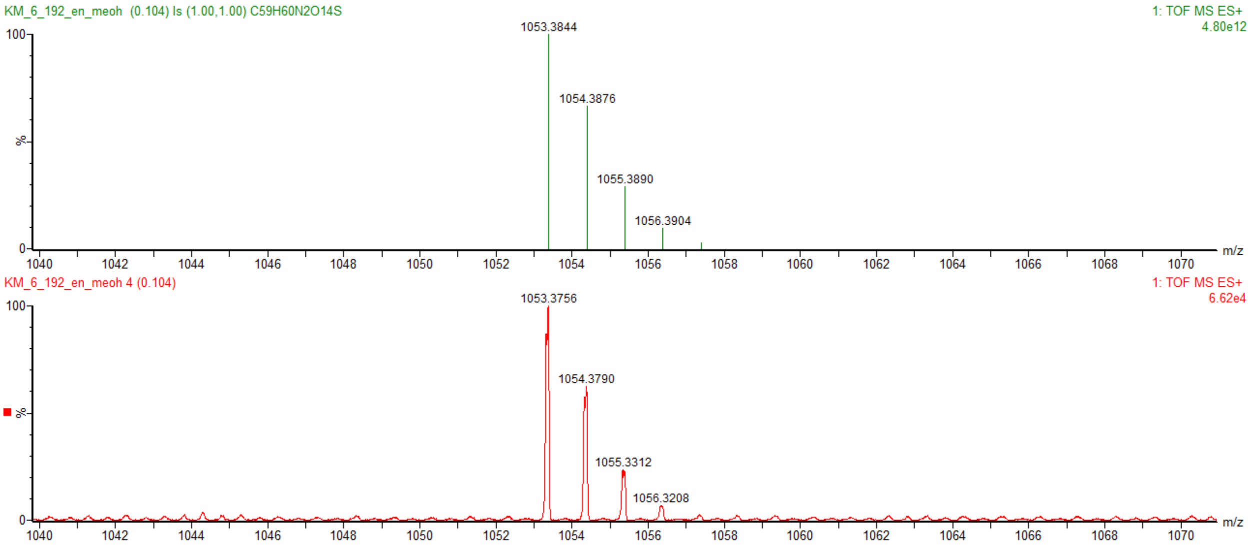

Figure S26: HRMS data of enaminone **3Bm**.

HRMS-ESI (m/z) ([M + H]$^+$): Calculated: 200.0534, Observed: 200.0525, |Δm| = 4.5 ppm

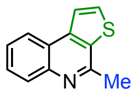

4B

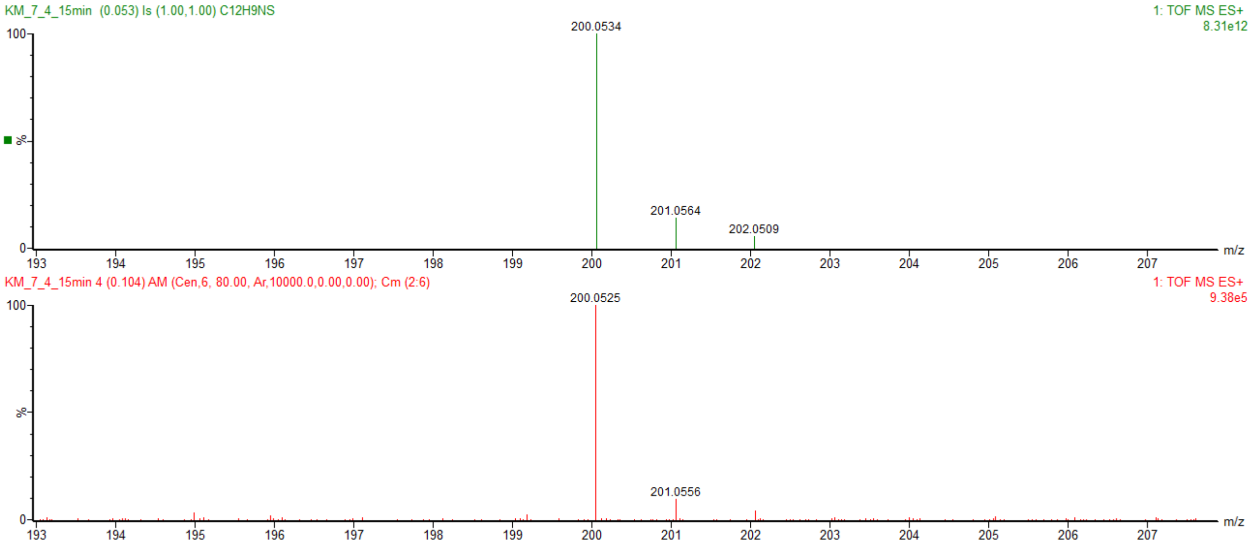

Figure S27: HRMS data of quinoline photoproduct **4B** on photolysis of enaminone **3Bm**.

HRMS-ESI (m/z) ([M + H]$^+$): Calculated: 854.3388, Observed: 854.3319, |Δm| = 8.07 ppm

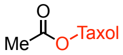

5m

Taxol acetate
(mix of isomers)

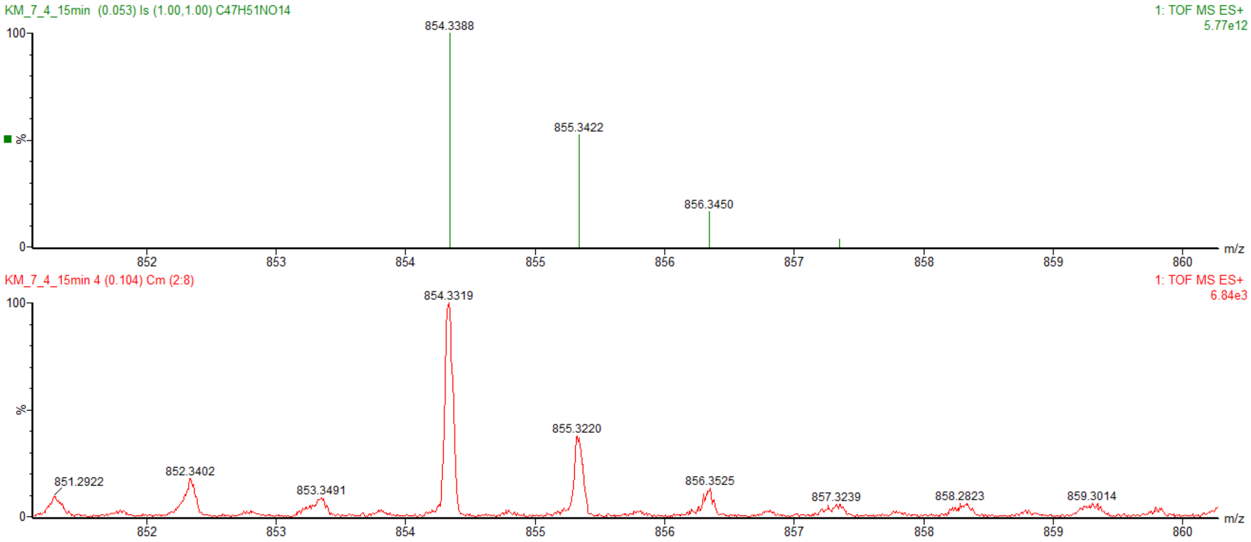

Figure S28: HRMS data of Taxol acetate **5m** on photolysis of enaminone **3Bm**.

14. Photolysis of enaminone 3Ha

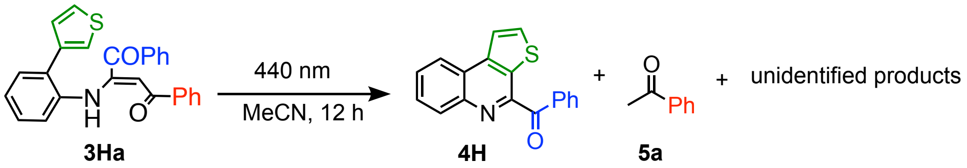

A solution of enaminone **3Ha** (0.67 mM) in acetonitrile is taken a Pyrex tube and sealed. The solution is degassed with N_2 for 20 mins. The resulting solution is irradiated at 440 nm Kessil LED (Pyrex tube placed in-between 2 Kessil LEDs placed at a distance of ~12 cm, 100% power) for 12 h with stirring. Formation of photoproduct **4H** was confirmed by ^{1}H NMR spectroscopy and High-Resolution Mass Spectrometry (HRMS). There were also other unidentified side products which were not characterized in the present study.

Note: Acetonitrile was employed as a solvent due to limited solubility of **3Ha** in methanol.

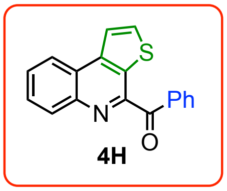

^{1}H NMR (600 MHz, $CDCl_3$) δ 8.44 – 8.42 (m, 1H), 8.41 – 8.38 (m, 1H), 8.37 – 8.33 (m, 1H), 8.08 (d, J = 5.4 Hz, 1H), 8.05 (d, J = 5.4 Hz, 1H), 7.81 – 7.77 (m, 2H), 7.68 – 7.63 (m, 2H), 7.58 – 7.54 (m, 2H).

HRMS-ESI (m/z) ([M + H]$^+$): Calculated: 290.0640, Observed: 290.0624, |Δm| = 5.5 ppm

HRMS-ESI (m/z) ([M + H]$^+$): Calculated: 290.0640, Observed: 290.0624, |Δm| = 5.5 ppm

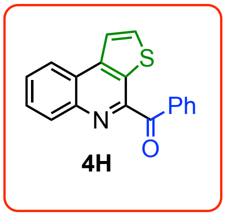

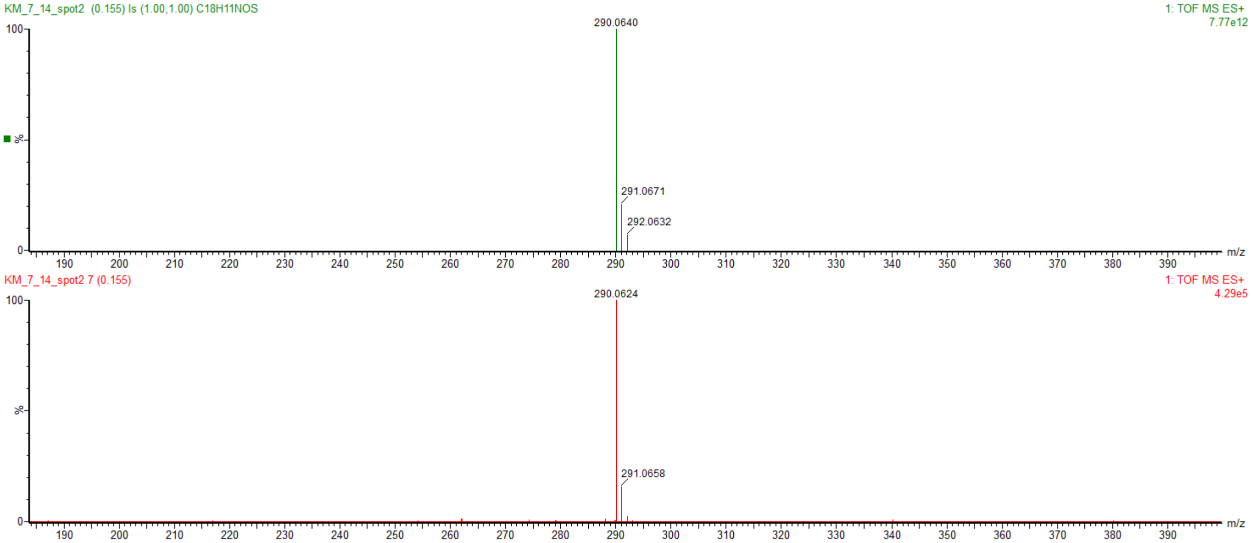

Figure S29: HRMS data of quinoline photoproduct **4H**.

15. Emission study of photoproduct.

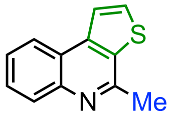

4B

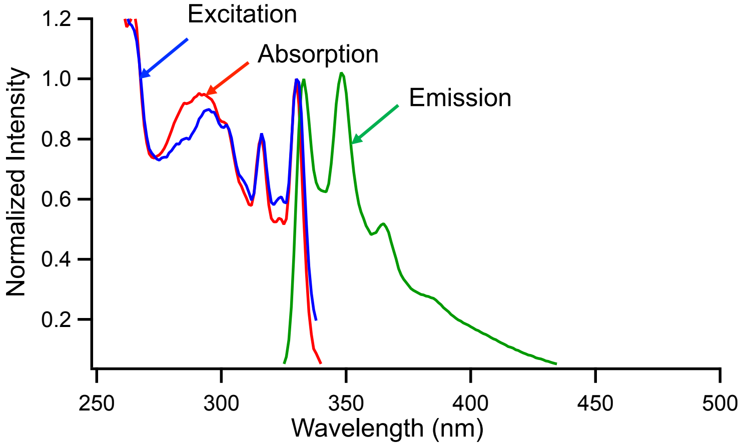

Figure S30: Absorption spectrum (red), excitation spectrum (blue) and emission spectrum (green) of photoproduct **4B** in acetonitrile (all spectra are normalized).

16. Emission study on photolysis of enaminone.

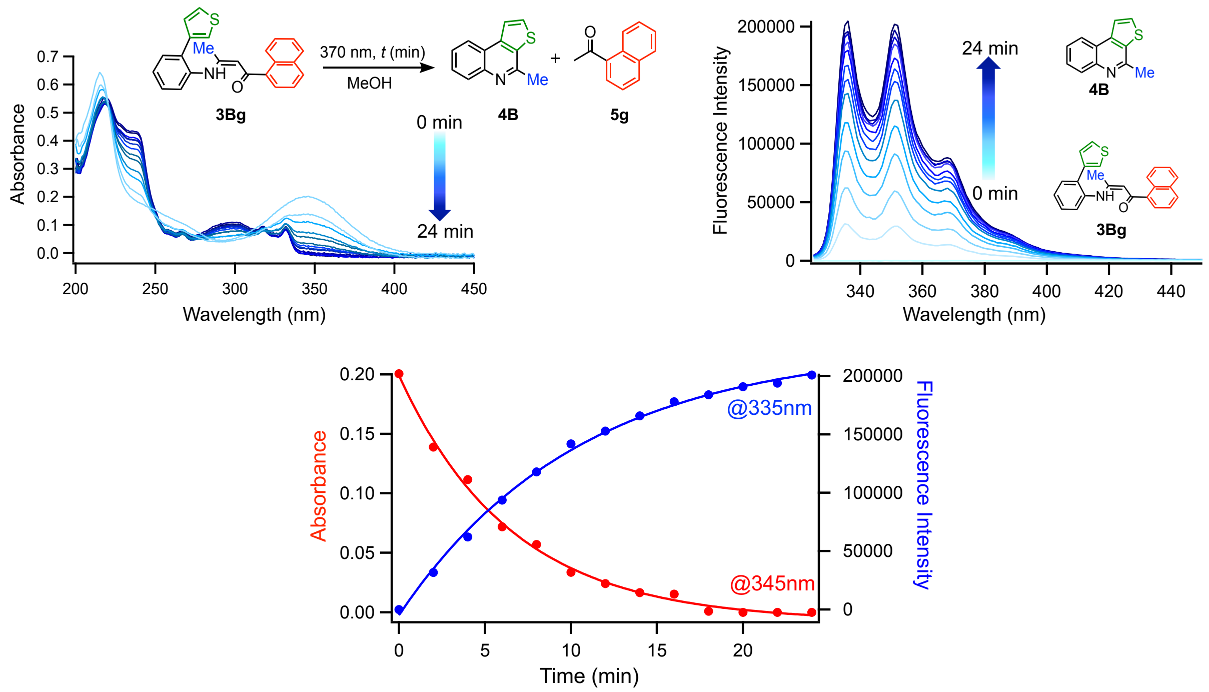

Figure S31: Photolysis study of enaminone **3Bg**.

Enaminone **3Bg** (absorbance ~2) was irradiated at 370 nm Kessil LED (employing a Kessil LED with 25% power, placed at a distance of 10 cm) in a cuvette at a time interval of 2 min. Absorption and emission spectra was recorded after 2 min of irradiation for a time period of 24 min. A plot of absorption at 345 nm and emission intensity at 335 nm *vs* time is shown in figure S31-bottom.

17. Characterization of photoproducts.

17.1. Characterization of photoproduct **4A**.

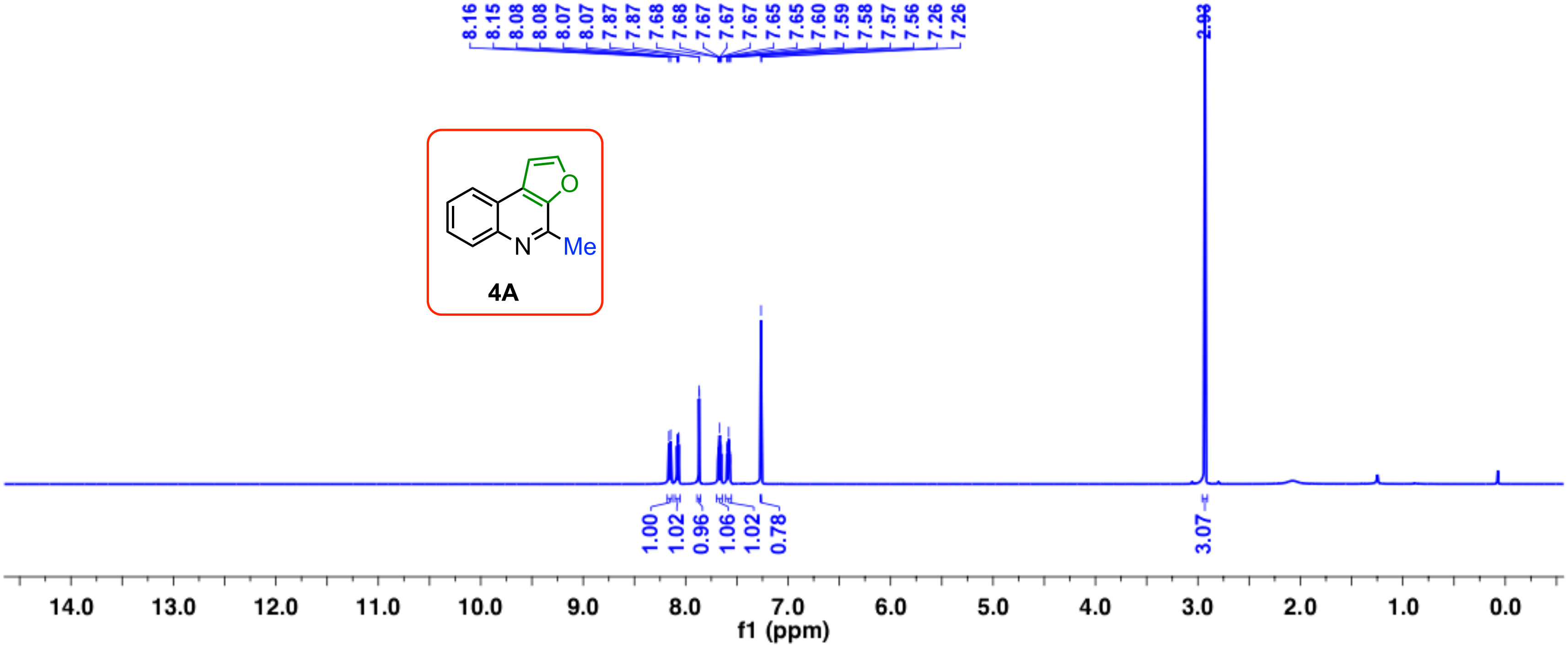

Figure S32: ^{1}H NMR of quinoline photoproduct **4A**.

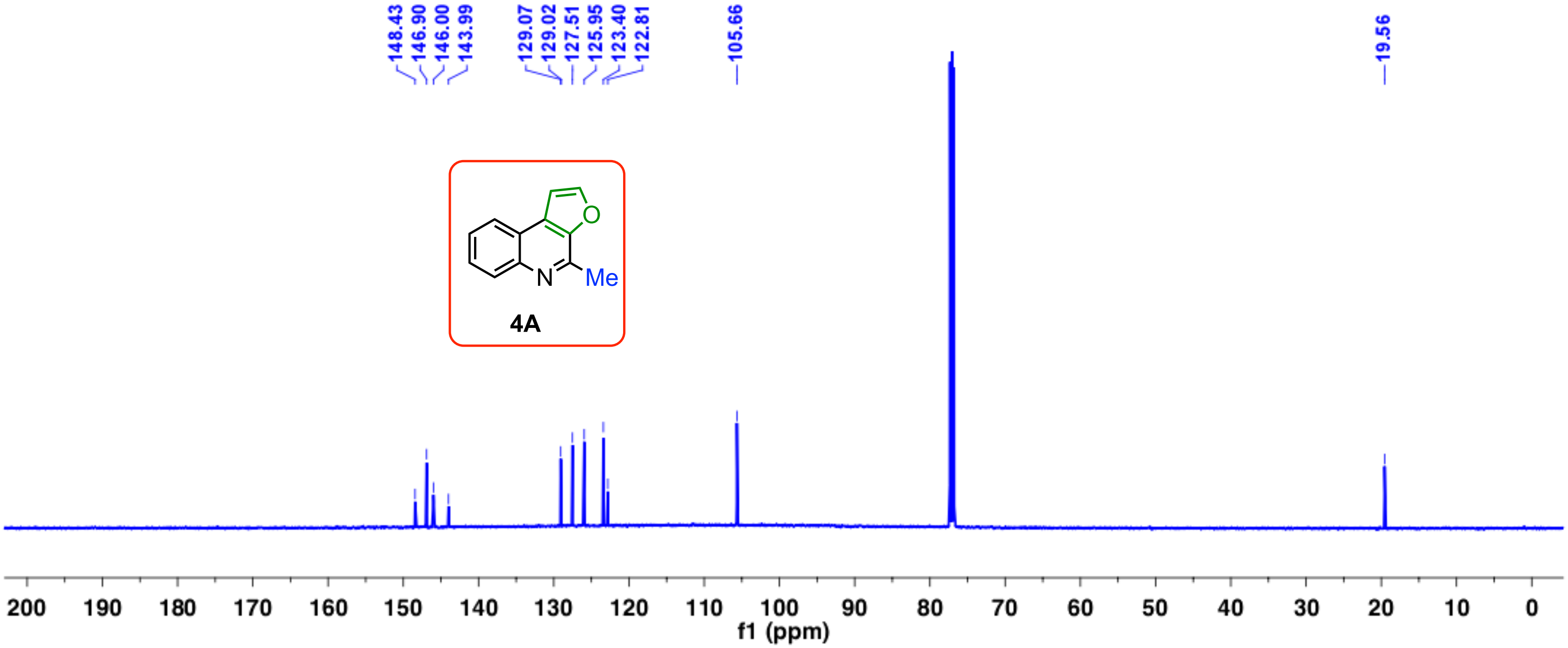

Figure S33: ^{13}C NMR of quinoline photoproduct **4A**.

HRMS-ESI (m/z) ([M + H]$^+$):

Calculated: 184.0762

Observed: 184.0762

|Δm| = 0 ppm

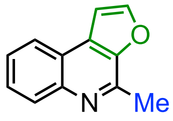

4A

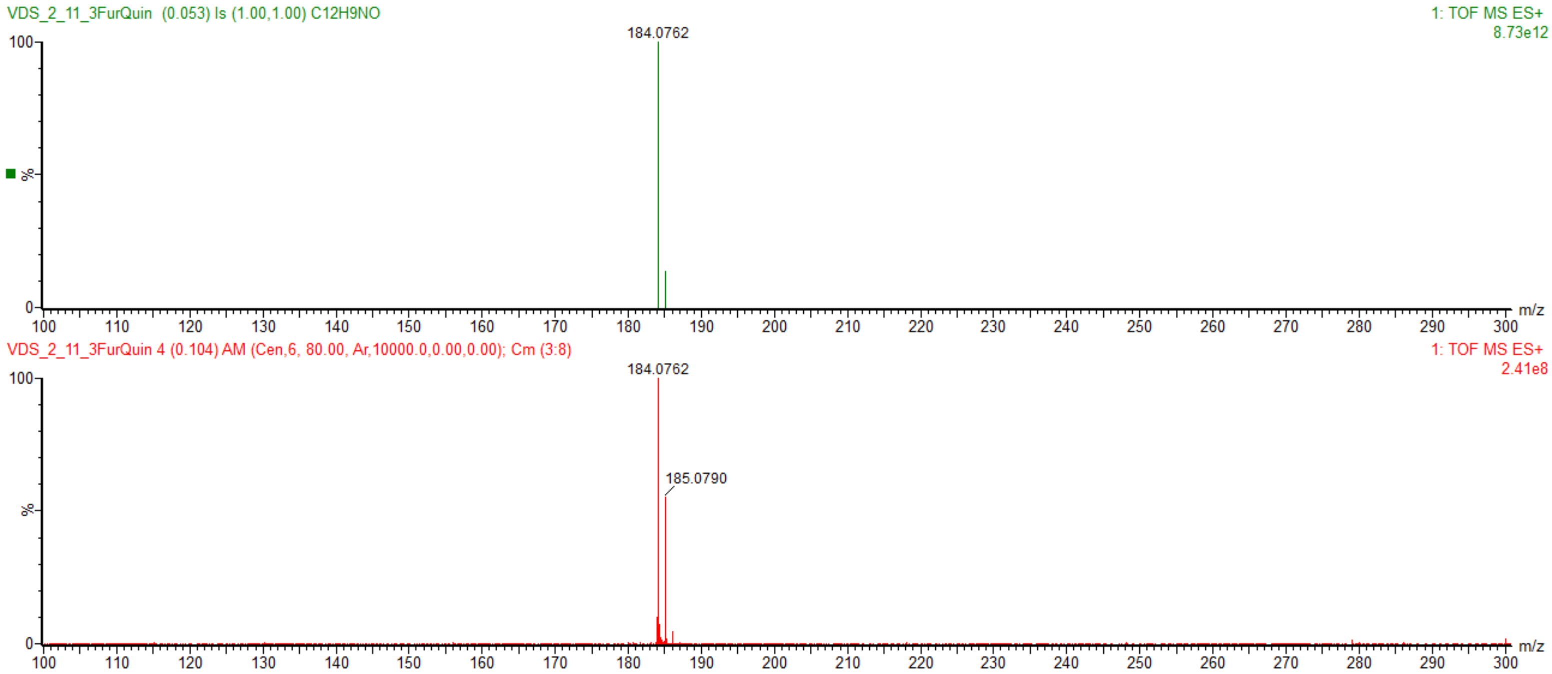

Figure S34: HRMS data of quinoline photoproduct **4A**.

17.2. Characterization of photoproduct **4B**.

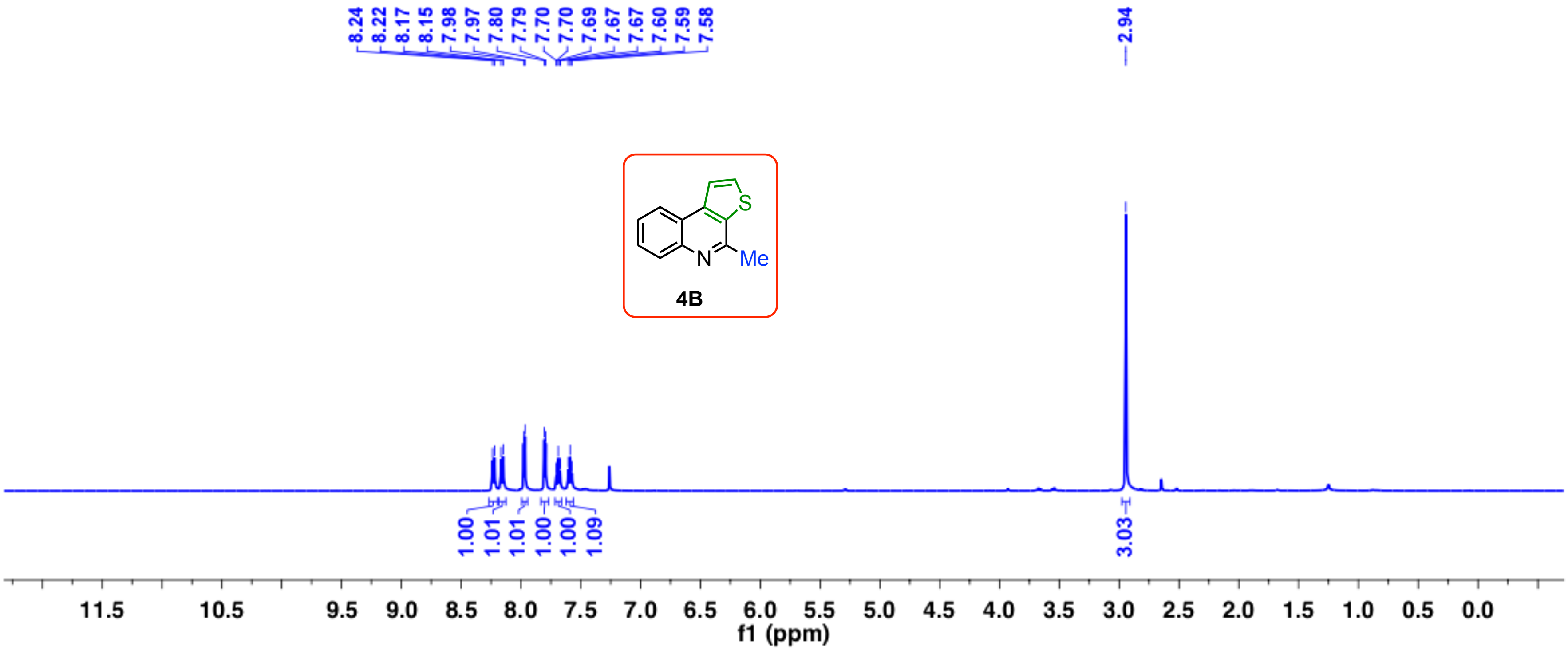

Figure S35: ^{1}H NMR of quinoline photoproduct **4B**.

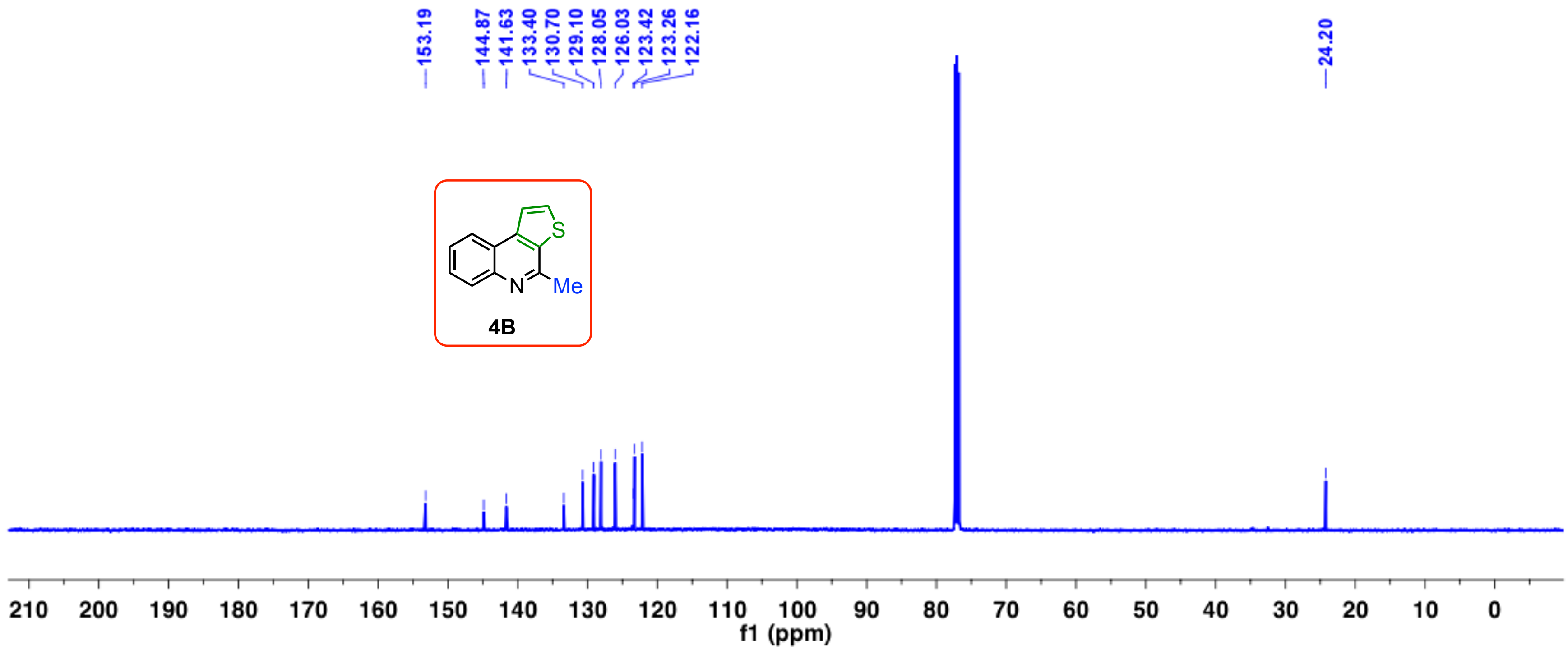

Figure S36: ^{13}C NMR of quinoline photoproduct **4B**.

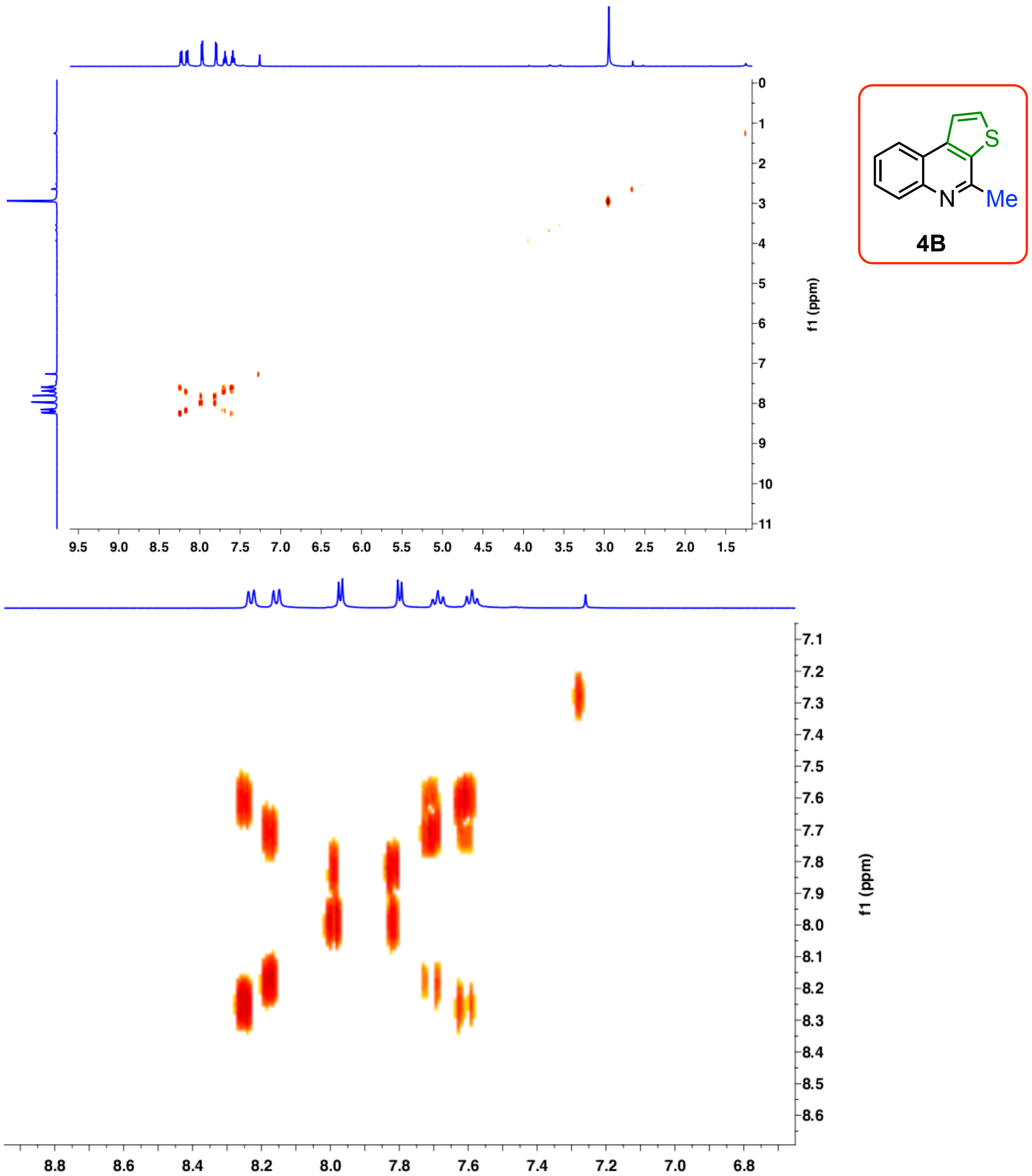

Figure S37: 2D NMR spectra (COSY) of quinoline photoproduct **4B**.

HRMS-ESI (m/z) ([M + H]$^+$):

Calculated: 200.0534

Observed: 200.0530

|Δm| = 1.9 ppm

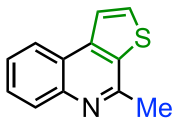

4B

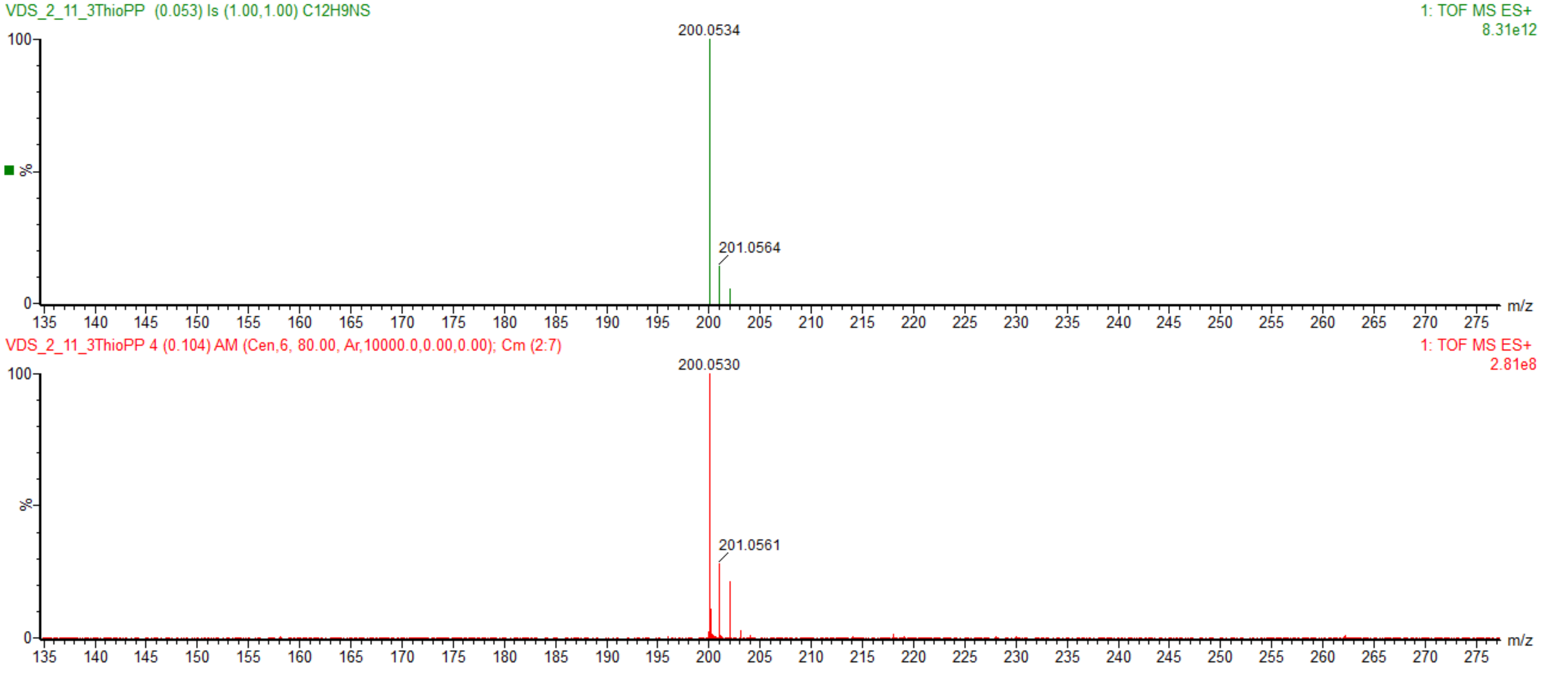

Figure S38: HRMS data of quinoline photoproduct **4B**.

17.3. Characterization of photoproduct **4C**.

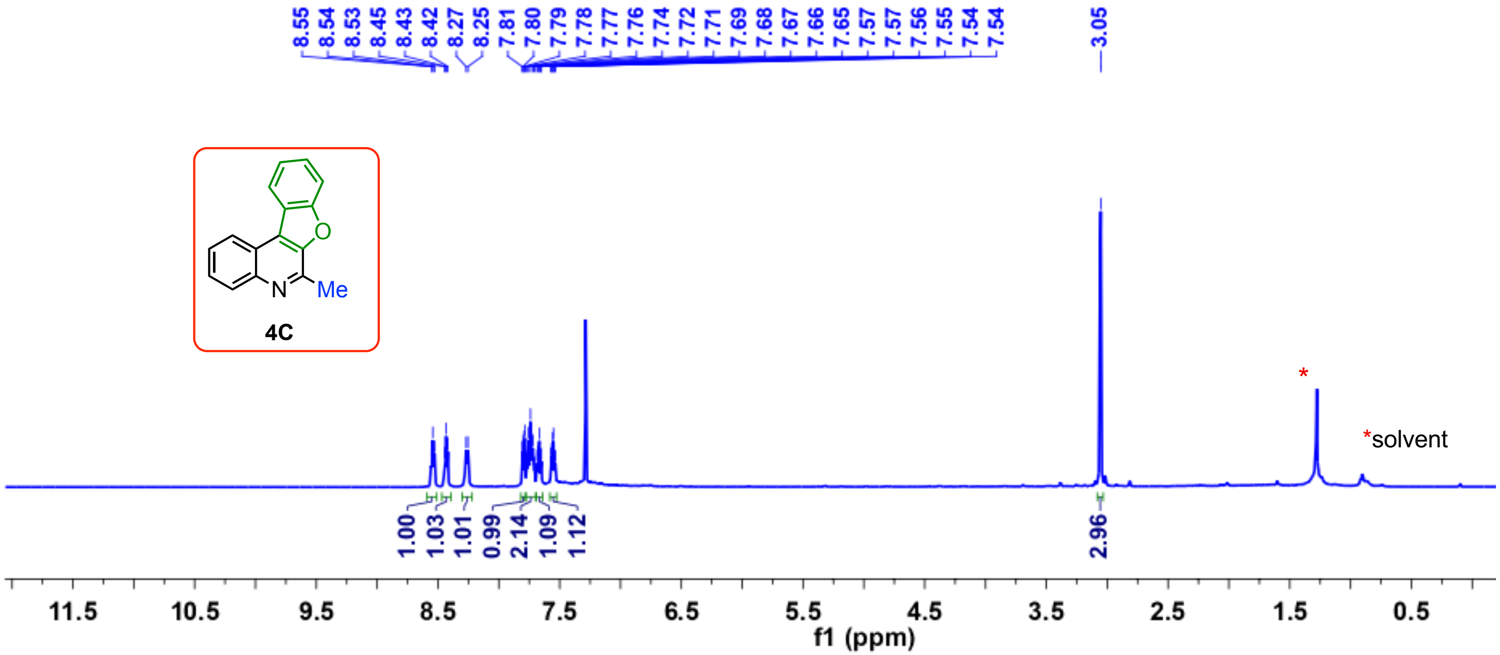

Figure S39: ^{1}H NMR of quinoline photoproduct **4C**.

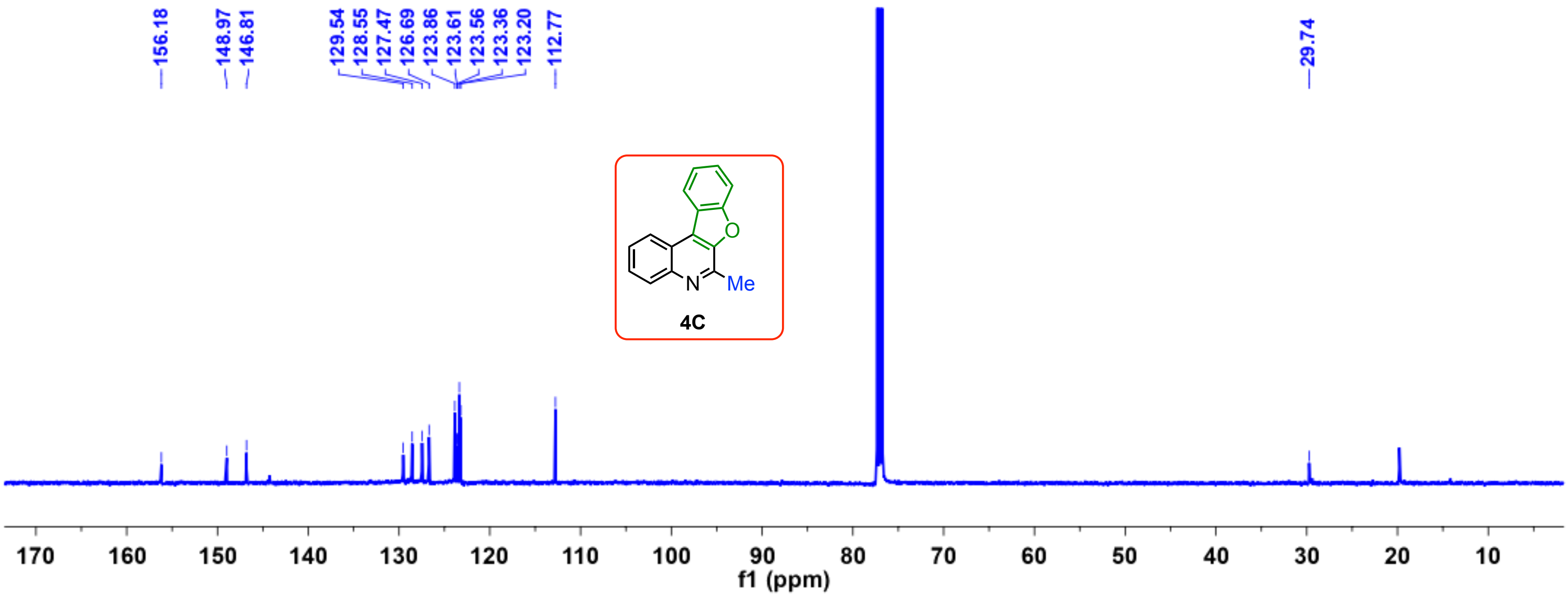

Figure S40: ^{13}C NMR of quinoline photoproduct **4C**.

HRMS-ESI (m/z) ([M + H]$^{+}$):

Calculated: 234.0919

Observed: 234.0930

|Δm| = 4.7 ppm

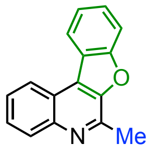

4C

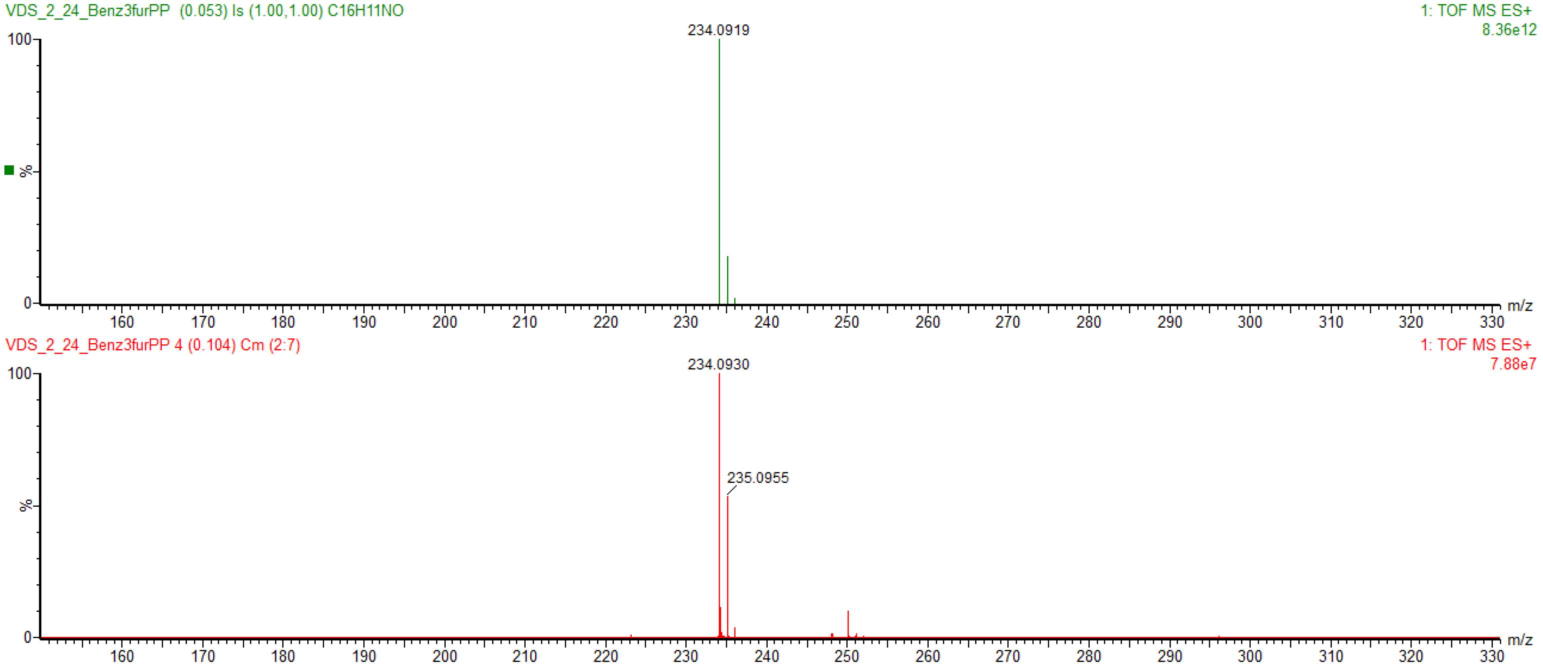

Figure S41: HRMS data of quinoline photoproduct **4C**.

17.4. Characterization of photoproduct **4D**.

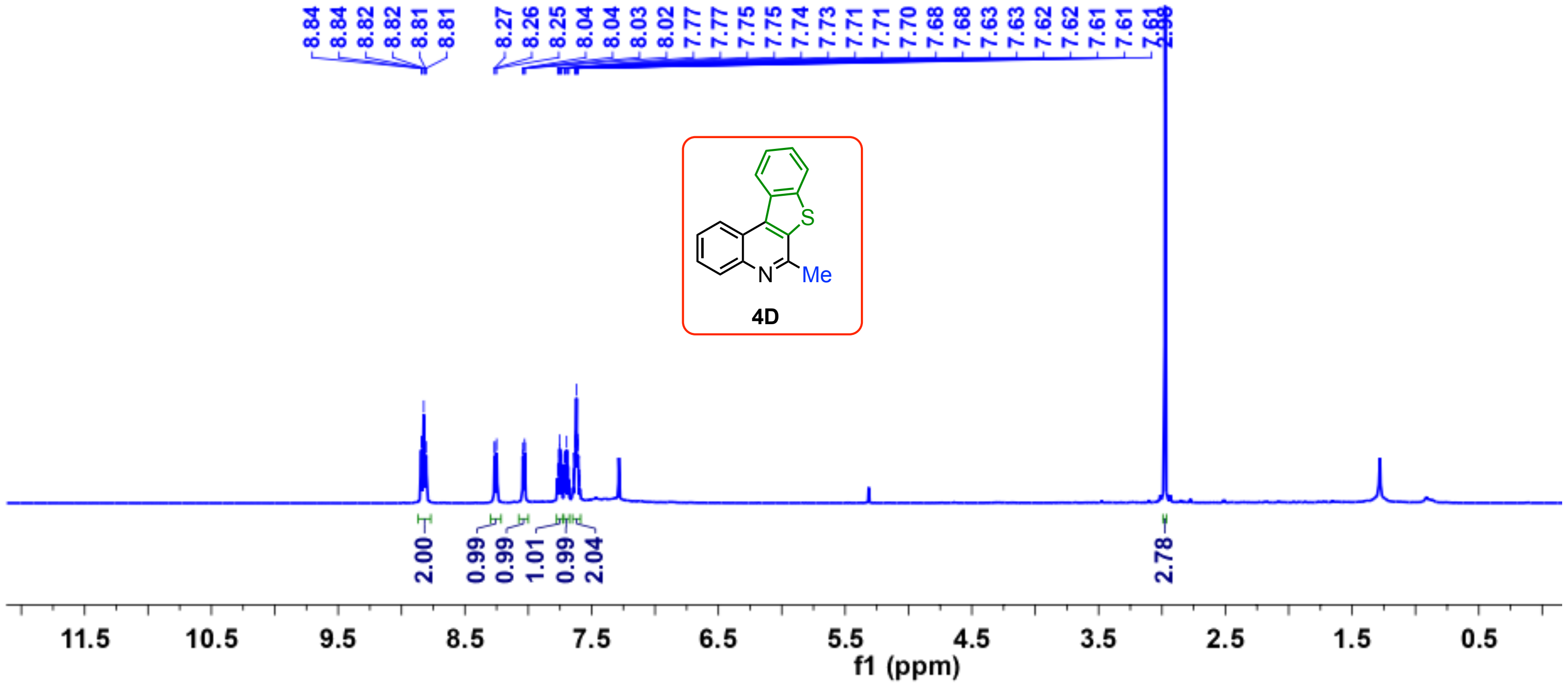

Figure S42: ^{1}H NMR of quinoline photoproduct **4D**.

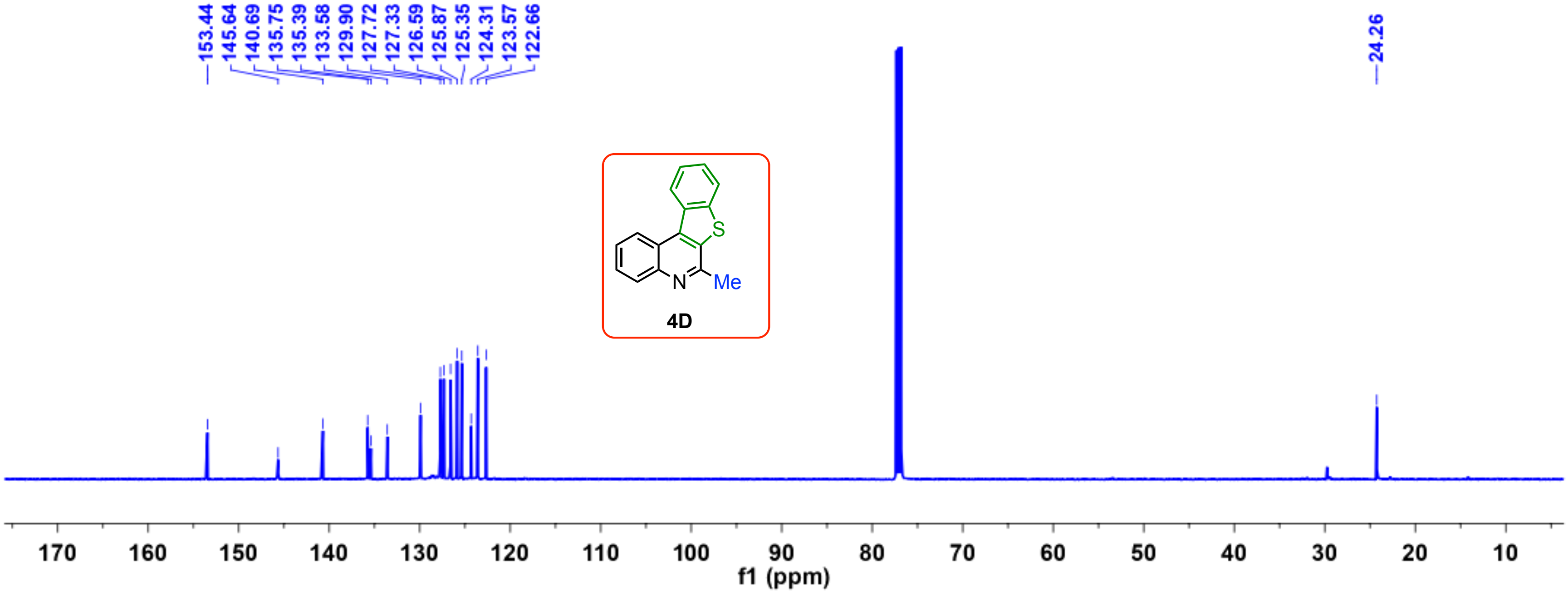

Figure S43: ^{13}C NMR of quinoline photoproduct **4D**.

HRMS-ESI (m/z) ([M + H]$^+$):

Calculated: 250.0690

Observed: 250.0697

|Δm| = 2.7 ppm

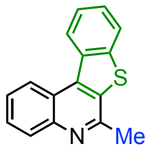

4D

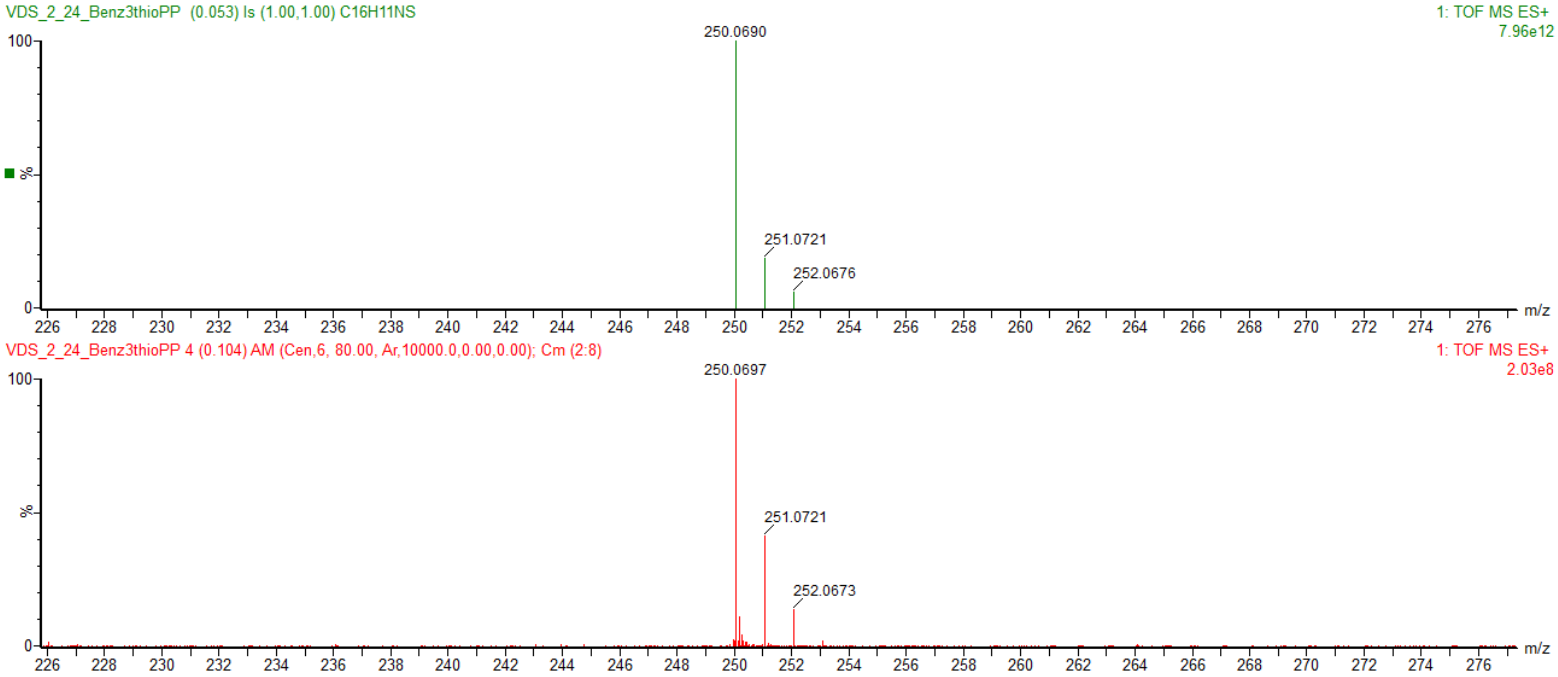

Figure S44: HRMS data of quinoline photoproduct **4D**.

17.5. Characterization of photoproduct **4E**.

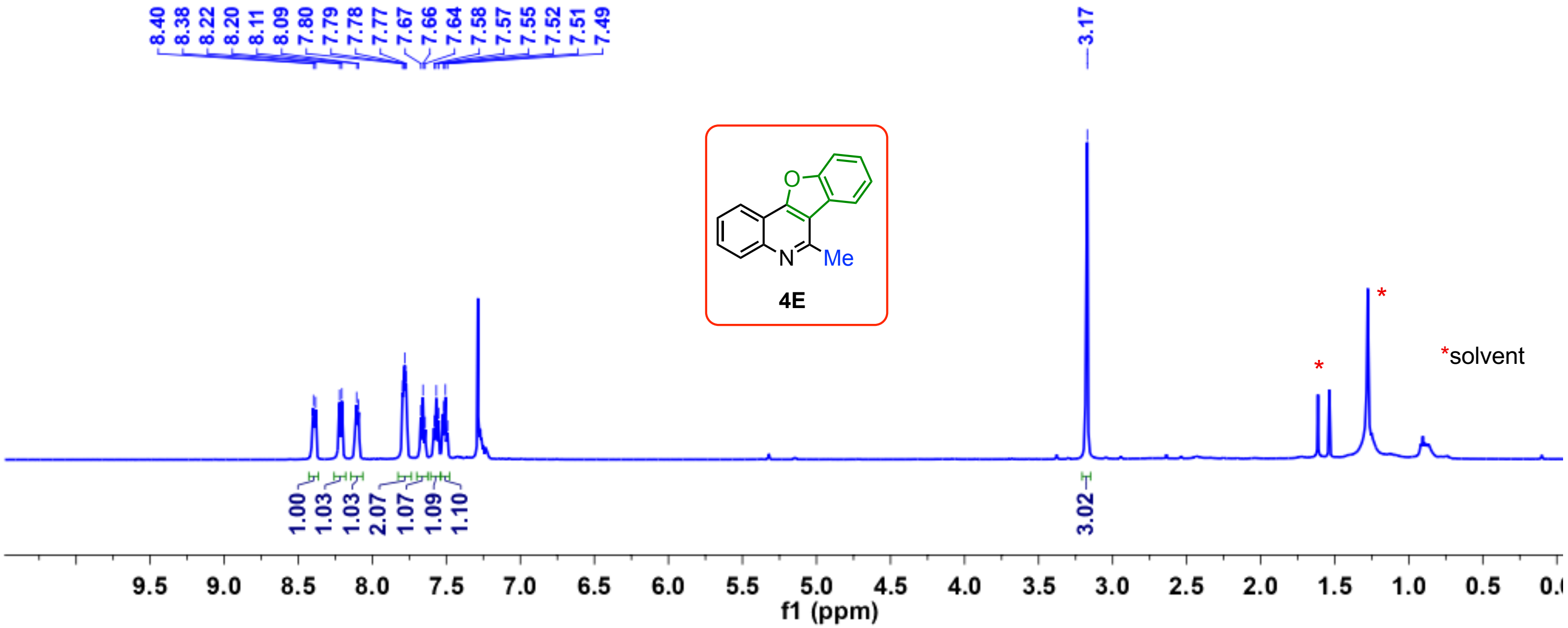

Figure S45: ^{1}H NMR of quinoline photoproduct **4E**.

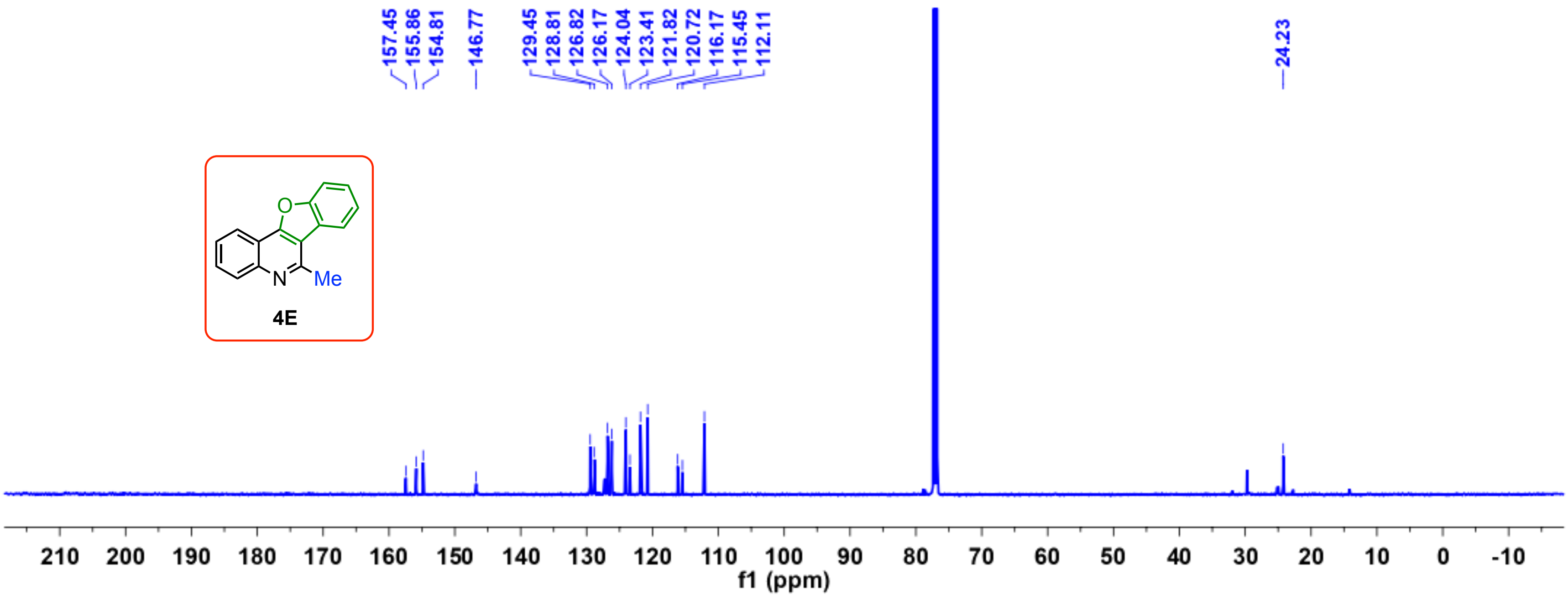

Figure S46: ^{13}C NMR of quinoline photoproduct **4E**.

HRMS-ESI (m/z) ([M + H]$^+$):

Calculated: 234.0919

Observed: 234.0916

|Δm| = 1.28 ppm

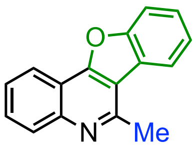

4E

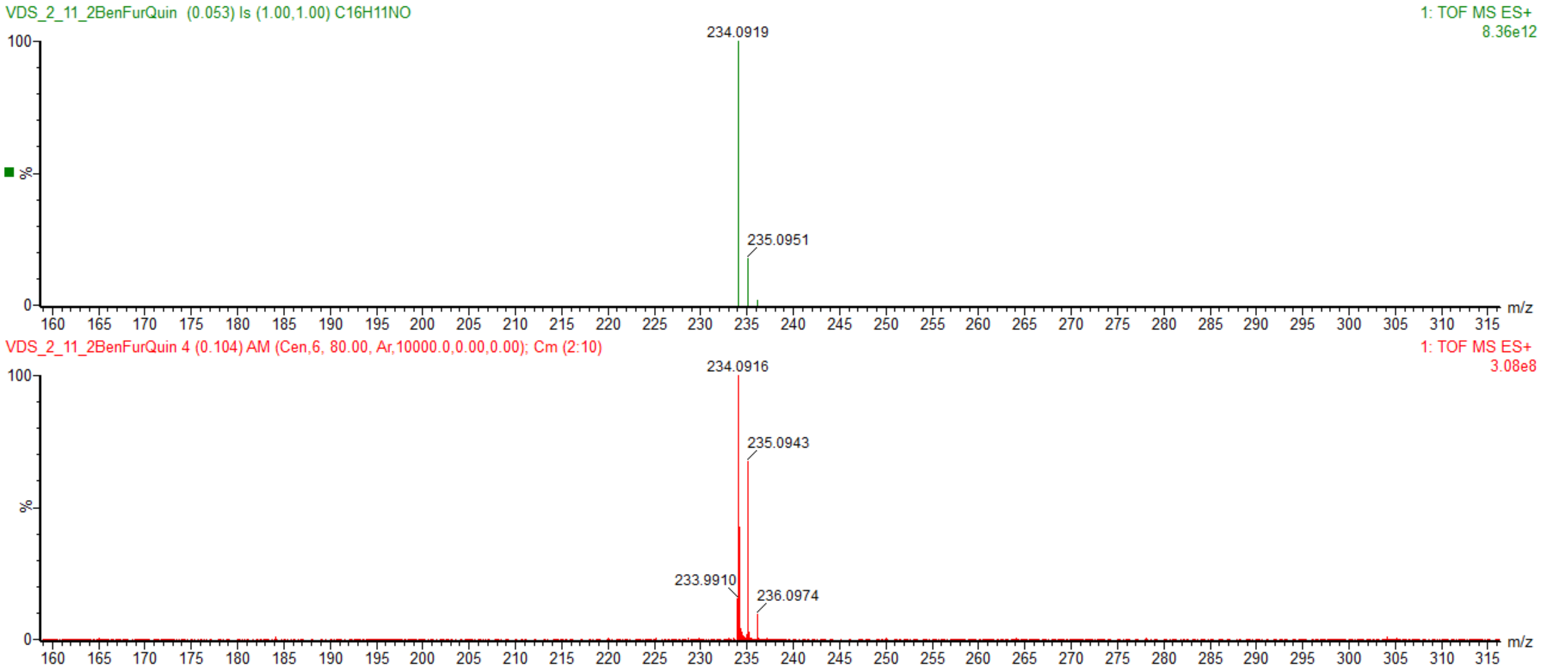

Figure S47: HRMS data of quinoline photoproduct **4E**.

17.6. Characterization of photoproduct **4F**.

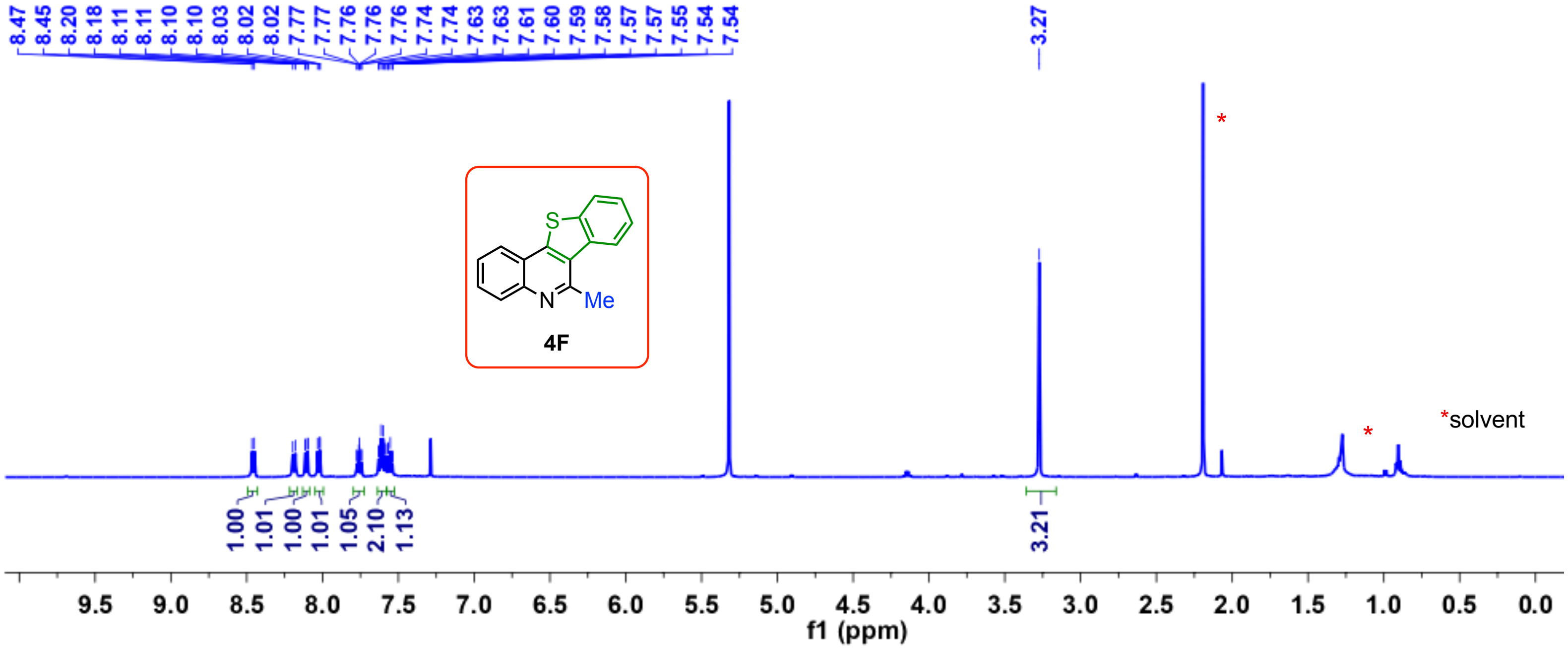

Figure S48: ^{1}H NMR of quinoline photoproduct **4F**.

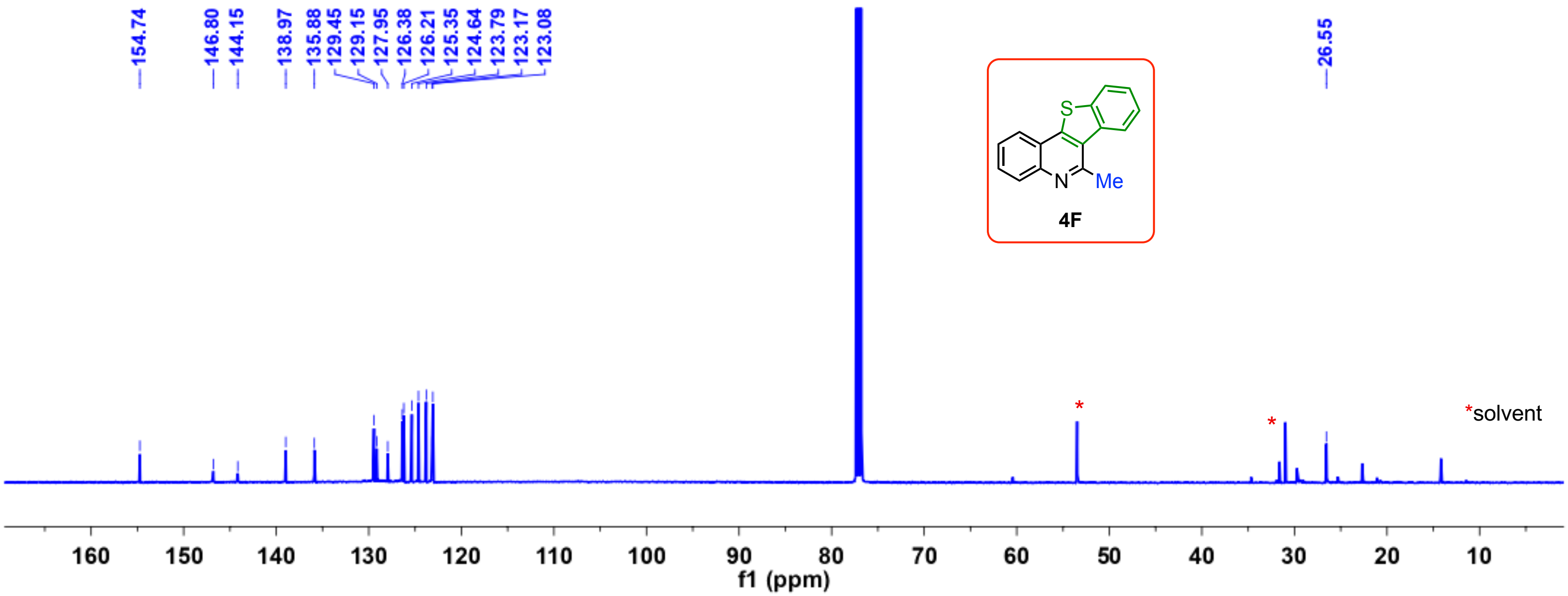

Figure S49: ^{13}C NMR of quinoline photoproduct **4F**.

HRMS-ESI (m/z) ([M + H]$^+$):

Calculated: 250.0690

Observed: 250.0697

|Δm| = 2.7 ppm

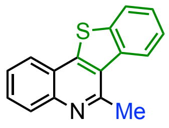

4F

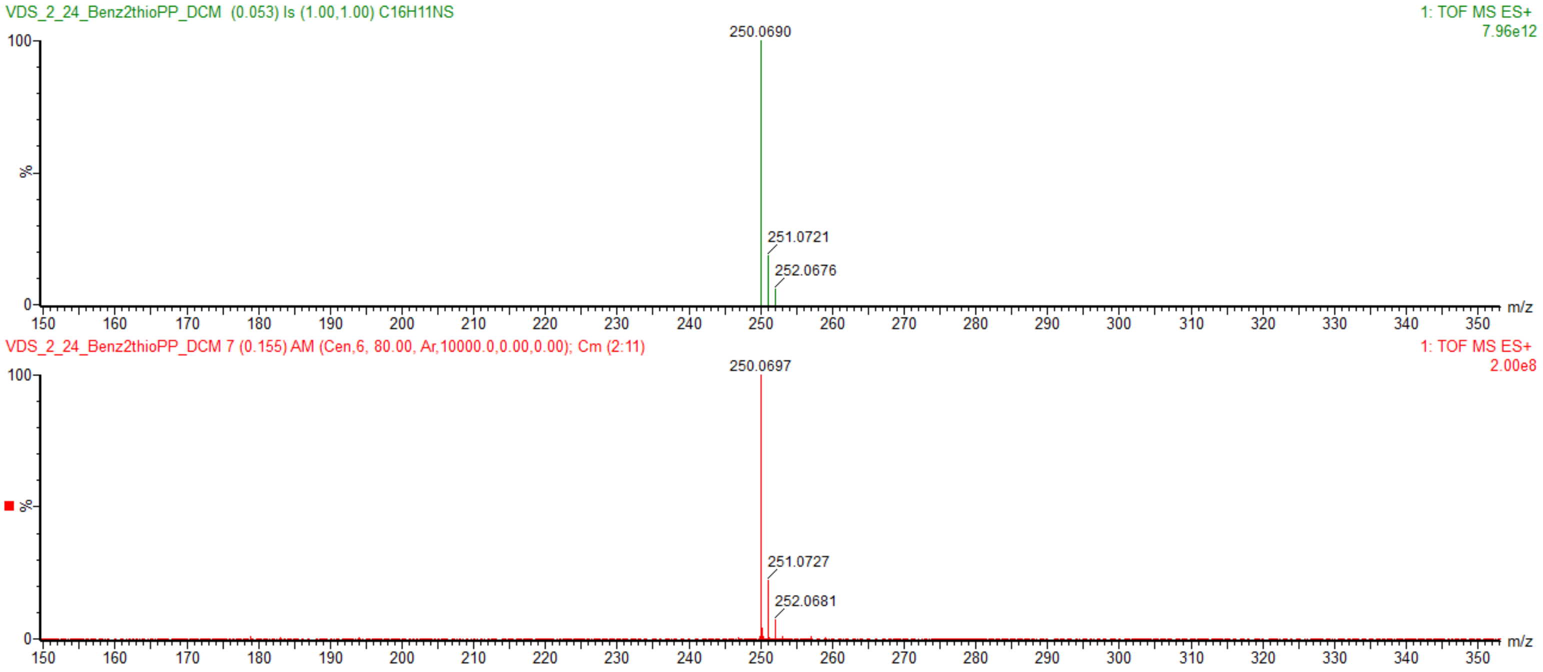

Figure S50: HRMS data of quinoline photoproduct **4F**.

17.7. Characterization of photoproduct **4G**.

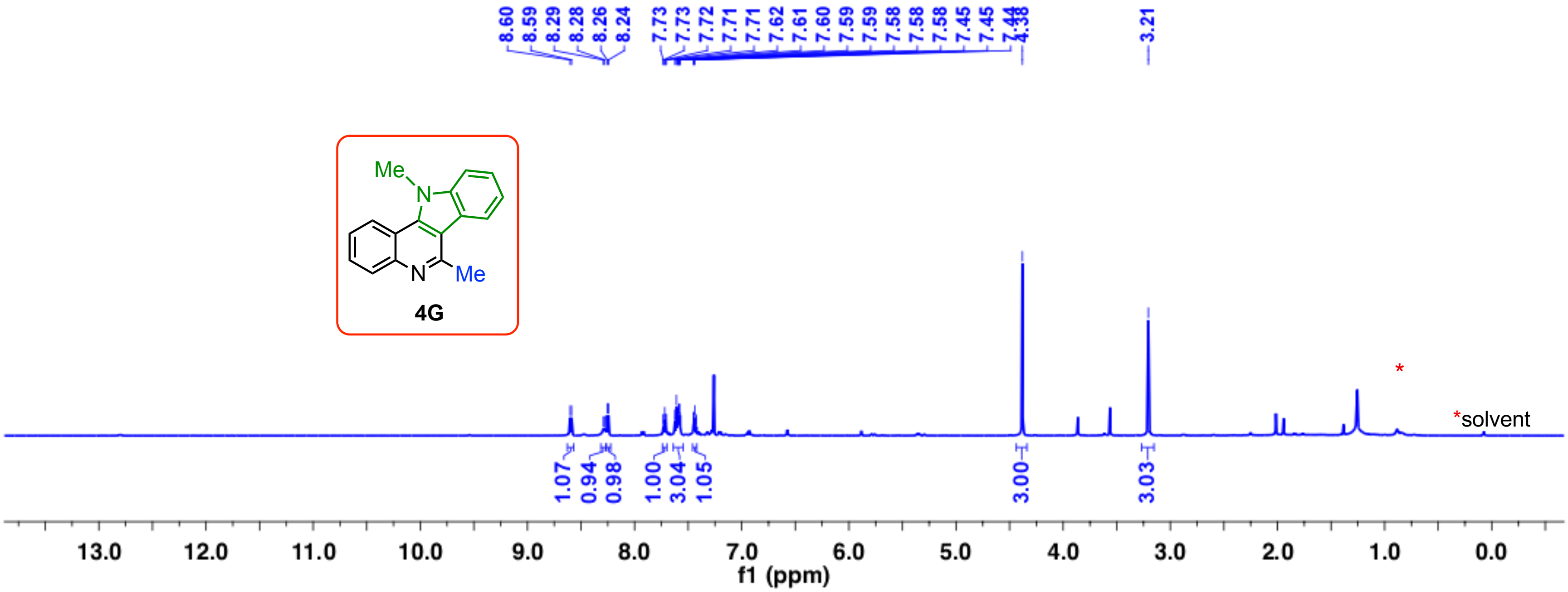

Figure S51: ^{1}H NMR of quinoline photoproduct **4G**.

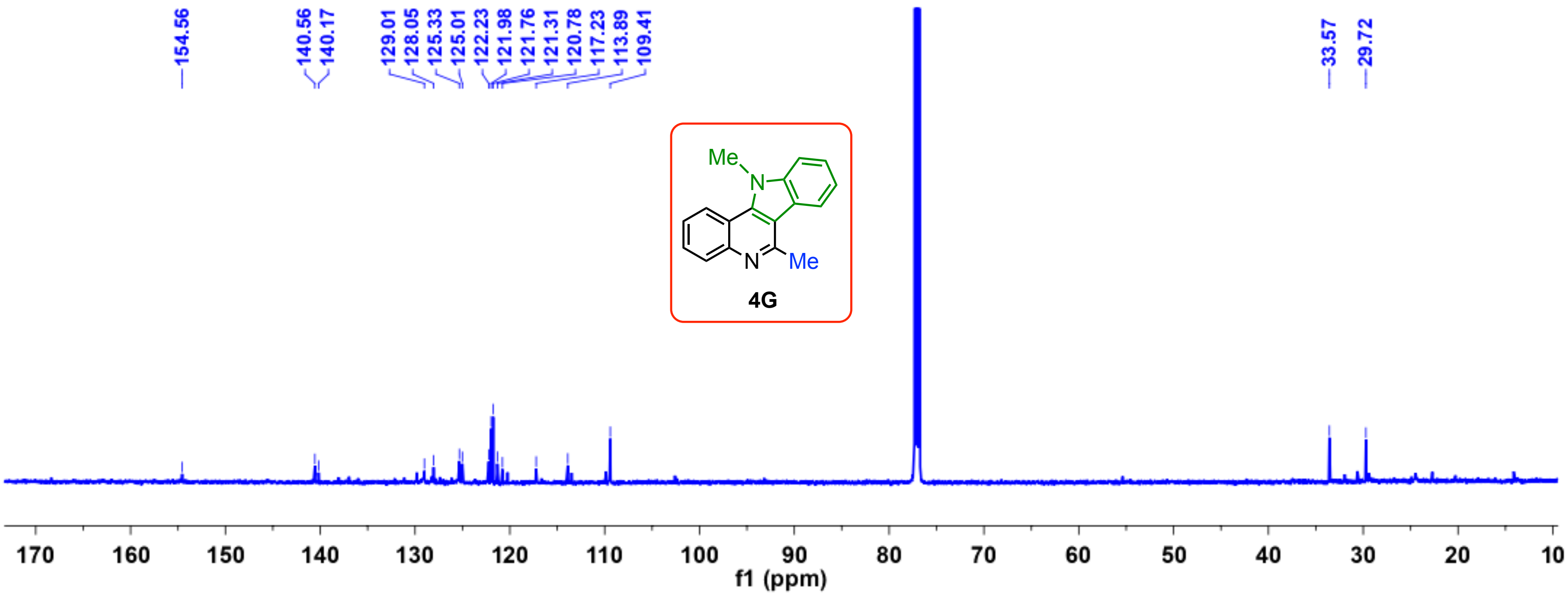

Figure S52: ^{13}C NMR of quinoline photoproduct **4G**.

HRMS-ESI (m/z) ([M + H]$^+$):

Calculated: 247.1235

Observed: 247.1246

|Δm| = 4.45 ppm

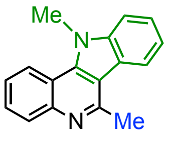

4G

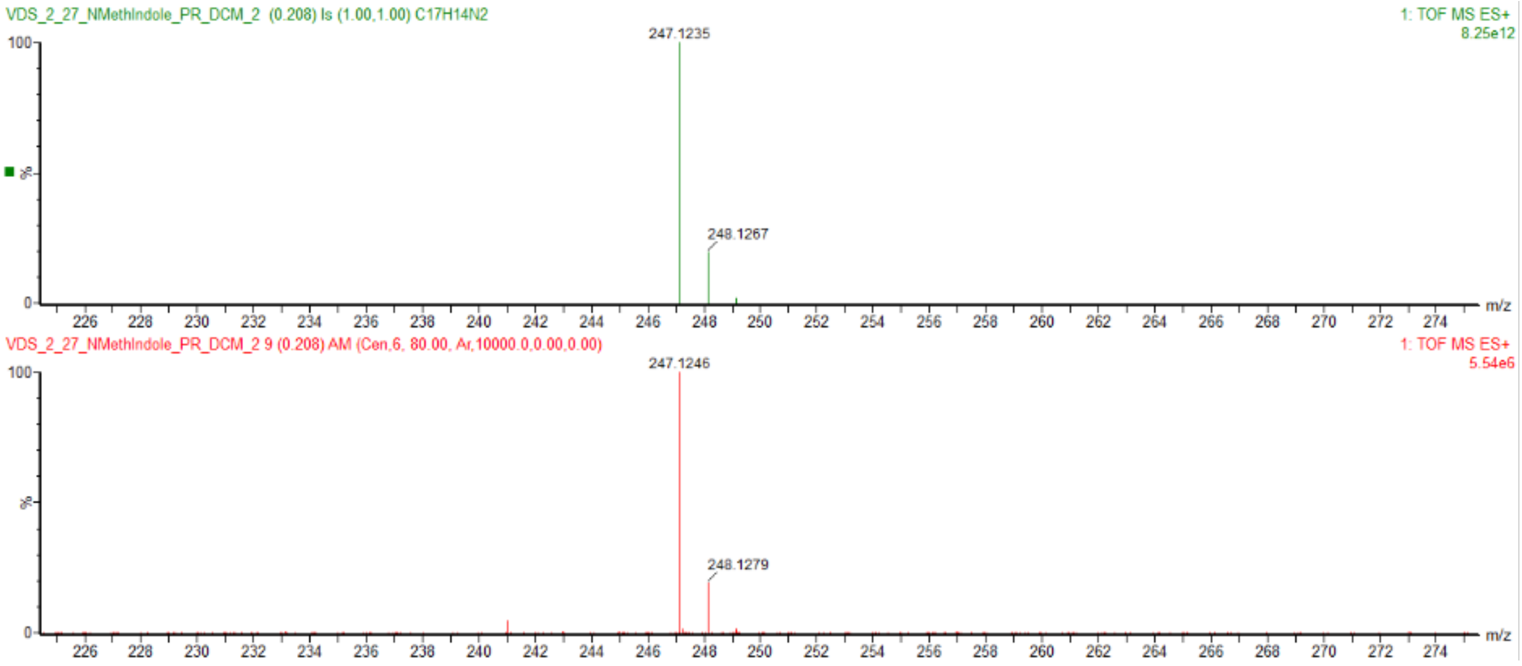

Figure S53: HRMS data of quinoline photoproduct **4G**.

17.1. Characterization of photoproduct **4H**.

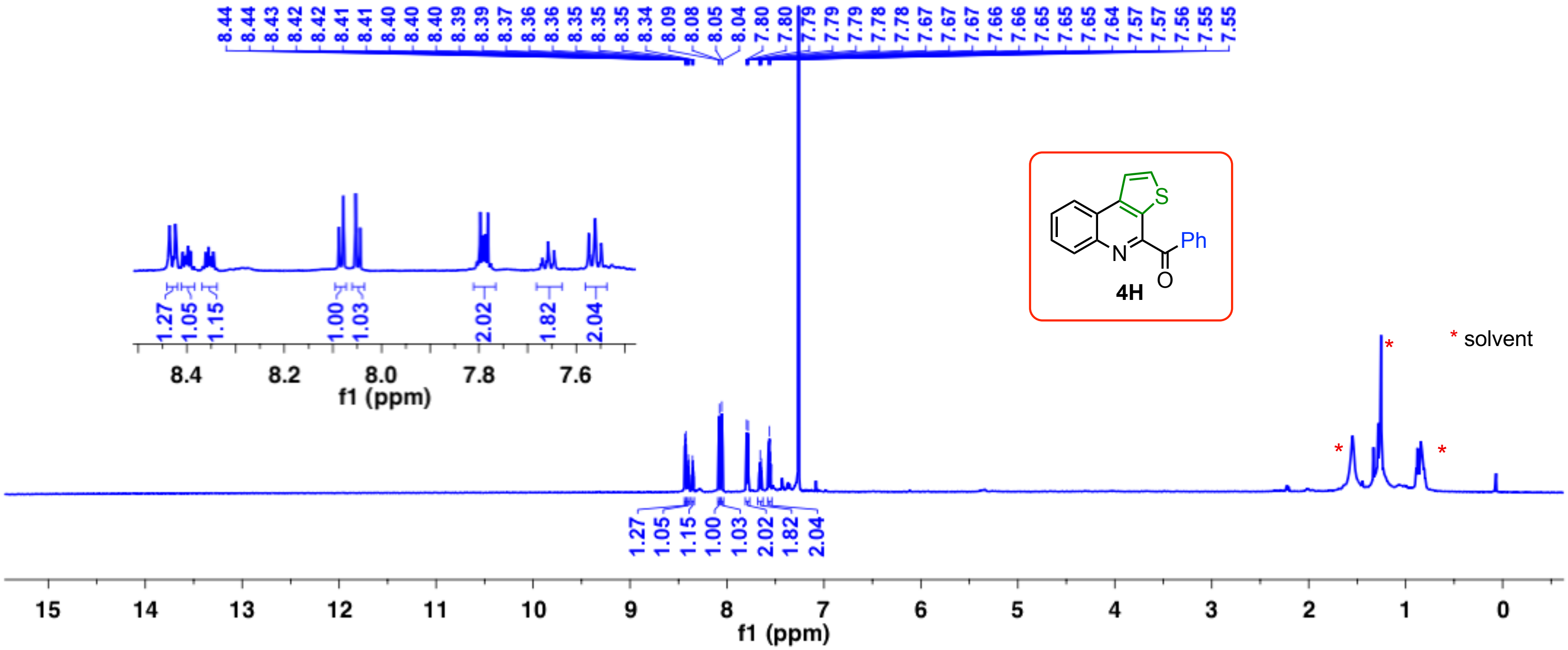

Figure S54: ^{1}H NMR of quinoline photoproduct **4H**.

HRMS-ESI (m/z) ([M + H]$^+$):

Calculated: 290.0640

Observed: 290.0624

|Δm| = 5.5 ppm

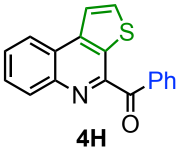

4H

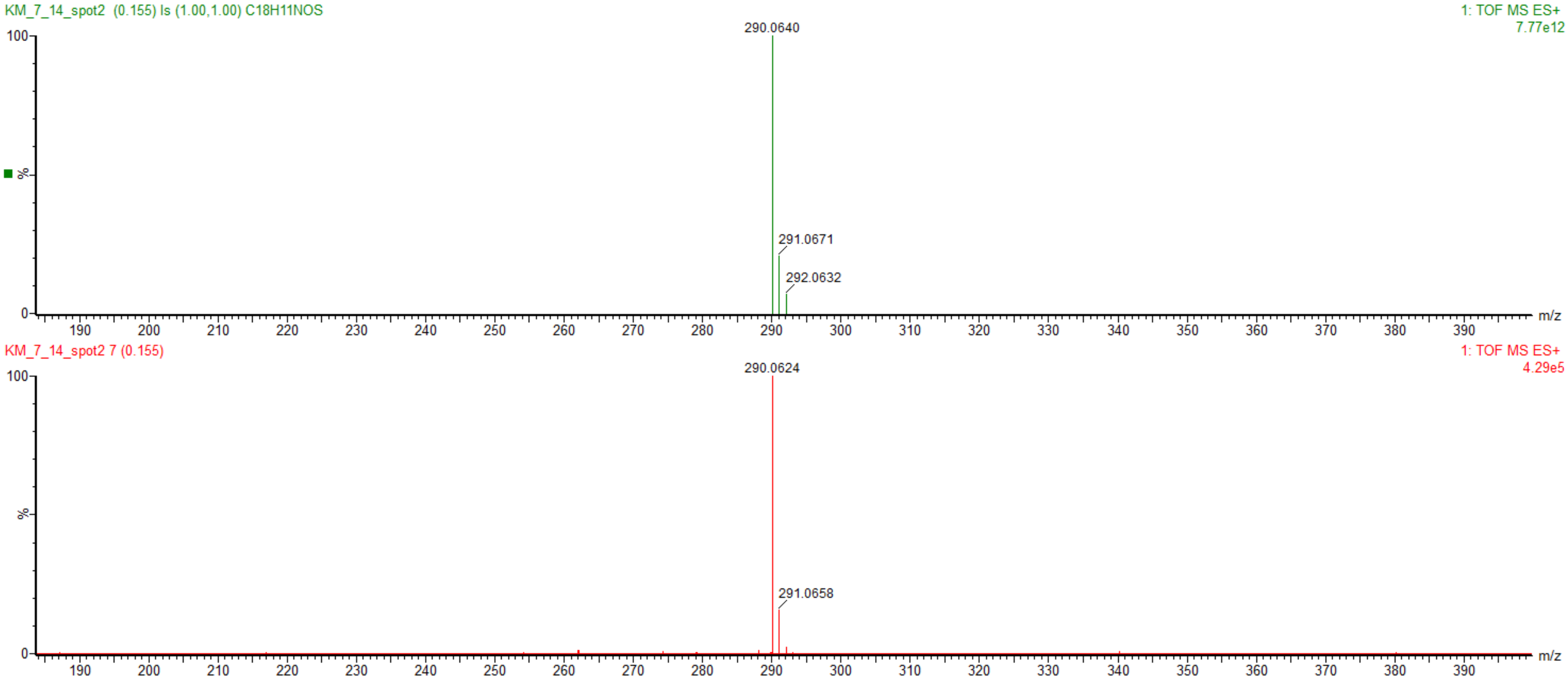

Figure S55: HRMS data of quinoline photoproduct **4H**

18. Spectroscopic characterization of precursors used for synthesis.

18.1. Characterization of 1,3-diketone **1b**.

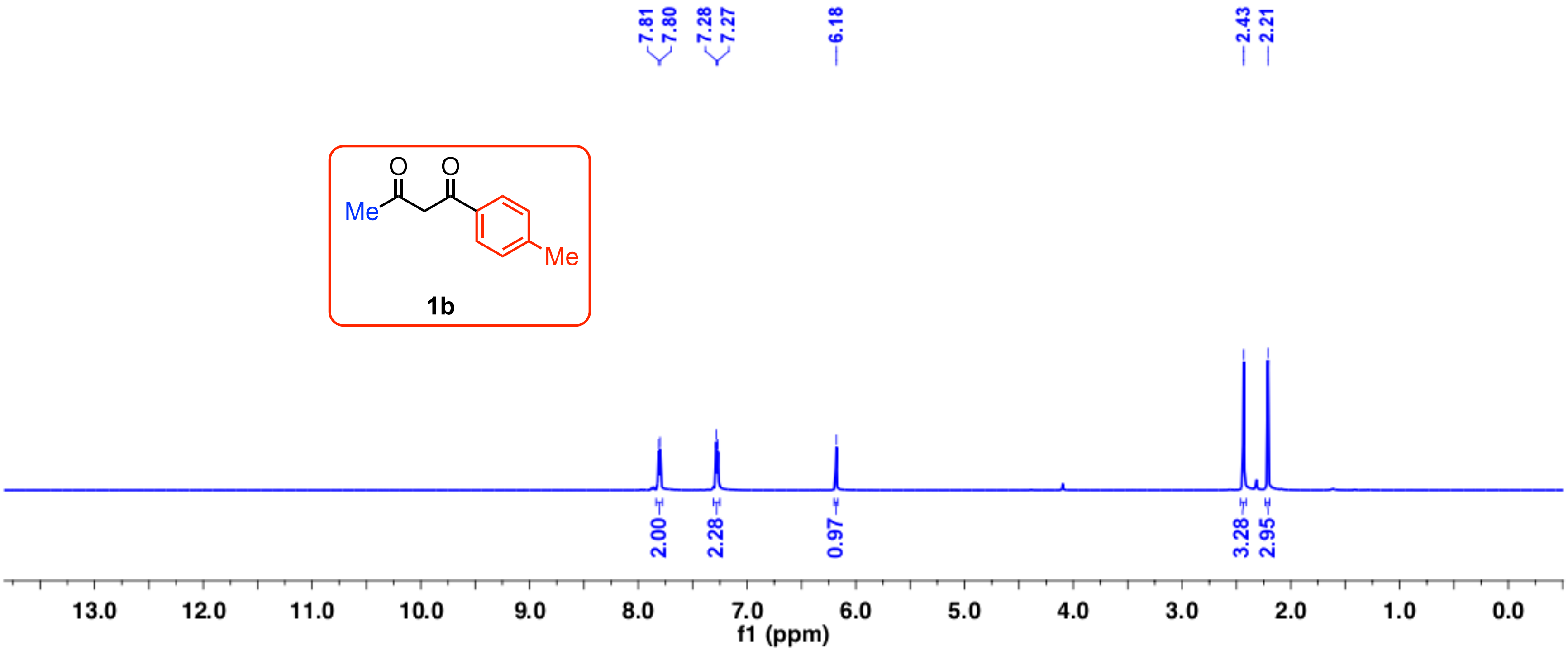

Figure S56: ^{1}H NMR of 1,3-diketone **1b**.

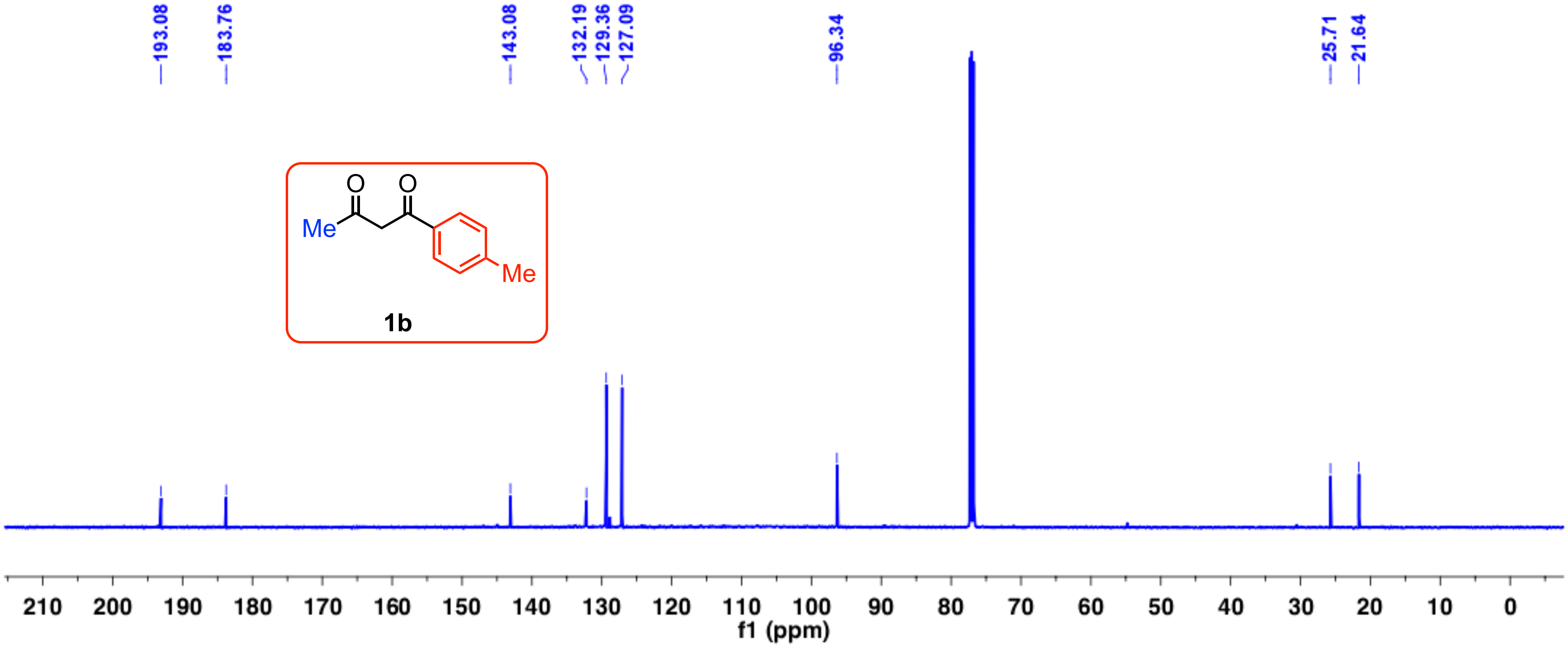

Figure S57: ^{13}C NMR of 1,3-diketone **1b**.

18.2. Characterization of 1,3-diketone **1c**.

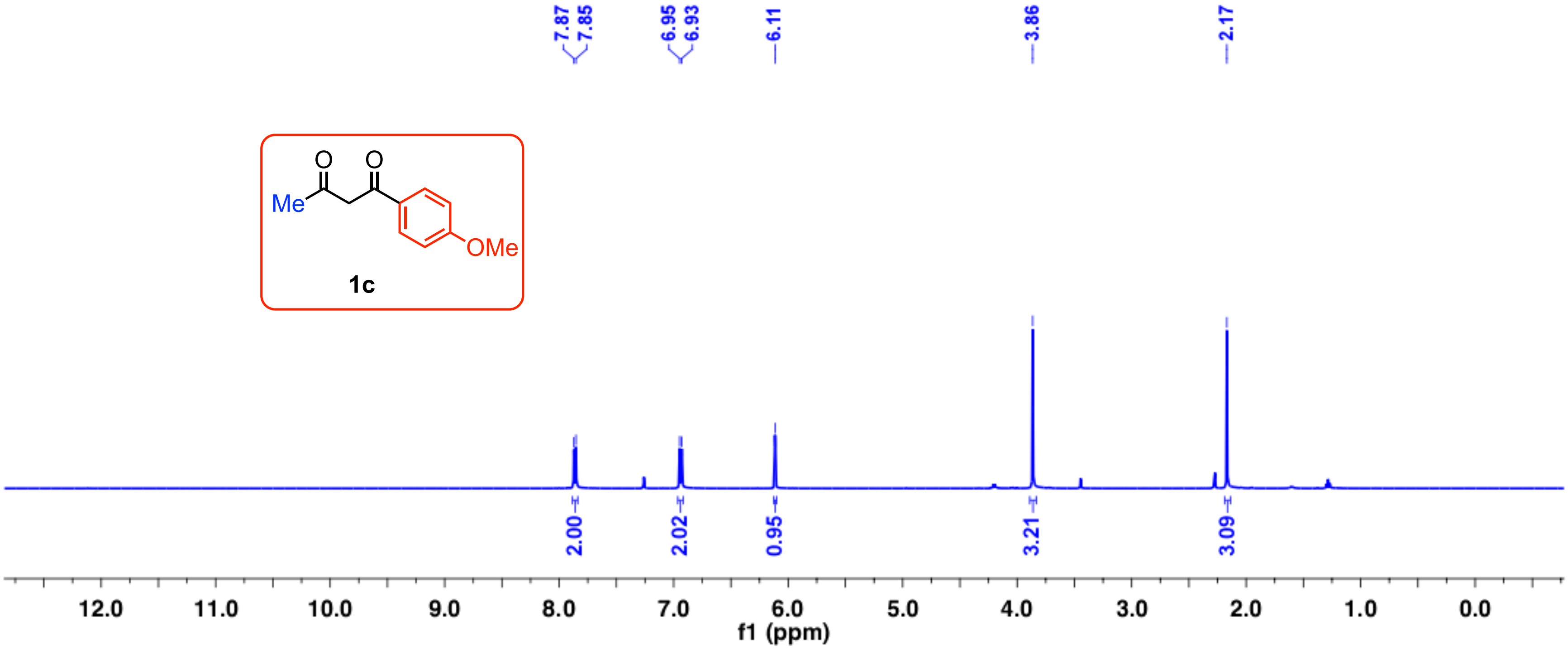

Figure S58: ^{1}H NMR of 1,3-diketone **1c**.

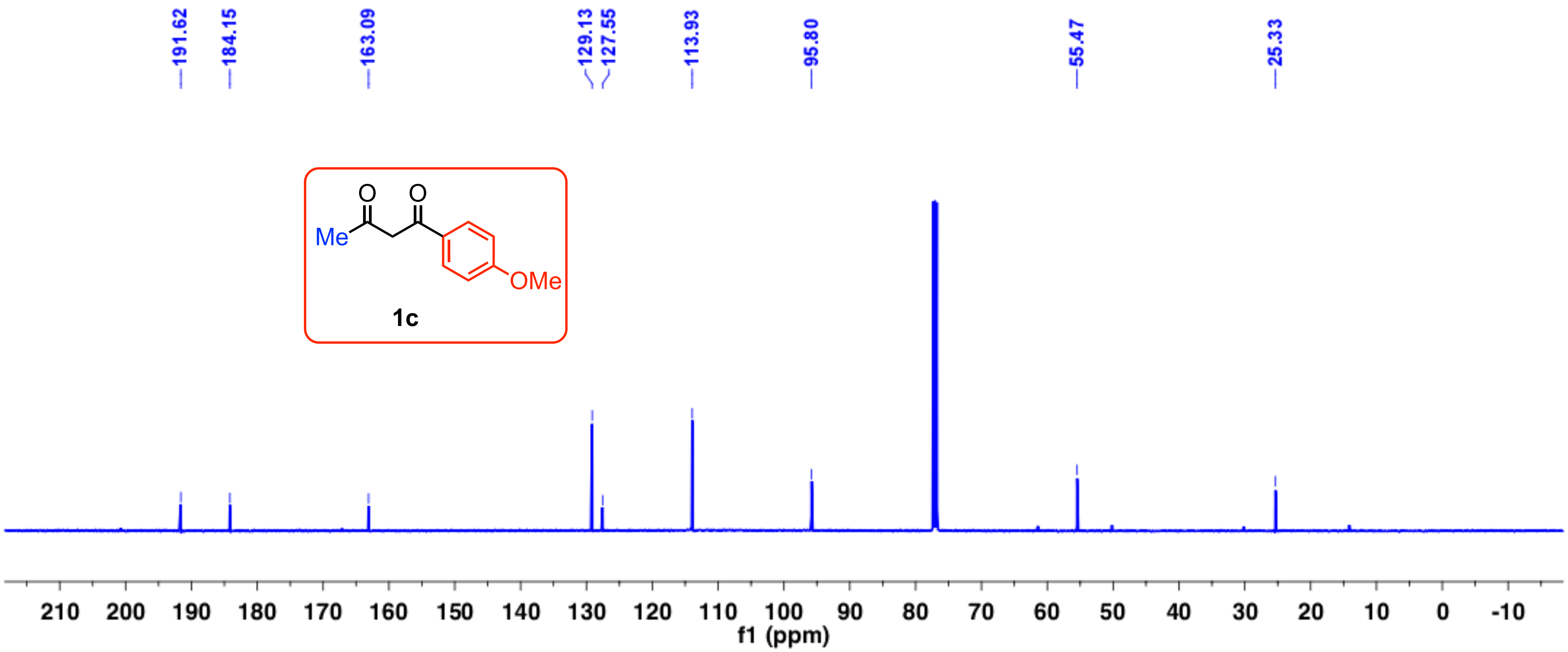

Figure S59: ^{13}C NMR of 1,3-diketone **1c**.

18.3. Characterization of 1,3-diketone **1d**.

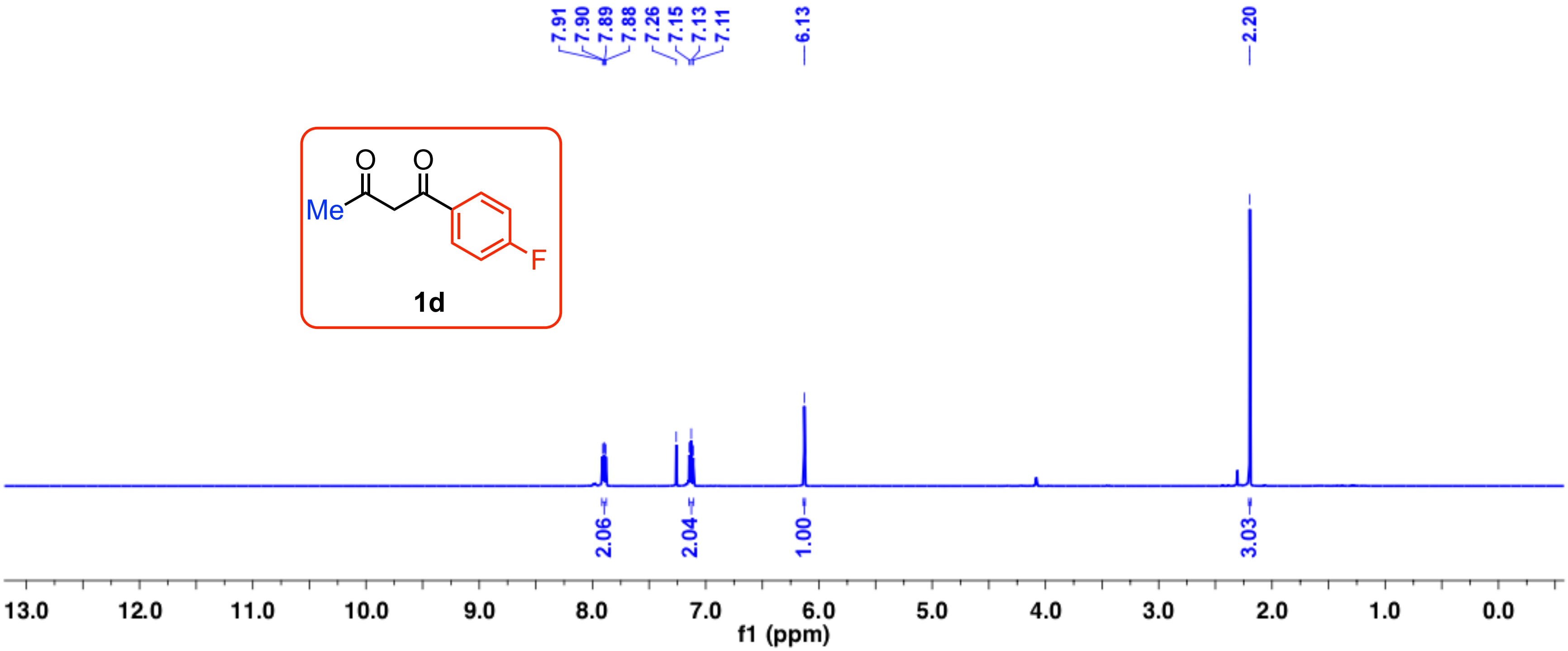

Figure S60: ^{1}H NMR of 1,3-diketone **1d**.

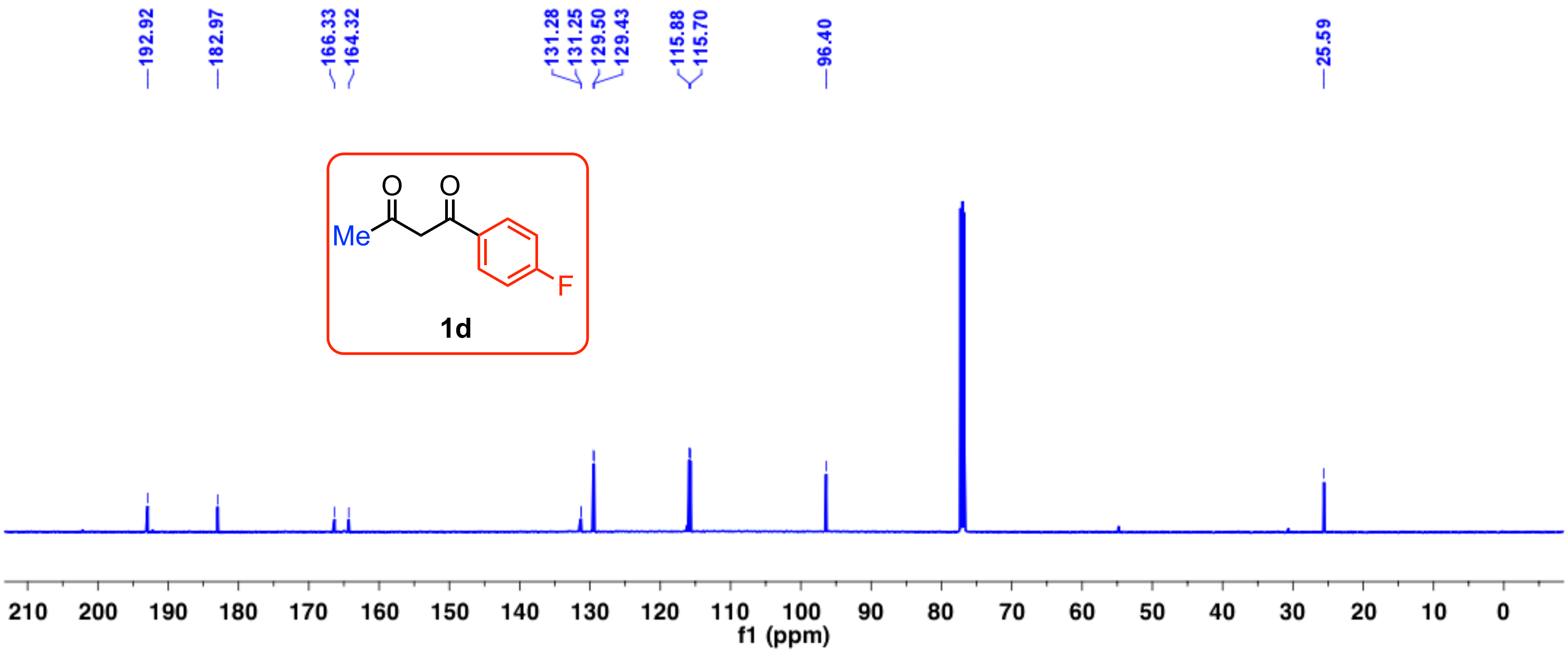

Figure S61: ^{13}C NMR of 1,3-diketone **1d**.

18.4. Characterization of 1,3-diketone **1e**.

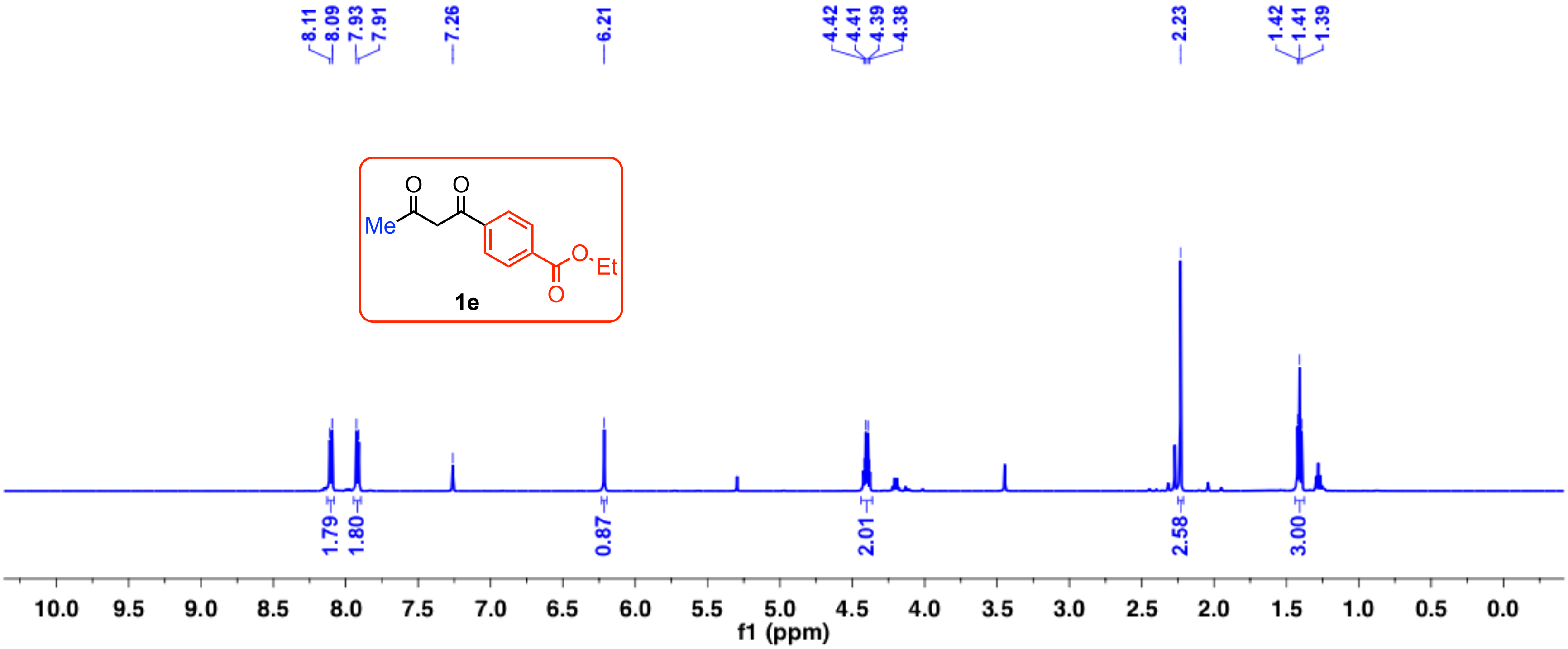

Figure S62: ^{1}H NMR of 1,3-diketone **1e**.

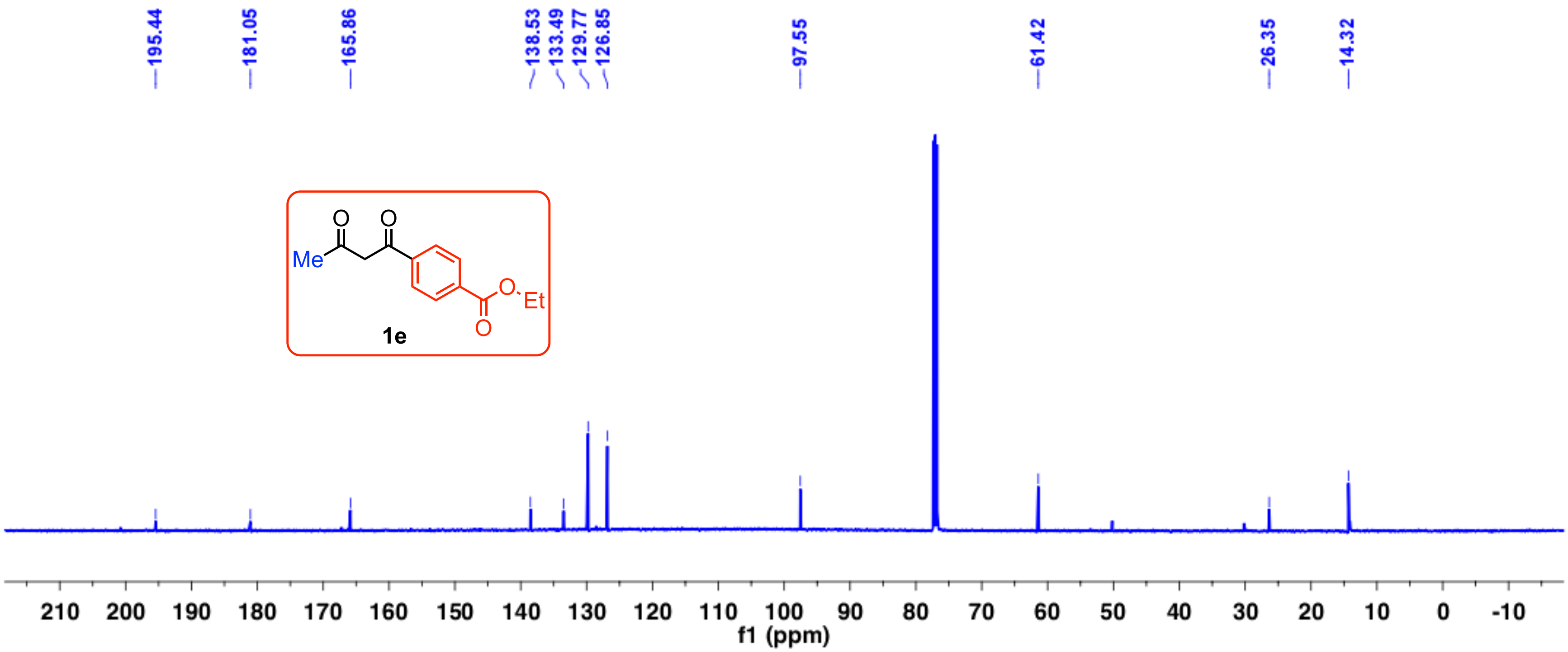

Figure S63: ^{13}C NMR of 1,3-diketone **1e**.

18.5. Characterization of 1,3-diketone **1f**.

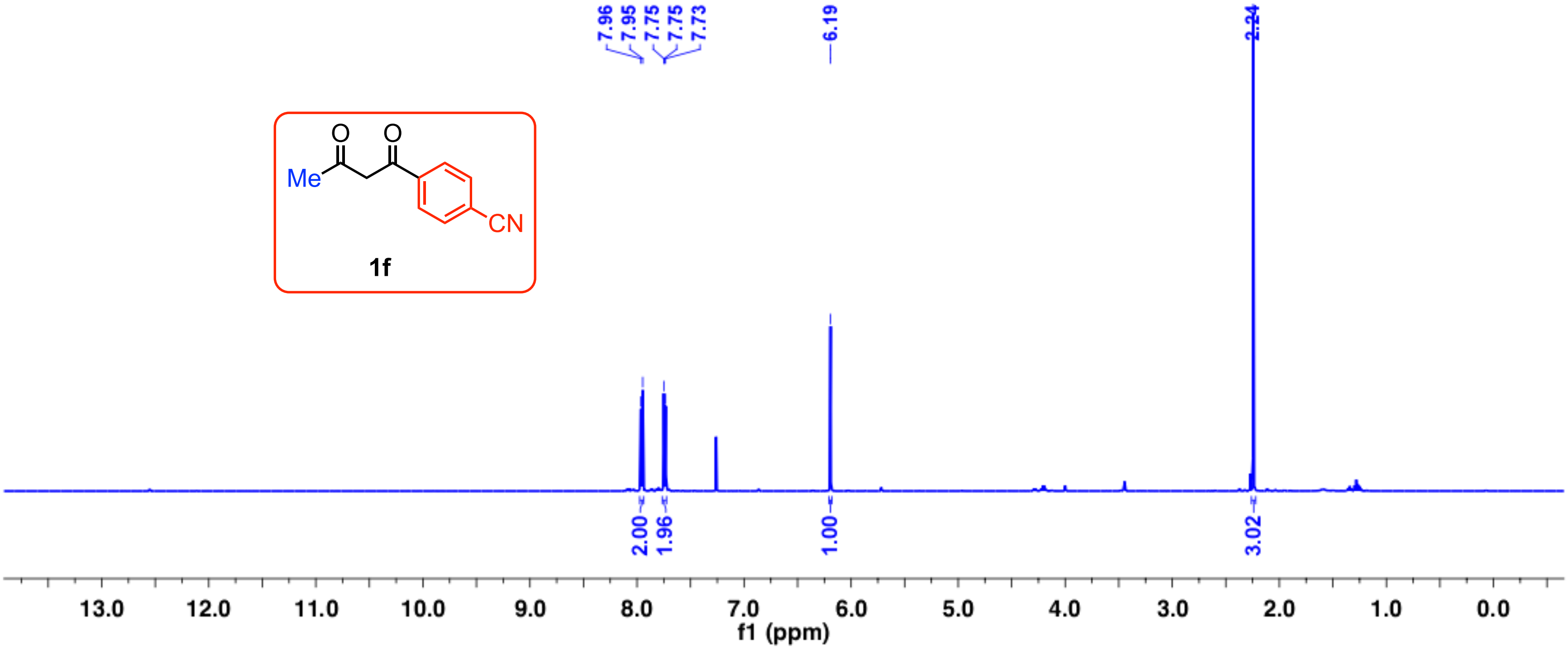

Figure S64: ^{1}H NMR of 1,3-diketone **1f** .

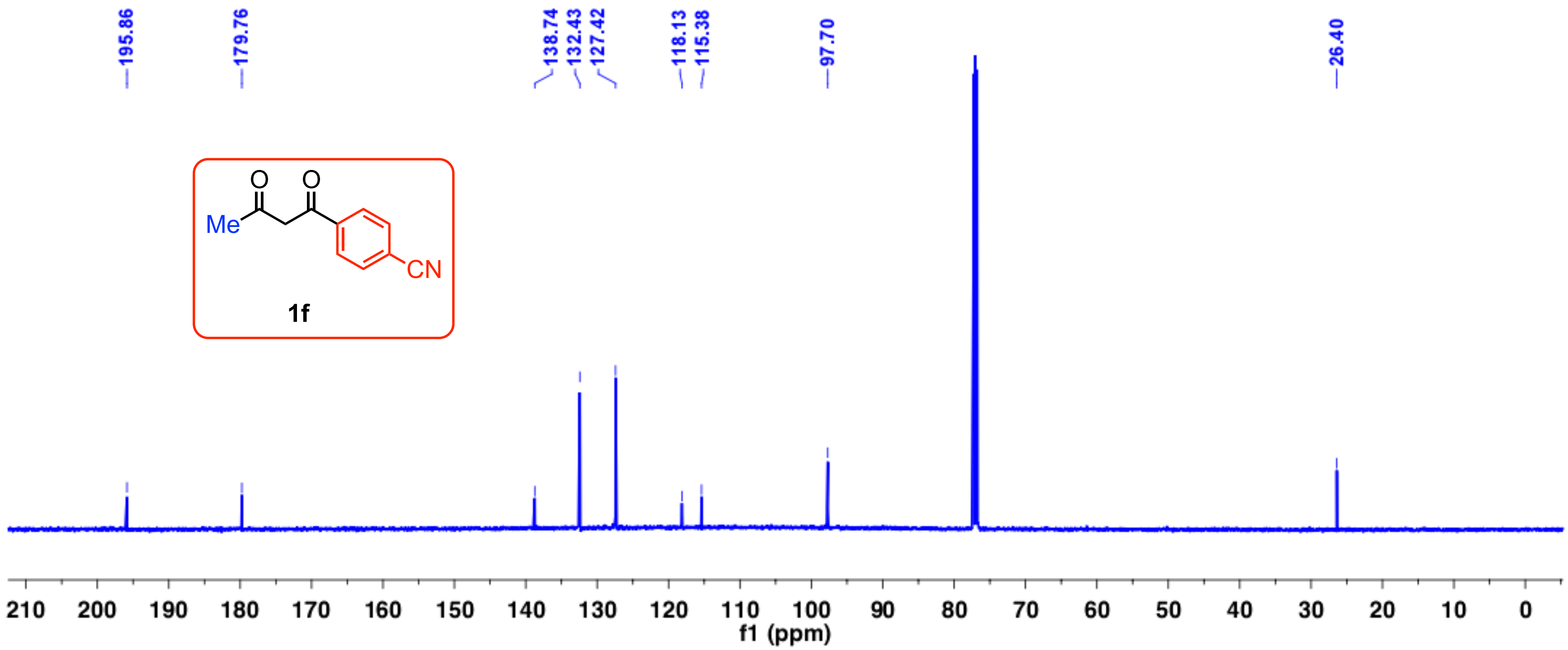

Figure S65: ^{13}C NMR of 1,3-diketone **1f** .

18.6. Characterization of 1,3-diketone **1g**.

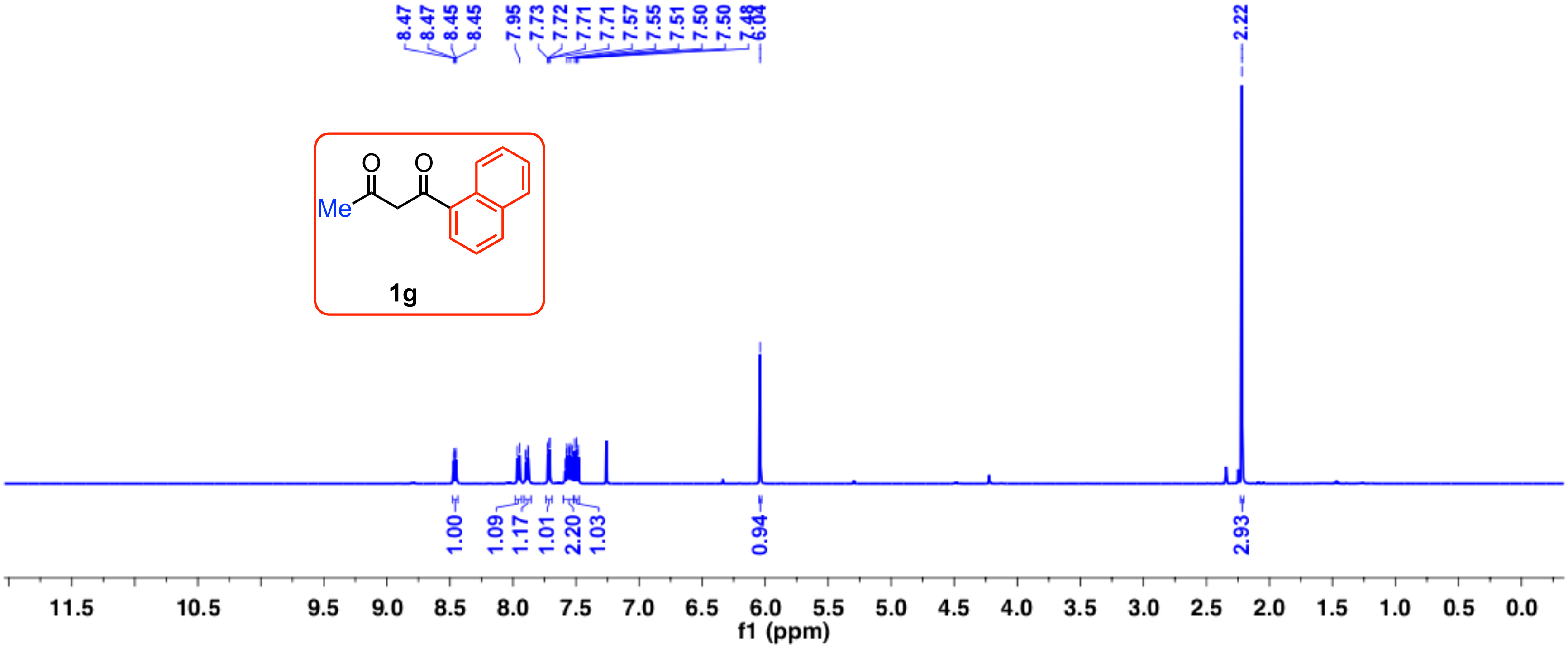

Figure S66: ^{1}H NMR of 1,3-diketone **1g**.

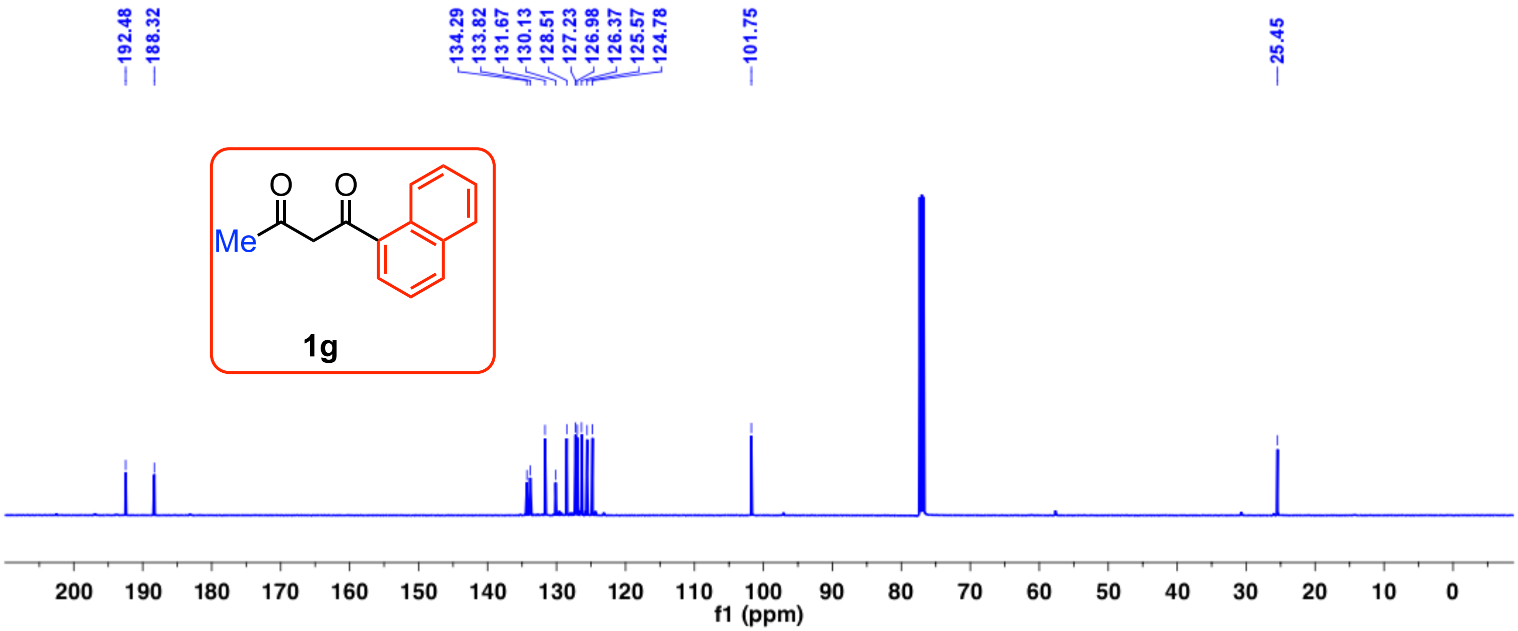

Figure S67: ^{13}C NMR of 1,3-diketone **1g**.

18.7. Characterization of 1,3-diketone **1h**.

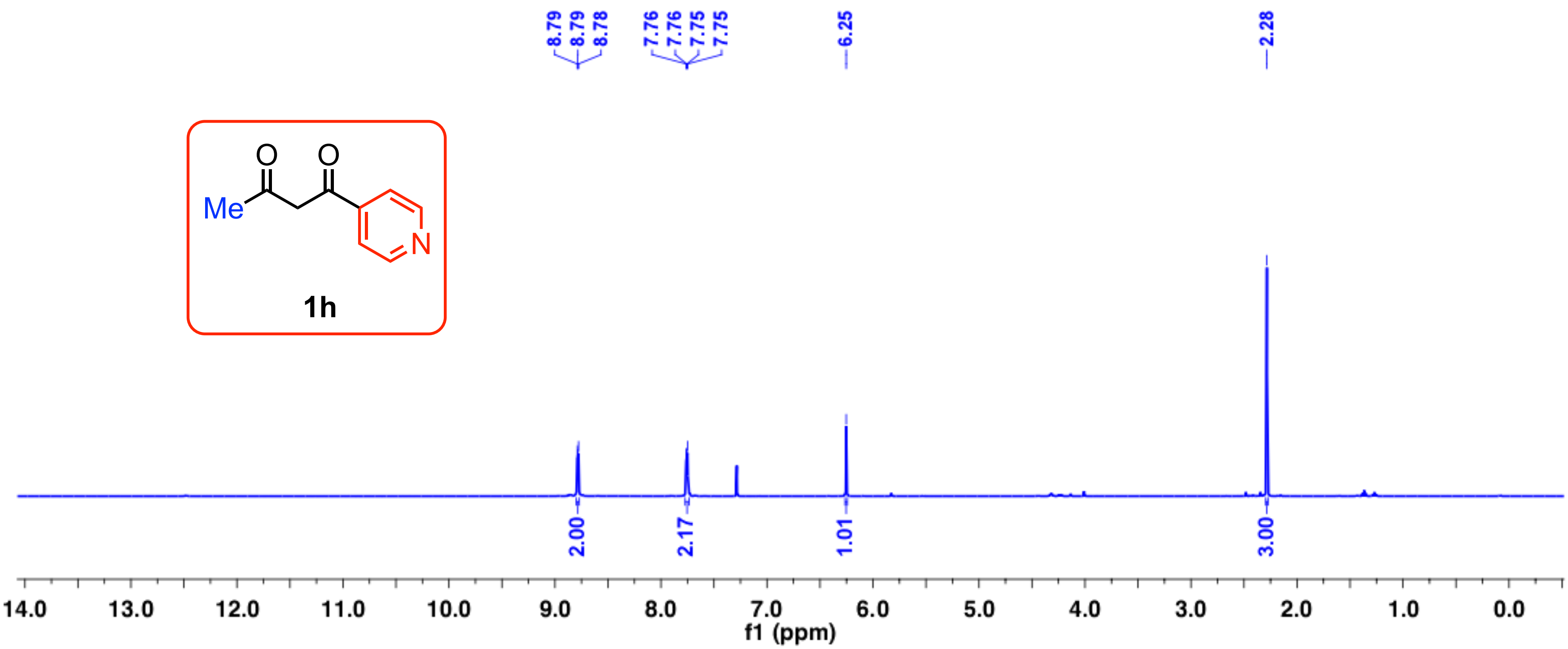

Figure S68: ^{1}H NMR of 1,3-diketone **1h** .

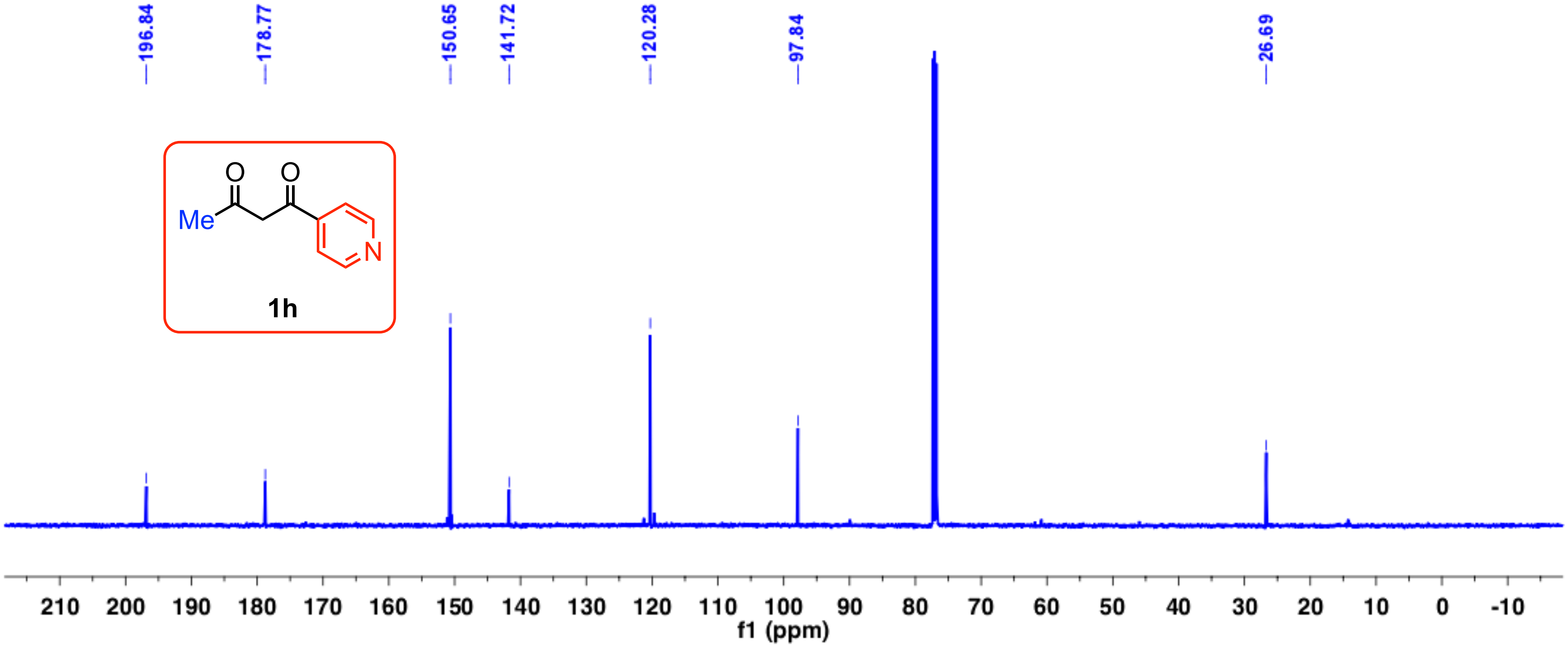

Figure S69: ^{13}C NMR of 1,3-diketone **1h**.

18.8. Characterization of 1,3-diketone **1i**.

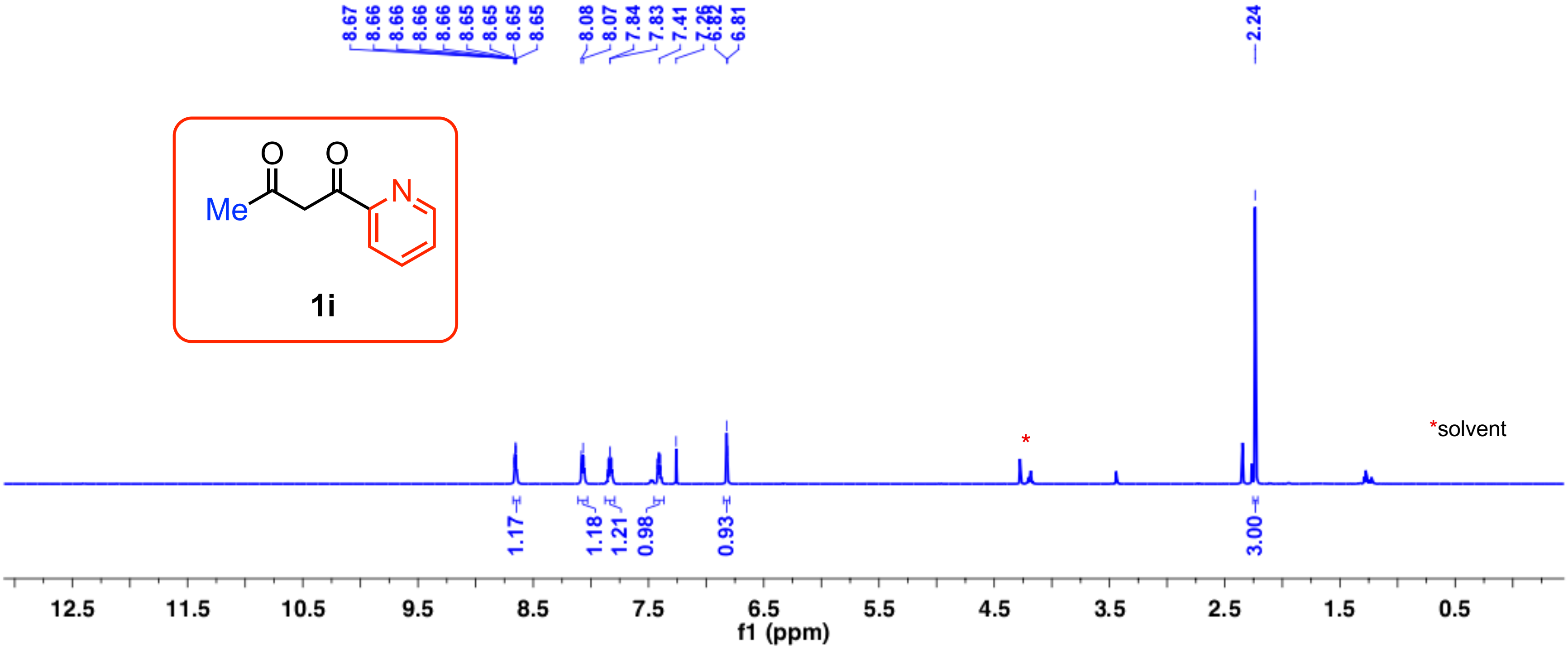

Figure S70: ^{1}H NMR of 1,3-diketone **1i**.

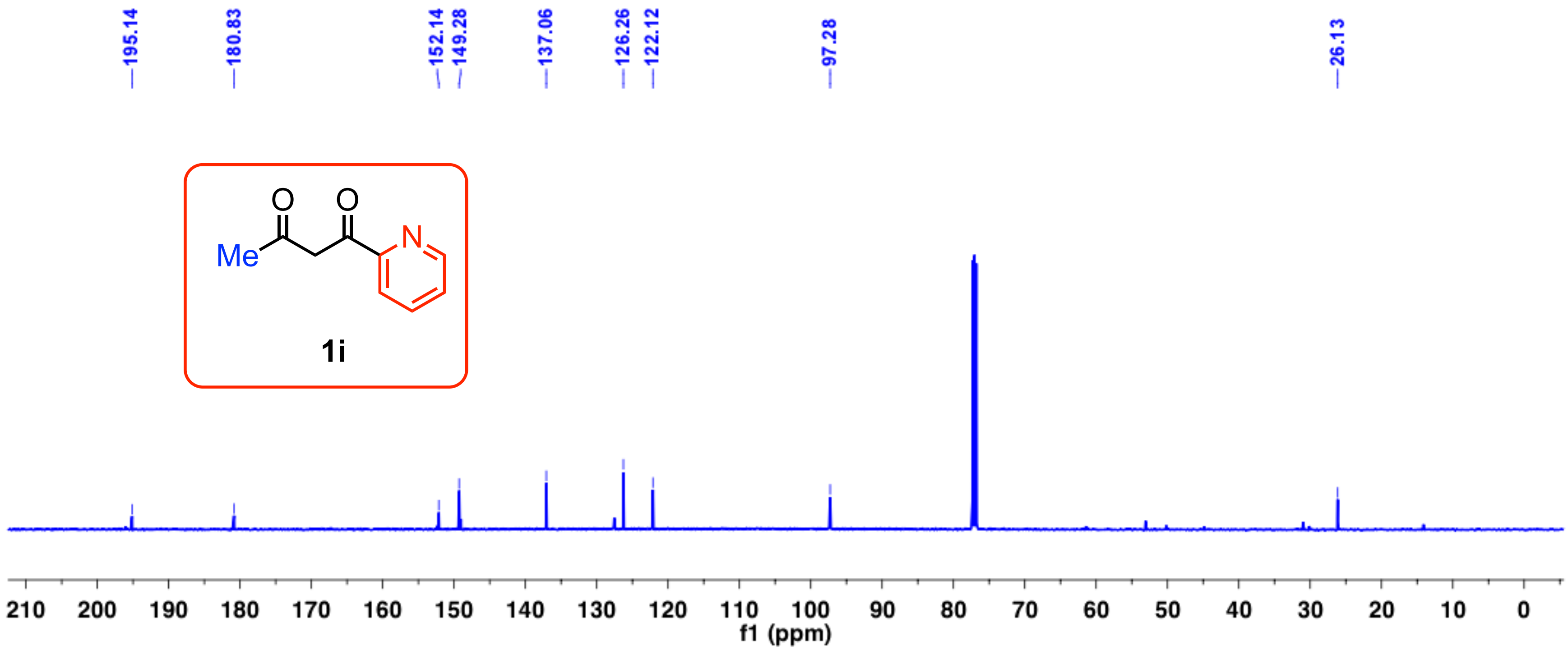

Figure S71: ^{13}C NMR of 1,3-diketone **1i**.

18.9. Characterization of 1,3-diketone **8**.

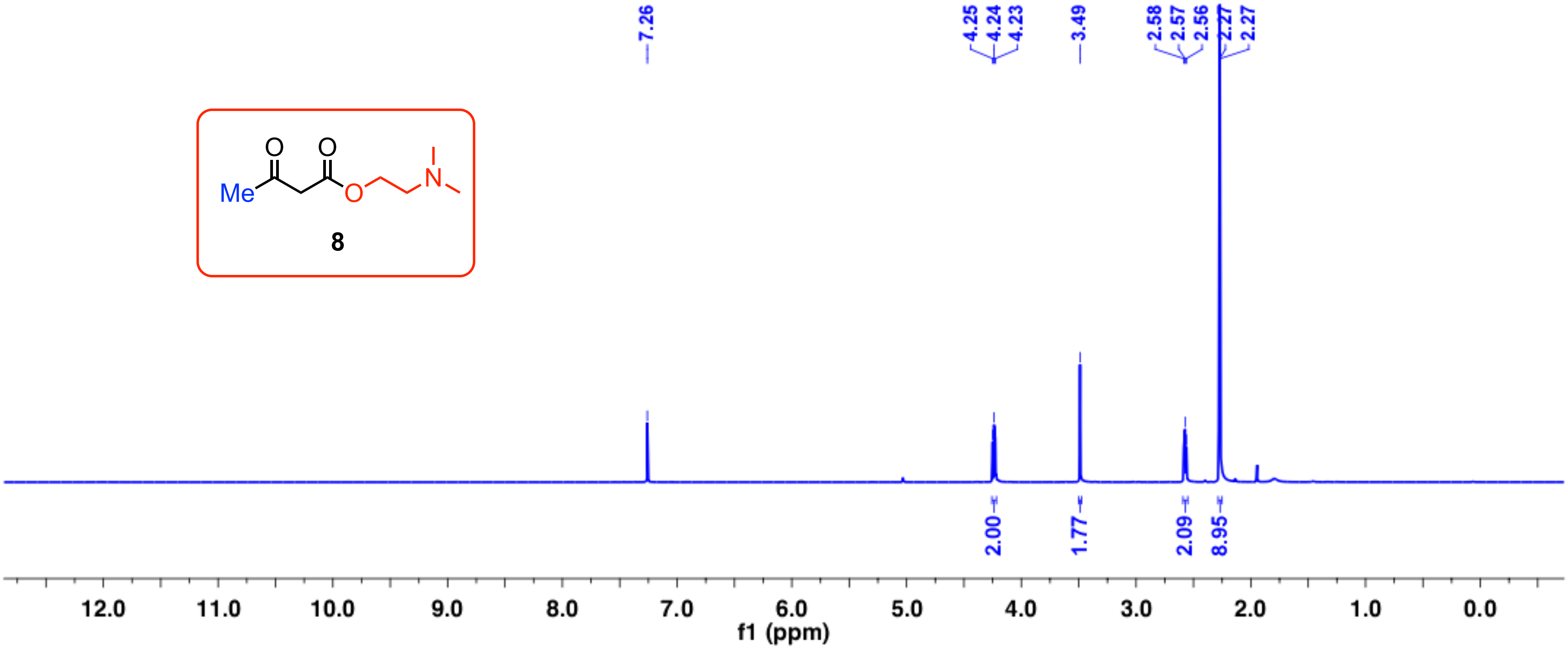

Figure S72: ^{1}H NMR of 1,3-diketone **8**.

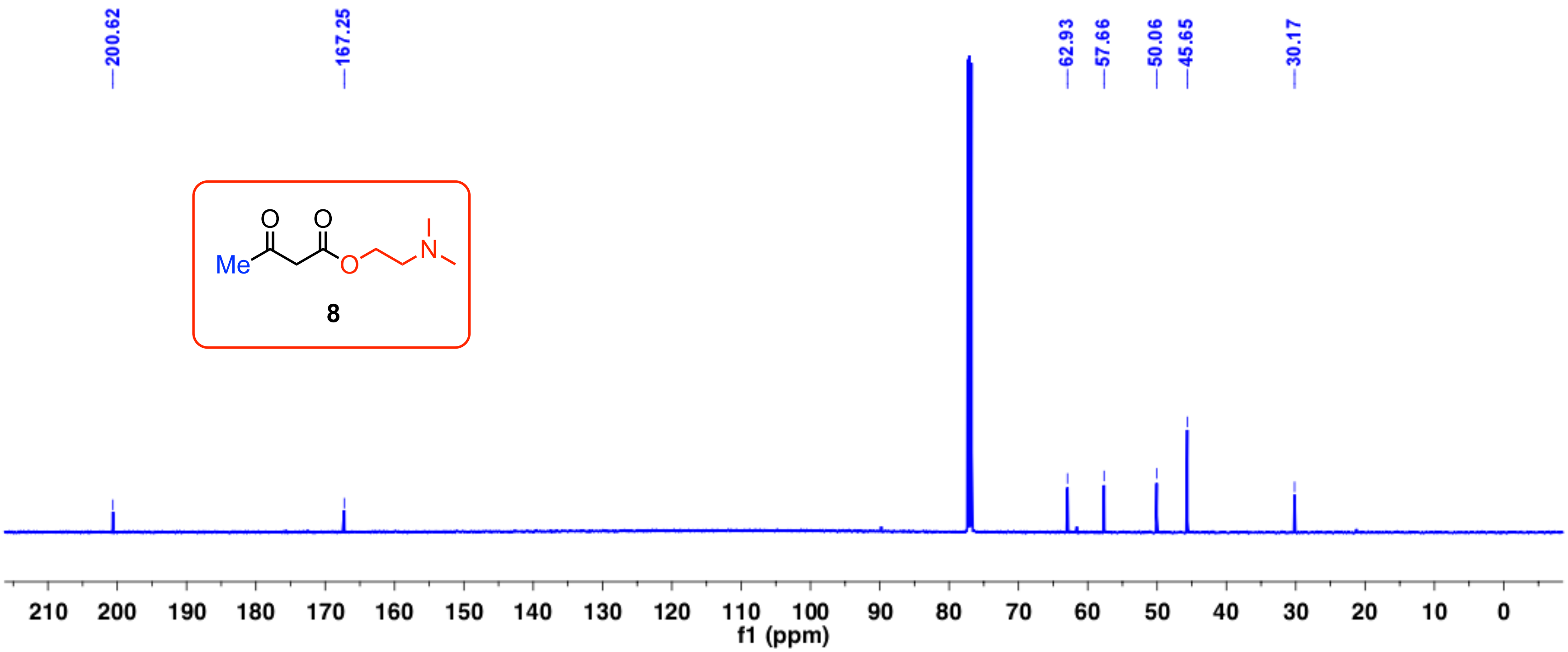

Figure S73: ^{13}C NMR of 1,3-diketone **8**.

18.10. Characterization of 1,3-diketone **1k**.

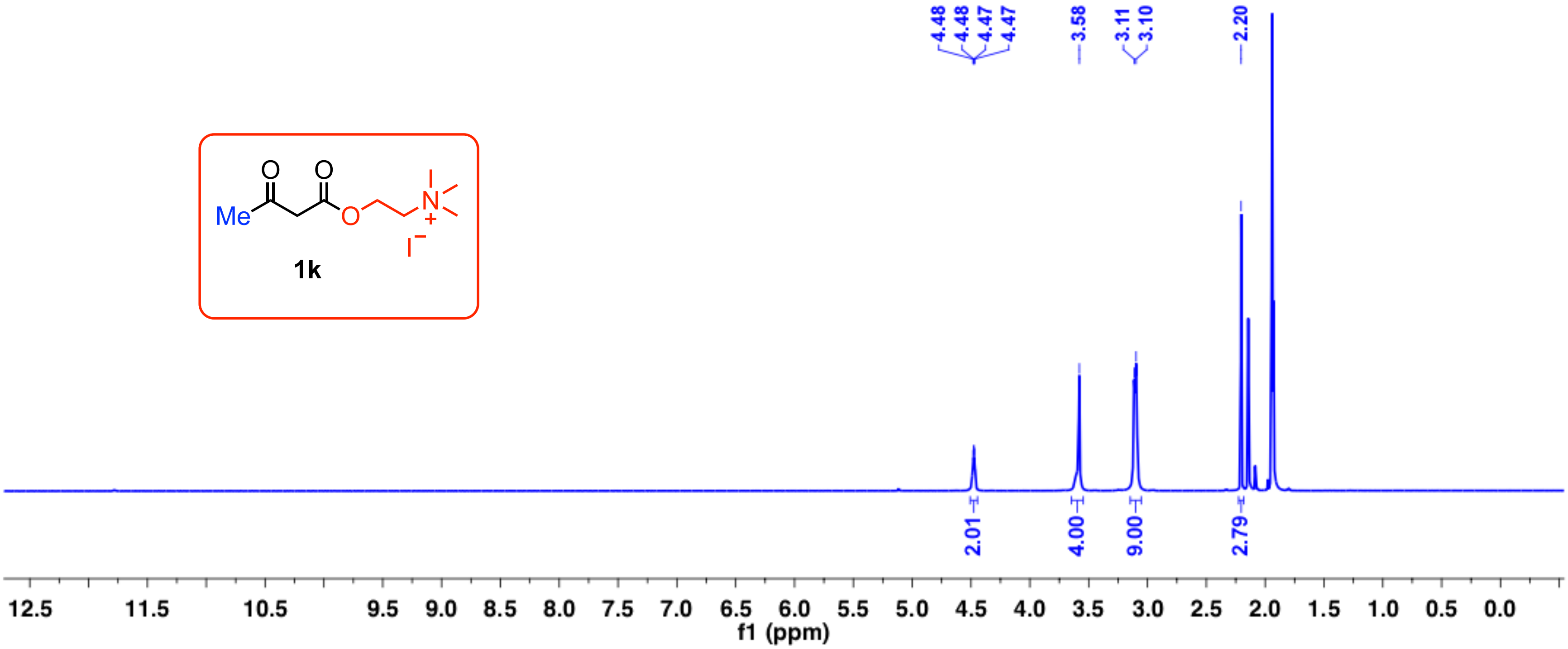

Figure S74: ^{1}H NMR of 1,3-diketone **1k**.

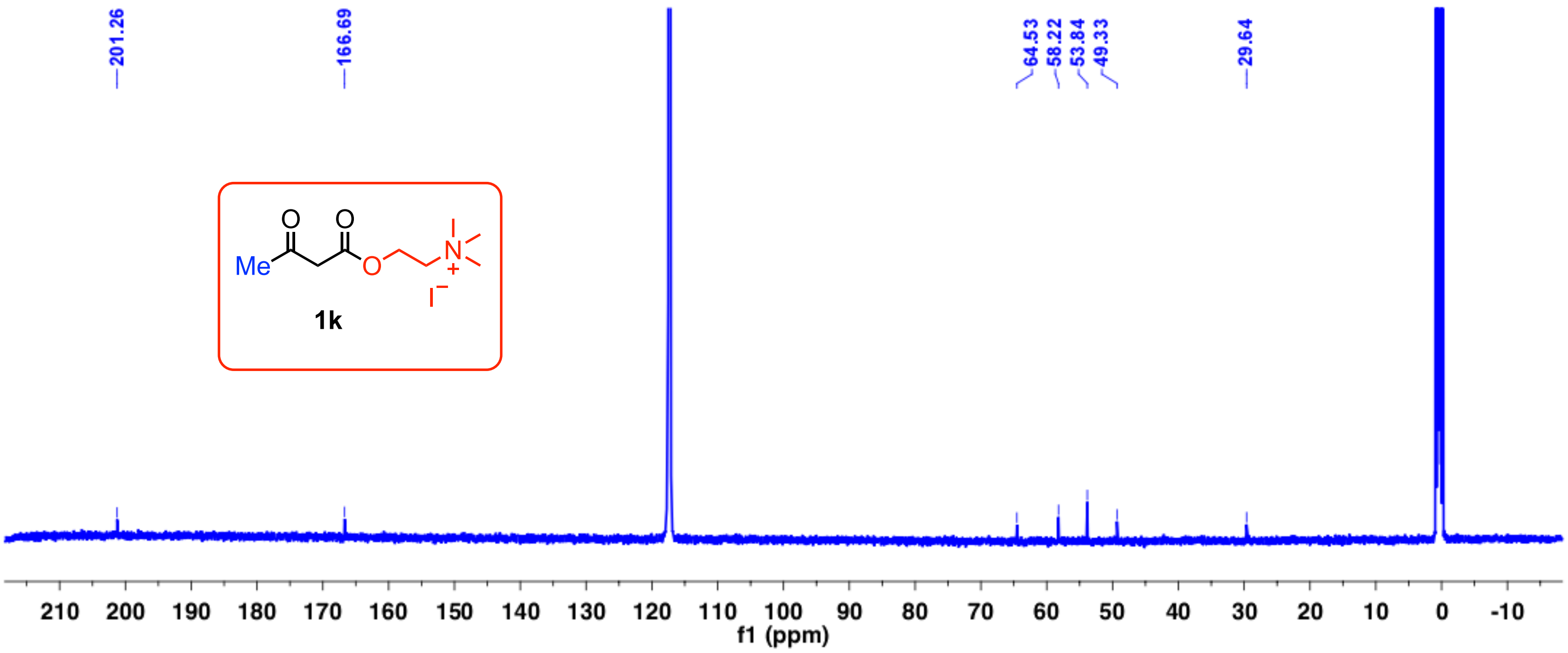

Figure S75: ^{13}C NMR of 1,3-diketone **1k**.

18.11. Characterization of diketone **1n**.

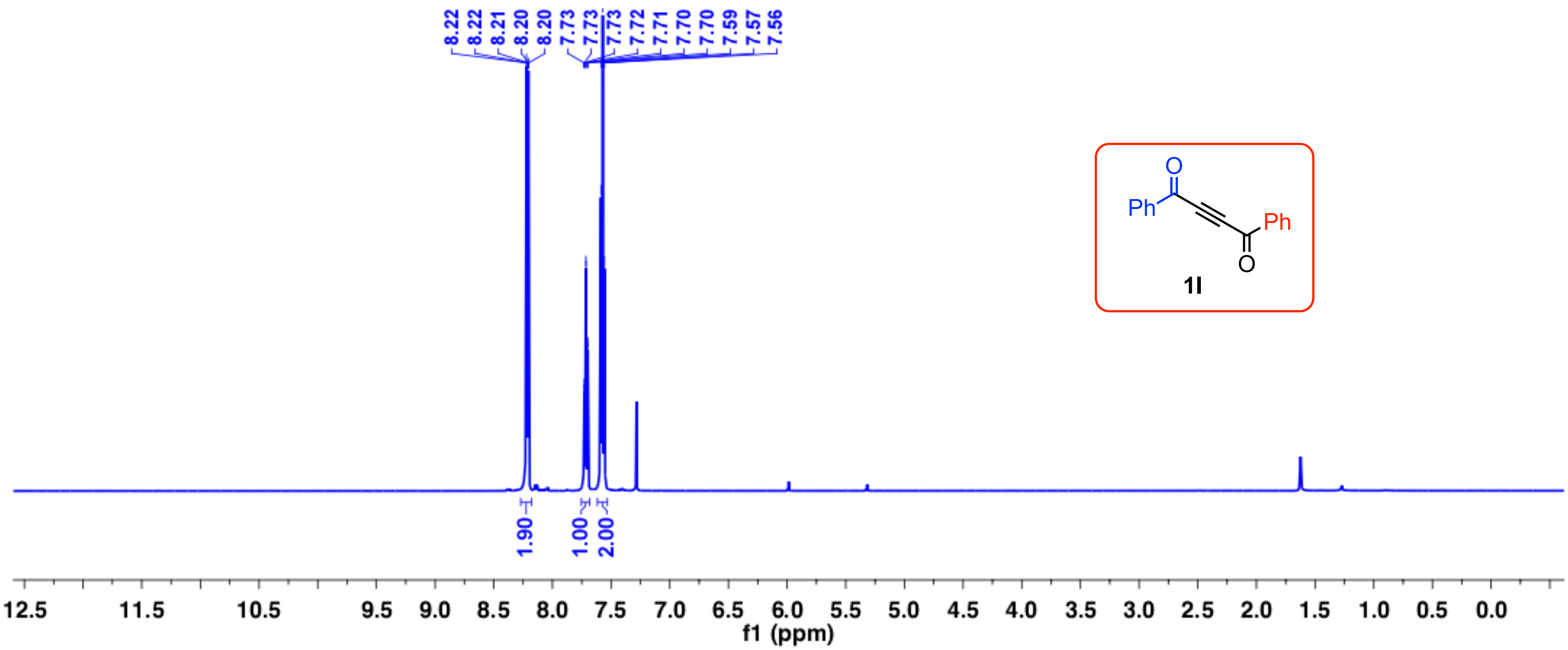

Figure S76: ^{1}H NMR of diketone **1n**.

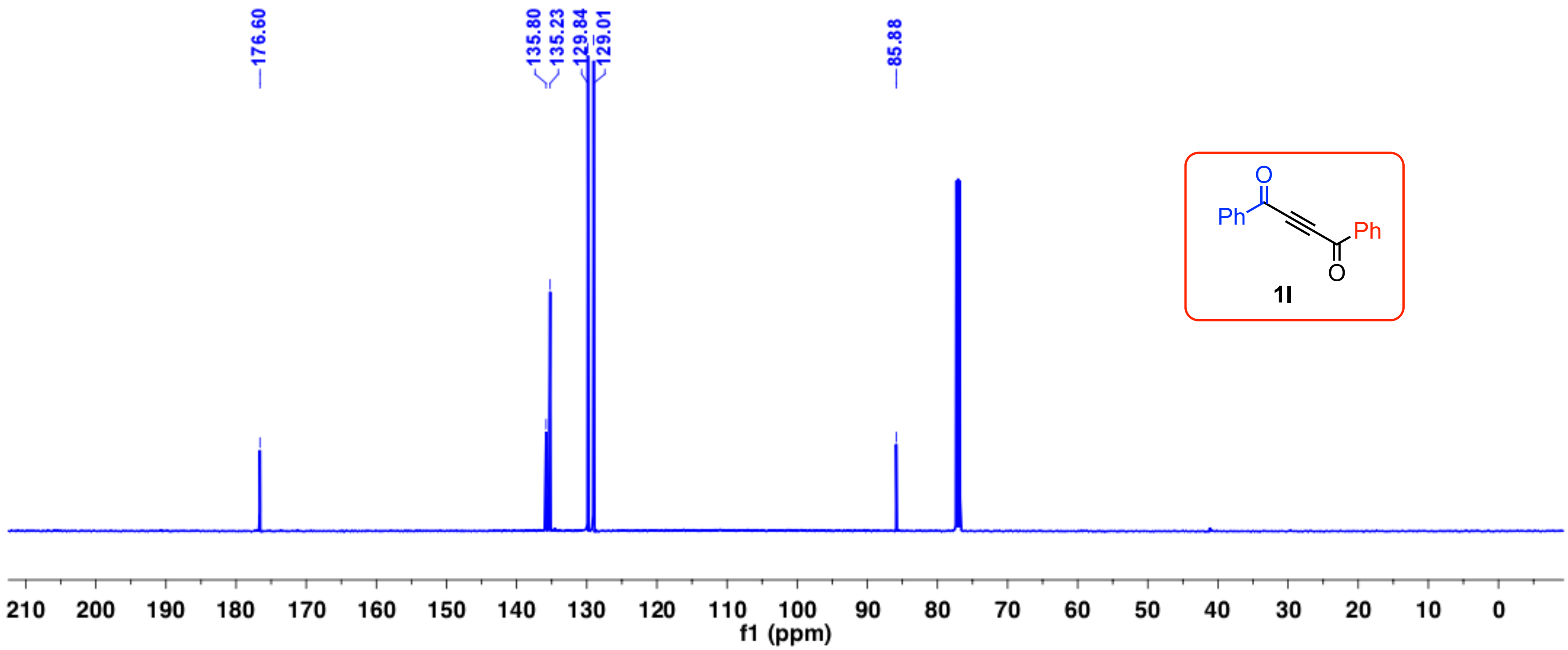

Figure S77: ^{13}C NMR of 1,3-diketone **1l**.

18.1. Characterization of 2(3-furan)benzenamine **2A**.

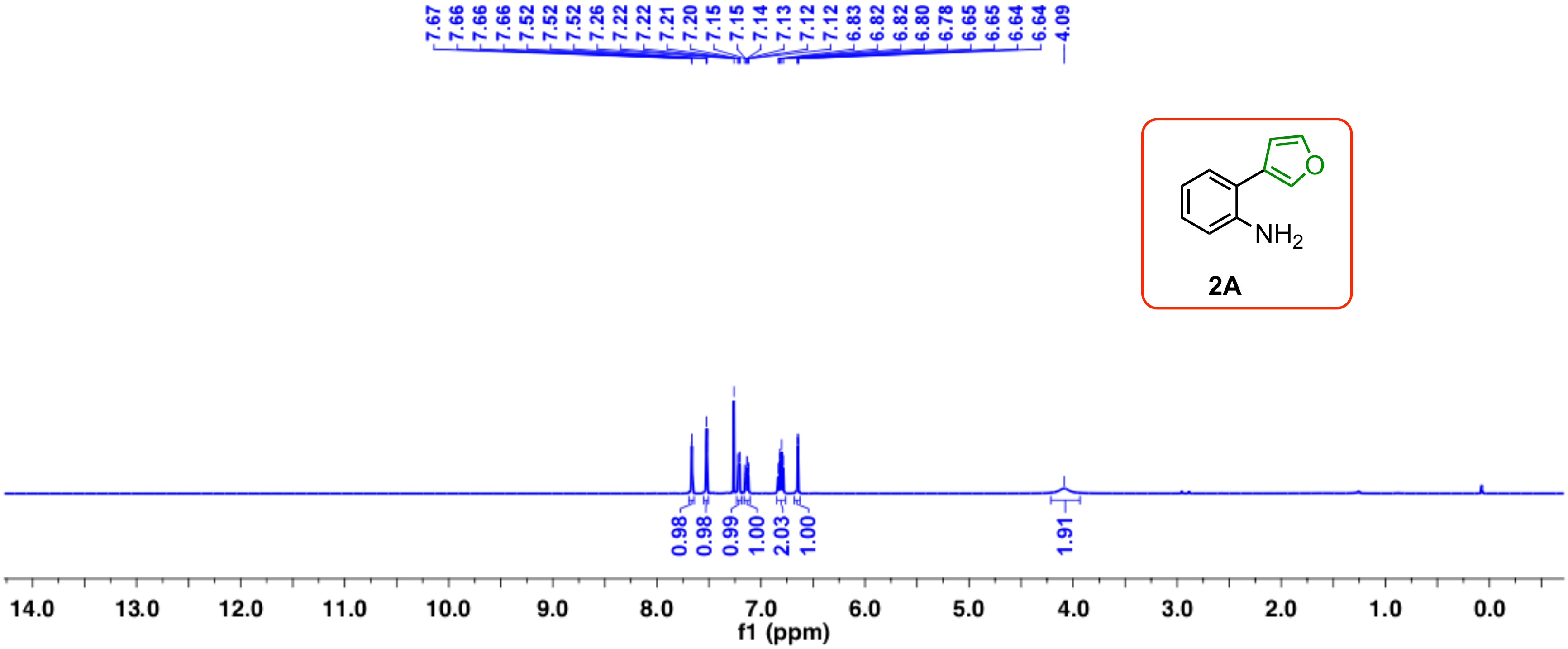

Figure S78: ^{1}H NMR of 2(3-furan)benzenamine **2A**.

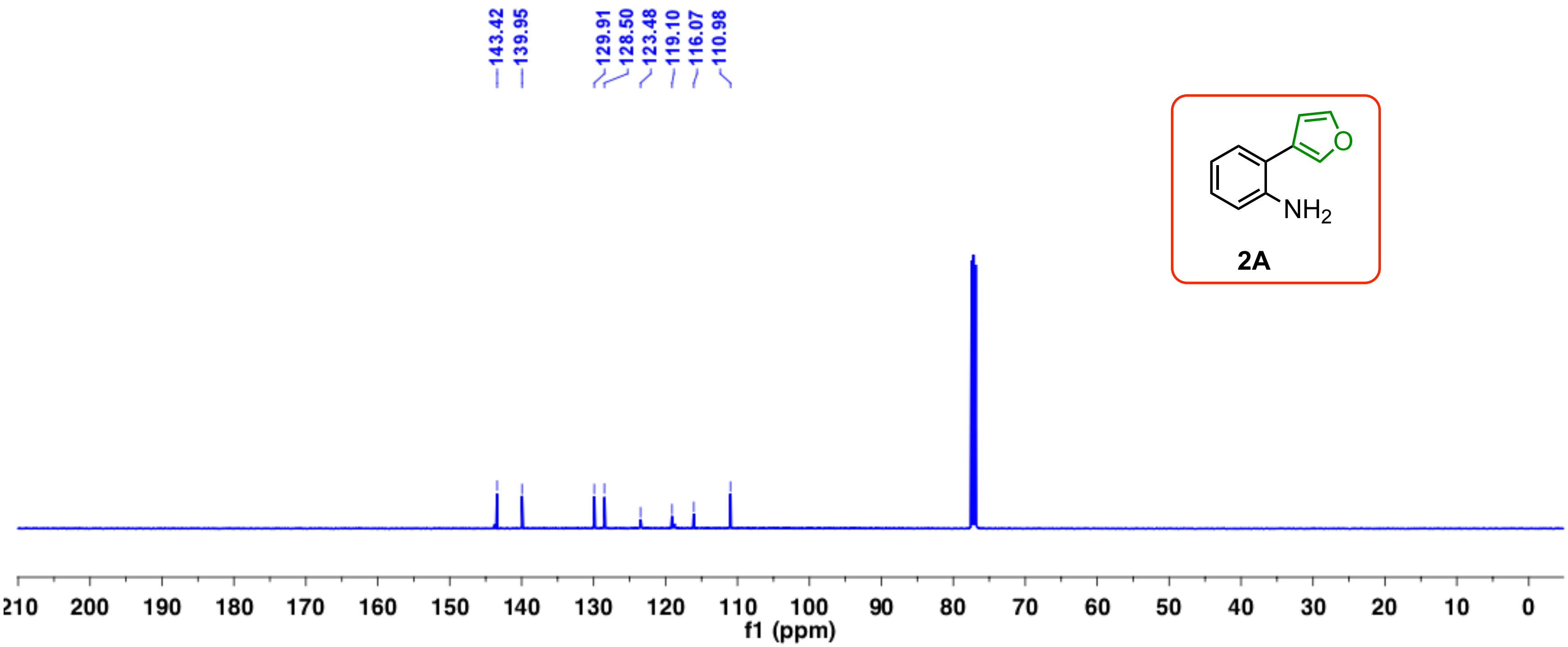

Figure S79: ^{13}C NMR of 2(3-furan)benzenamine **2A**.

18.2. Characterization of 2(3-thienyl)benzenamine **2B**.

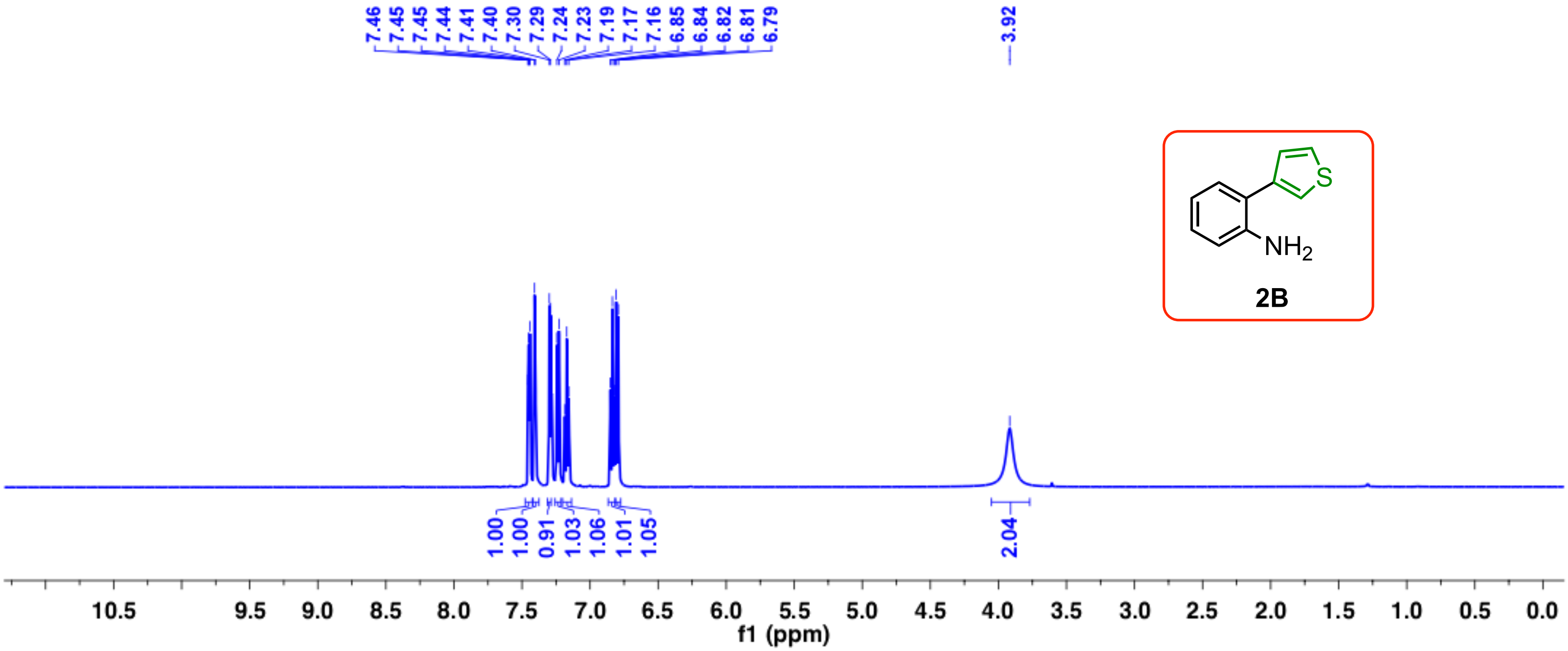

Figure S80: ^{1}H NMR of 2(3-thienyl)benzenamine **2B**.

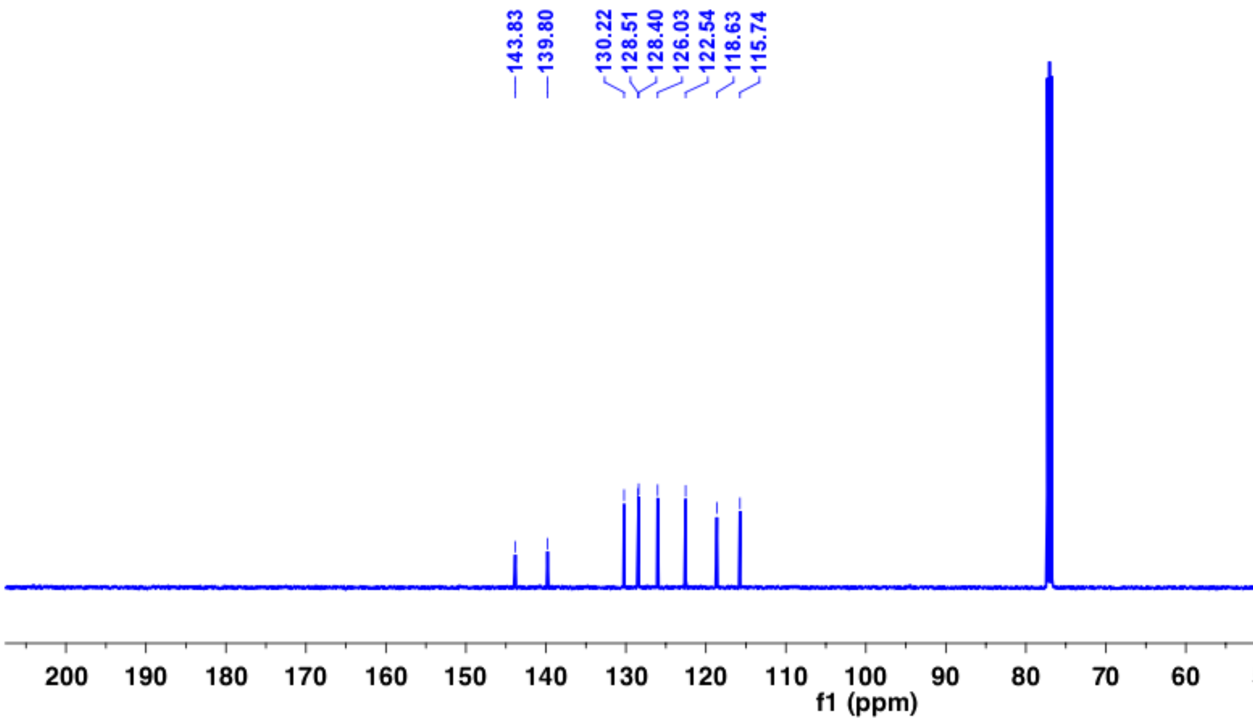

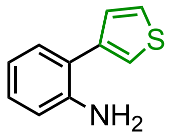

2B

Figure S81: ^{13}C NMR of 2(3-thionyl)benzaniline **2B**.

18.1. Characterization of 2(3-benzofuranyl)benzaniline **2C**.

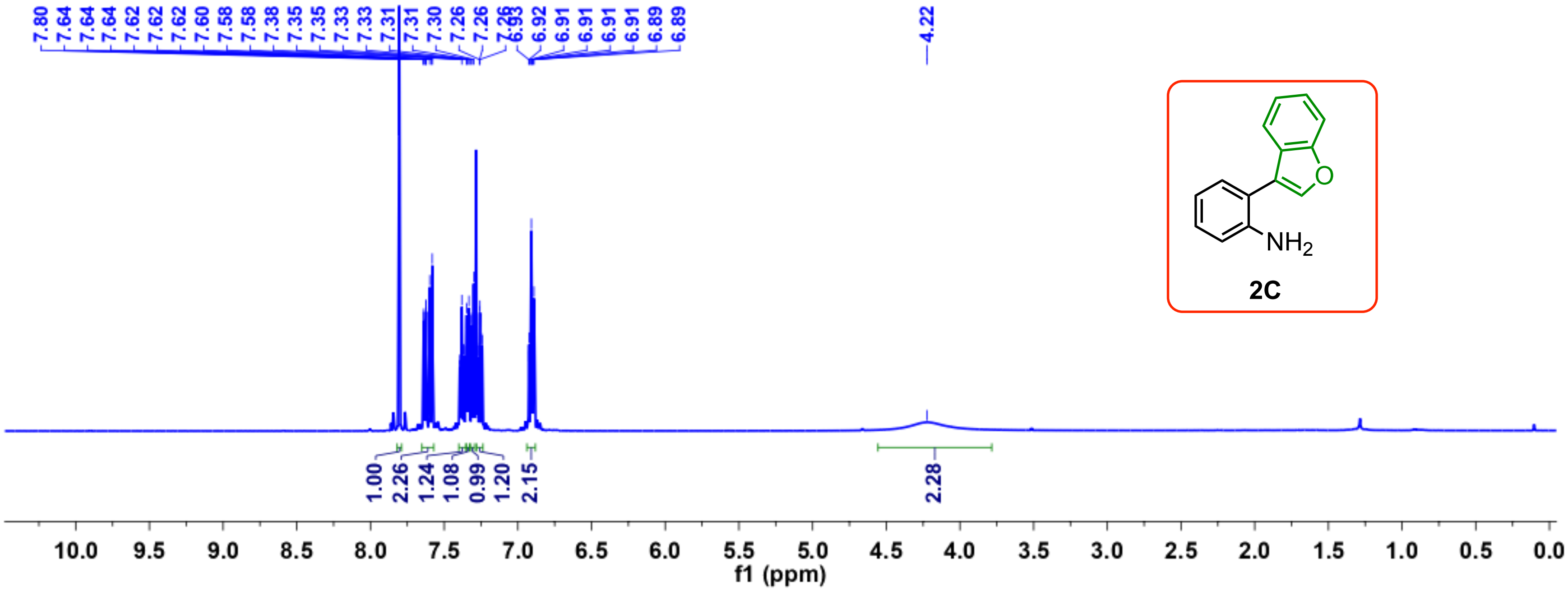

Figure S82: ^{1}H NMR of 2(3-benzofuranyl)benzaniline **2C**.

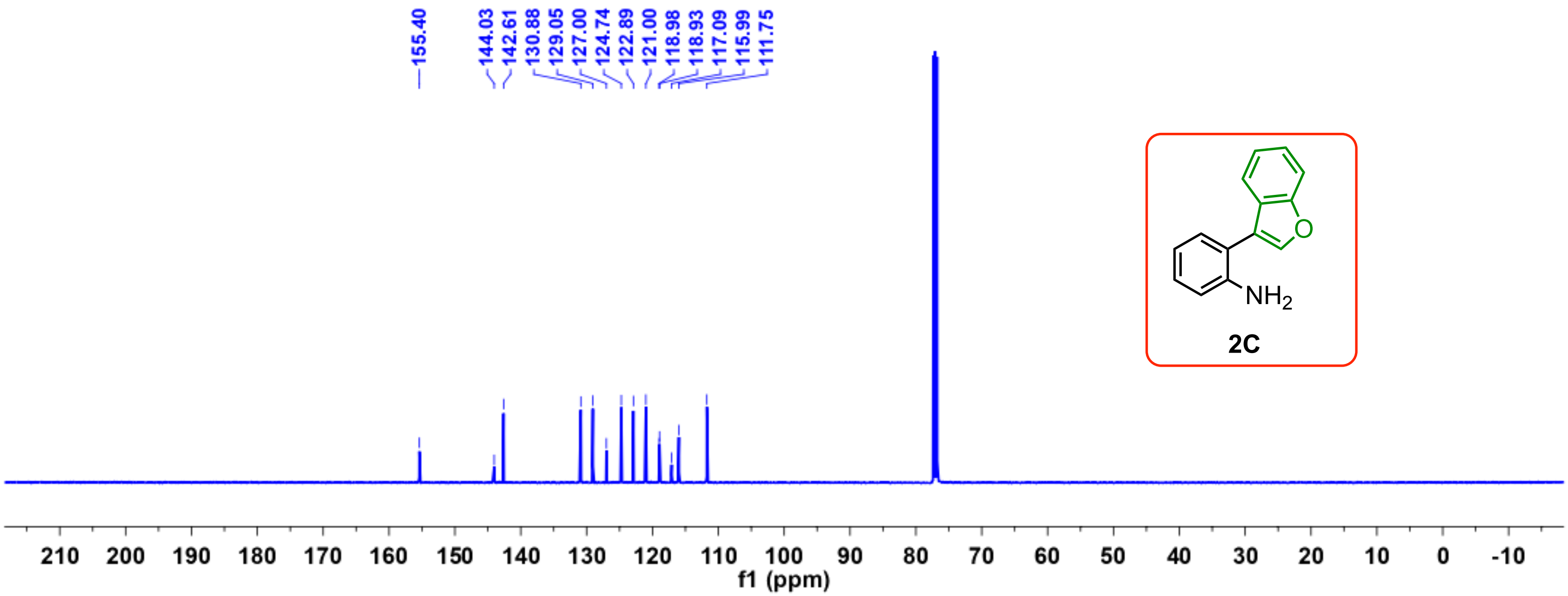

Figure S83: ^{13}C NMR of 2(3-benzofuranyl)benzaniline **2C**.

18.1. Characterization of 2(3-benzothienyl)benzaniline **2D**.

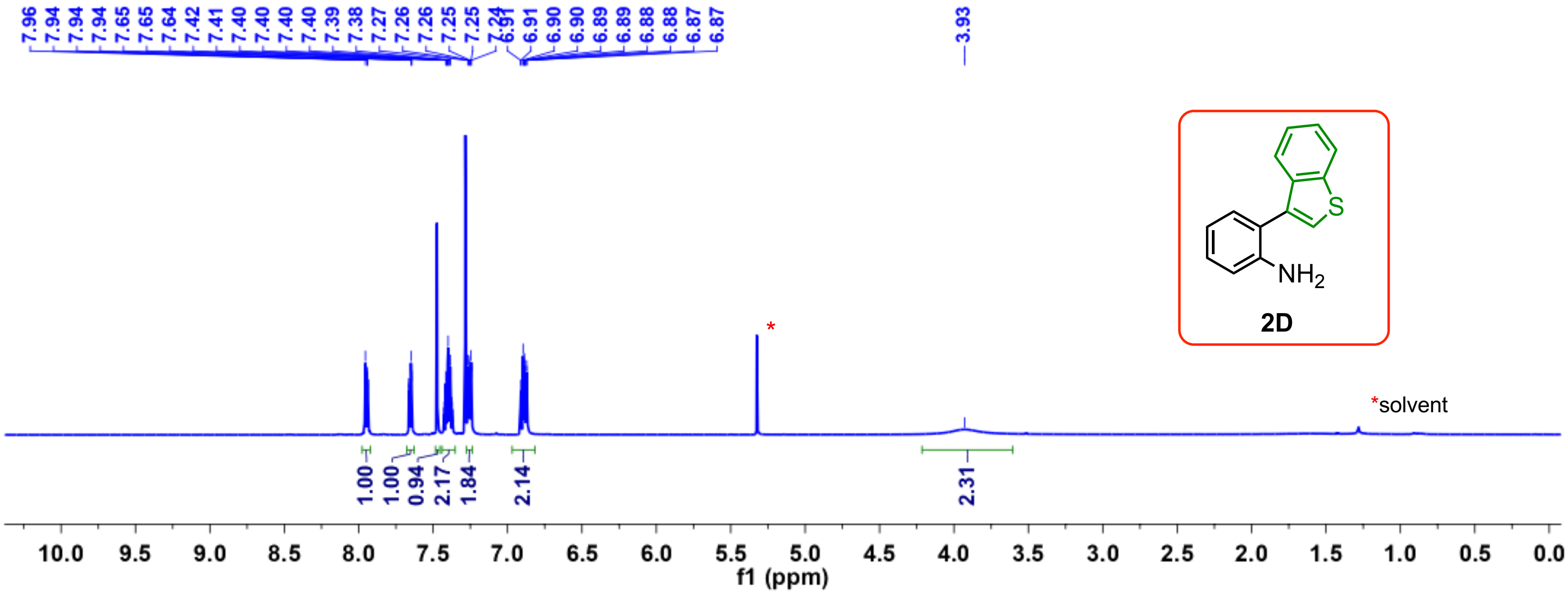

Figure S84: ^{1}H NMR of 2(3-benzothienyl)benzaniline **2D**.

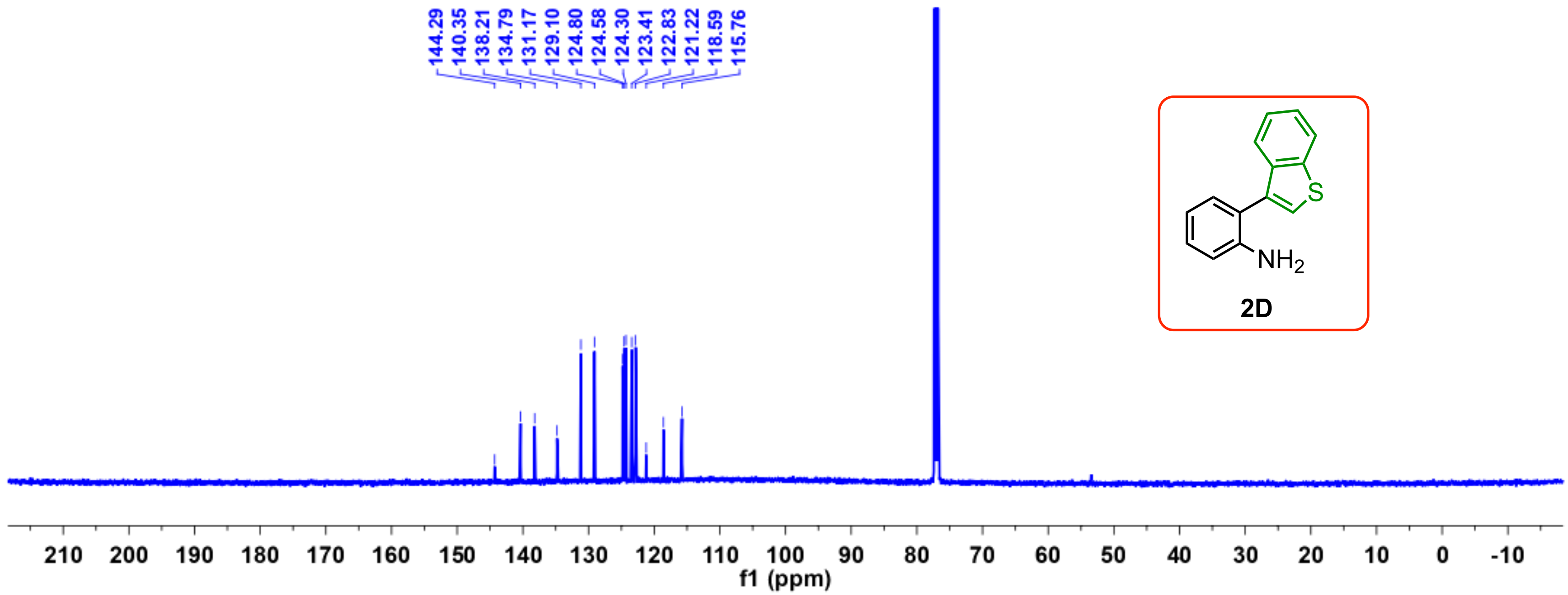

Figure S85: ^{13}C NMR of 2(3-benzothienyl)benzaniline **2D**.

18.1. Characterization of 2-(2-benzofuranyl)benzaniline **2E**.

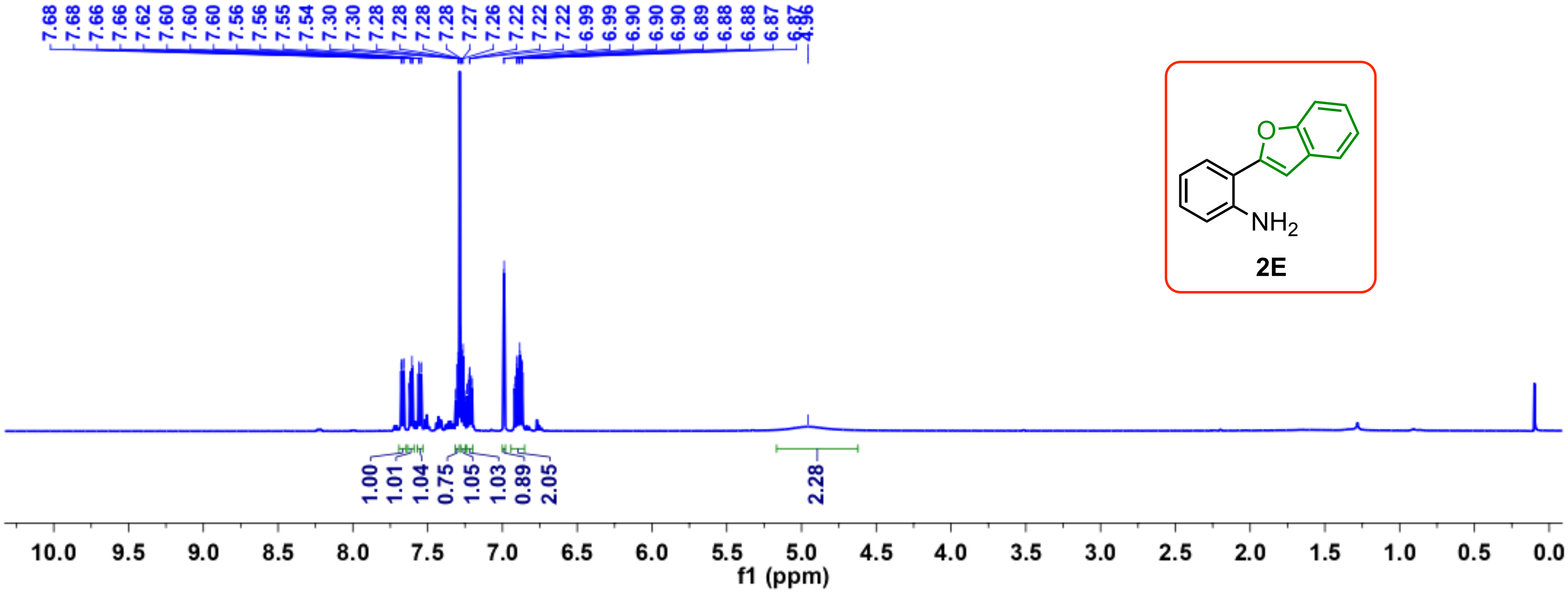

Figure S86: ^{1}H NMR of 2-(2-benzofuranyl)benzaniline **2E**.

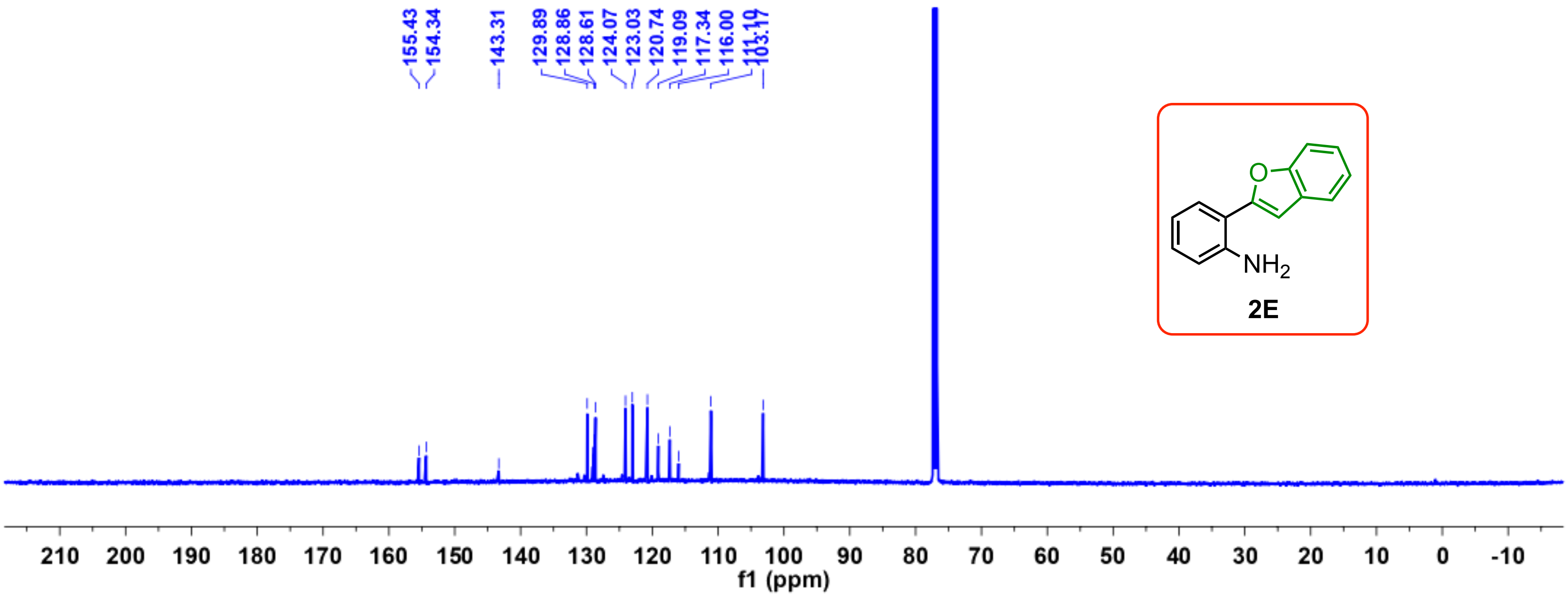

Figure S87: ^{13}C NMR of 2-(2-benzofuranyl)benzaniline **2E**.

18.1. Characterization of 2-(2-benzothienyl)benzaniline **2F**.

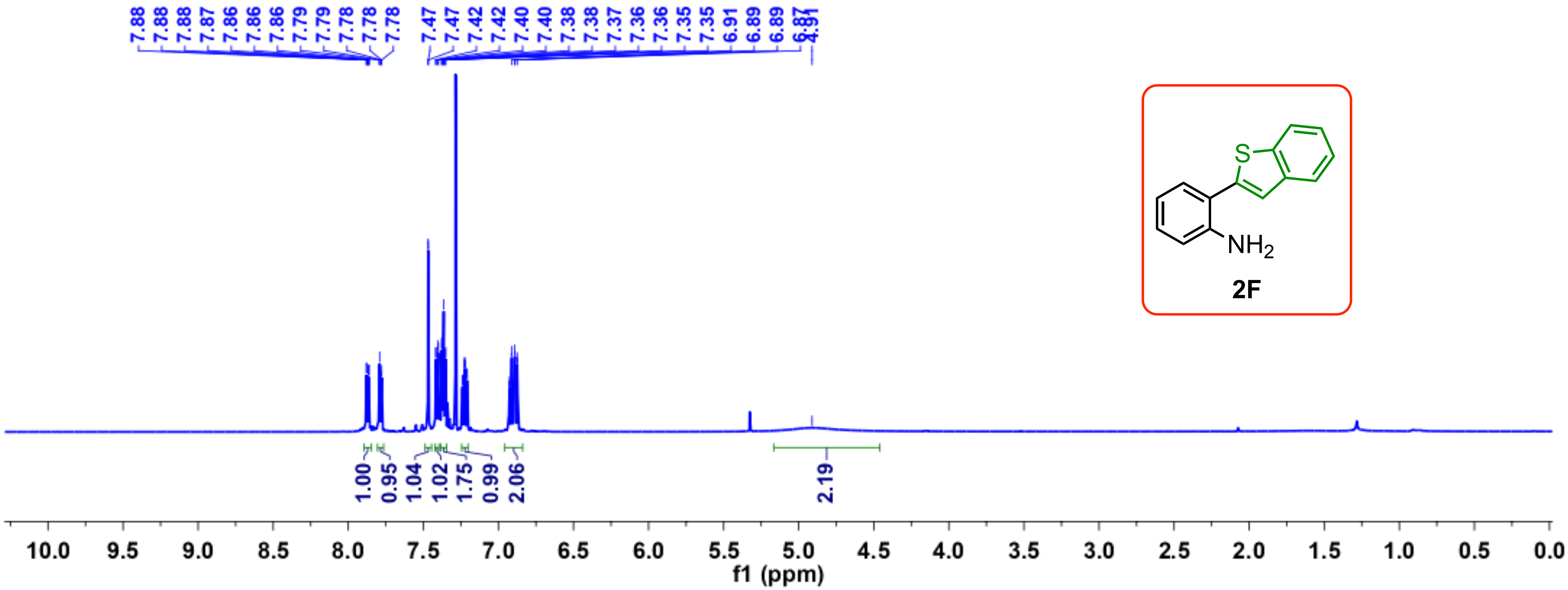

Figure S88: ^{1}H NMR of 2-(2-benzothienyl)benzaniline **2F**.

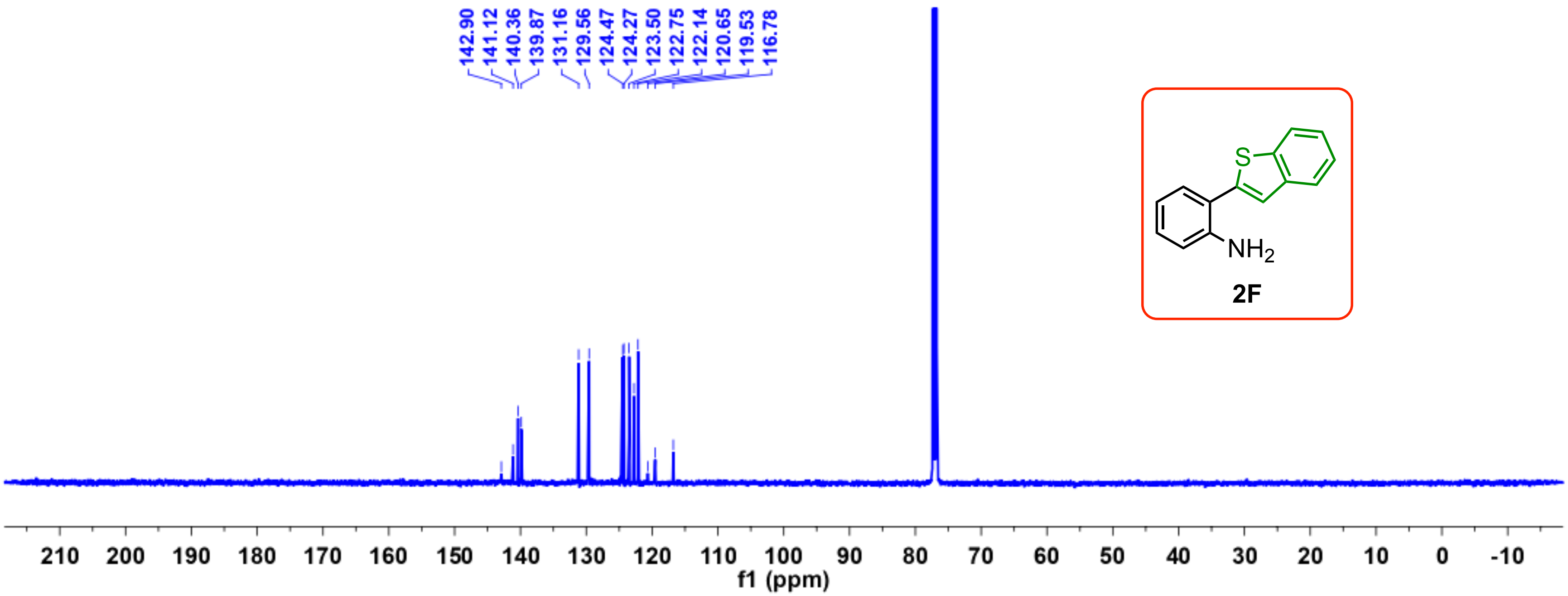

Figure S89: ^{13}C NMR of 2-(2-benzothienyl)benzaniline **2F**.

18.1. Characterization of 1-(2-Aminophenyl)ethanone 2-methyl-2-phenylhydrazone **15**.

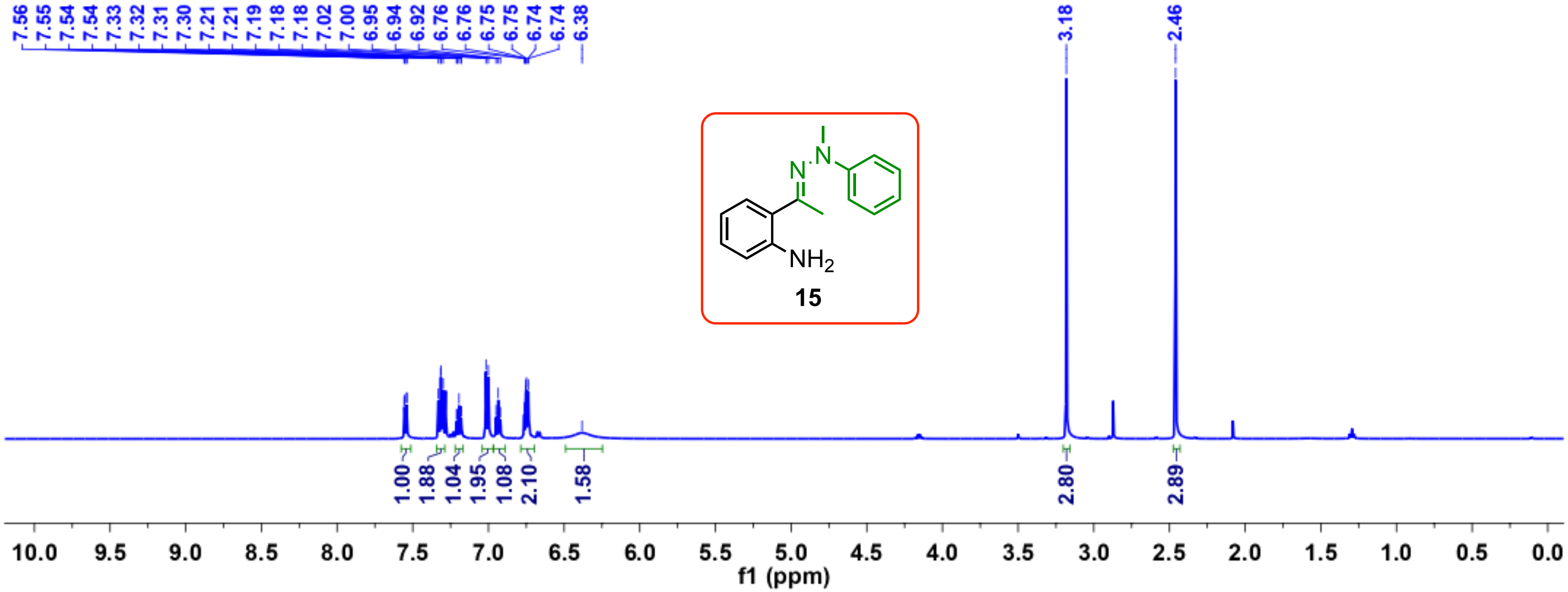

Figure S90: ^{1}H NMR of compound **15**.

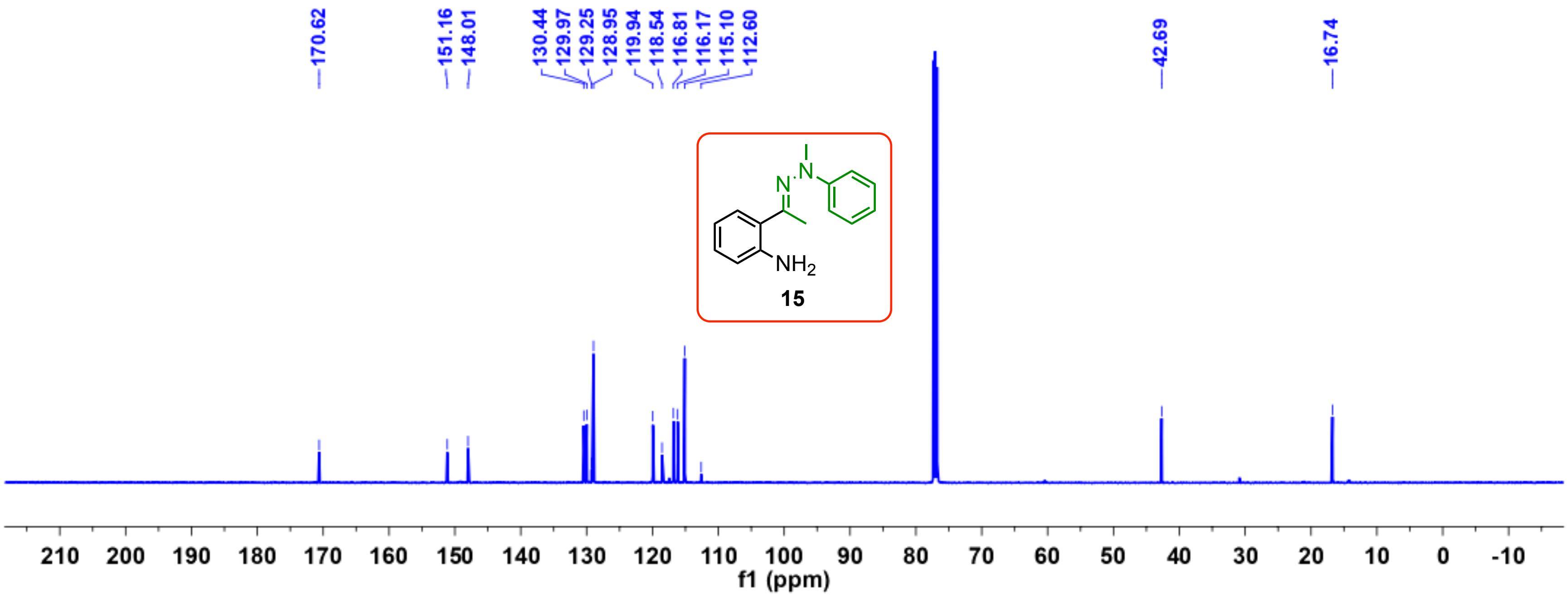

Figure S91: ^{13}C NMR of compound **15**.

18.1. Characterization of 2-(1-Methyl-1H-indol-2-yl)benzenaniline **2G**.

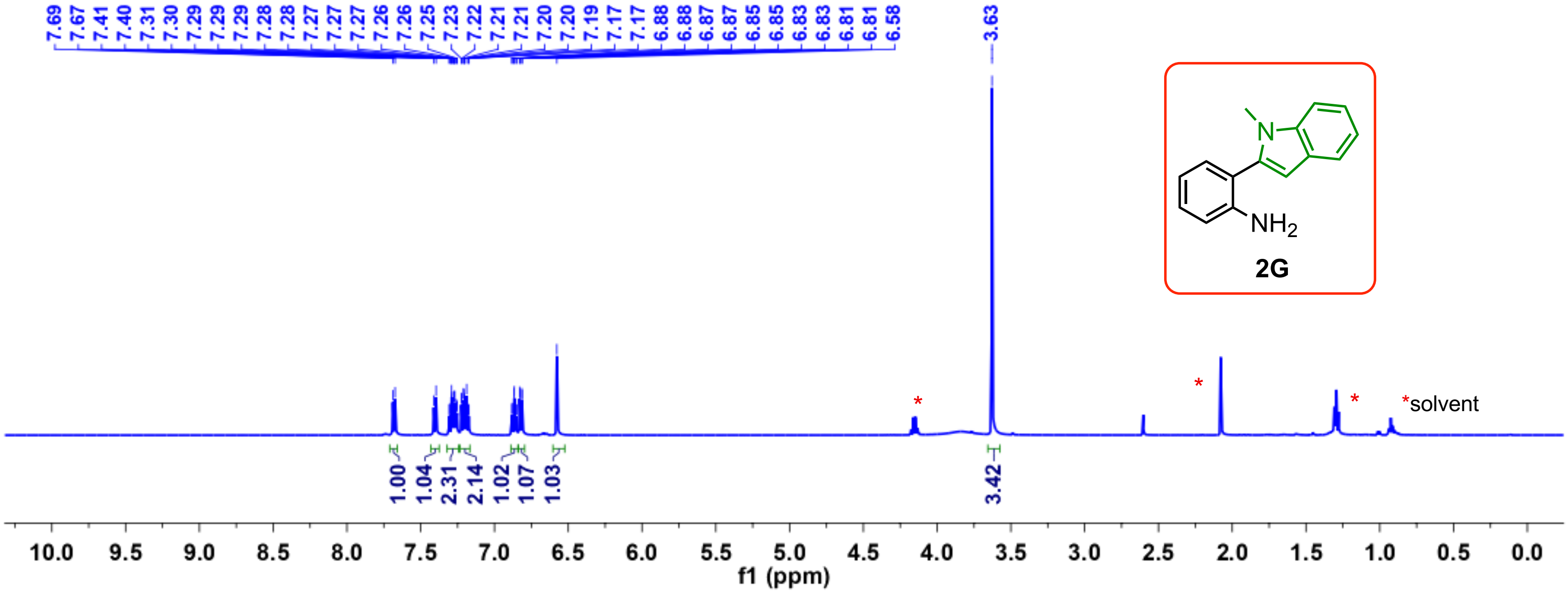

Figure S92: ^{1}H NMR of 2-(1-Methyl-1H-indol-2-yl)benzenaniline **2G**.

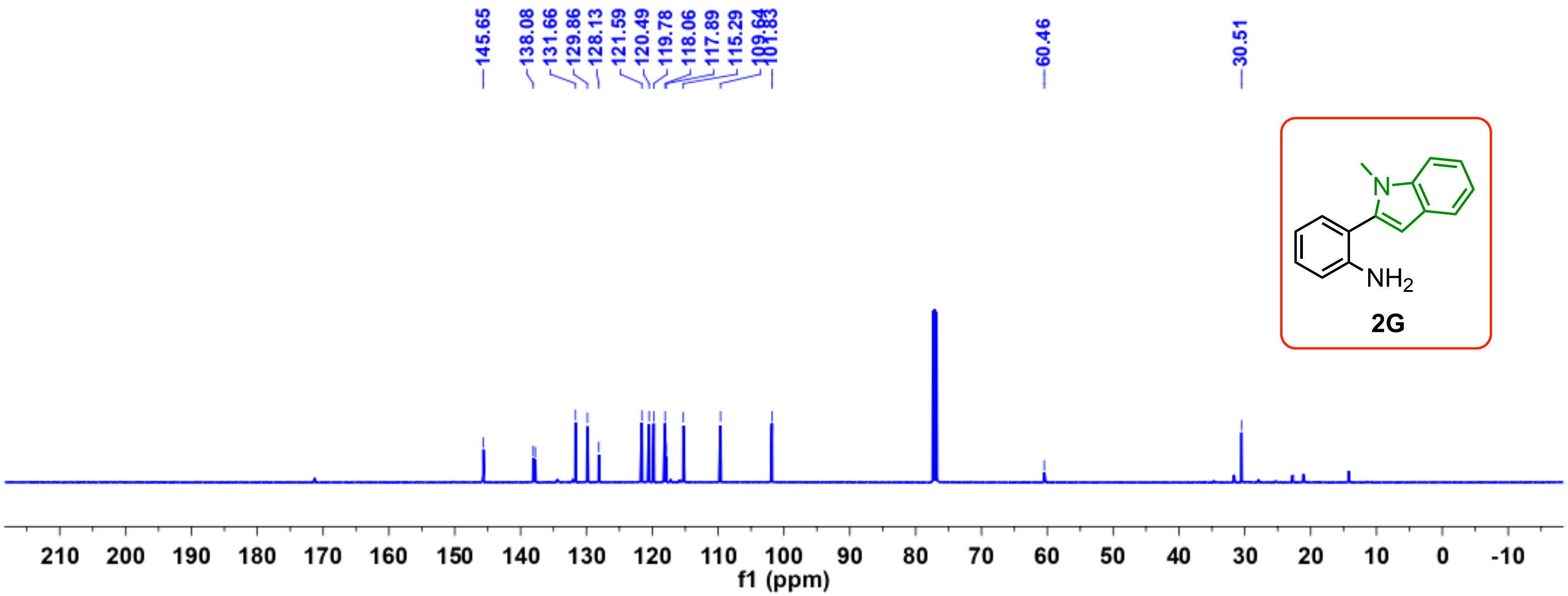

Figure S93: ^{13}C NMR of 2-(1-Methyl-1H-indol-2-yl)benzenaniline **2G**.

18.2. Characterization of enaminone **3Aa**.

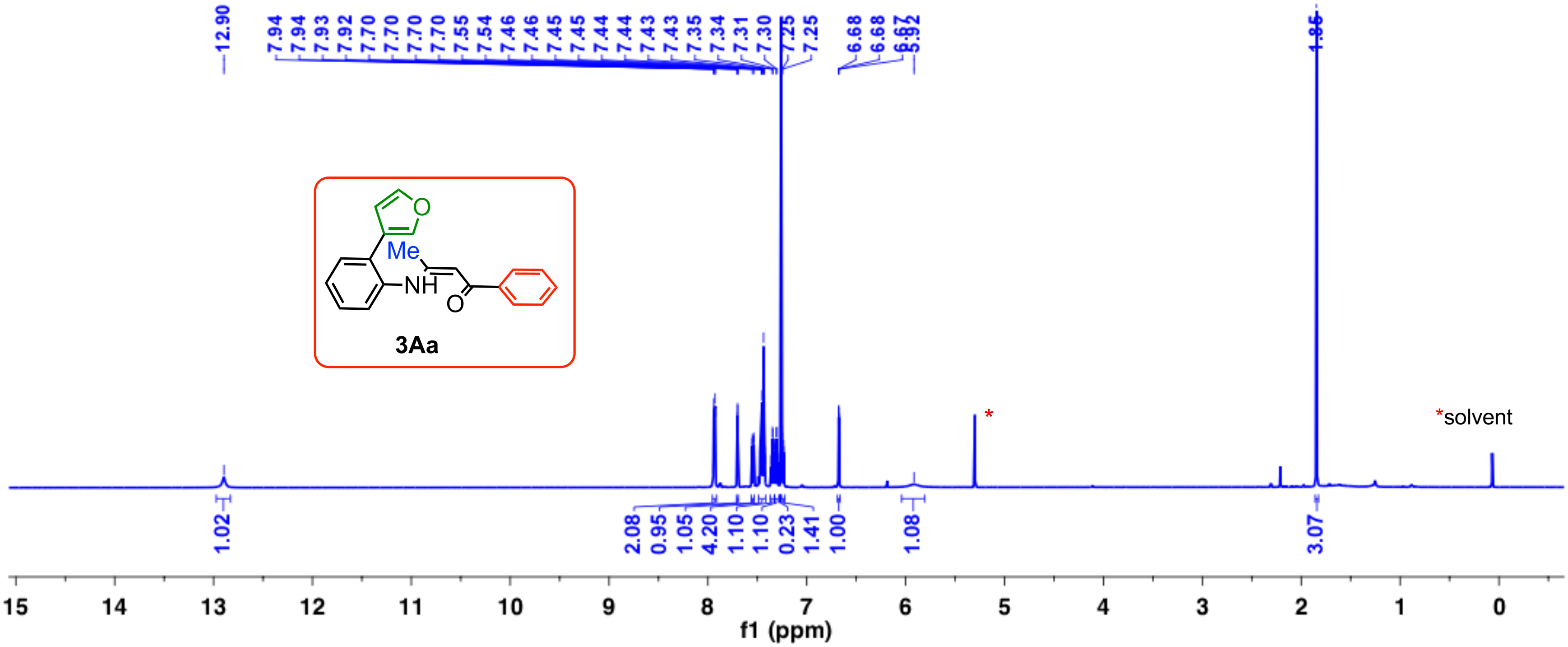

Figure S94: ^{1}H NMR of enaminone **3Aa**.

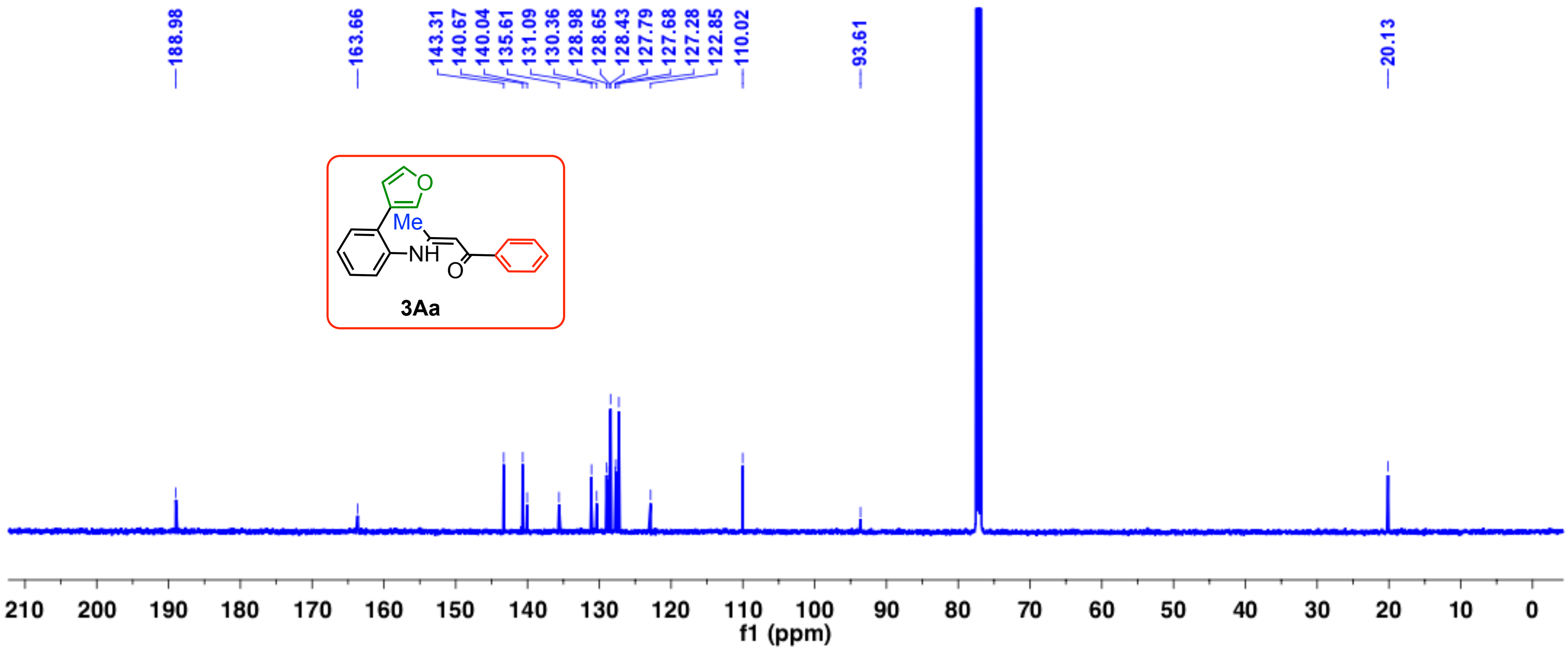

Figure S95: ^{13}C NMR of enaminone **3Aa**.

HRMS-ESI (m/z) ([M + H]$^+$):

Calculated: 340.1338

Observed: 340.1340

|Δm| = 0.66 ppm

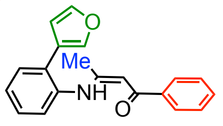

3Aa

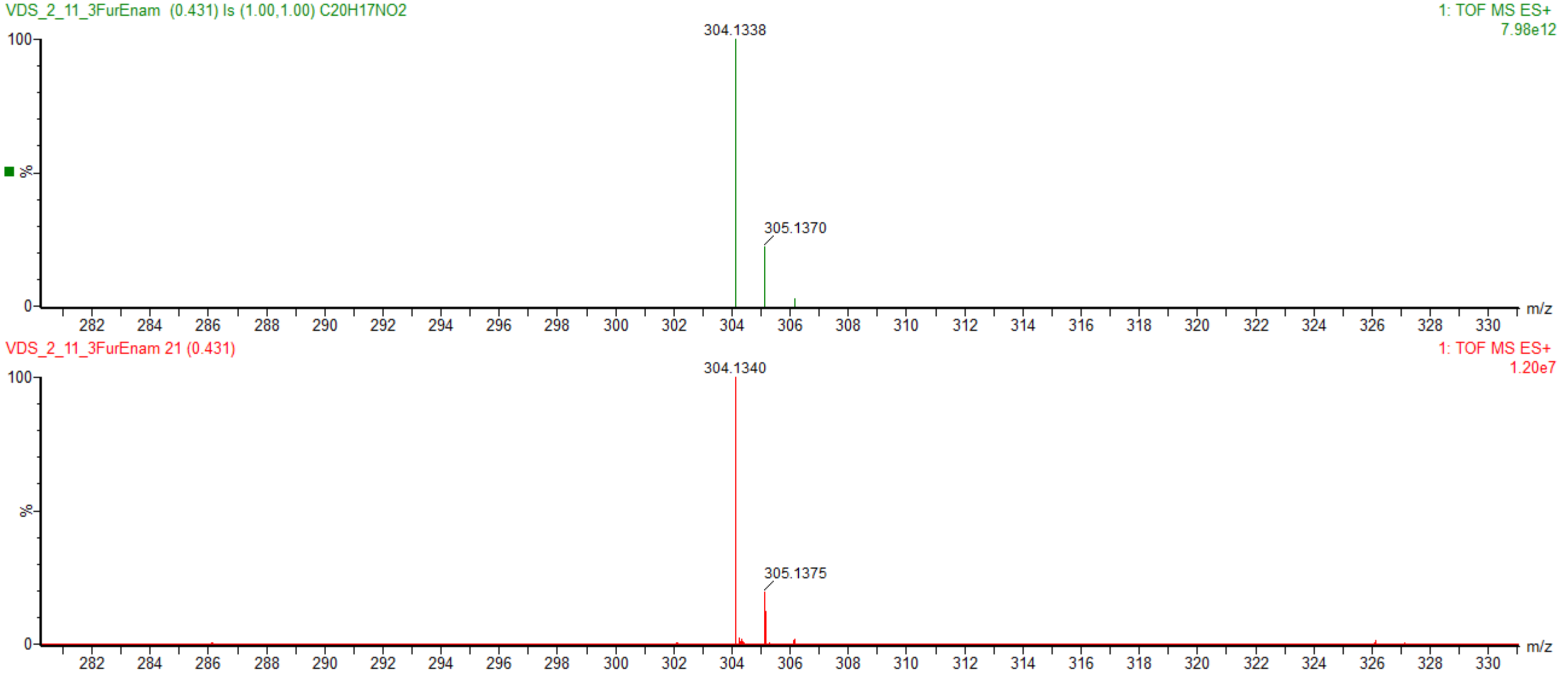

Figure S96: HRMS data of quinoline photoproduct **3Aa**.

18.3. Characterization of enaminone **3Ba**.

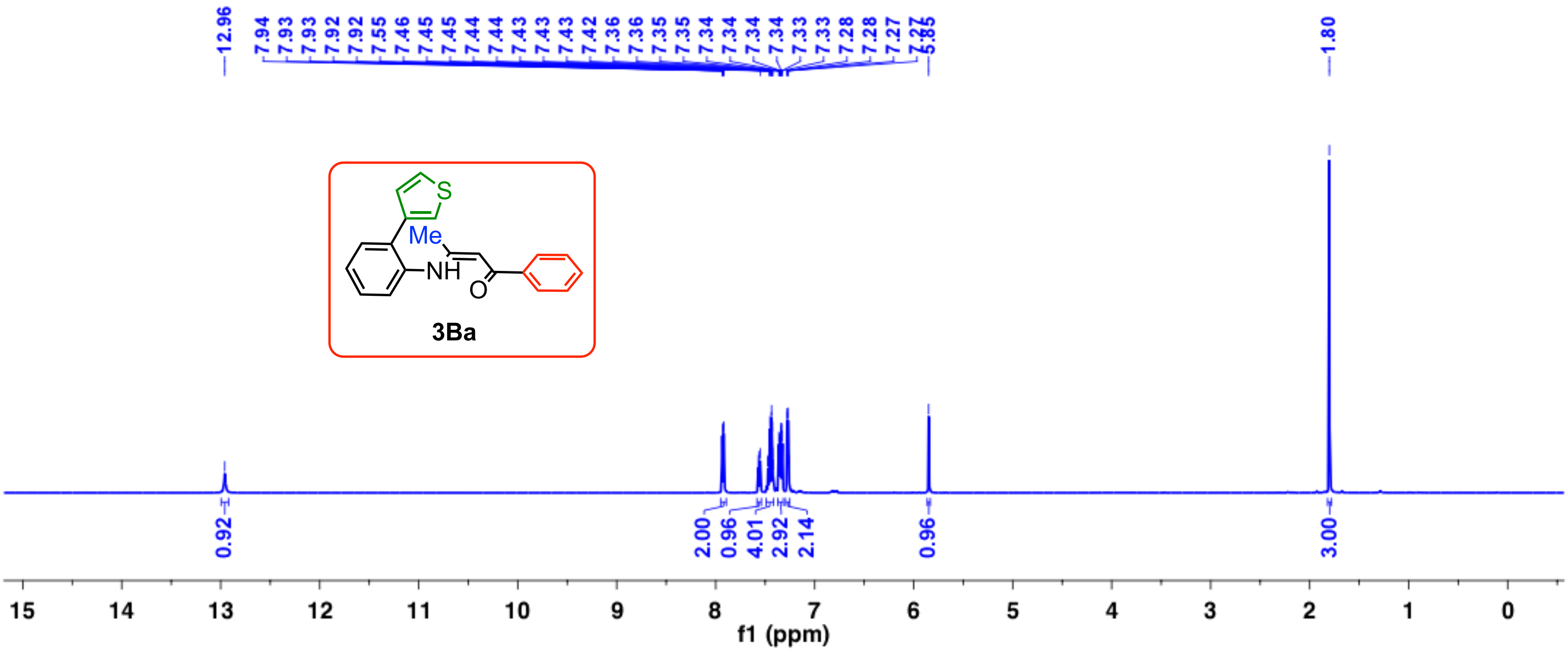

Figure S97: ^{1}H NMR of enaminone **3Ba**.

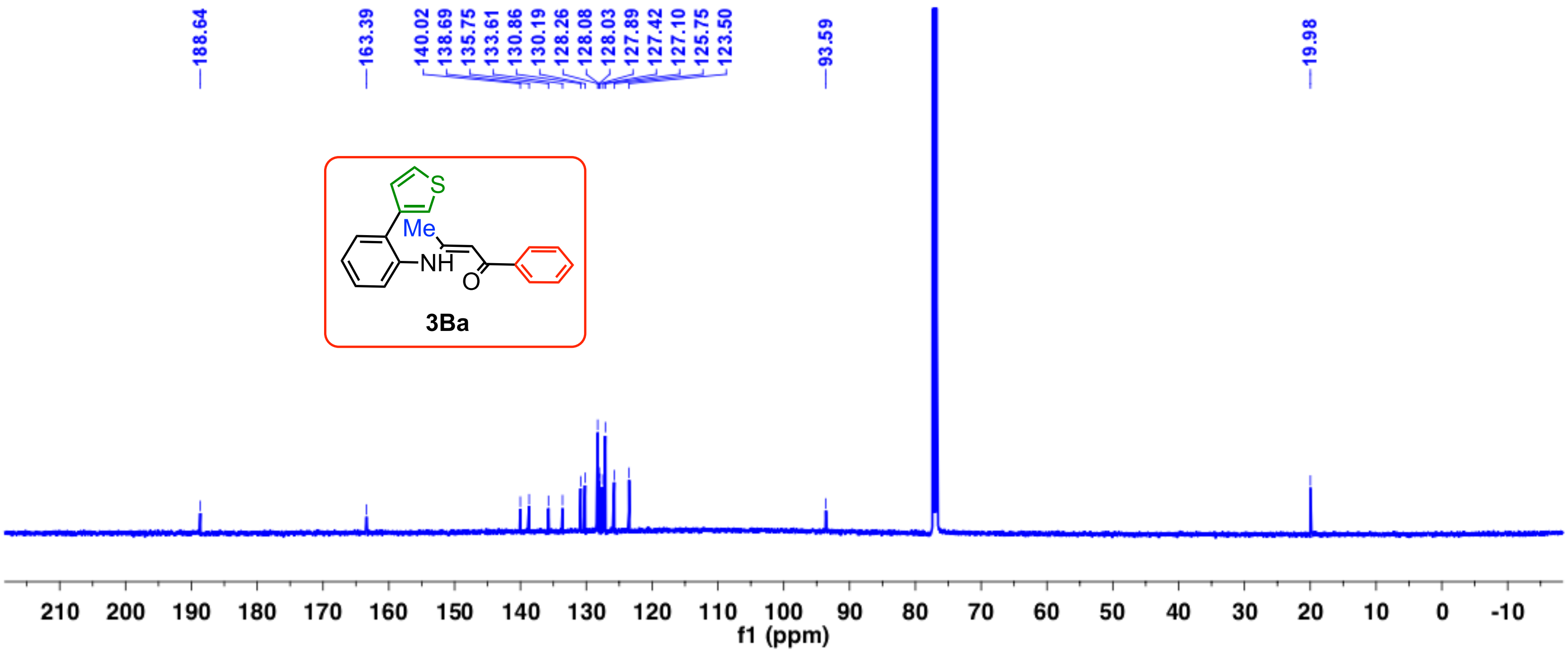

Figure S98: ^{13}C NMR of enaminone **3Ba**.

HRMS-ESI (m/z) ([M + H]$^+$):

Calculated: 320.1109

Observed: 320.1124

|Δm| = 4.7 ppm

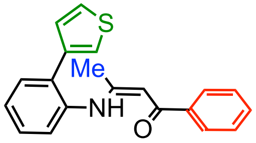

3Ba

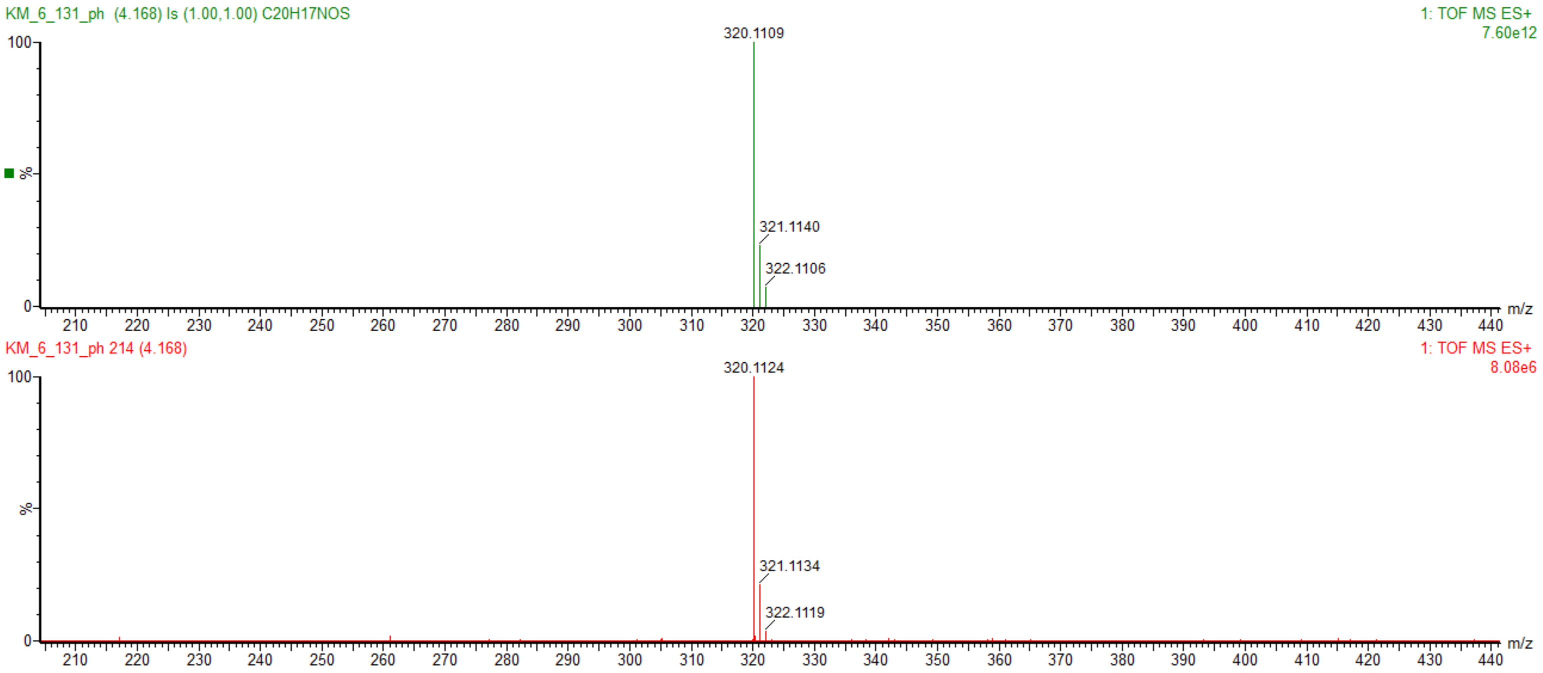

Figure S99: HRMS data of enaminone **3Ba**.

18.1. Characterization of enaminone **3Ca**.

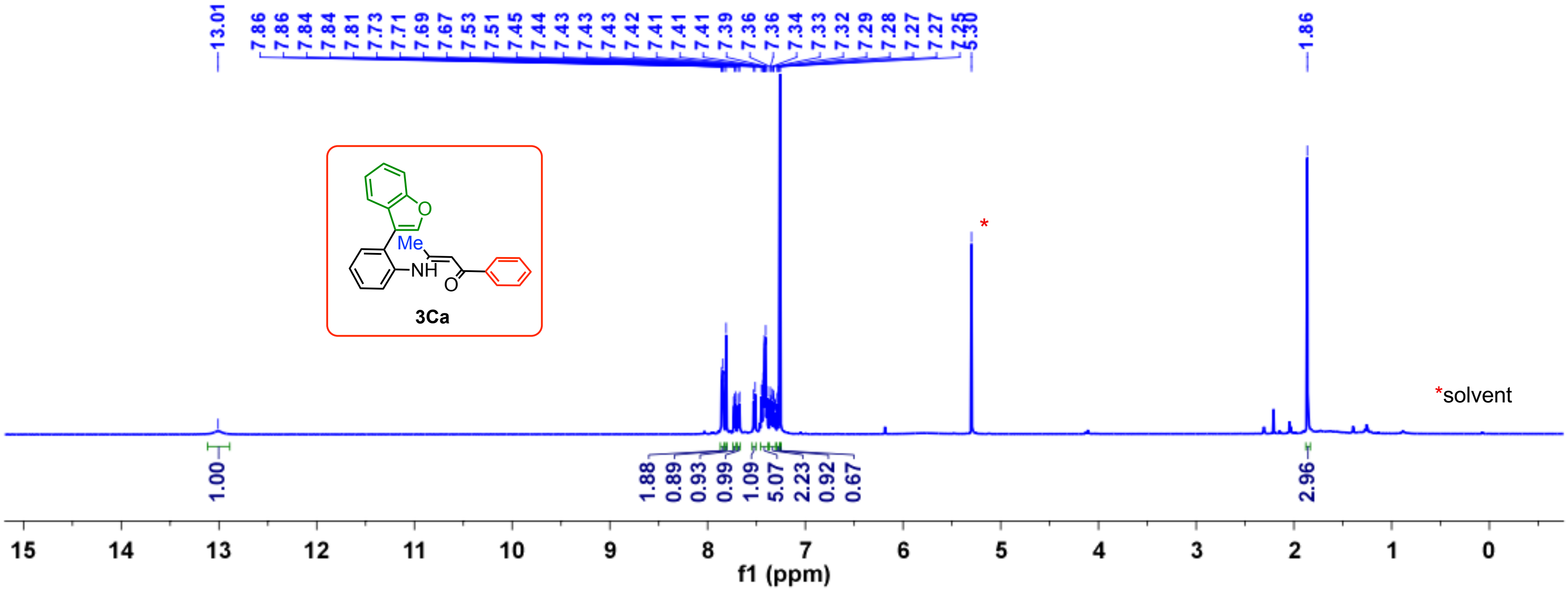

Figure S100: ^{1}H NMR of enaminone **3Ca**.

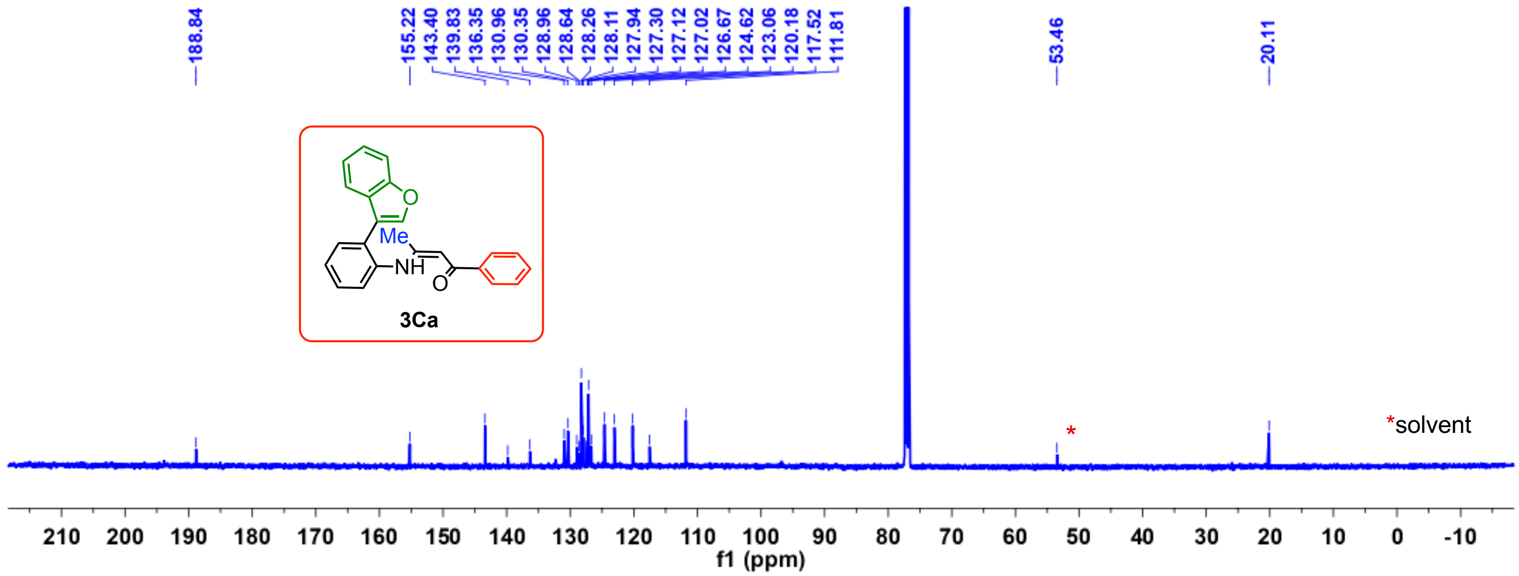

Figure S101: ^{13}C NMR of enaminone **3Ca**.

HRMS-ESI (m/z) ([M + H]$^+$):

Calculated: 354.1494

Observed: 354.1491

|Δm| = 0.85 ppm

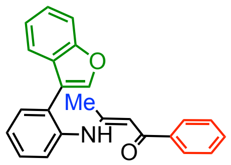

3Ca

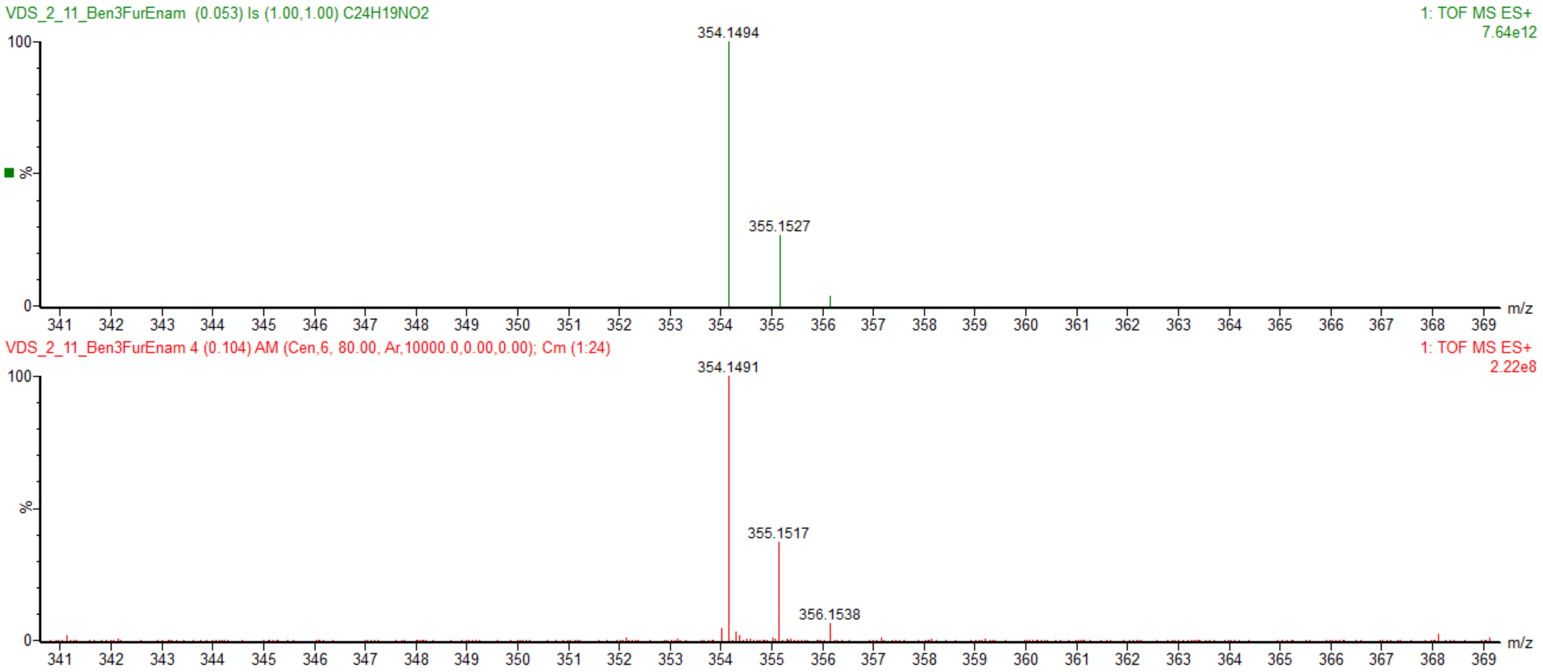

Figure S102: HRMS data of quinoline photoproduct **3Ca**.

18.1. Characterization of enaminone **3Da**.

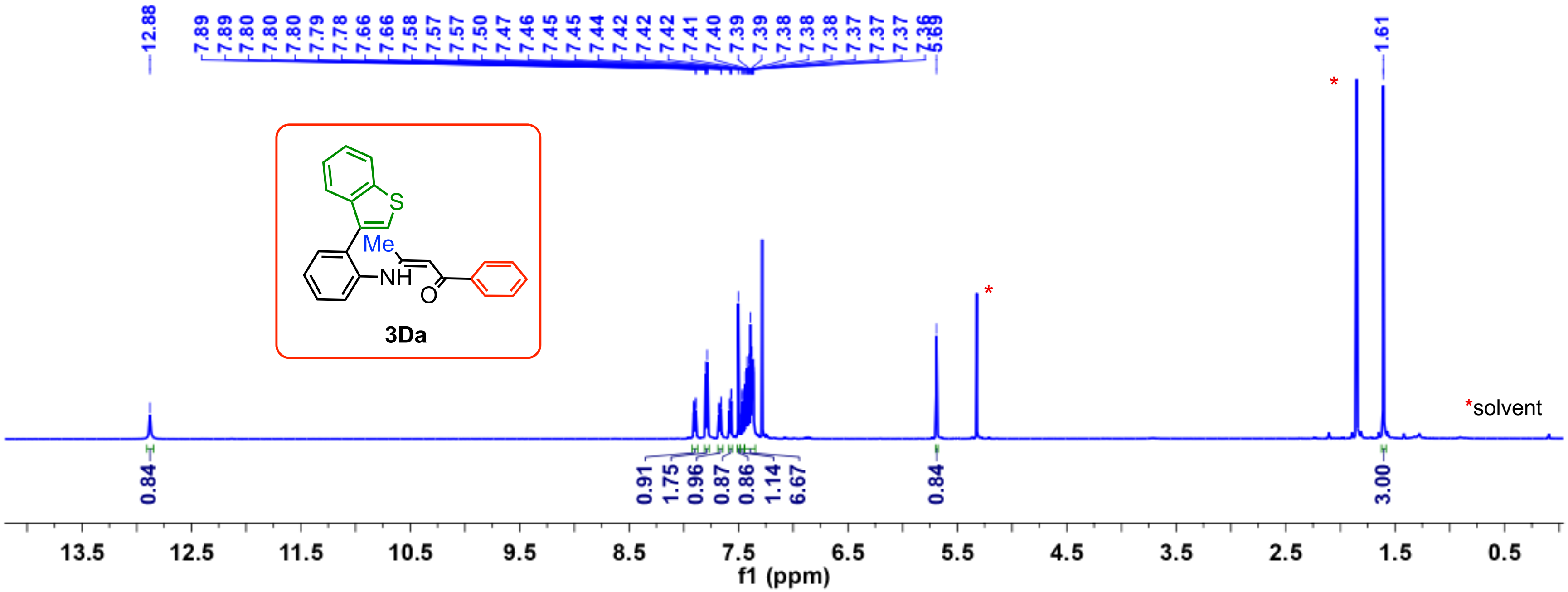

Figure S103: ^{1}H NMR of enaminone **3Da**.

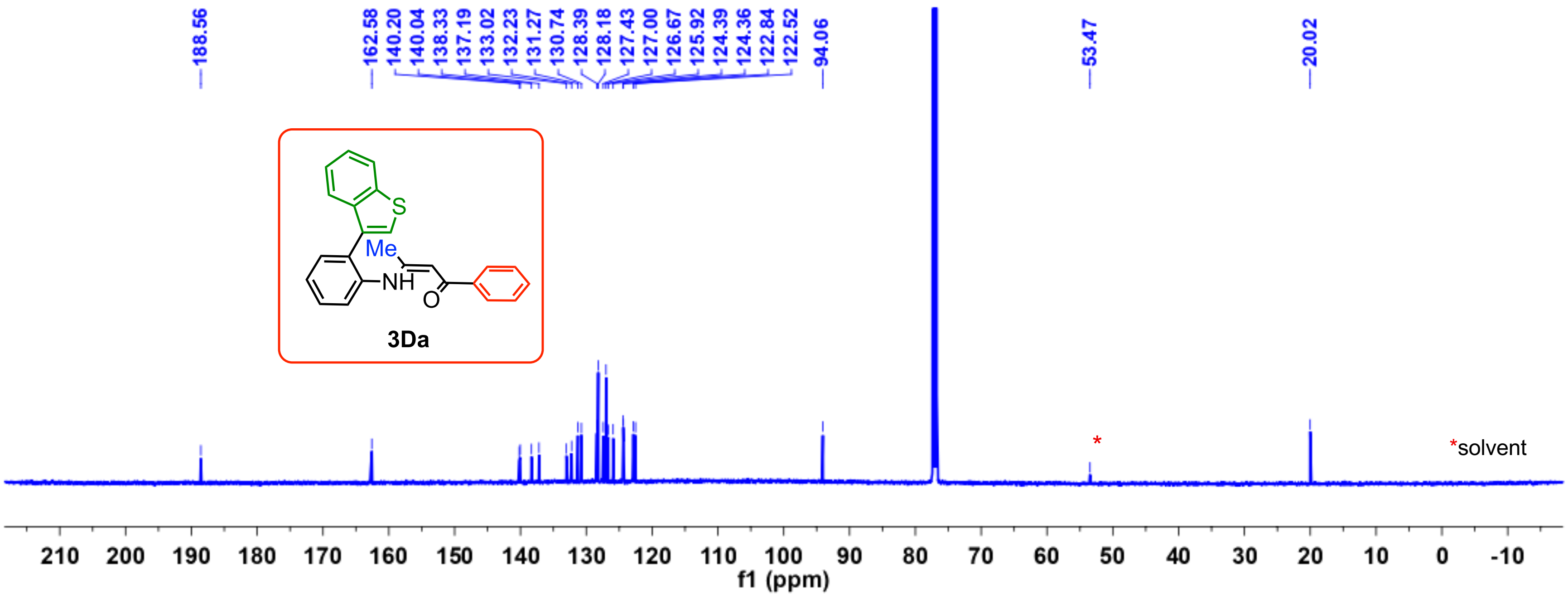

Figure S104: ^{13}C NMR of enaminone **3Da**.

HRMS-ESI (m/z) ([M + H]$^+$):

Calculated: 370.1266

Observed: 370.1257

|Δm| = 2.43 ppm

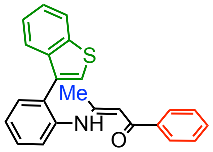

3Da

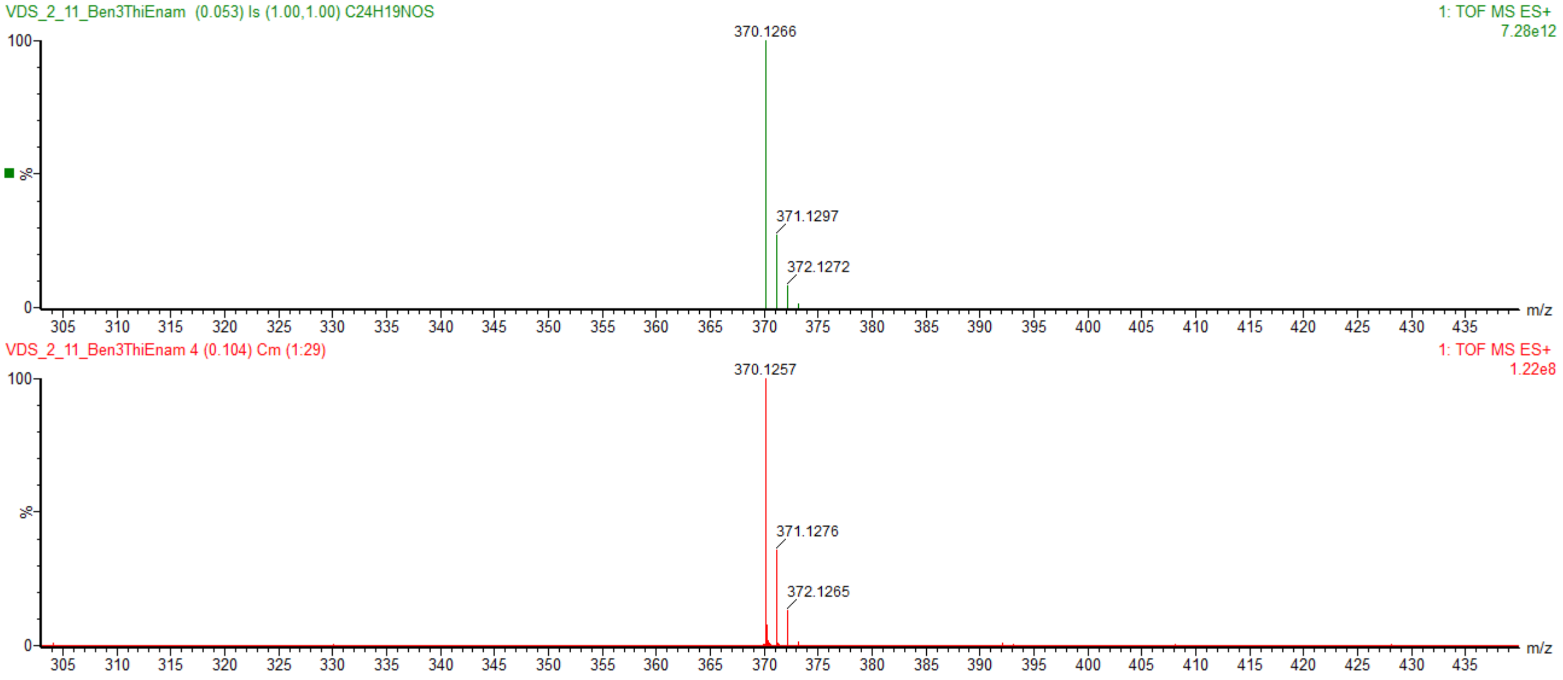

Figure S105: HRMS data of quinoline photoproduct **3Da**.

18.1. Characterization of enaminone **3Ea**.

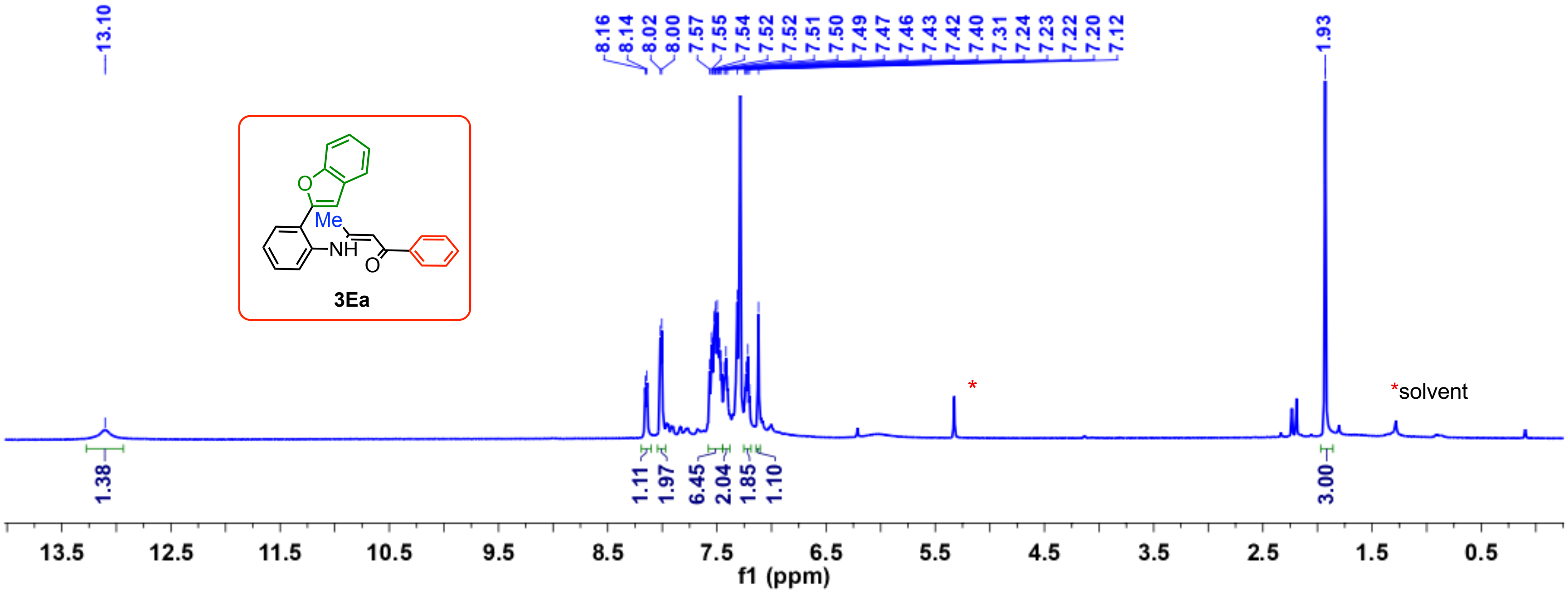

Figure S106: ^{1}H NMR of enaminone **3Ea**.

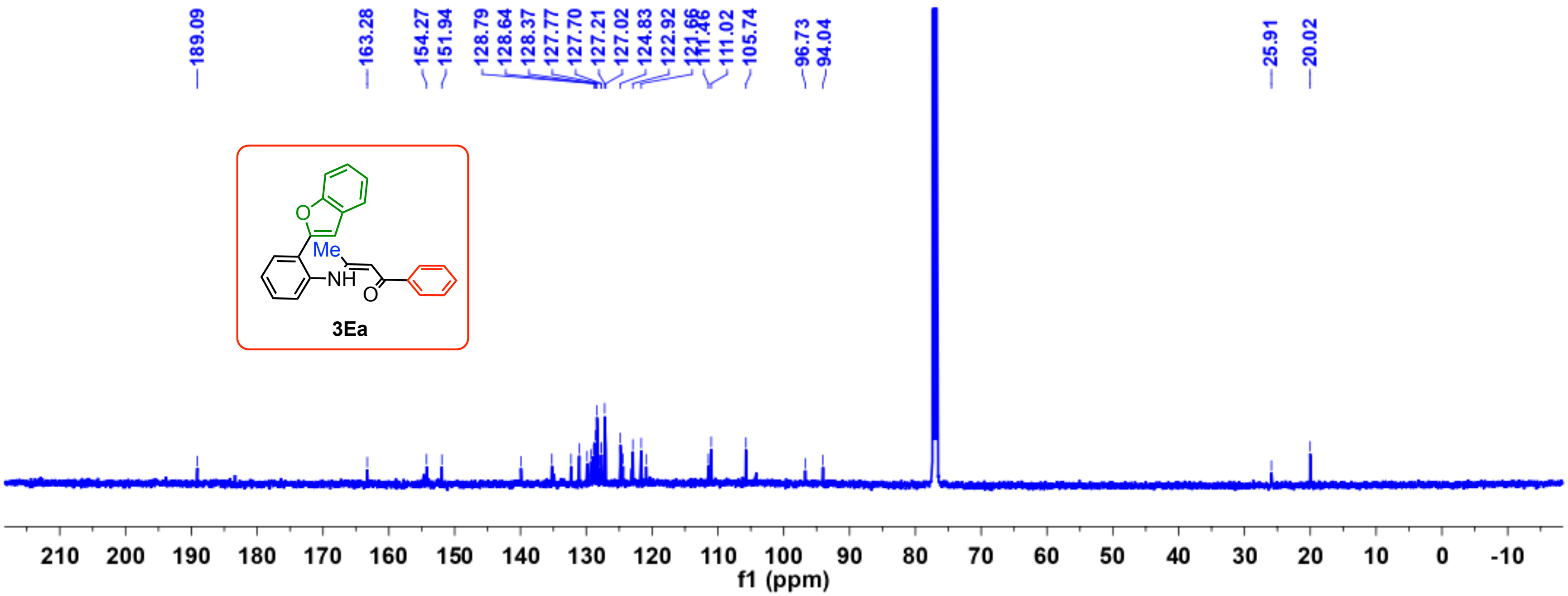

Figure S107: ^{13}C NMR of enaminone **3Ea**.

HRMS-ESI (m/z) ([M + H]$^+$):

Calculated: 354.1494

Observed: 354.1491

|Δm| = 0.85 ppm

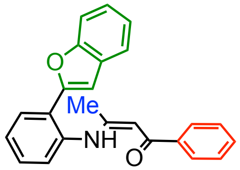

3Ea

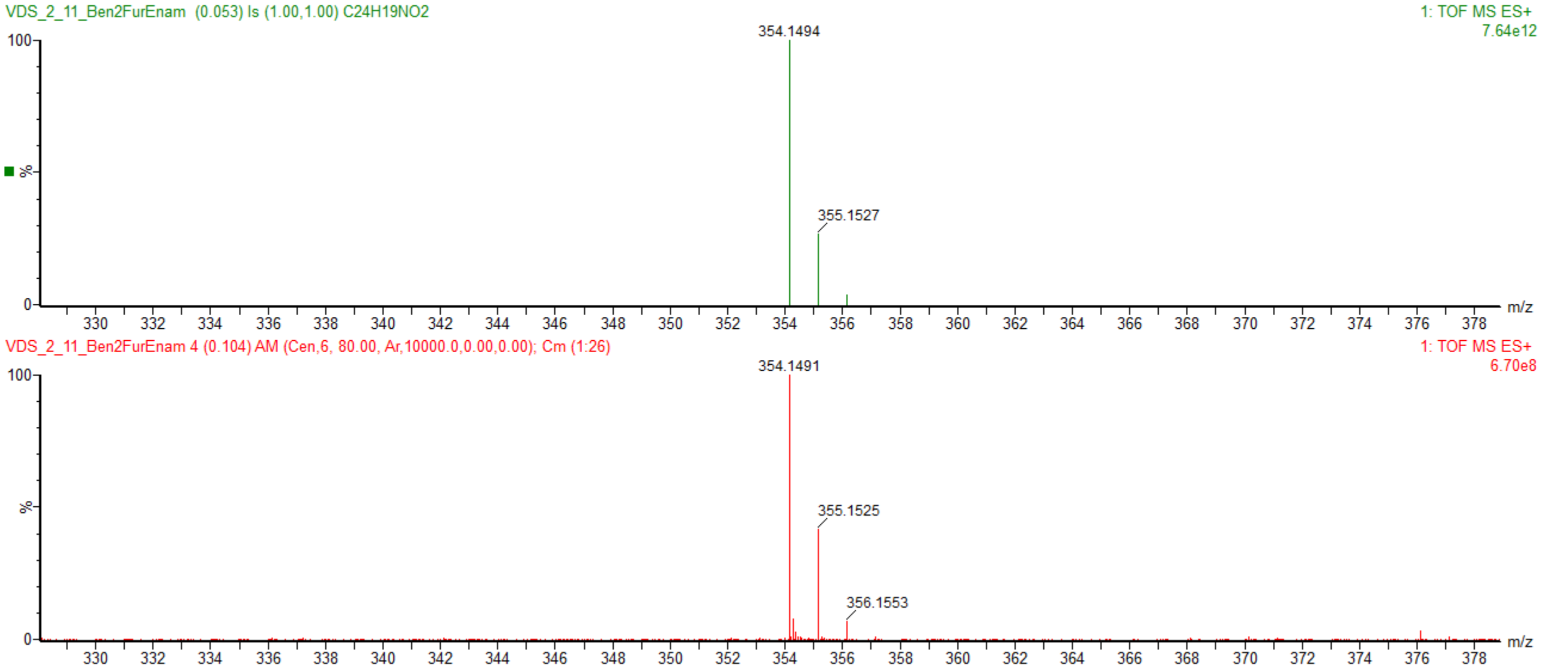

Figure S108: HRMS data of enaminone **3Ea**.

18.2. Characterization of enaminone **3Fa**.

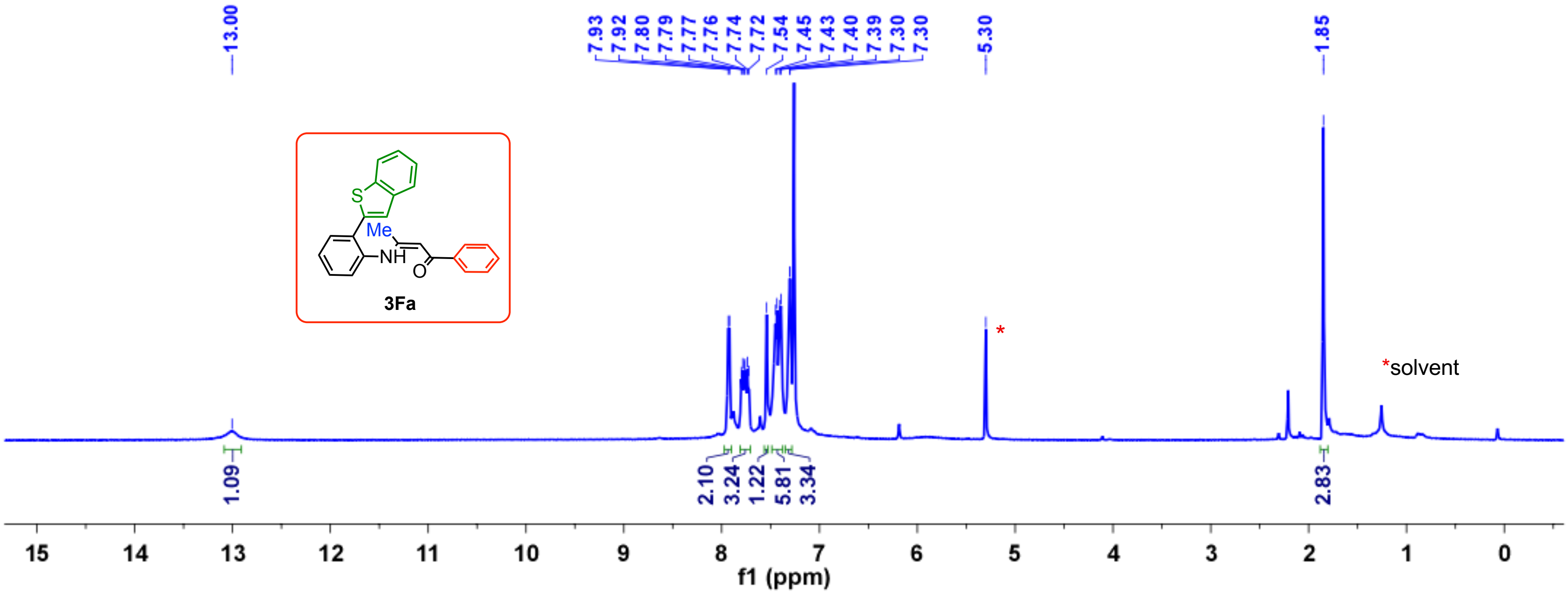

Figure S109: ^{1}H NMR of enaminone **3Fa**.

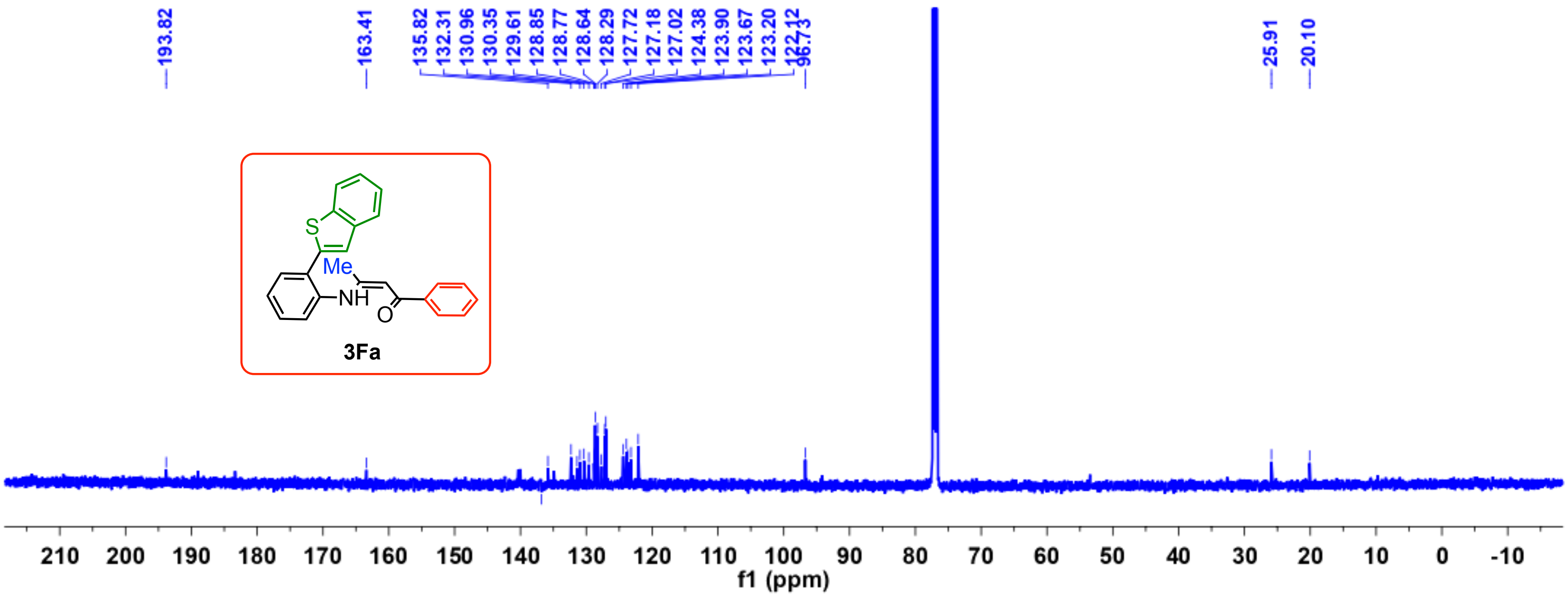

Figure S110: ^{13}C NMR of enaminone **3Fa**.

HRMS-ESI (m/z) ([M + H]$^+$):

Calculated: 370.1266

Observed: 370.1264

|Δm| = 0.54 ppm

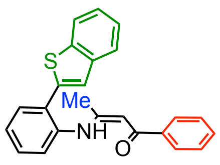

3Fa

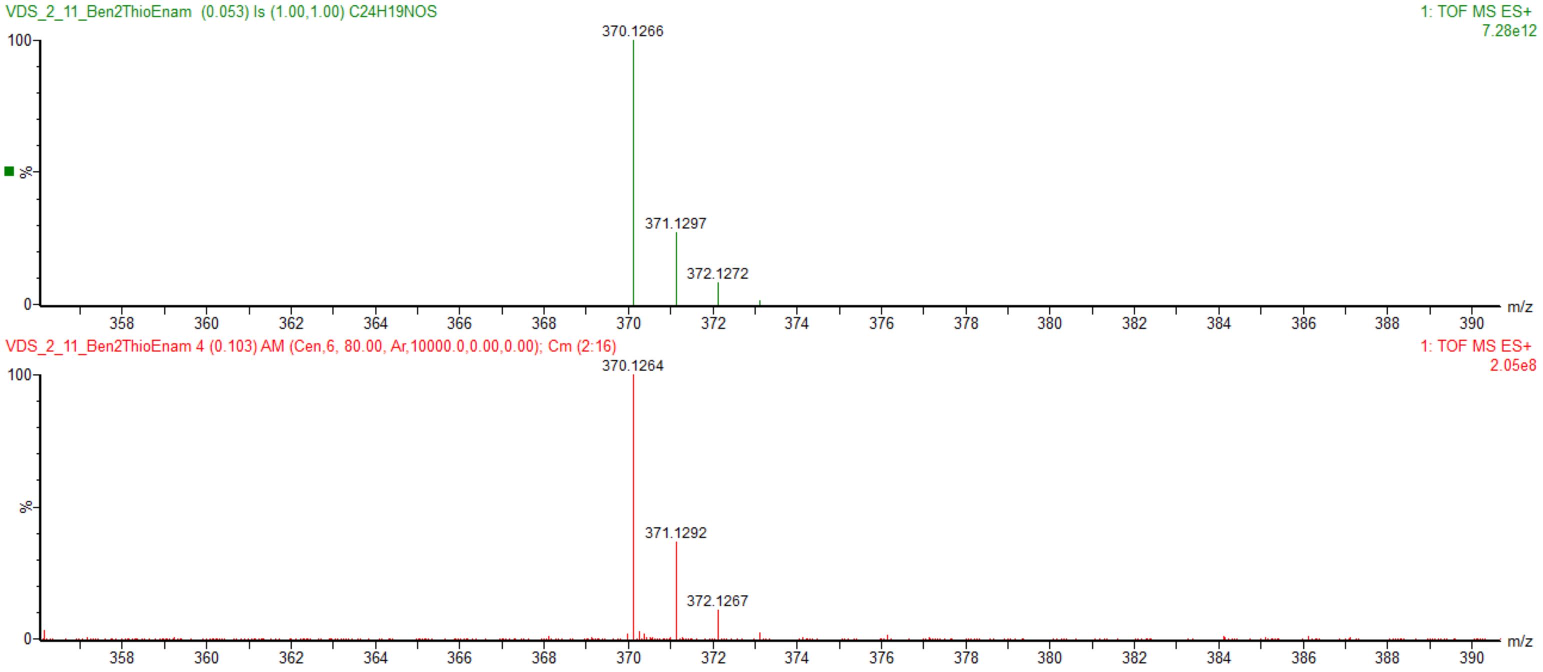

Figure S111: HRMS data of enaminone **3Fa**.

18.3. Characterization of enaminone **3Ga**.

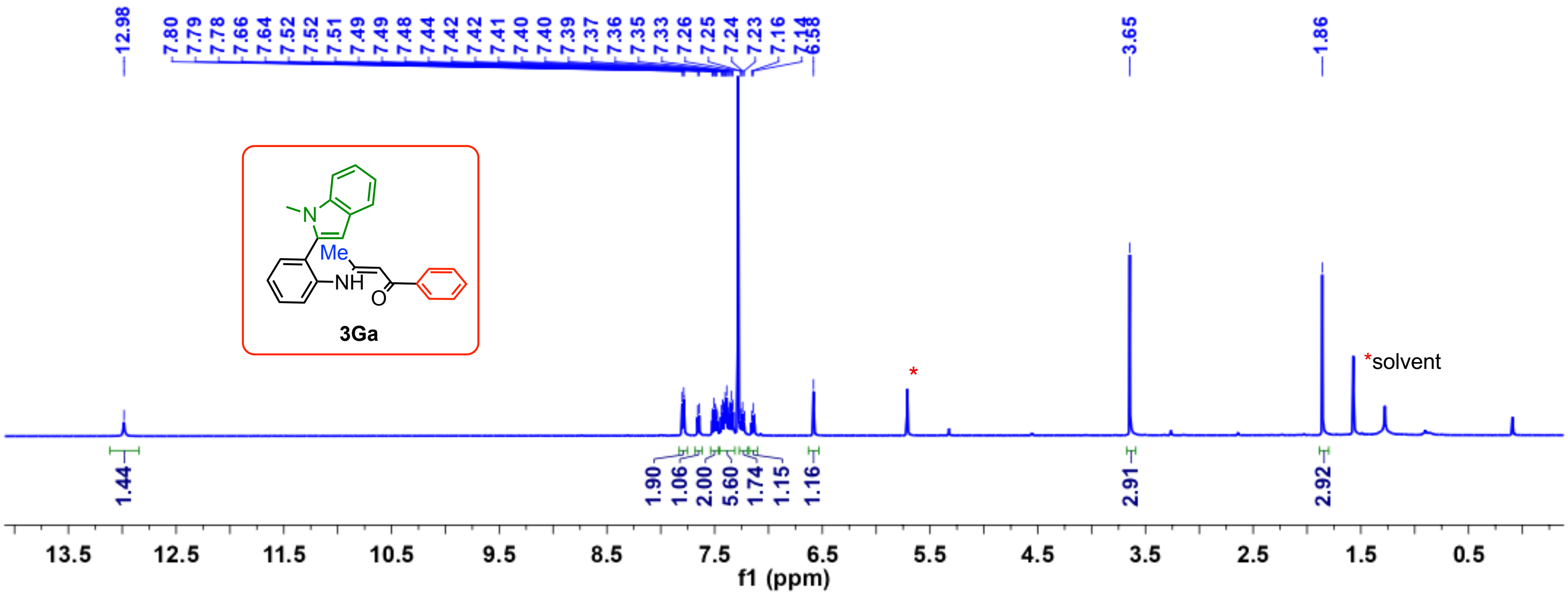

Figure S112: ^{1}H NMR of enaminone **3Ga**.

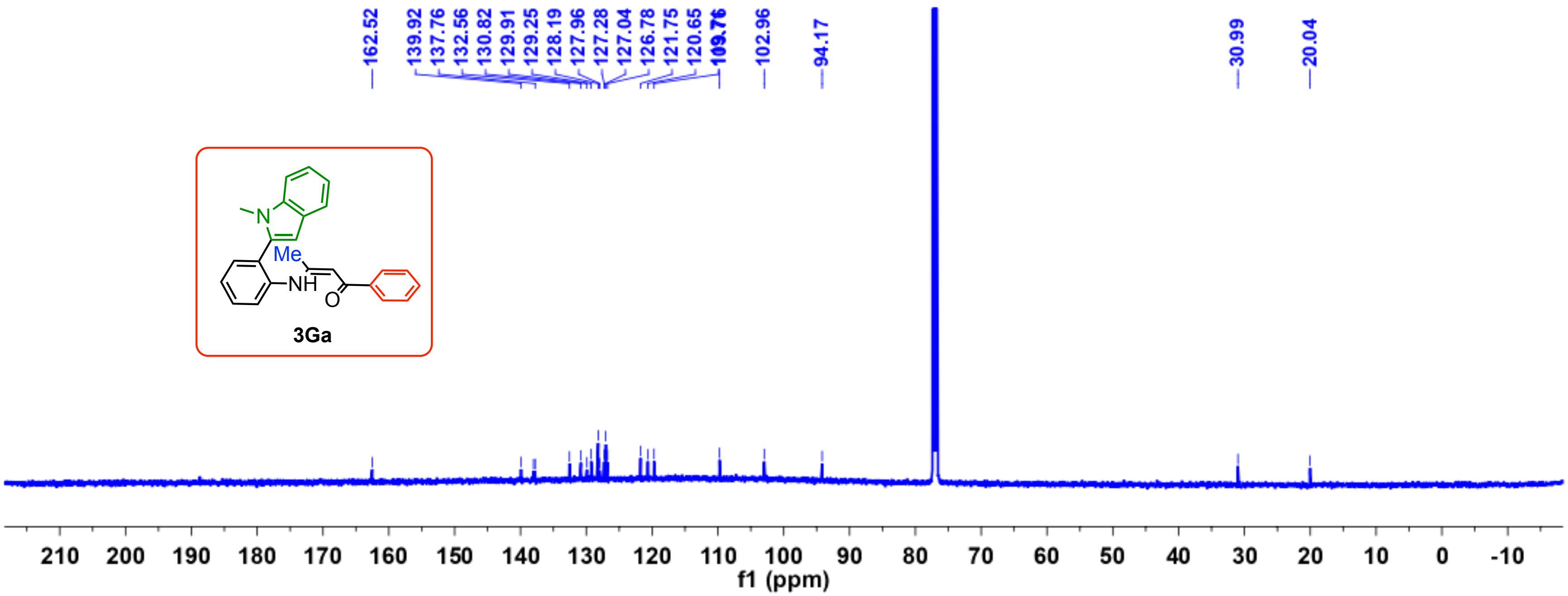

Figure S113: ^{13}C NMR of enaminone **3Ga**.

HRMS-ESI (m/z) ([M + H]$^+$):

Calculated: 367.1810

Observed: 367.1821

$|\Delta m|$ = 2.99 ppm

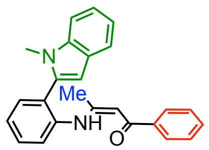

3Ga

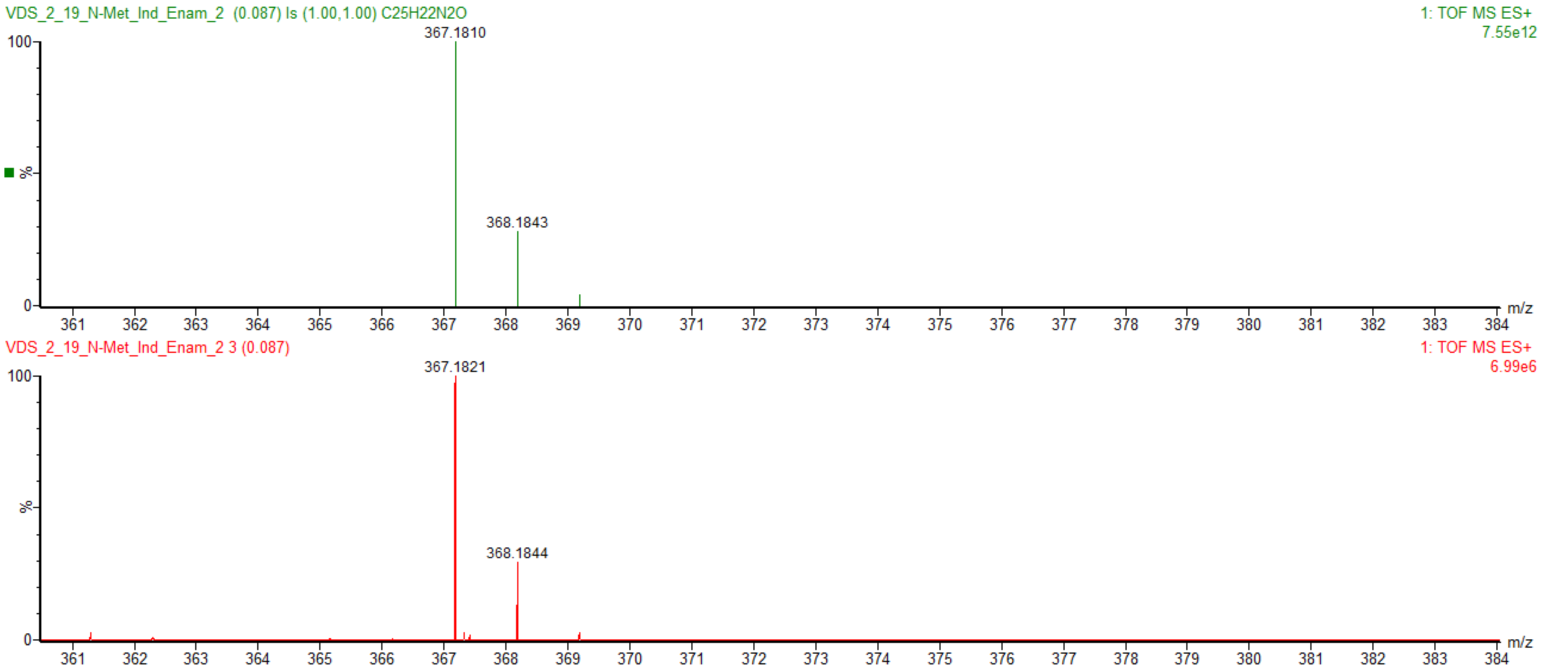

Figure S114: HRMS data of enaminone **3Ga**.

18.4. Characterization of enaminone **3Bb**.

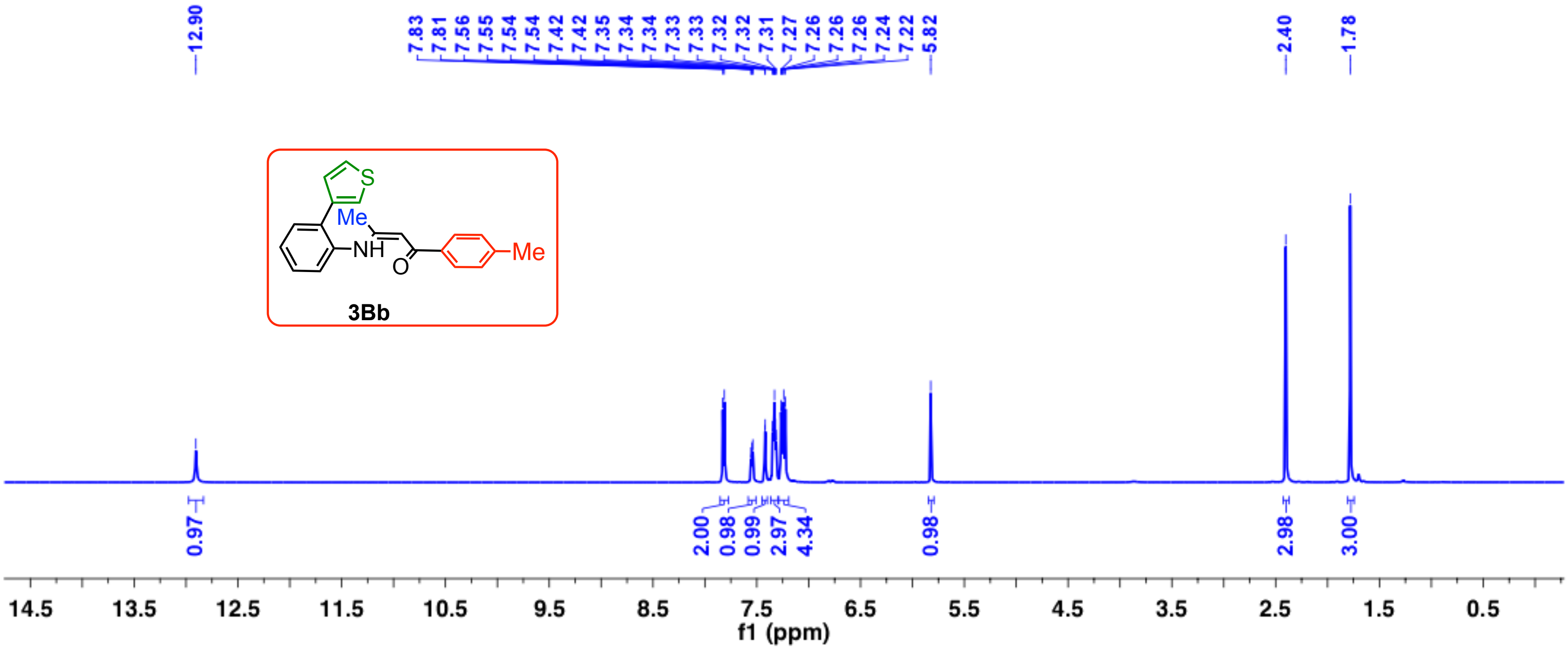

Figure S115: ^{1}H NMR of enaminone **3Bb**.

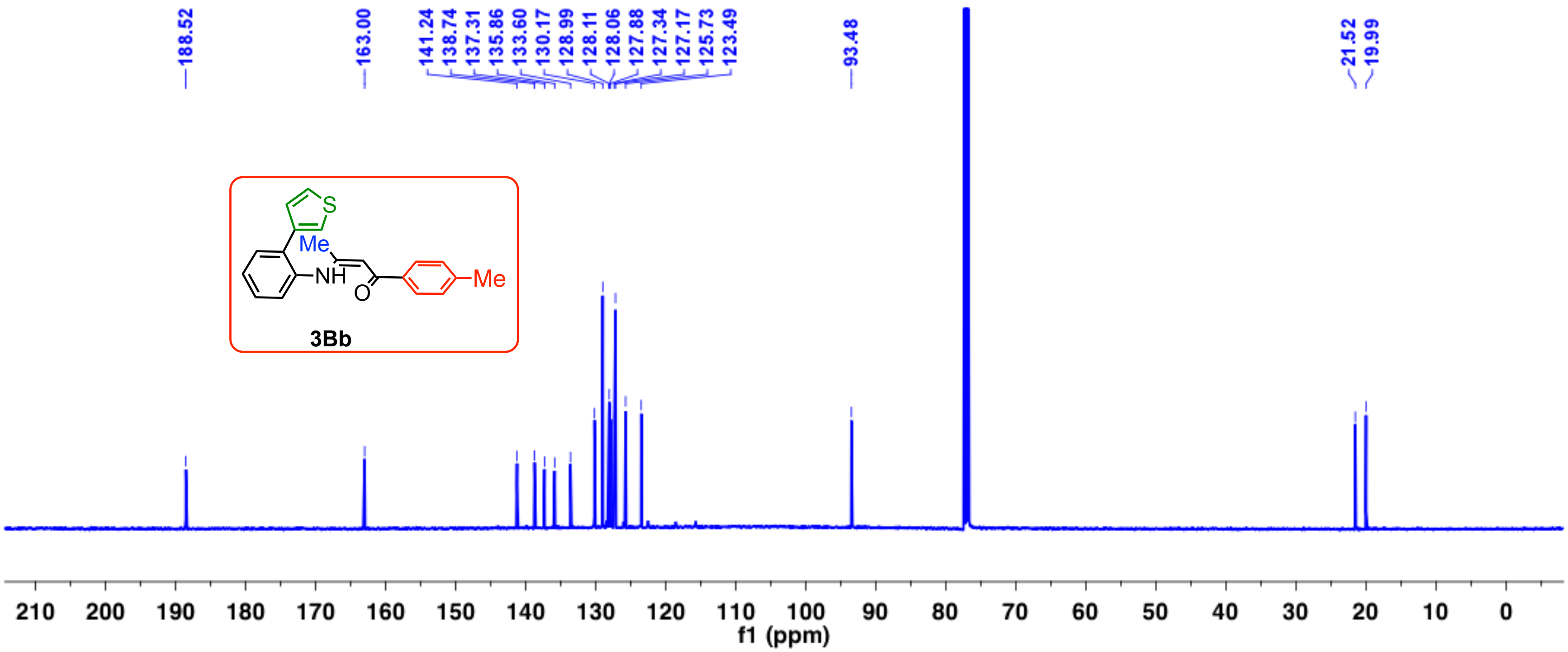

Figure S116: ^{13}C NMR of enaminone **3Bb**.

HRMS-ESI (m/z) ([M + H]$^+$):

Calculated: 334.1266

Observed: 334.1278

|Δm| = 3.6 ppm

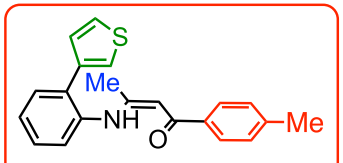

3Bb

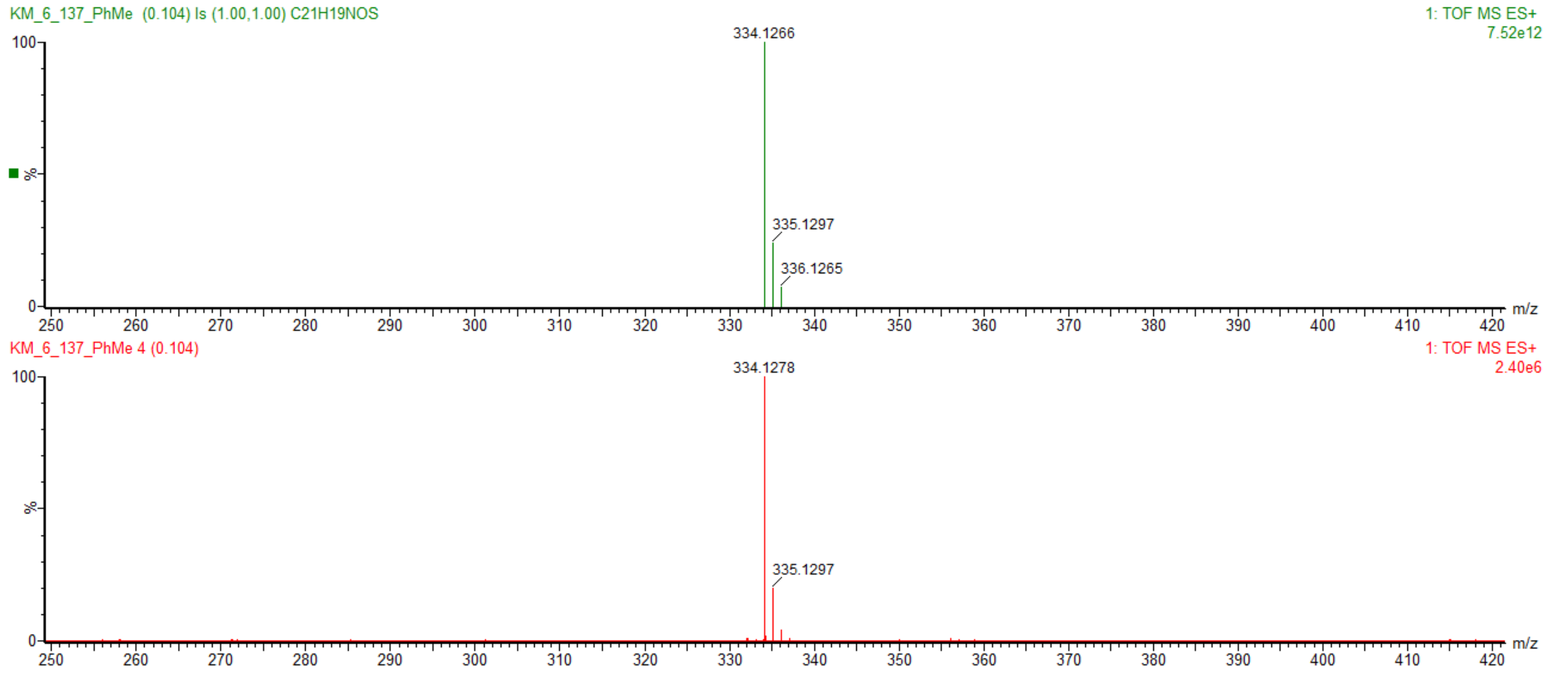

Figure S117: HRMS data of enaminone **3Bb**.

18.1. Characterization of enaminone **3Bc**.

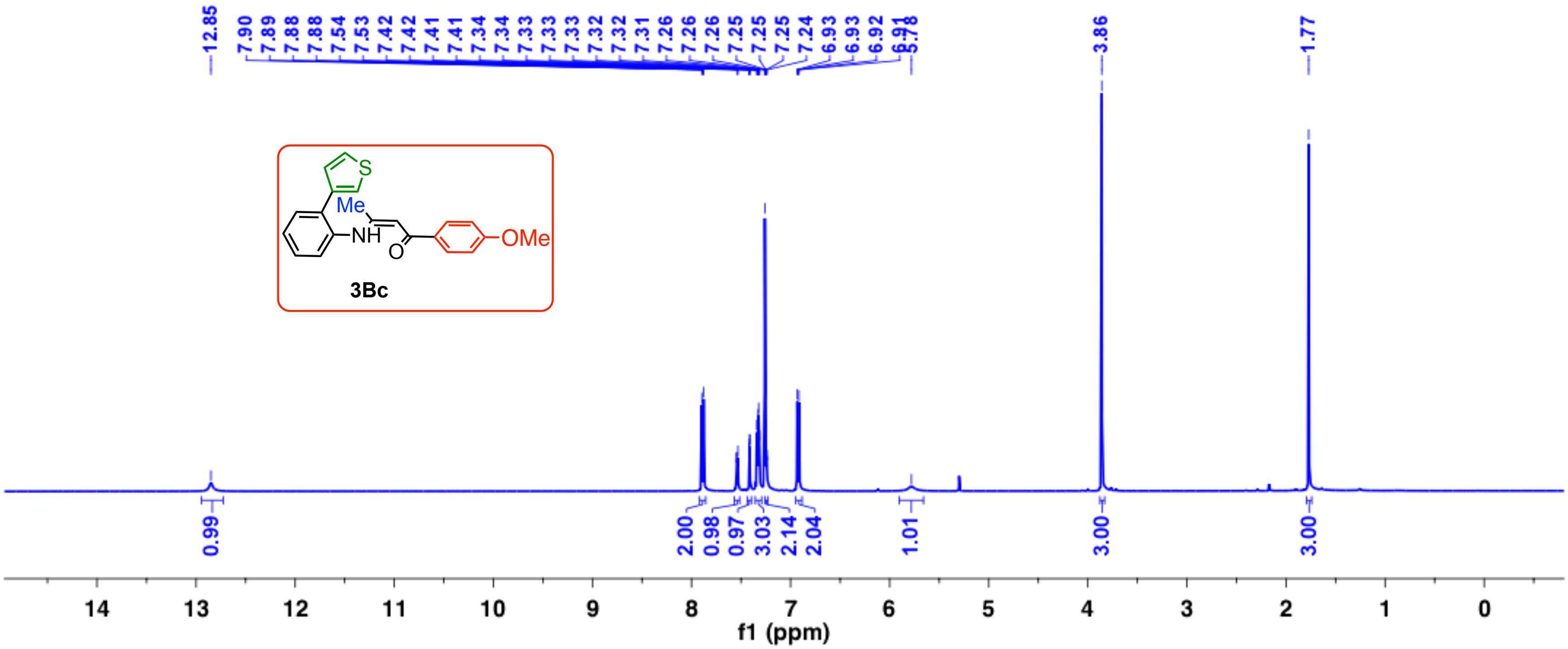

Figure S118: ^{1}H NMR of enaminone **3Bc**.

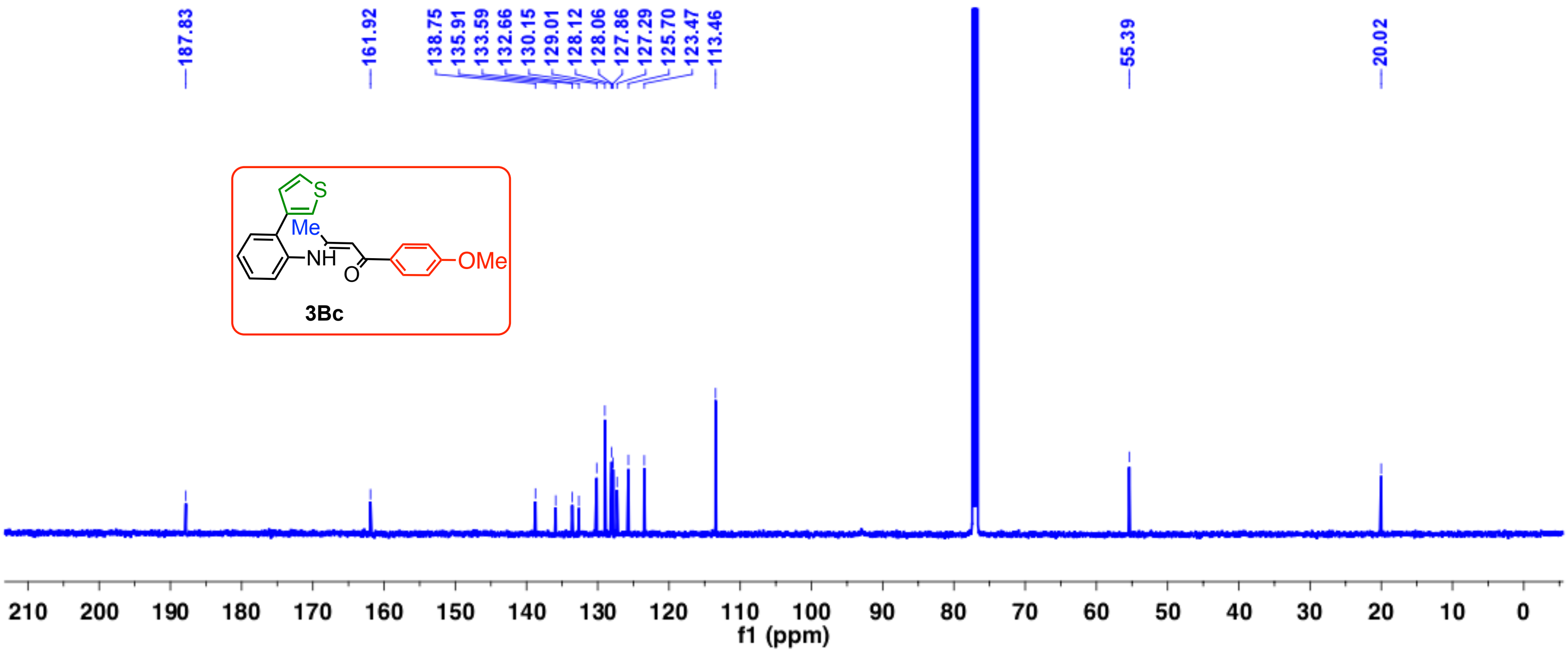

Figure S119: ^{13}C NMR of enaminone **3Bc**.

HRMS-ESI (m/z) ([M + H]$^+$):

Calculated: 350.1215

Observed: 350.1211

|Δm| = 1.14 ppm

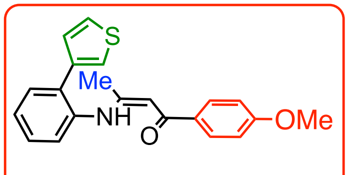

3Bc

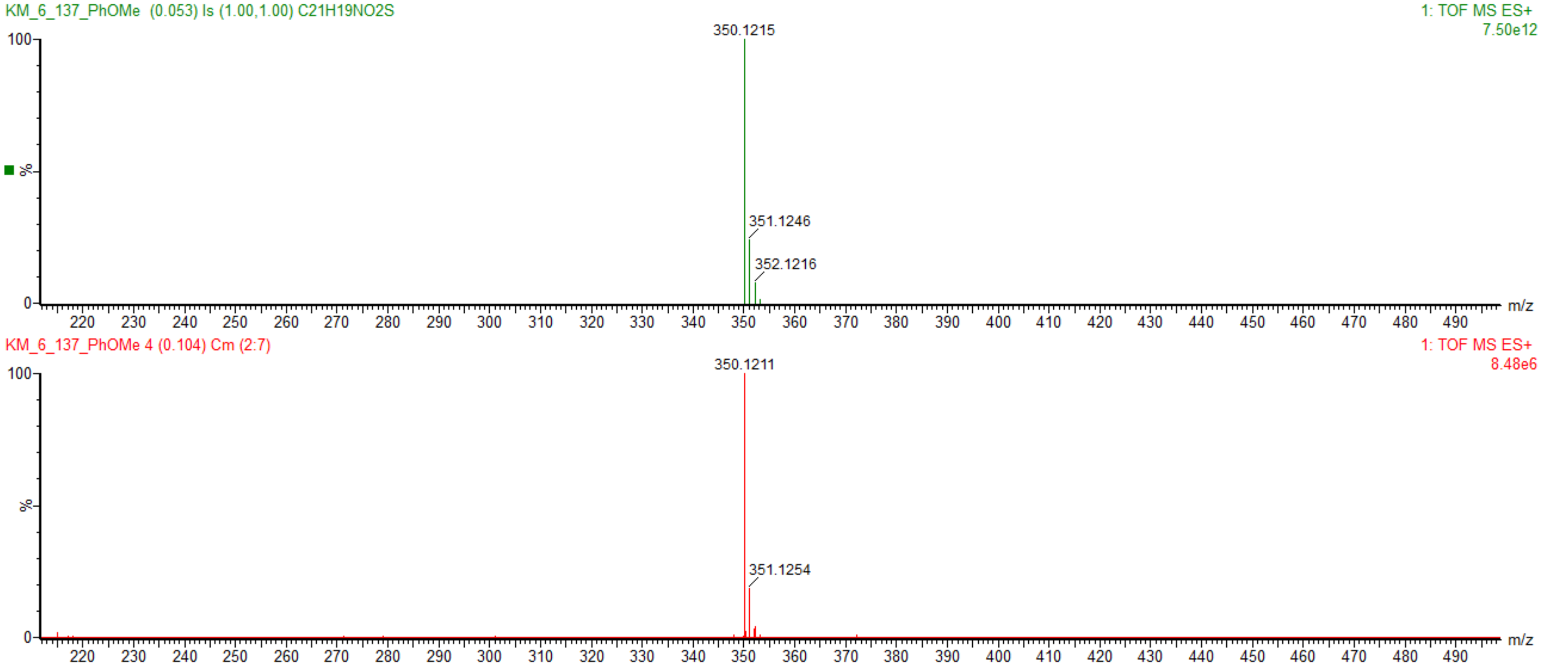

Figure S120: HRMS data of enaminone **3Bc**.

18.2. Characterization of enaminone **3Bd**.

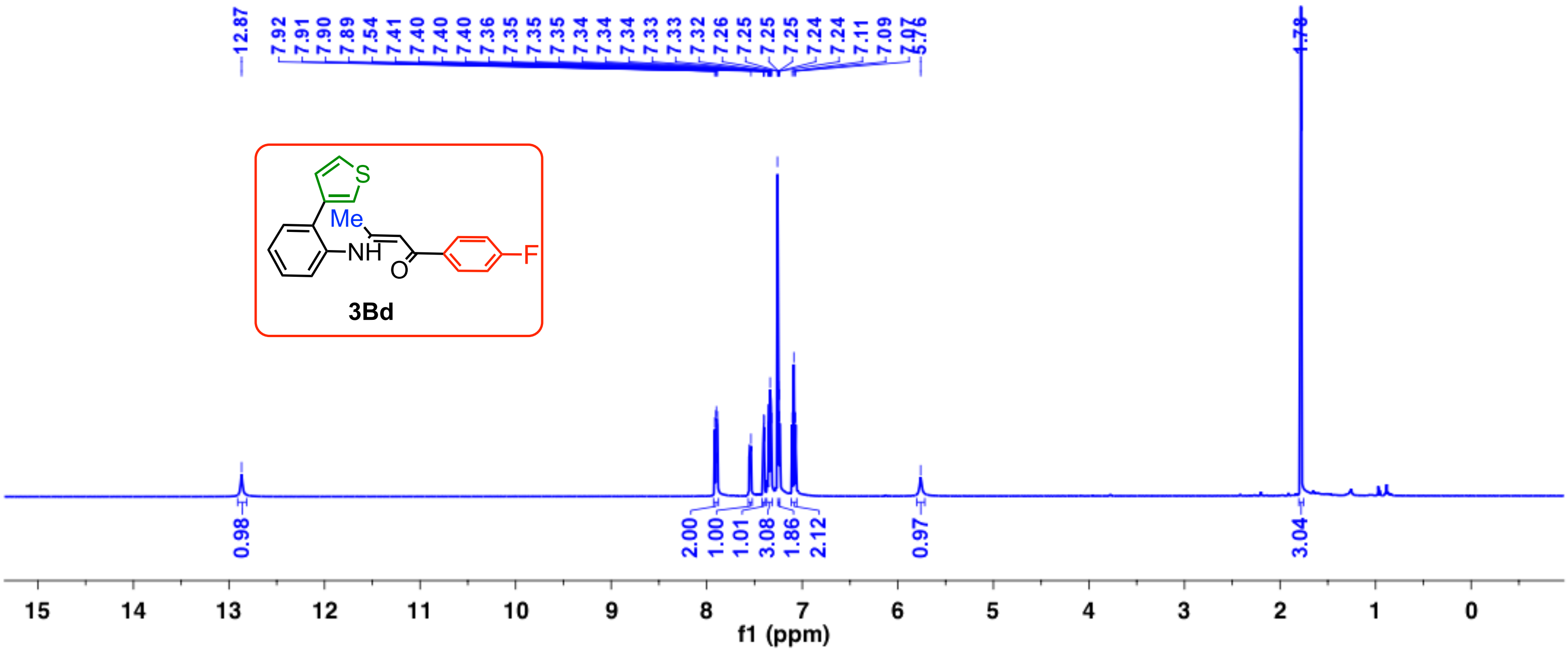

Figure S121: ^{1}H NMR of enaminone **3Bd**.

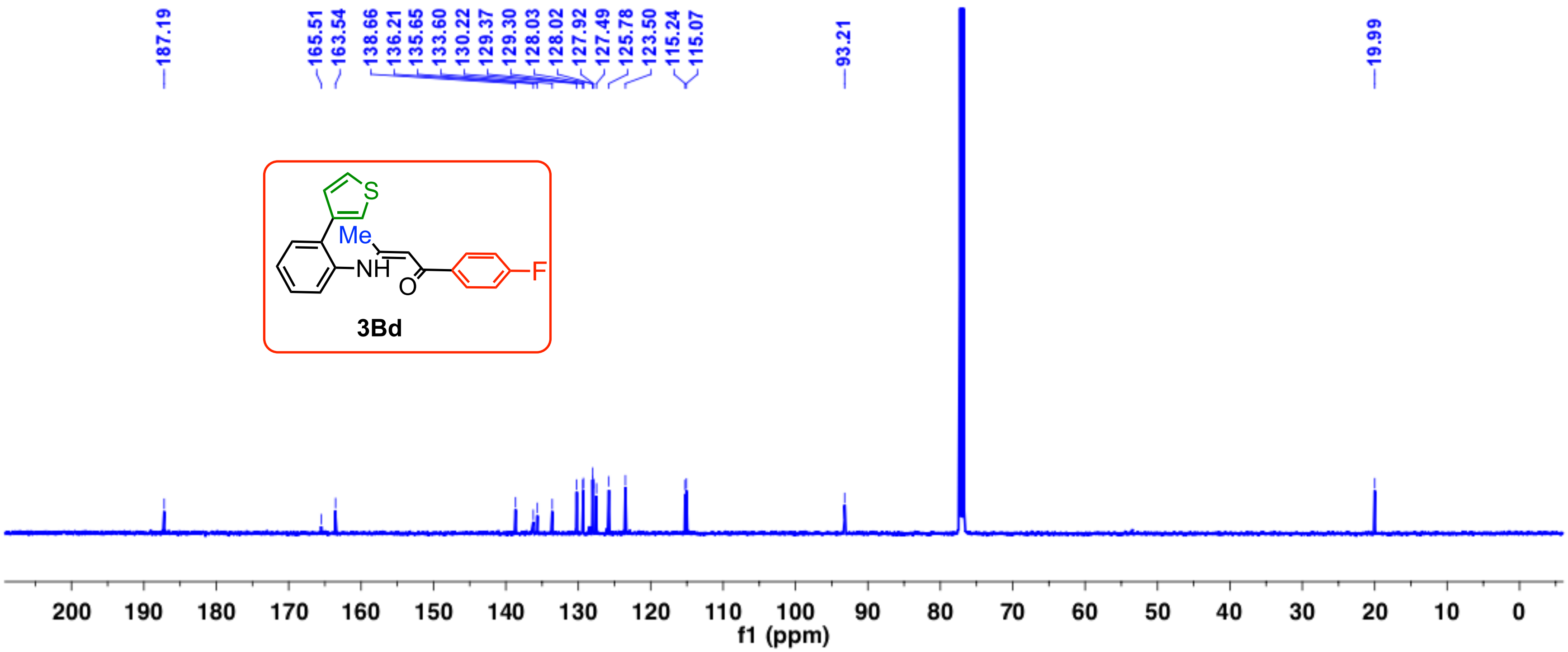

Figure S122: ^{13}C NMR of enaminone **3Bd**.

HRMS-ESI (m/z) ([M + H]$^+$):

Calculated: 338.1015

Observed: 338.1025

|Δm| = 2.95 ppm

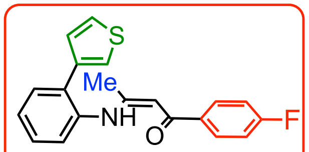

3Bd

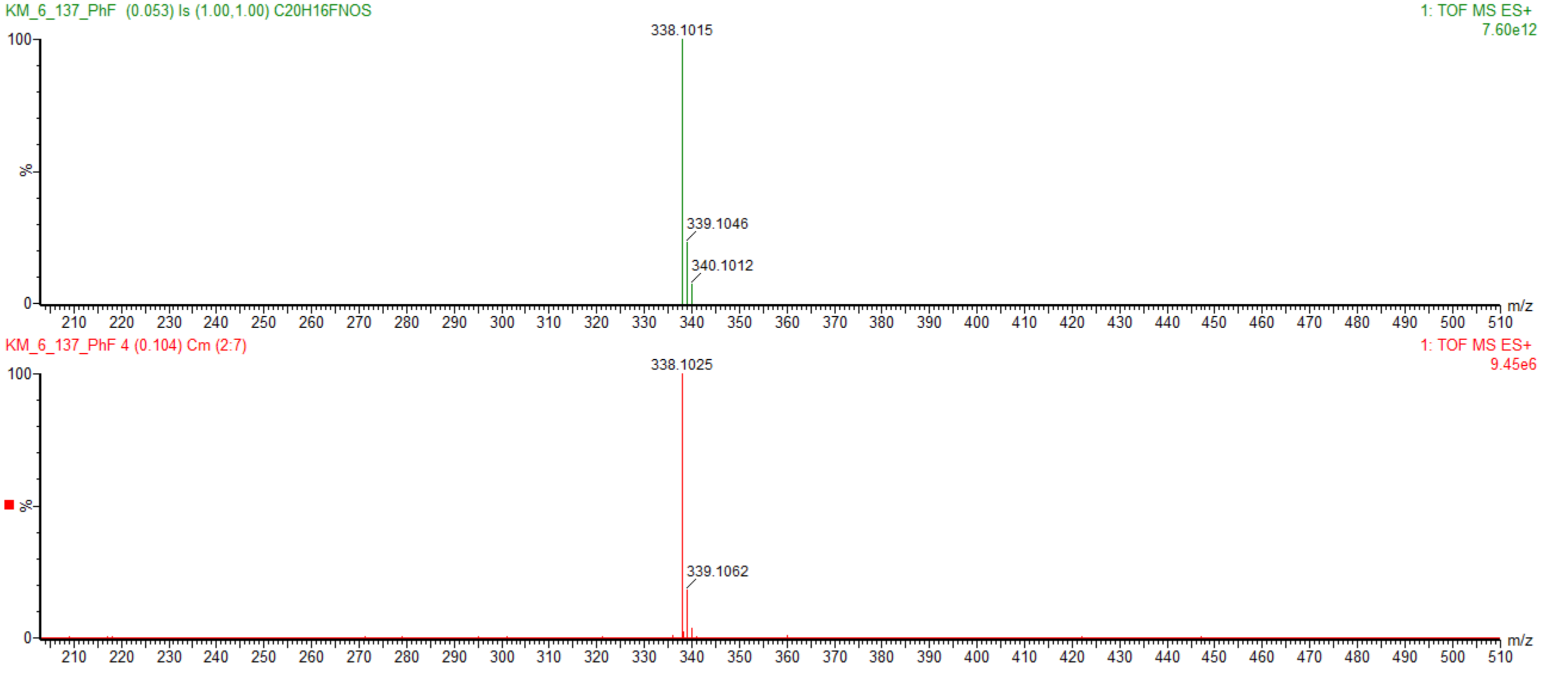

Figure S123: HRMS data of enaminone **3Bd**.

18.3. Characterization of enaminone **3Be**.

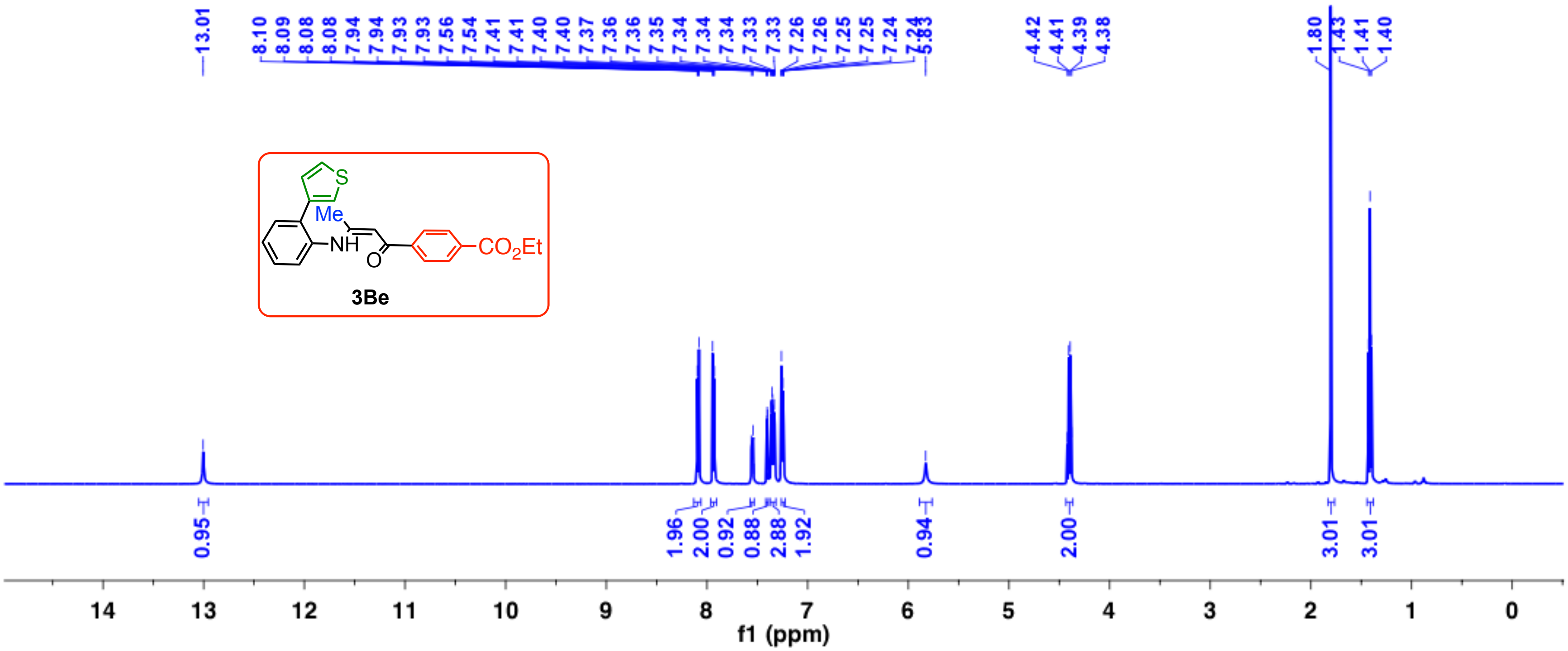

Figure S124: ^{1}H NMR of enaminone **3Be**.

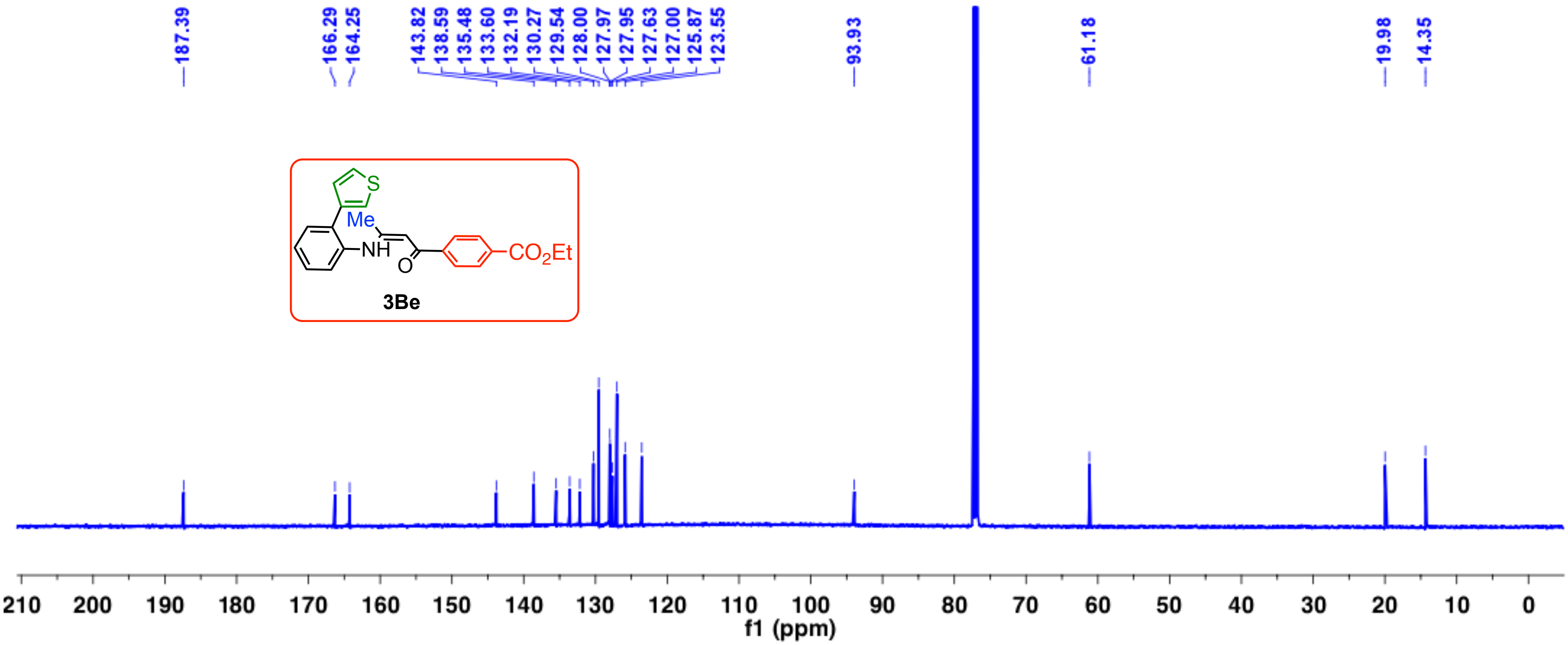

Figure S125: ^{13}C NMR of enaminone **3Be**.

HRMS-ESI (m/z) ([M + H]$^+$):

Calculated: 392.1320

Observed: 392.1310

$|\Delta m|$ = 2.5 ppm

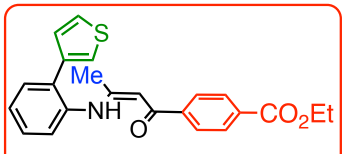

3Be

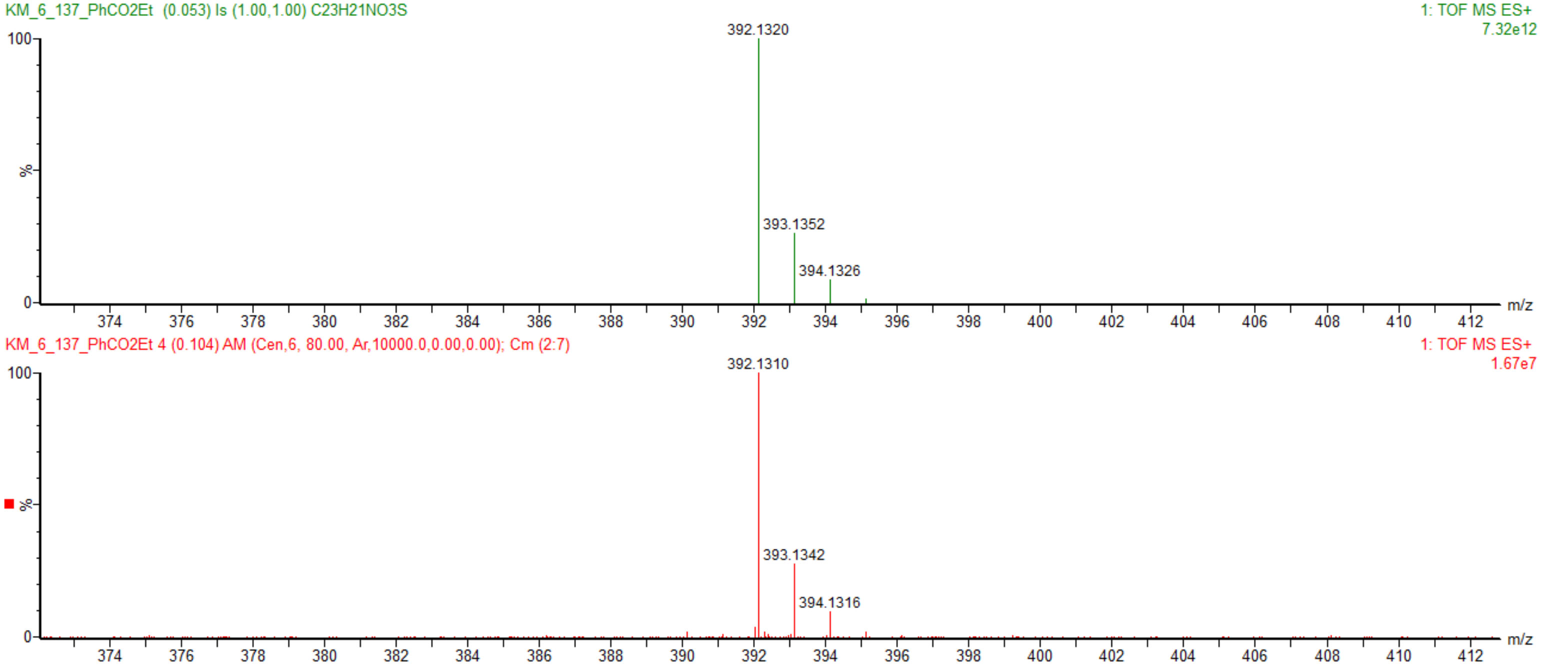

Figure S126: HRMS data of enaminone **3Be**.

18.1. Characterization of enaminone **3Bf**.

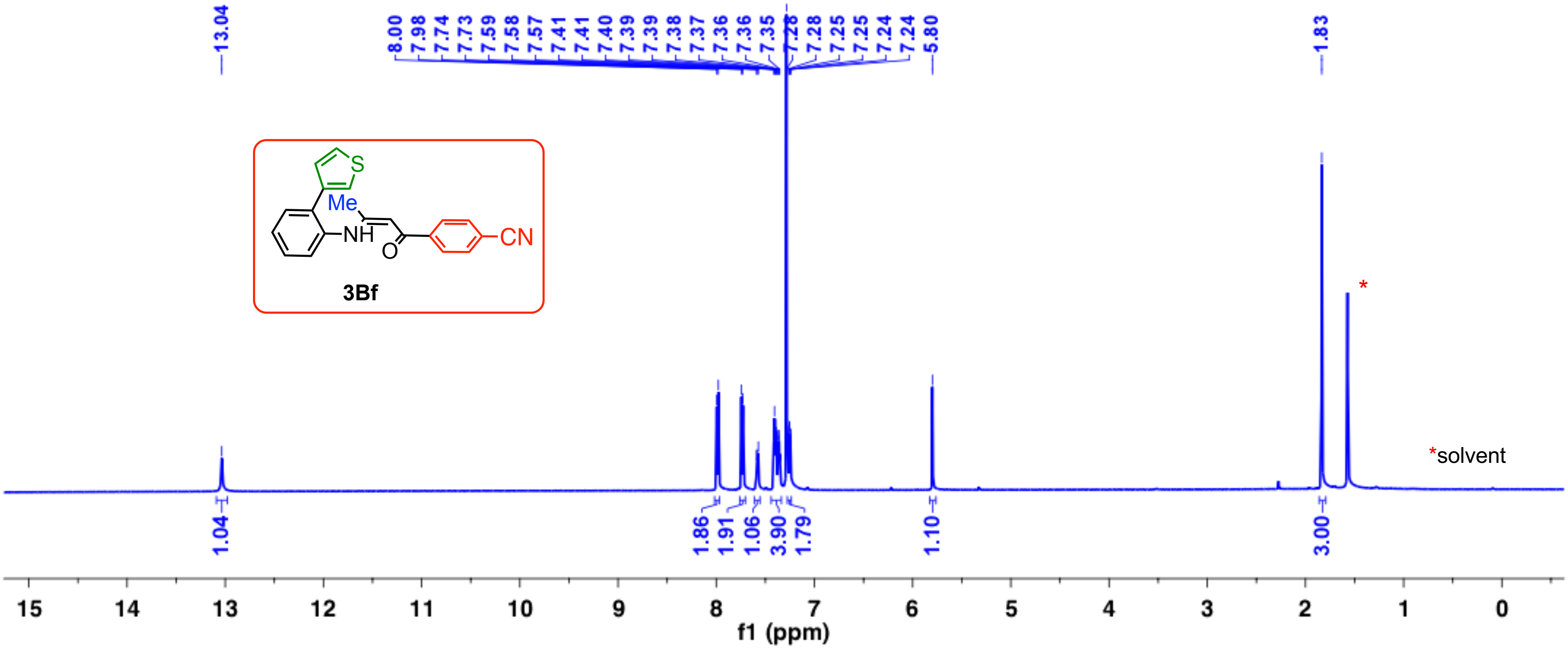

Figure S127: ^{1}H NMR of enaminone **3Bf**.

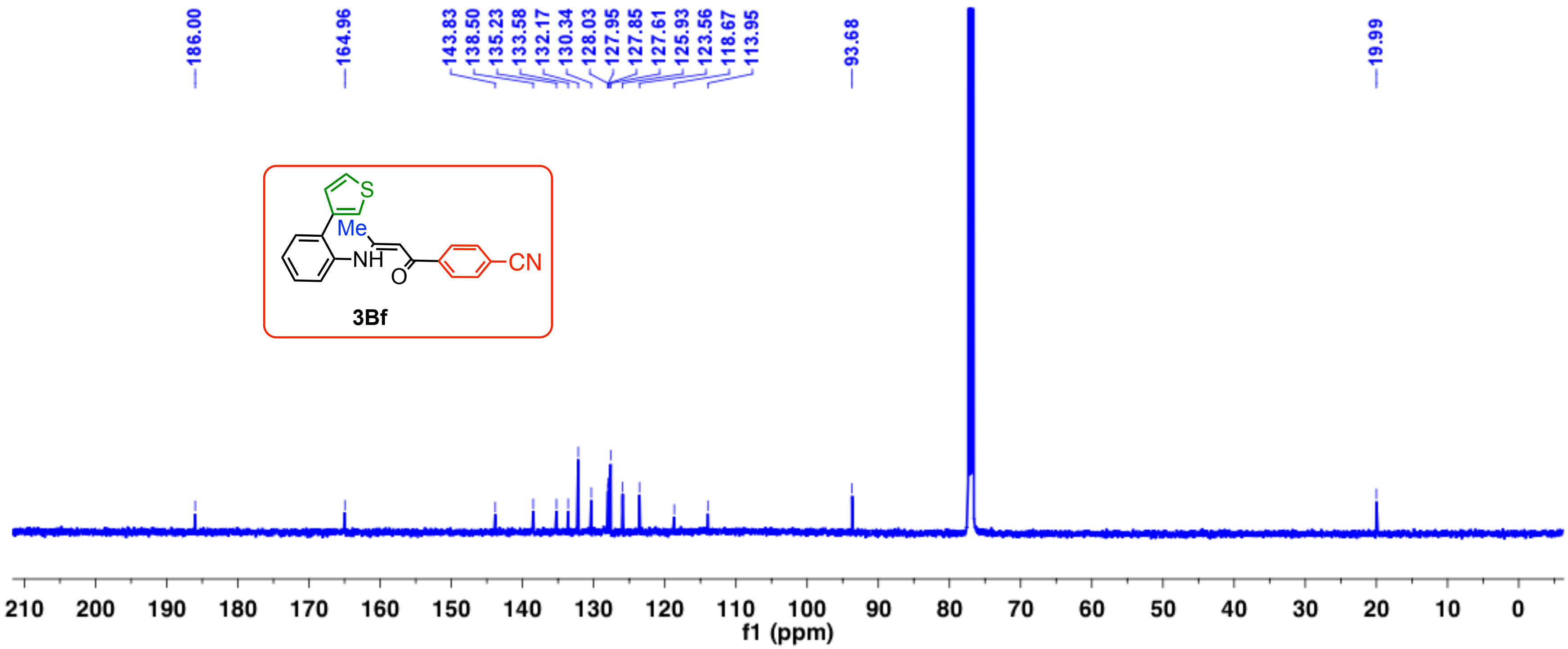

Figure S128: ^{13}C NMR of enaminone **3Bf**.

HRMS-ESI (m/z) ([M + H]$^+$):

Calculated: 345.1062

Observed: 345.1063

|Δm| = 0.3 ppm

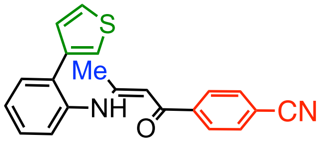

3Bf

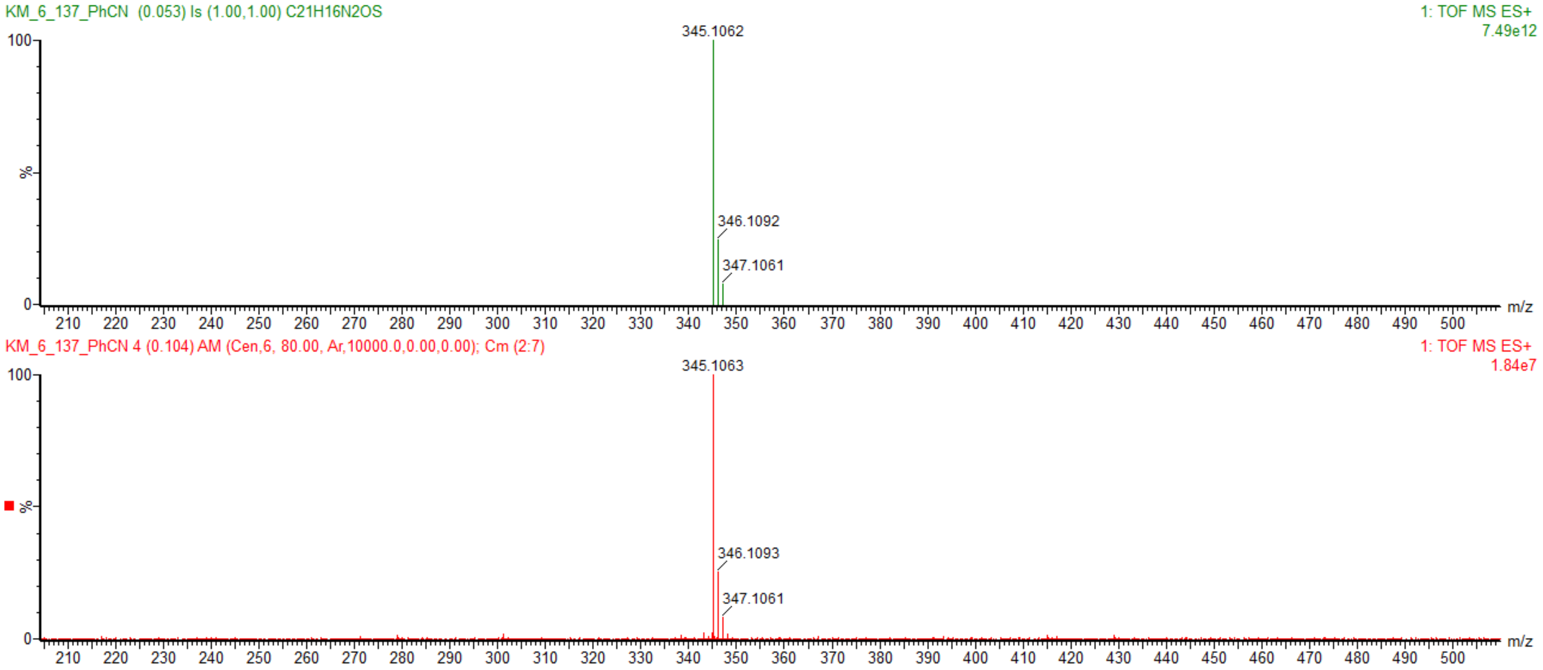

Figure S129: HRMS data of enaminone **3Bf**.

18.1. Characterization of enaminone **3Bg**.

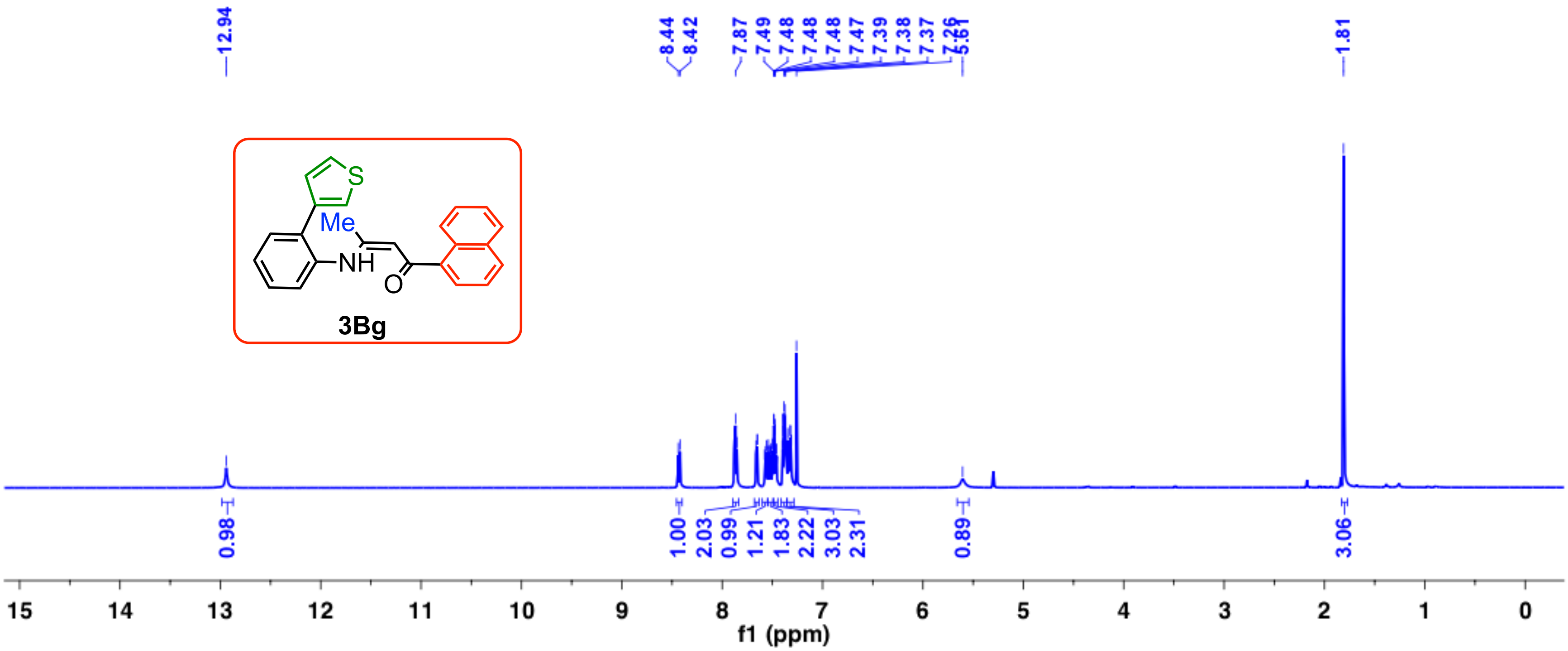

Figure S130: ^{1}H NMR of enaminone **3Bg**.

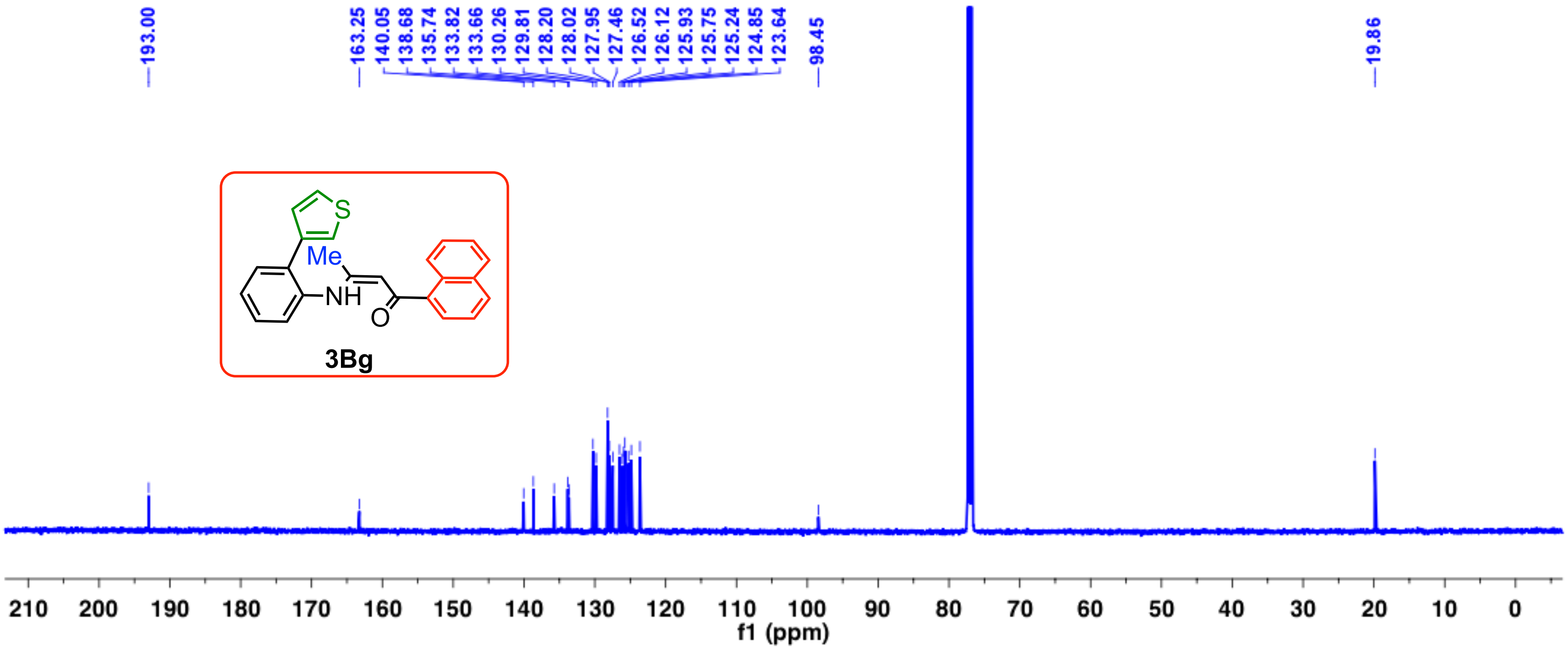

Figure S131: ^{1}H NMR of enaminone **3Bg**.

HRMS-ESI (m/z) ([M + H]$^+$):

Calculated: 370.1266

Observed: 370.1257

|Δm| = 2.43 ppm

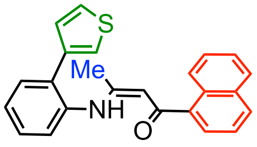

3Bg

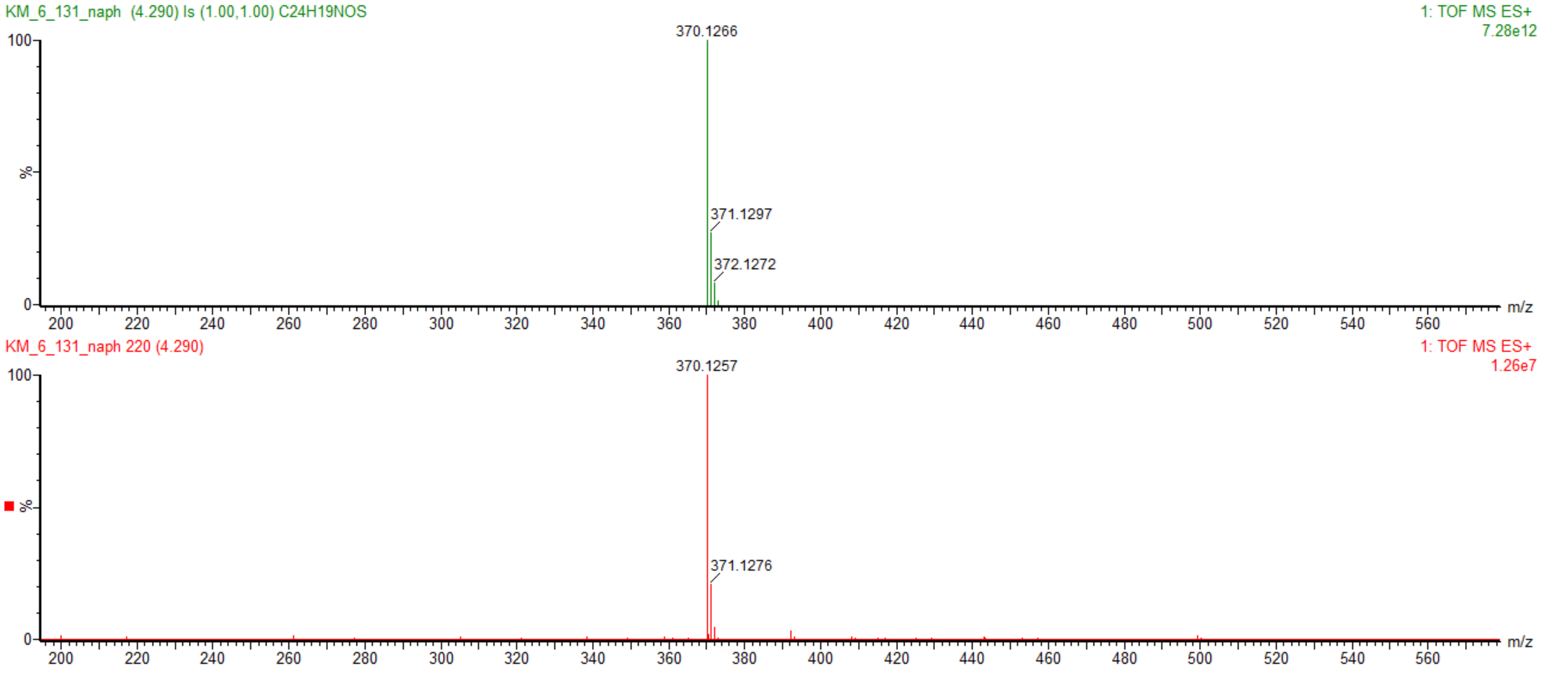

Figure S132: HRMS data of enaminone **3Bg**.

18.2. Characterization of enaminone **3Bh**.

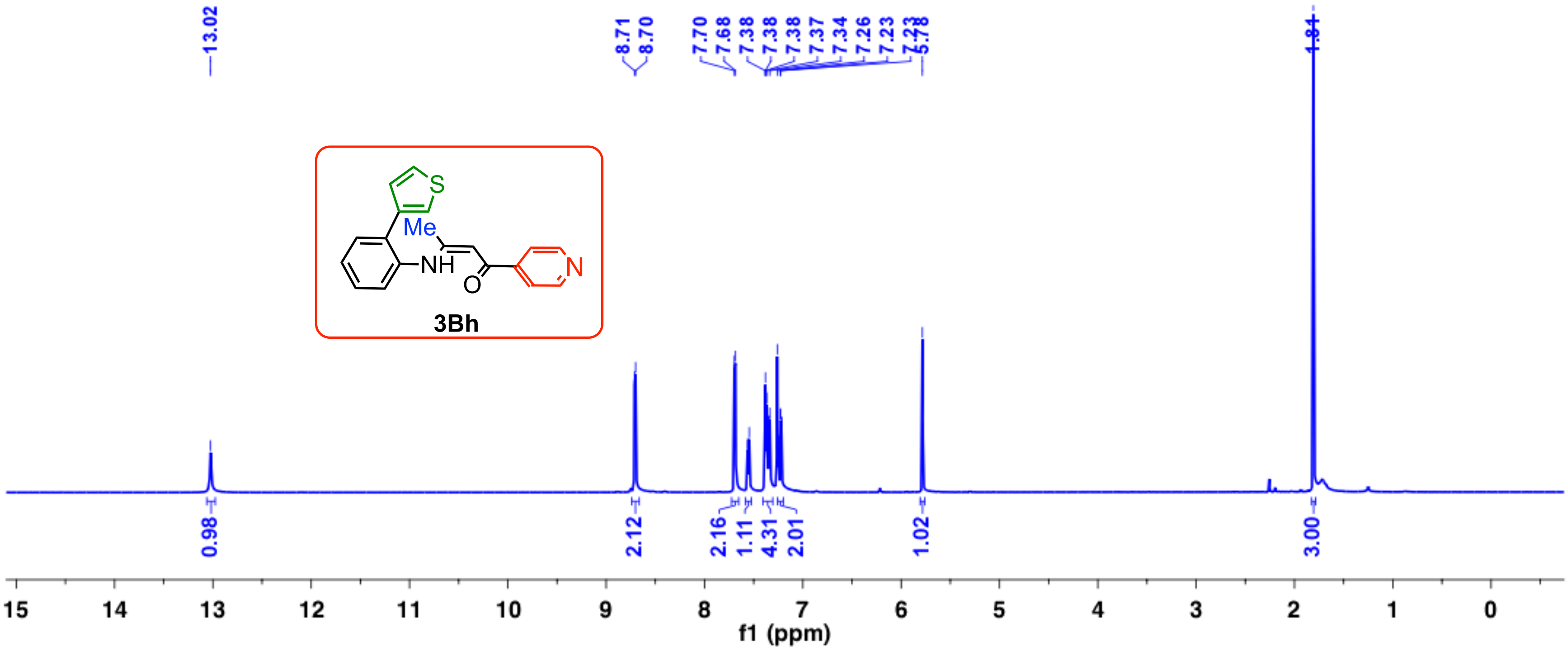

Figure S133: ^{1}H NMR of enaminone **3Bh**.

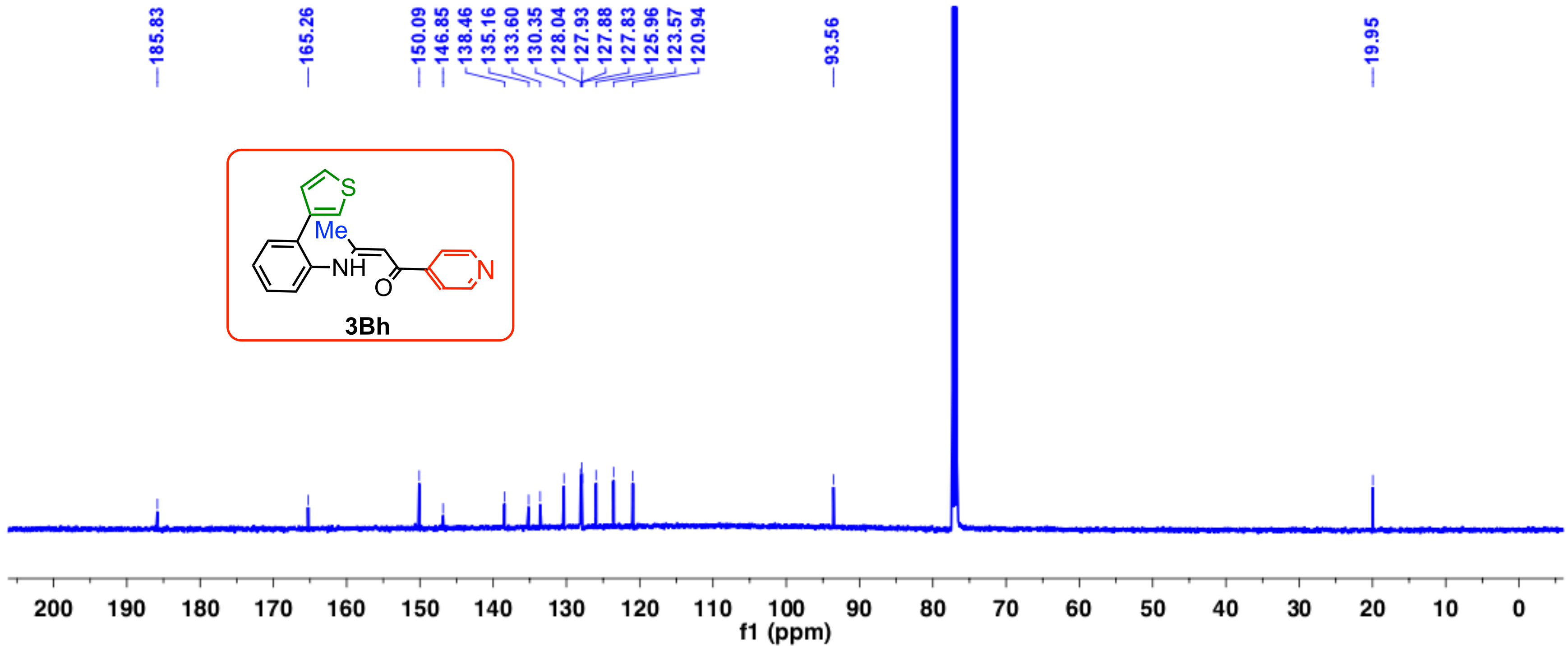

Figure S134: ^{13}C NMR of enaminone **3Bh**.

HRMS-ESI (m/z) ([M + H]$^+$):

Calculated: 321.1062

Observed: 321.1053

|Δm| = 2.8 ppm

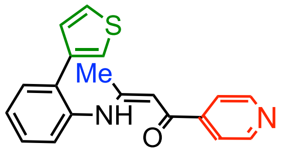

3Bh

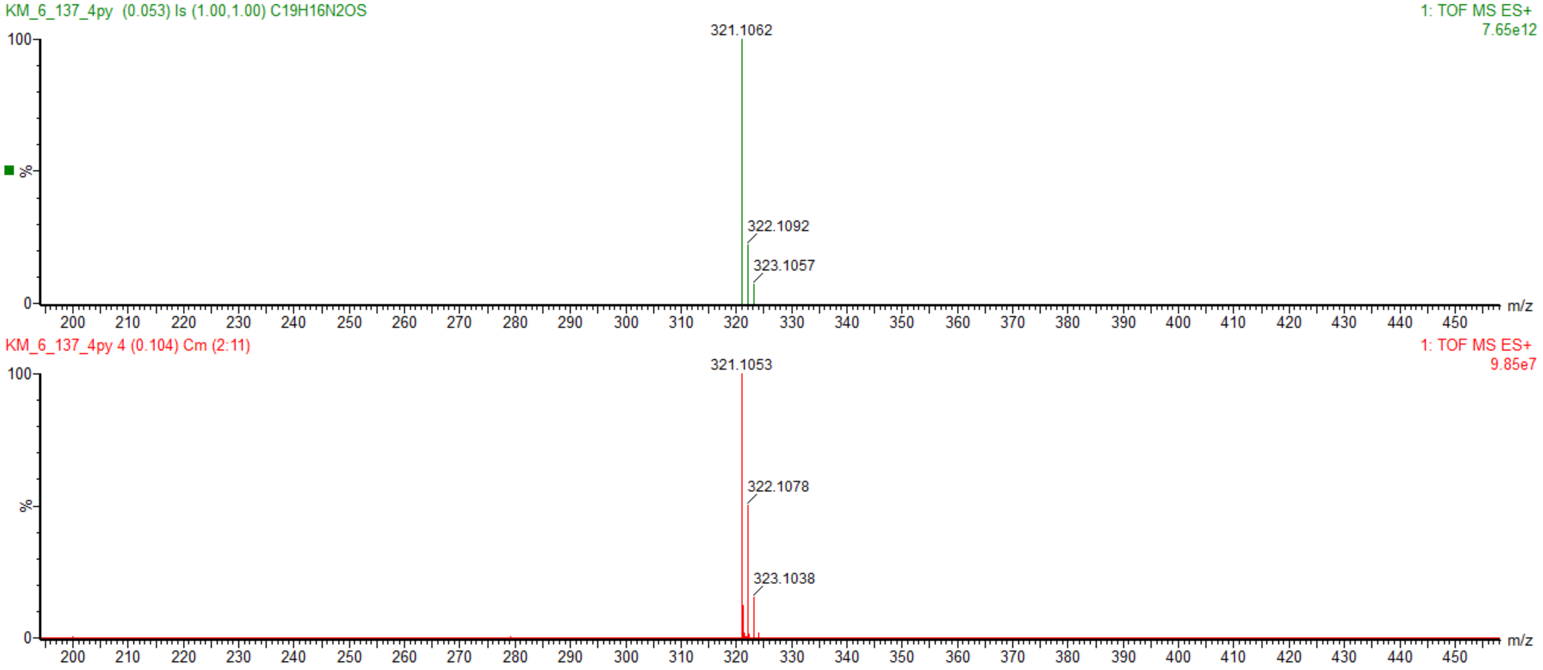

Figure S135: HRMS data of enaminone **3Bh**.

18.1. Characterization of enaminone **3Bi**.

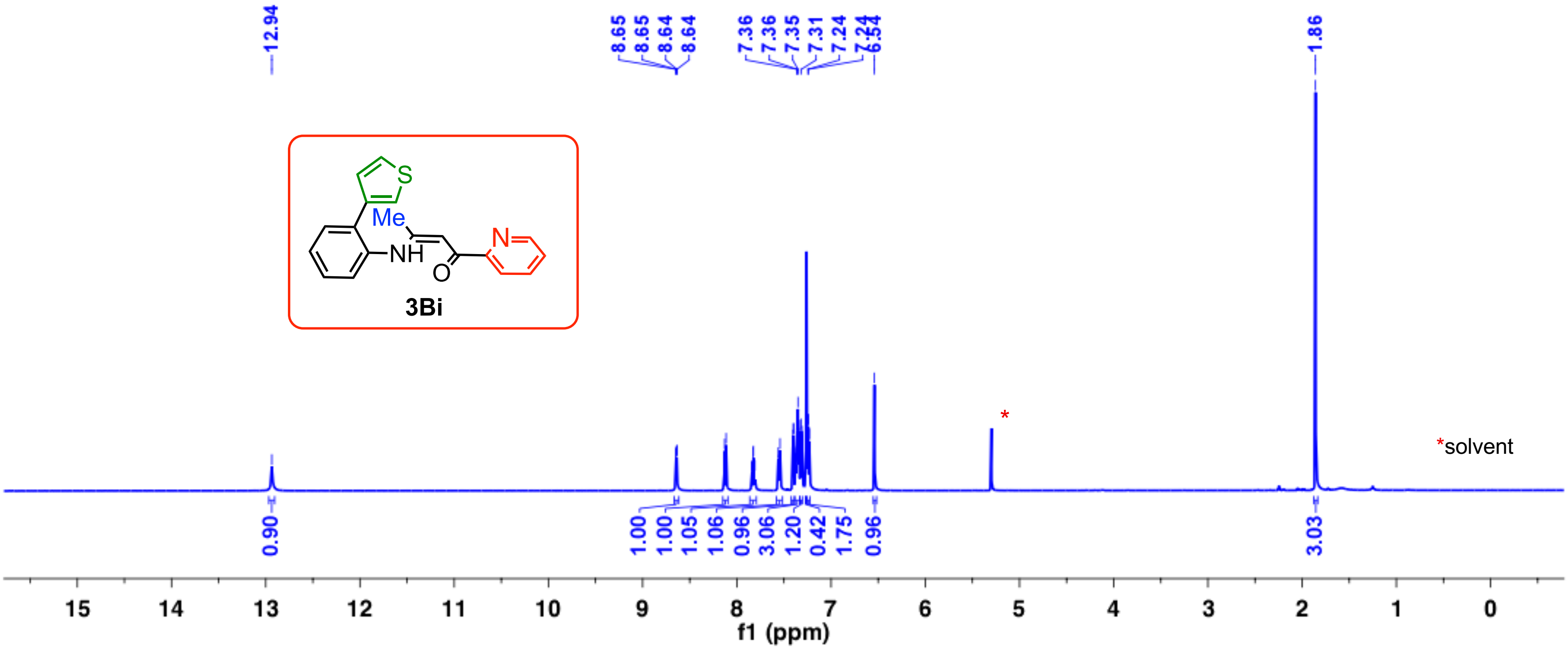

Figure S136: ^{1}H NMR of enaminone **3Bi**.

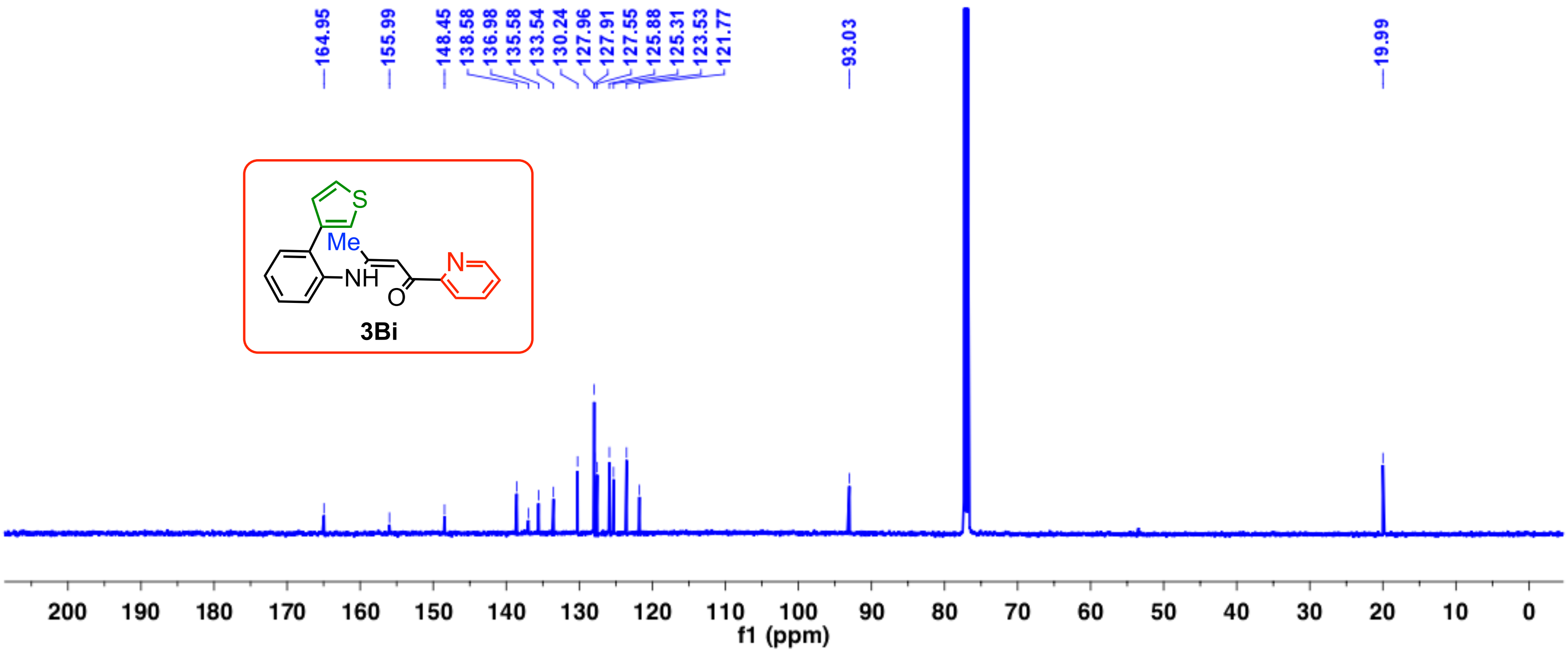

Figure S137: ^{13}C NMR of enaminone **3Bi**.

HRMS-ESI (m/z) ([M + H]$^+$):

Calculated: 321.1062

Observed: 321.1053

|Δm| = 2.8 ppm

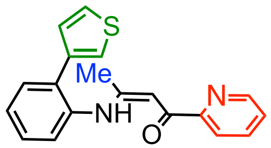

3Bi

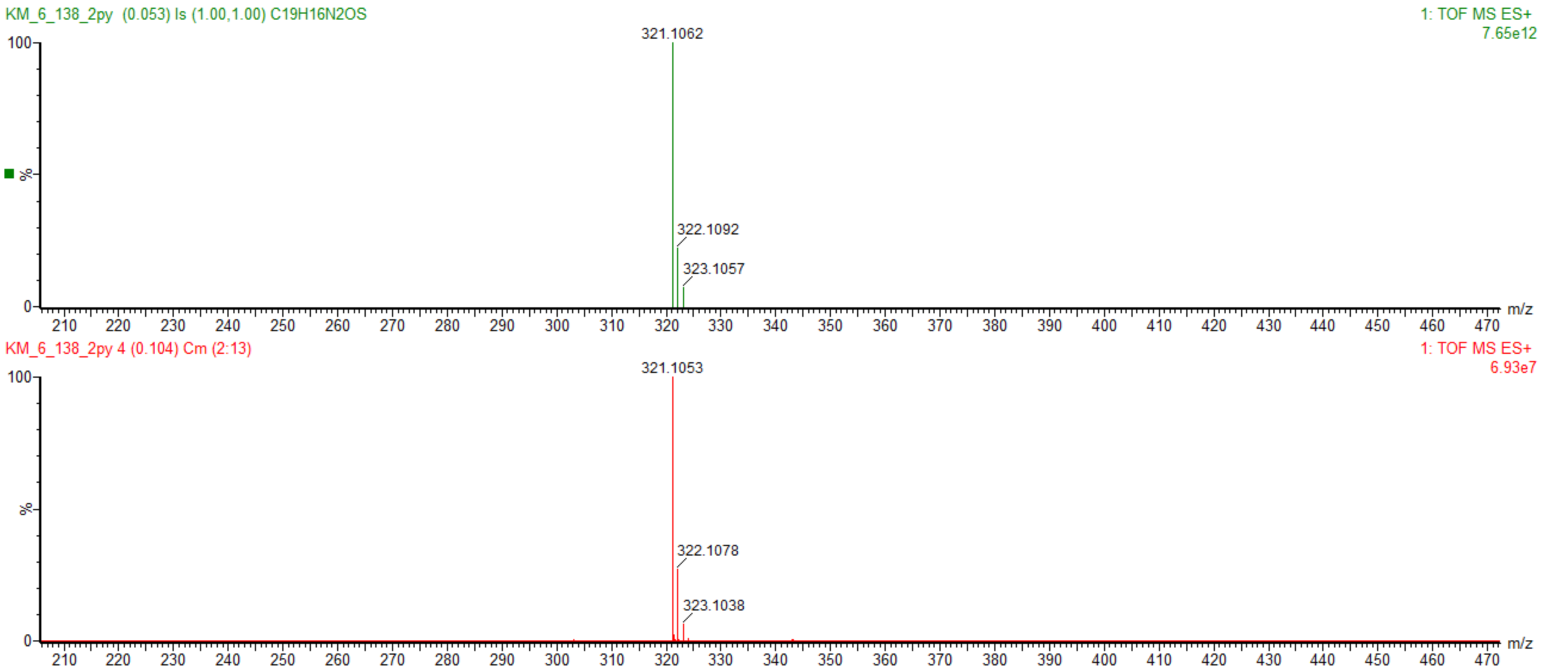

Figure S138: HRMS data of enaminone **3Bi**.

18.2. Characterization of enaminone **3Bj**.

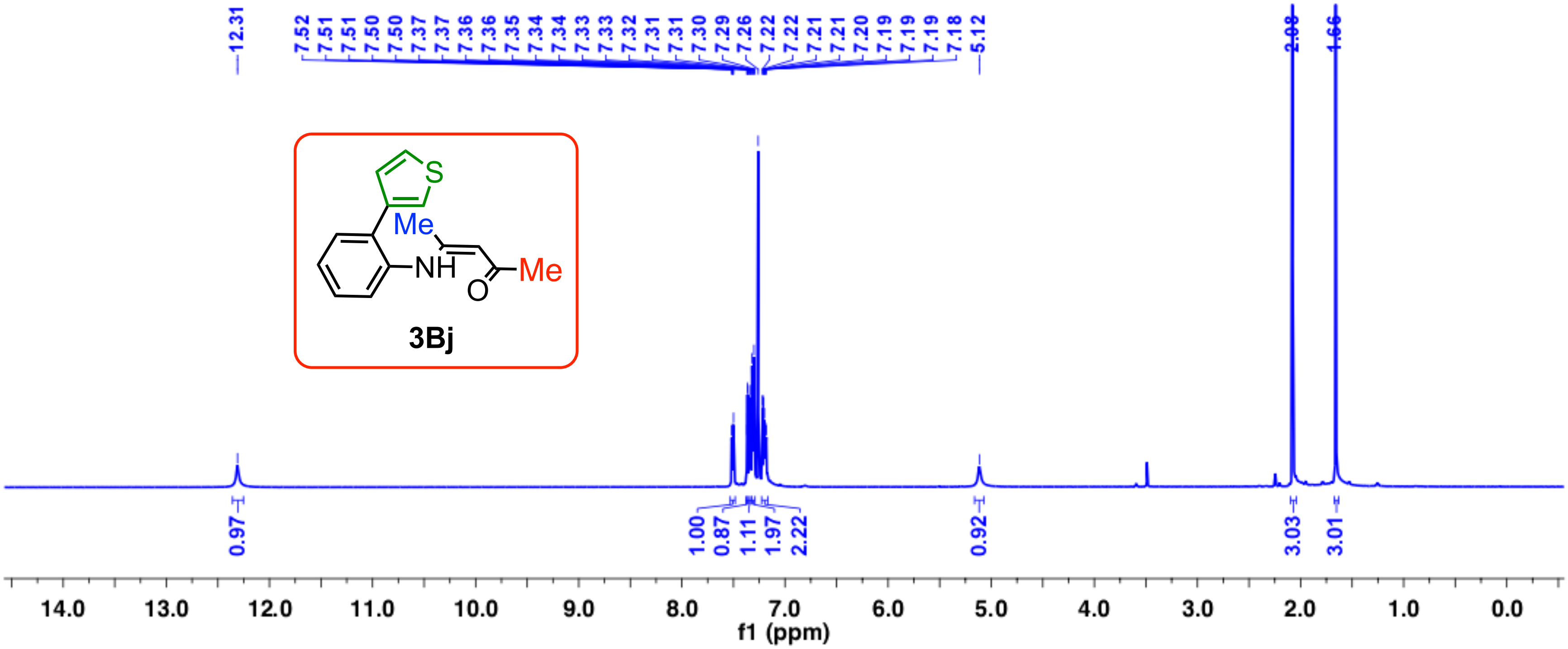

Figure S139: ^{1}H NMR of enaminone **3Bj**.

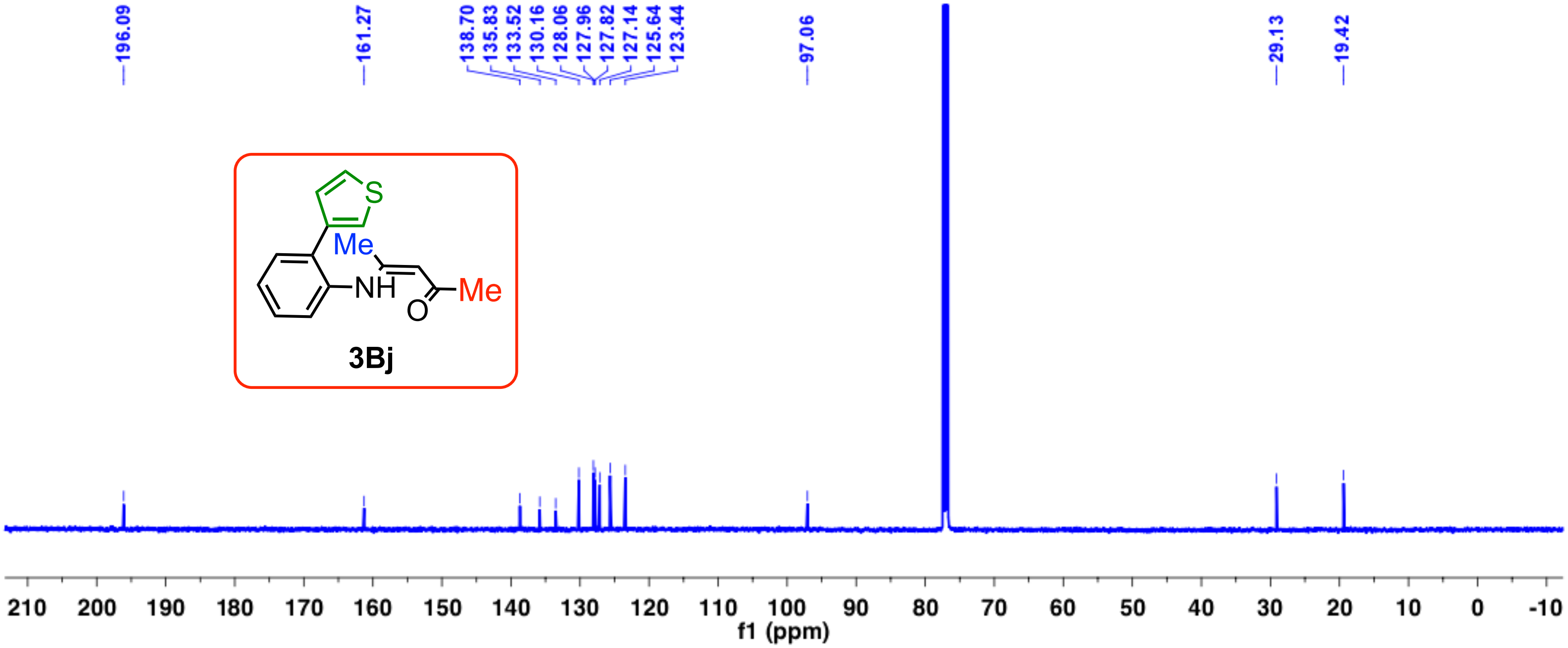

Figure S140: ^{13}C NMR of enaminone **3Bj**.

HRMS-ESI (m/z) ([M + H]$^+$):

Calculated: 258.0952

Observed: 258.0943

|Δm| = 3.48 ppm

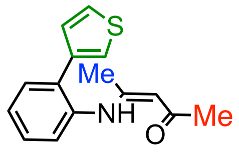

3Bj

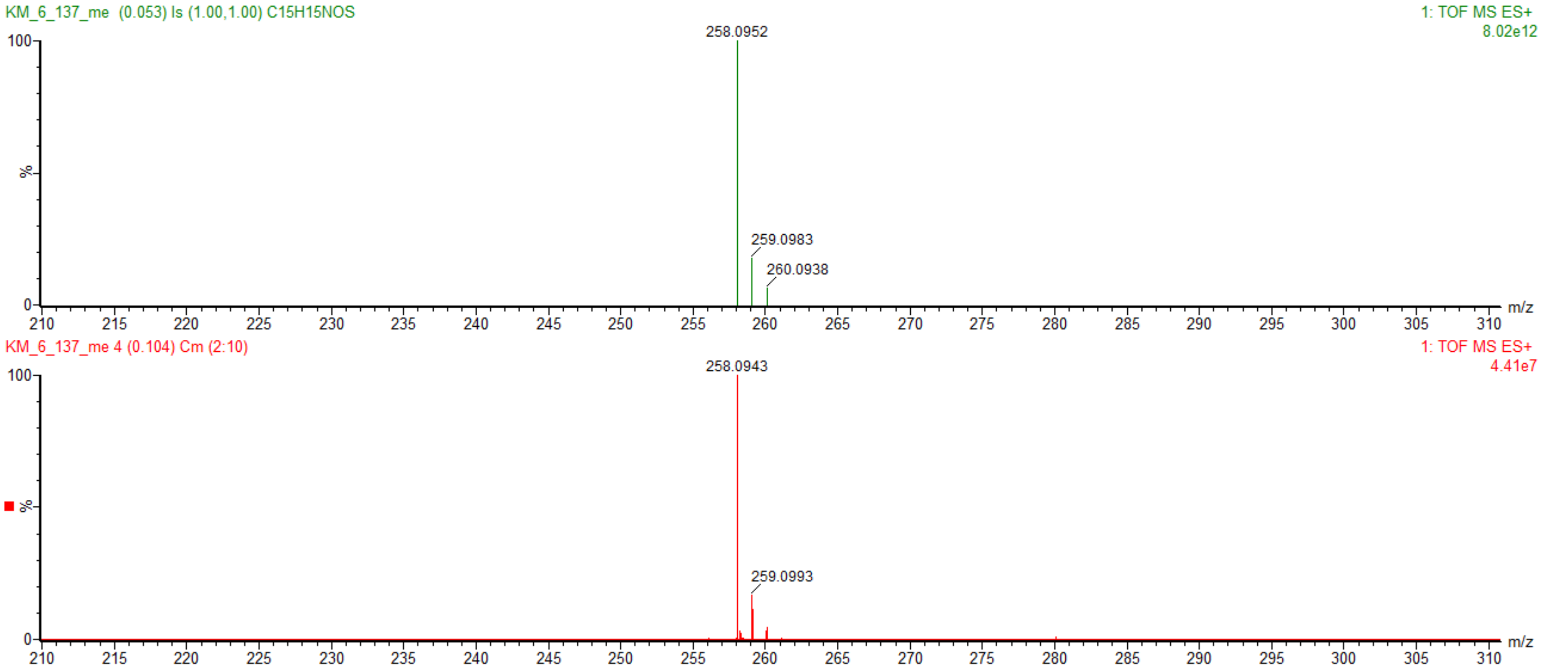

Figure S141: HRMS data of enaminone **3Bj**.

18.1. Characterization of enaminone **3Bk**.

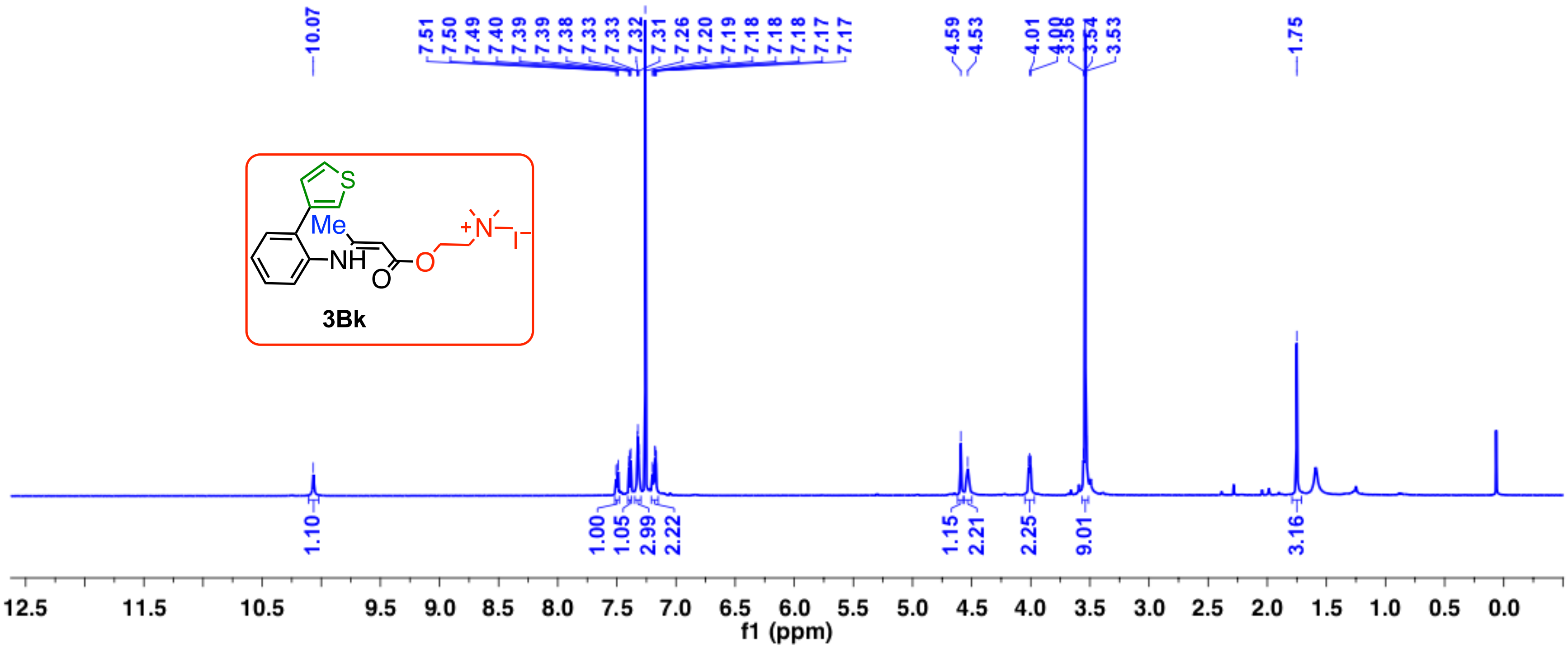

Figure S142: ^{1}H NMR of enaminone **3Bk**.

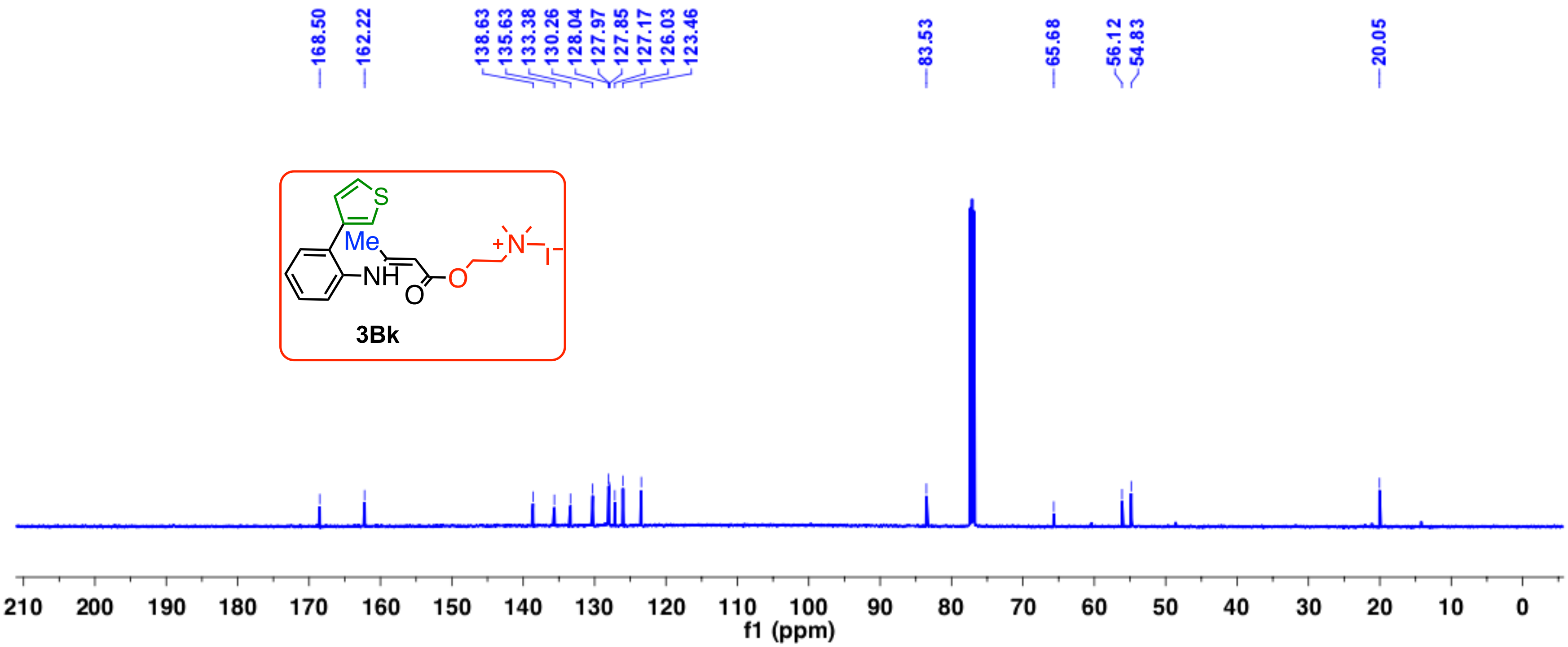

Figure S143: ^{13}C NMR of enaminone **3Bk**.

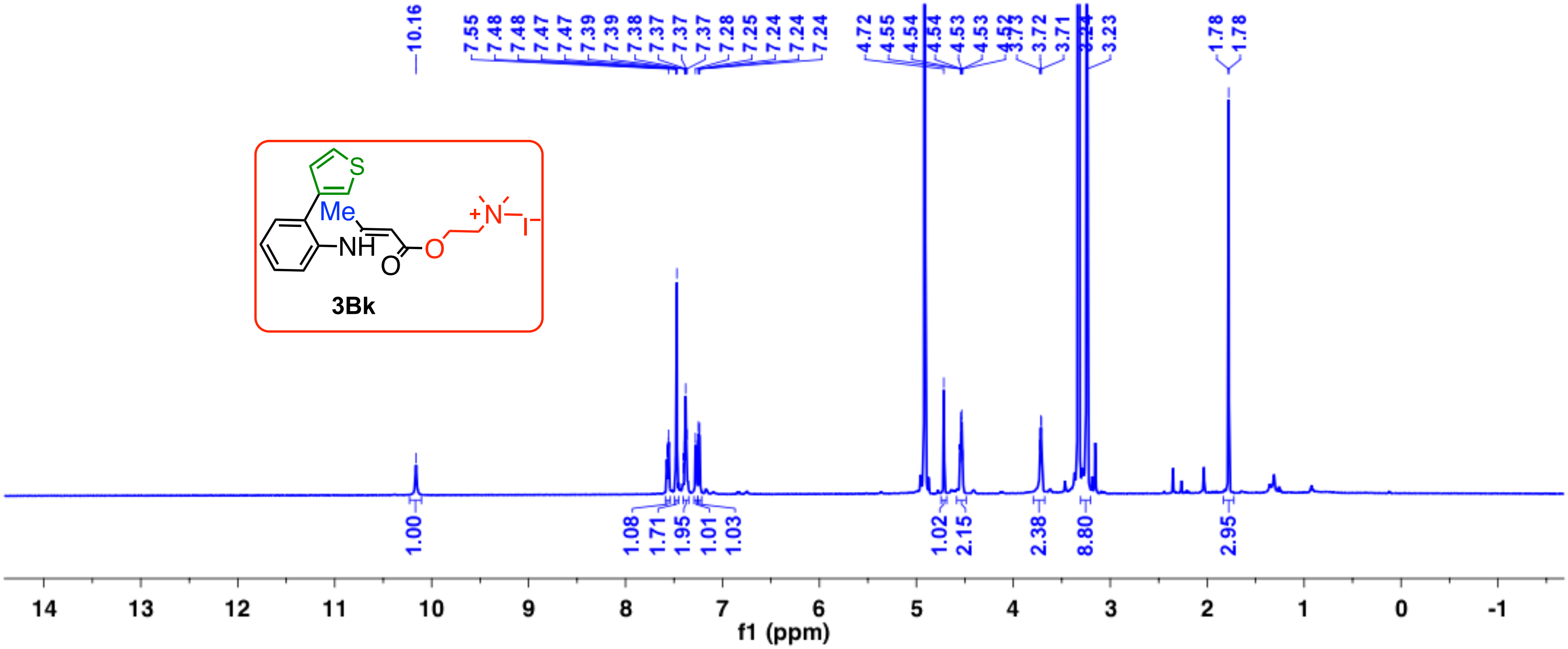

Figure S144: ^{1}H NMR of enaminone **3Bk** in CD_3OD.

HRMS-ESI (m/z) ([M]$^+$):

Calculated: 345.1637

Observed: 345.1635

|Δm| = 0.5 ppm

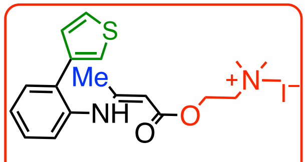

3Bk

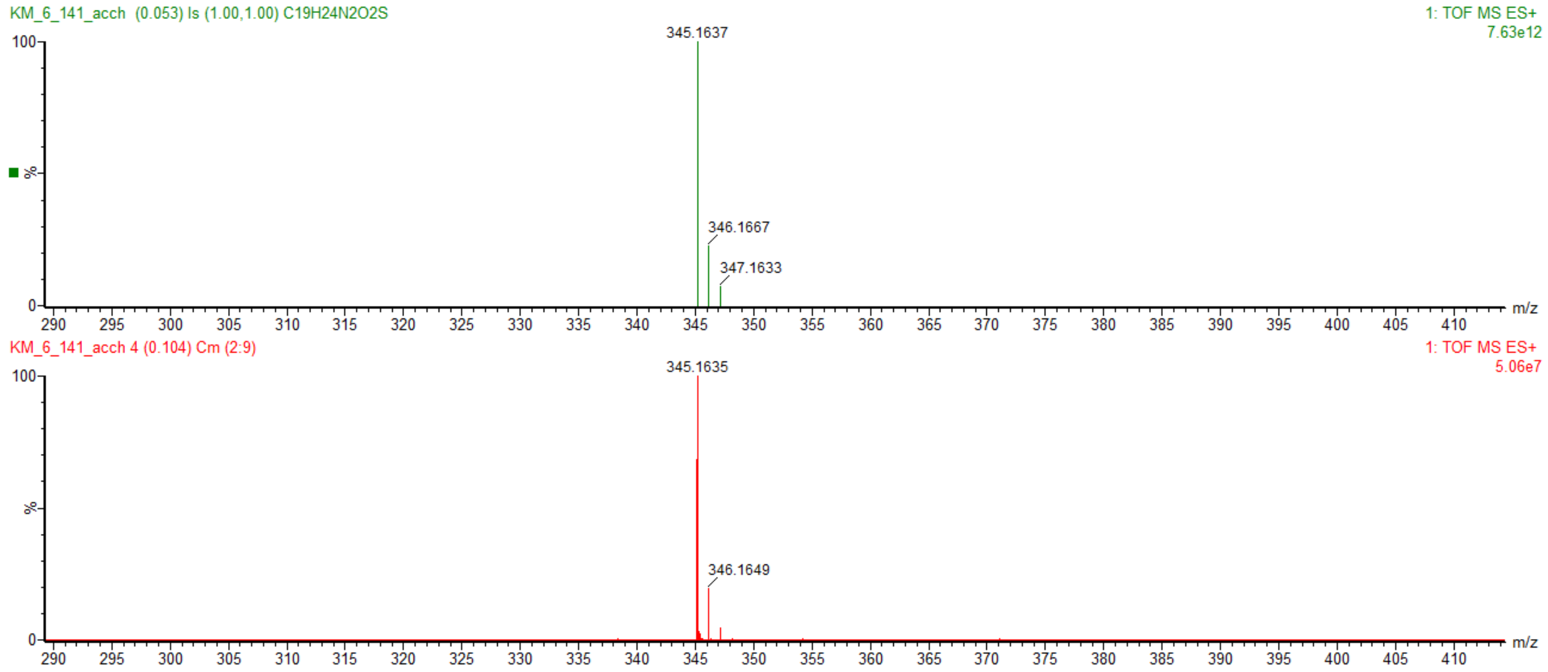

Figure S145: HRMS data of photoproduct **3Bk**.

18.1. Characterization of enaminone **3Ha**.

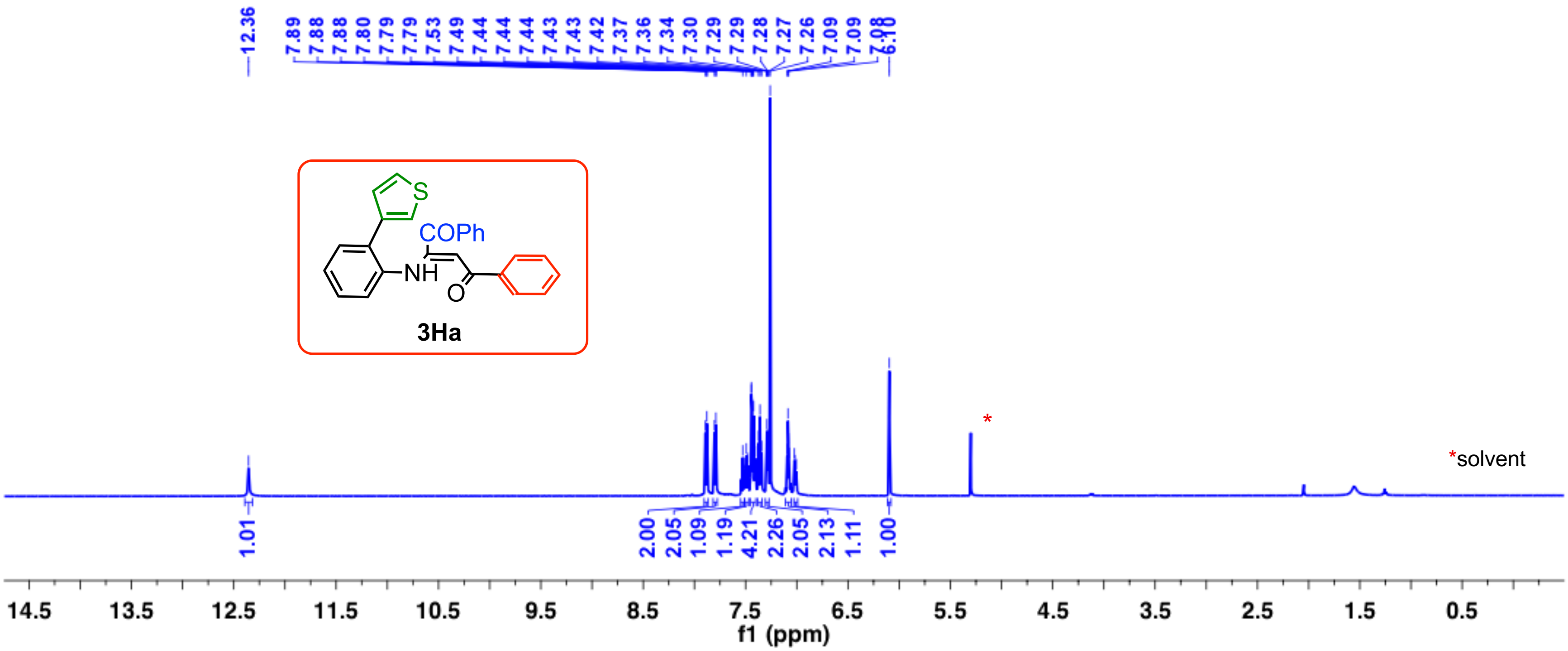

Figure S146: ^{13}C NMR of enaminone **3Ha**.

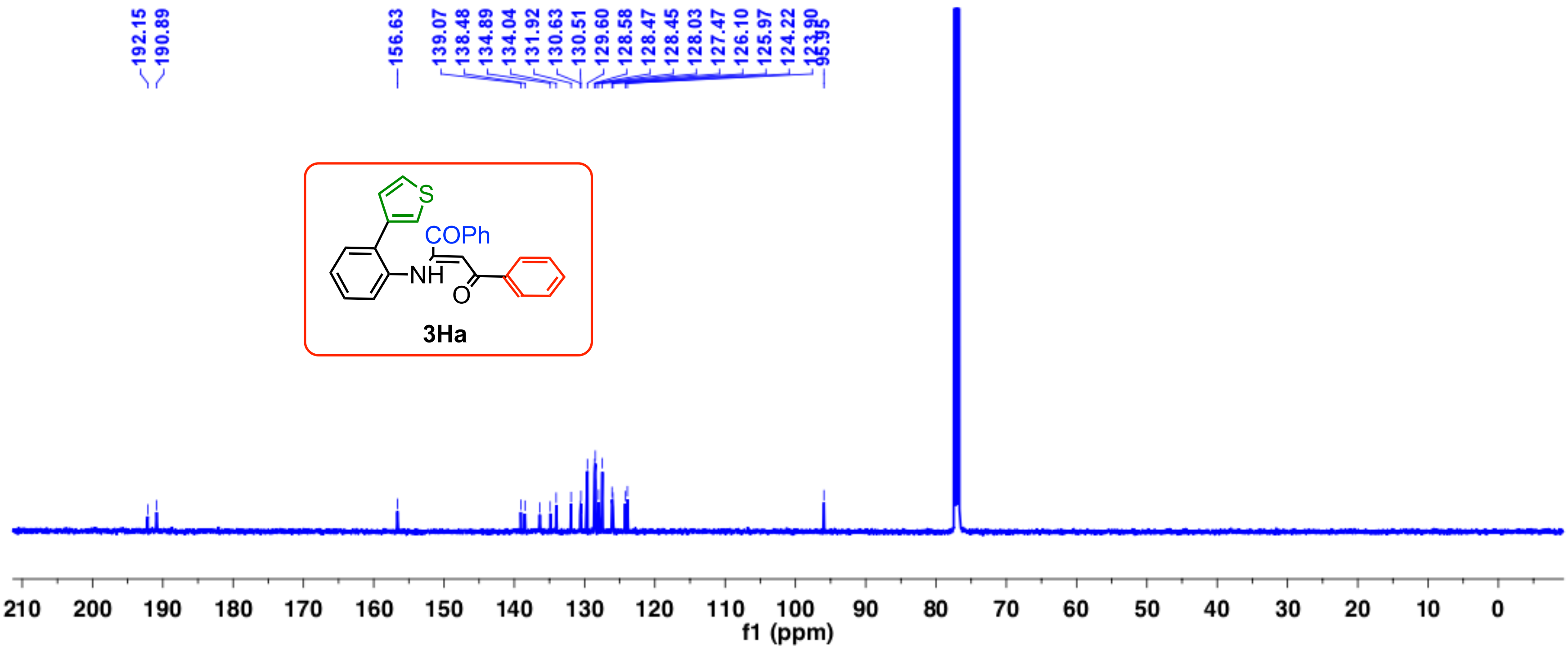

Figure S147: ^{1}H NMR of enaminone **3Ha**.

HRMS-ESI (m/z) ([M + H]$^+$):

Calculated: 410.1215

Observed: 410.1205

|Δm| = 2.4 ppm

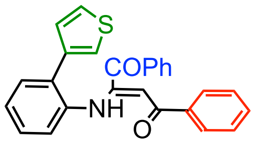

3Ha

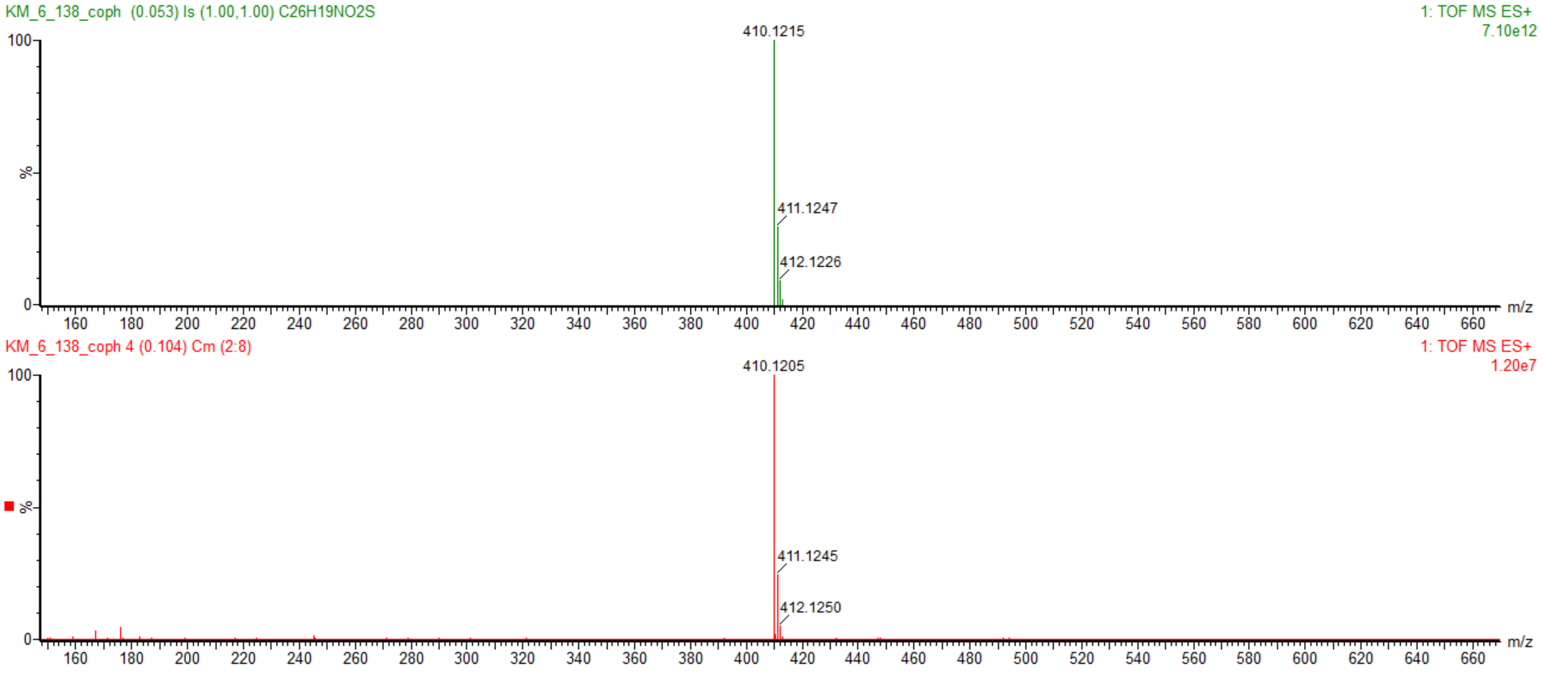

Figure S148: HRMS data of enaminone **3Ha**.

19. XRD analysis

Entry	**3Bf**	**3Bg**	**3Ha**	**4A**	**4B**
Formula	$C_{21}H_{16}N_2OS$	$C_{24}H_{19}NOS$	$C_{26}H_{19}NO_2S$	$C_{12}H_9NO$	$C_{12}H_9NS$
FW	344.42	369.46	409.48	183.20	199.26
cryst. size_max [mm]	0.2	0.47	0.32	0.61	0.37
cryst. size_mid [mm]	0.2	0.45	0.28	0.35	0.3
cryst. size_min [mm]	0.2	0.42	0.27	0.35	0.28
cryst. system	Monoclinic	Monoclinic	Monoclinic	Monoclinic	Monoclinic
Space group, Z	P 21/c	P 21/c	P 21/n	P 21/c	P 21/c
a [Å]	9.0869(4)	14.0383(14)	13.682(2)	4.6158(5)	4.9793(2)
b [Å]	26.0042(11)	11.7854(12)	9.4155(15)	14.6468(15)	13.5986(6)
c [Å]	7.5820(3)	12.6713(12)	17.255(3)	13.6398(14)	14.3455(7)
α	90	90	90	90	90
β	97.342(2)	113.761(4)	107.711(6)	92.712(5)	91.321(2)
γ	90	90	90	90	90
V[Å^3]	1776.92(13)	1918.7(3)	2117.4(6)	921.11(17)	971.10(7)
ρ_{calc} [g/cm^3]	1.287	1.279	1.285	1.321	1.363
μ [mm^{-1}]	1.692	1.588	1.530	0.678	2.565
Radiation type	CuKα (λ=1.54178)	CuKα (λ=1.54178)	CuKα (λ=1.54178)	CuKα (λ=1.54178)	CuKα (λ=1.54178)
F(000)	720.0	776.0	856.0	384.0	416.0
No of collected ref.	40924	43096	43582	15254	18411
No of independent ref.	3483	3771	4182	1821	1884
R1/wR2 (I≥2σ) [%]	0.0604/0.1857	0.0649/0.1975	0.0732/0.2424	0.0615/0.1668	0.0401/0.1055
R1/wR2 (all data) [%]	0.1240/0.1990	0.1057/0.2160	0.0990/0.2575	0.1302/0.1853	0.0588/0.1118

19.1. Structure of enaminone **3Bf**.

Enaminone **3Bf** was crystallized in methanol, by slow evaporation.

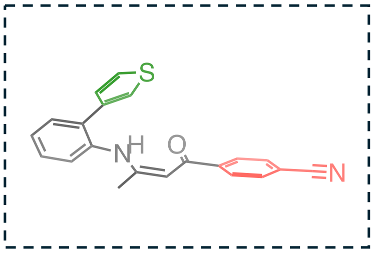

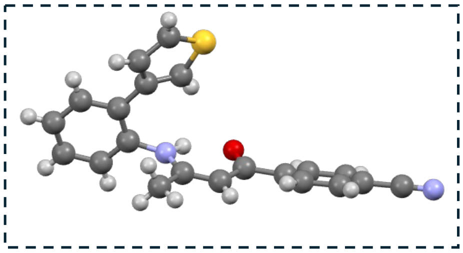

19.2. Structure of enaminone **3Bg**.

Enaminone **3Bg** was crystallized in methanol, by slow evaporation.

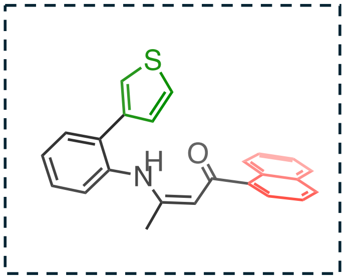

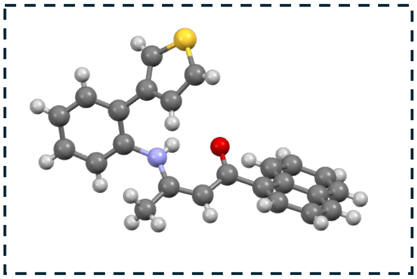

19.3. Structure of enaminone **3Ha**.

Enaminone **3Ha** was crystallized in methanol, by slow evaporation.

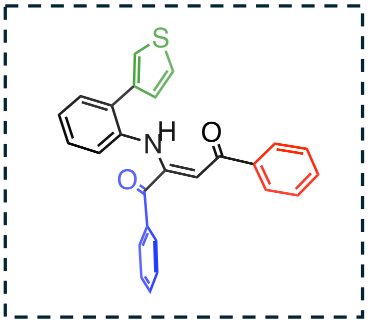

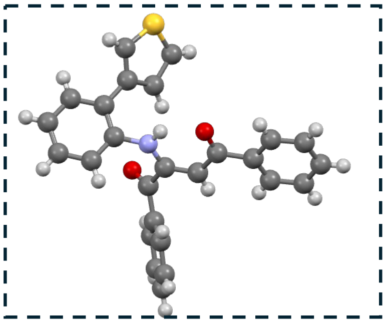

19.4. Structure of quinoline photoproduct **4A**.

Quinoline photoproduct **4A** was crystallized in methanol, by slow evaporation.

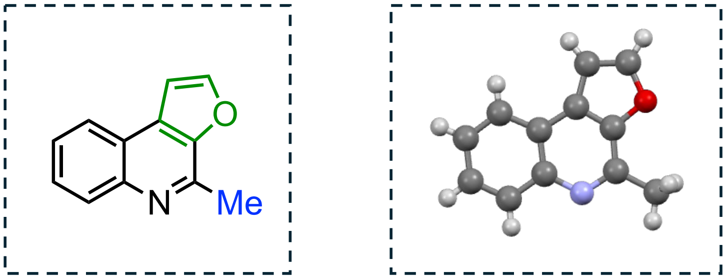

19.5. Structure of quinoline photoproduct **4B**.

Quinoline photoproduct **4B** was crystallized in methanol/ hexane solvent mixture, by slow evaporation.

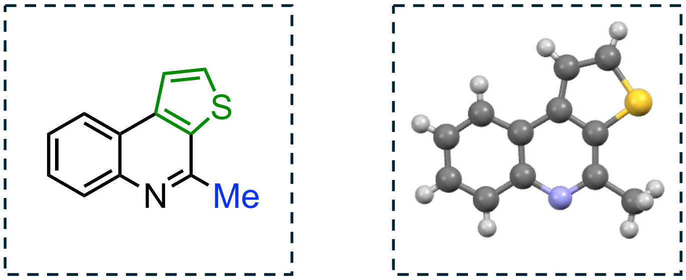

20. References.

1. Inagaki, S.; Saito, K.; Suto, S.; Aihara, H.; Sugawara, A.; Tamura, S.; Kawano, T., Synthesis of 5-Aryl-3(2 H)-furanones Using Intramolecular Cyclization of Sulfonium Salts. *J. Org. Chem.* **2018,** *83*, 13834-13846.
2. Dharma Rao, G. B.; Acharya, B. N.; Kaushik, M. P., An efficient synthesis of β-ketoesters via transesterification and its application in Biginelli reaction under solvent-free, catalyst-free conditions. *Tetrahedron Lett.* **2013,** *54*, 6644-6647.
3. Acharya, B. N.; Rao, G. B. D.; Kumar, D.; Kumar, P.; Kaushik, M. P., Design, synthesis, and evaluation of dihydropyrimidinone (DHPM) based muscarinic receptor antagonist. *Med. Chem. Res.* **2015,** *24*, 1763-1775.
4. Zheng, C.; Zang, Q.; Nie, H.; Huang, W.; Zhao, Z.; Qin, A.; Hu, R.; Tang, B. Z., Fluorescence visualization of crystal formation and transformation processes of organic luminogens with crystallization-induced emission characteristics. *Materials Chem. Frontiers* **2018,** *2*, 180-188.
5. Akula, M.; Yogeeswari, P.; Sriram, D.; Jha, M.; Bhattacharya, A., Synthesis and anti-tubercular activity of fused thieno-/furo-quinoline compounds. *RSC Advances* **2016,** *6*, 46073-46080.
6. Thorve, P. R.; Maji, B., Synthesis of Furo- and Thienoquinolines by Using an Amine Oxidase-Inspired Catalyst. *Synlett* **2023,** *34*, 2433-2438.
7. Tymann, D.; Tymann, D. C.; Bednarzick, U.; Iovkova-Berends, L.; Rehbein, J.; Hiersemann, M., Development of an alkyne analogue of the de Mayo reaction: synthesis of medium-sized carbacycles and cyclohepta [b] indoles. *Angew. Chem. Int. Ed.* **2018,** *57*, 15553-15557.
8. Adib, M.; Mahdavi, M.; Abbasi, A.; Jahromi, A. H.; Bijanzadeh, H. R., Efficient synthesis of imidazo [1, 2-a] pyridin-3 (2H)-ones. *Tetrahedron Lett.* **2007,** *48*, 3217-3220.
9. Morris, J. V.; Mahaney, M. A.; Huber, J. R., Fluorescence quantum yield determinations. 9, 10-Diphenylanthracene as a reference standard in different solvents. *J. Phys. Chem.* **1976,** *80*, 969-974.
10. Brouwer, A. M., Standards for photoluminescence quantum yield measurements in solution (IUPAC Technical Report). *Pure App. Chem.* **2011,** *83*, 2213-2228.
11. Hatchard, C. G.; Parker, C. A., A new sensitive chemical actinometer - II. Potassium ferrioxalate as a standard chemical actinometer. *Proc. R. Soc. Lond. A. Math. Phys. Sci.* **1956,** *235*, 518-536.
12. Murov, S., L., *Handbook of Photochemistry*. Marcel Dekker: New York, 1973;
13. Rabani, J.; Mamane, H.; Pousty, D.; Bolton, J. R., Practical chemical actinometry—a review. *Photochem. Photobiol.* **2021,** *97*, 873-902.
14. Dyer, J.; Jockusch, S.; Balsanek, V.; Sames, D.; Turro, N. J., Two-photon induced uncaging of a reactive intermediate. Multiphoton in situ detection of a potentially valuable label for biological applications. *J. Org. Chem.* **2005,** *70*, 2143-2147.
